# Collaborative passive cooling of impact-hardening interfaces enabled by nacre-mimetic design

**DOI:** 10.1038/s41467-026-74755-w

**Published:** 2026-06-22

**Authors:** Zimu Li, Sheng Wang, Shuai Liu, Jianpeng Wu, Wenhui Wang, Zhentao Zhang, Shilong Duan, Liangyuan Qi, Yuan Hu, Xinglong Gong

**Affiliations:** 1 CAS Key Laboratory of Mechanical Behavior and Design of Materials, Department of Modern Mechanics, University of Science and Technology of China, Hefei, PR China; 2 State Key Laboratory of Fire Science, University of Science and Technology of China, Hefei, PR China

**Keywords:** Bioinspired materials, Mechanical properties, Composites

## Abstract

Balancing thermal management with mechanical buffering is critical for protecting outdoor devices and expanding their application scenarios. Here we propose a nacre-mimetic strategy that synergistically improves passive cooling and impact resistance through brick-and-mortar component regulation, surpassing numerous advanced high-performance composites. Dynamic crosslinking within the composition imparts non-absorption in specific spectral bands and strain-rate-dependent impact hardening. The as-designed composite exhibits a thermal anisotropy ratio of 44.47 and remains nonflammable under an 873 K flame for 1 h, releasing low-carbon gaseous products. It achieves solar reflectance and mid-infrared emittance of 0.97 at 393 K, translating to urban cooling energy savings exceeding 40%. The composite resists projectile penetration at 50 m s^−1^, and closed-loop recycling retains thermo-mechanical performance comparable to the pristine counterpart. Building on these attributes, we develop a thermo-mechanically coupled protective sandwich configuration featuring high volume resistivity and a low dielectric constant. This design delivers a maximum cooling effect of 20.5 K and dissipates 97.90% of the kinetic impact force in overheated outdoor devices. Life-cycle assessment quantifies a low environmental footprint. Collectively, this nacre-inspired paradigm illustrates sustainable multi-physics coupling management and holds strong promise for safeguarding outdoor devices in extremely harsh environments.

## Abstract (web summary)

This study presents a nacre-inspired material that passively cools outdoor devices while absorbing impacts, offering recyclable, low-carbon protection for electronics and equipment in harsh environments.

## Introduction

The marked improvements in the performance of outdoor devices have sharply increased power density and heat flux, in turn elevating the energy demand for thermal management^1–4^. In alignment with the goals of green energy conservation and carbon neutrality, there is therefore an urgent need for sustainable thermal management solutions^5–7^. Passive cooling technologies, which operate without external energy input, enable temperature reduction without electrical power or mechanical actuation^8–10^. Current approaches are dominated by two strategies. The first is conductive cooling, which employs high-thermal-conductivity composites to spread and dissipate heat via conduction. The second is radiative cooling, which uses spectral engineering to achieve high solar reflectance (0.3–2.5 μm) and high thermal emissivity in the atmospheric transparency window (8.0–13.0 μm), thereby reflecting solar irradiance and radiating heat directly to outer space. Owing to their energy autonomy, these technologies show substantial promise in building-envelope energy conservation, off-grid electronics thermal management, and wearable personal thermal comfort^11–13^.

However, outdoor systems such as vehicles and communication base stations are continually subjected to stochastic high-frequency dynamic loads, including hail impacts and mechanical vibrations^14–16^. Conventional composites are largely static, lacking strain-rate sensitivity and adaptive thermal response, and thus cannot simultaneously satisfy the dual requirements of mechanical protection and thermal management. Their properties are fixed after fabrication and cannot dynamically respond to changing environments. It is therefore essential to develop advanced material systems capable of simultaneously delivering efficient impact energy absorption and heat dissipation. Shear-stiffening gel (SSG) is an exemplary strain-rate-sensitive smart material that exhibits viscous flow under quasi-static loading^17,18^. Under high-speed impact, dynamic crosslinking within their molecular chain networks becomes rapidly entangled and strengthened because the relaxation time far exceeds the loading timescale, causing the storage modulus to increase by up to three orders of magnitude. This impact-hardening effect enables substantial attenuation of kinetic energy. For instance, Wu et al.^19^ fabricated an SSG-based aramid composite that reduced impact force by 65–79%. Engineering SSG also enables multi-functional integration of impact mitigation and thermal management. Zhang et al.^20^ reported an SSG-based glass with simultaneous improvements in thermal insulation and impact resistance. Nevertheless, simple incorporation of cooling particles tends to degrade the dynamic response of SSG, creating cross-scale co-design challenges in integrating mechanical protection with passive cooling. The central task, therefore, is to overcome the material’s intrinsic limitations while preserving its existing performance, and to establish continuous conduction and radiation pathways to ensure cooling efficiency.

Biological materials with multi-level heterogeneous architectures offer a compelling paradigm for designing high-performance systems and addressing coupled functional challenges^21–23^. Among these, nacre-inspired designs, featuring hierarchical brick-and-mortar assemblies, have emerged as a model for integrating thermal and mechanical functions^24,25^. The anisotropic architecture, formed by alternating long-range ordered thermal conduction microplatelets and organic interfaces, constructs continuous phonon transport channels in the plane, enabling efficient directional heat dissipation. For example, Han et al.^26^ developed a nacre-mimetic boron nitride nanosheet (BNNS)/epoxy network for conductive cooling with a thermal conductivity of 6.07 W m^−1^ K^−1^ and high electrical resistivity. Ma et al.^27^ prepared a hierarchical aerogel for radiative cooling, highlighting its potential for energy-efficient and sustainable applications. Concurrently, impact energy dissipation is enhanced via mechanisms such as crack deflection at organic/inorganic interface, layer slippage, and mortar deformation. Yin et al.^28^ fabricated a nacre-like transparent glass whose impact resistance surpasses that of monolithic and laminated glasses, underscoring its suitability for outdoor devices. Inspired by these bio-informed strategies, it is essential to devise a multi-level nacre-inspired model featuring zero-energy cooling and tunable mechanical protection to address the thermo-mechanical coupling protection challenges encountered by outdoor devices in extreme environments.

In this work, we propose a nacre-mimetic composite that synergistically integrates passive cooling with impact mitigation. The highly ordered brick network maximizes solar reflectance and facilitates outward emission of intrinsic thermal energy. Internal dynamic crosslinking imparts both selective non-absorption across the solar spectrum and impact hardening. This architecture establishes efficient heat transport channels that draw heat away from the device interface, while the composition and structure resist impact loads, dissipate kinetic energy, and safeguard the underlying system. Ultimately, we demonstrate an ingeniously configured sandwich structure that combines practicality with sustainability, paving the way for next-generation protective materials capable of robust thermo-mechanical coupling.

## Results

### Nacre-mimetic design

**Fig. 1:**
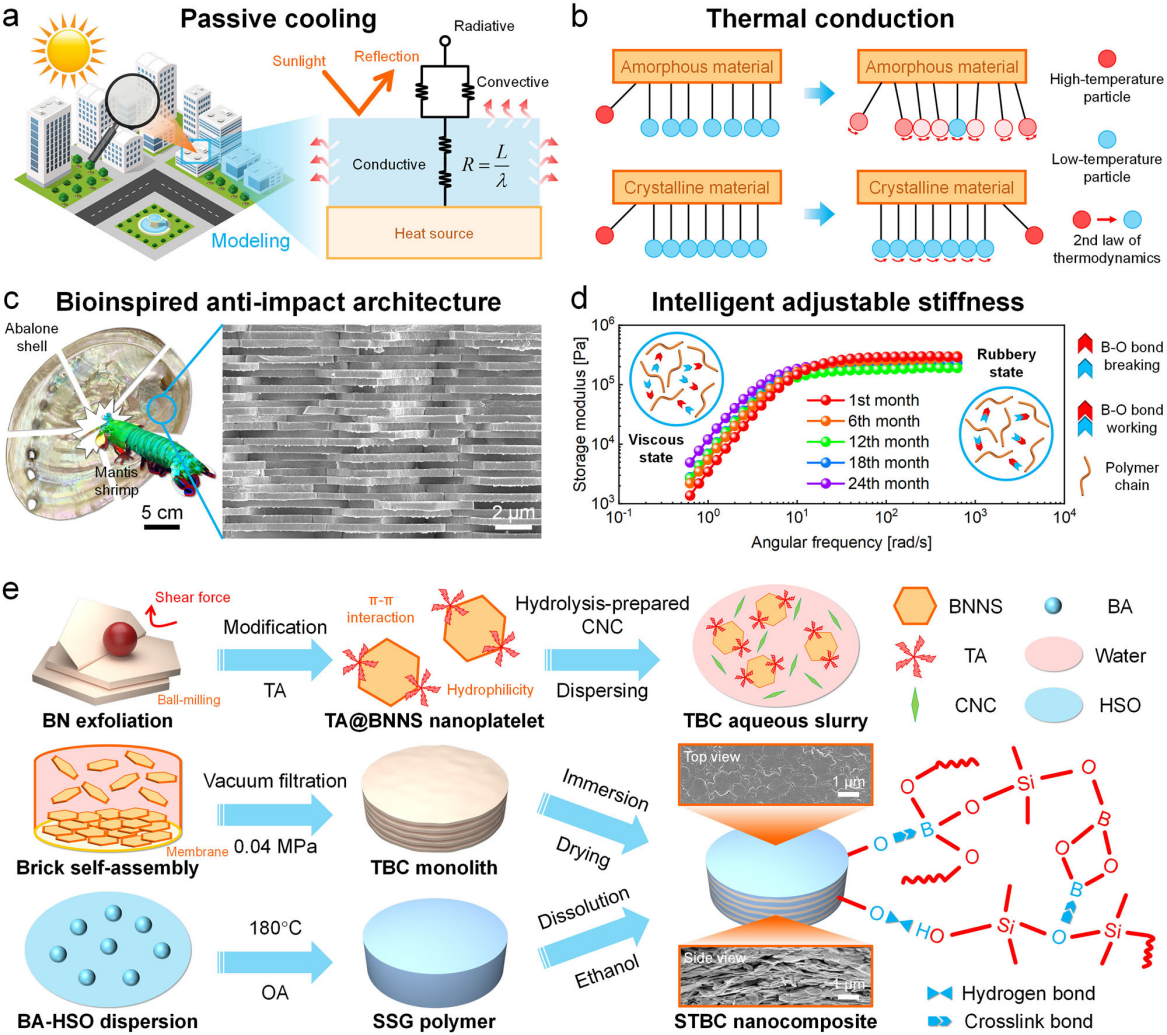
Nacre-inspired design and multi-functional integration. **a** Passive cooling model for urban heat islands (*R*: thermal resistance, *L*: thickness, *λ*: thermal conductivity). **b** Thermal conduction efficiency of amorphous versus crystalline materials. **c** Impact-resistant brick-and-mortar configuration inspired by nacre. **d** Intelligent tunable stiffness and long-term stability of SSG. **e** Preparation of SSG-TBC (STBC) nanocomposites based on a nacre-mimetic design (BN boron nitride, TA tannic acid, BNNS BN nanosheet, CNC cellulose nanocrystal, TBC TA@BNNS-CNC, BA boric acid, HSO hydroxyl silicone oil, OA octanoic acid, SSG shear-stiffening gel). The images were created using 3ds Max 2022 and Microsoft PowerPoint 2016. Source data are provided as a Source Data file.

The urban heat island effect was a pervasive and increasingly severe environmental challenge associated with modern urbanization, leading to elevated ambient temperatures, increased energy consumption, and worsened pollution^29,30^. Passive cooling strategies have been proven effective in mitigating this issue by reducing external heat input and increasing internal heat dissipation^5,12^. This work adopted a passive cooling model that reduced solar absorption while promoting the release of accumulated heat (Fig. 1a). Traditional building envelope materials, such as cement, brick, and concrete, were predominantly amorphous. As illustrated by the Newton’s cradle analogy (Fig. 1b), thermal excitation induced disordered oscillations in amorphous materials, with energy dissipating through scattering at defects and along polymer chains. In contrast, crystalline materials, owing to their long-range atomic order, transferred heat efficiently between particles with reduced scattering and energy loss^31,32^. This structural strategy helped circumvent the intrinsic thermal-resistance limitations of amorphous matrices, enabling efficient directional heat transfer. Incorporating ordered configurations into cooling materials could therefore substantially improve heat-transfer efficiency.

Frequent extreme events demanded materials with exceptional impact resistance. The brick-and-mortar structure of nacre was a quintessentially ordered architecture capable of withstanding aggressive attacks, such as those from mantis shrimp (Fig. 1c). Under significant impact loads, interfacial features redirected and redistributed stresses over larger areas, deflecting cracks from their original paths. The organic matrix absorbed energy and inhibited crack penetration^24,28^. SSG, a high-molecular-weight polyborosiloxane, was renowned for excellent impact resistance and pronounced rate-sensitive mechanical behavior (Fig. 1d). Under high strain-rate loading, molecular chains entangled and dynamic boron-oxygen (B-O) bonds lacked sufficient time to break, yielding a rubbery shear-stiffened state^18,33^. 24-month periodic strain-rate sweep tests confirmed the temporal stability of the dynamic B-O network, showing negligible modulus change and consistent stiffening behavior. Accordingly, combining functional brick elements with impact-hardening mortar could synergistically enhance impact resistance while imparting multifunctionality.

Guided by these principles, we developed a nacre-mimetic nanocomposite that integrated passive cooling with impact resistance, employing boron nitride (BN) and SSG as key constituents (Fig. 1e and Supplementary Fig. 1). BN was exfoliated via shear-assisted ball milling and modified with tannic acid (TA) to obtain TA@BNNS nanoplatelets. π-π interactions between TA and BNNS ensured interfacial adhesion, while TA functionalization converted hydrophobic BNNS into hydrophilic counterparts^34,35^. Cellulose nanocrystals (CNCs), prepared via cellulose hydrolysis, were incorporated to enhance overall thermo-mechanical performance^36,37^. During vacuum filtration^25,38^, the plate-like nanoplatelets preferentially oriented parallel to the filter surface under the combined influence of fluid shear and pressure normal to the membrane, self-assembling into long-range ordered stacks. CNCs interwoven between layers to form a TA@BNNS-CNC (TBC) scaffold featuring an interpenetrating 1D/2D network. Concurrently, boric acid (BA) and hydroxyl silicone oil (HSO) were dynamically crosslinked at elevated temperature in the presence of octanoic acid (OA) to produce SSG. SSG was then infiltrated into the TBC scaffold using ethanol as a carrier (Supplementary Fig. 2). Integrating the TBC brick with the SSG mortar yielded the nacre-inspired SSG-TA@BNNS-CNC (STBC) nanocomposite featuring an organic-inorganic dual network (Supplementary Fig. 3). These nanocomposites possessed abundant dynamic B-O bonds and interfacial hydrogen bonds. The hybrid architecture endowed the systems with anisotropic thermal transport, passive radiative cooling, and kinetic impact protection, thereby overcoming the functional fragmentation and performance trade-offs that commonly limited conventional composites.

### Anisotropic thermal transport

**Fig. 2:**
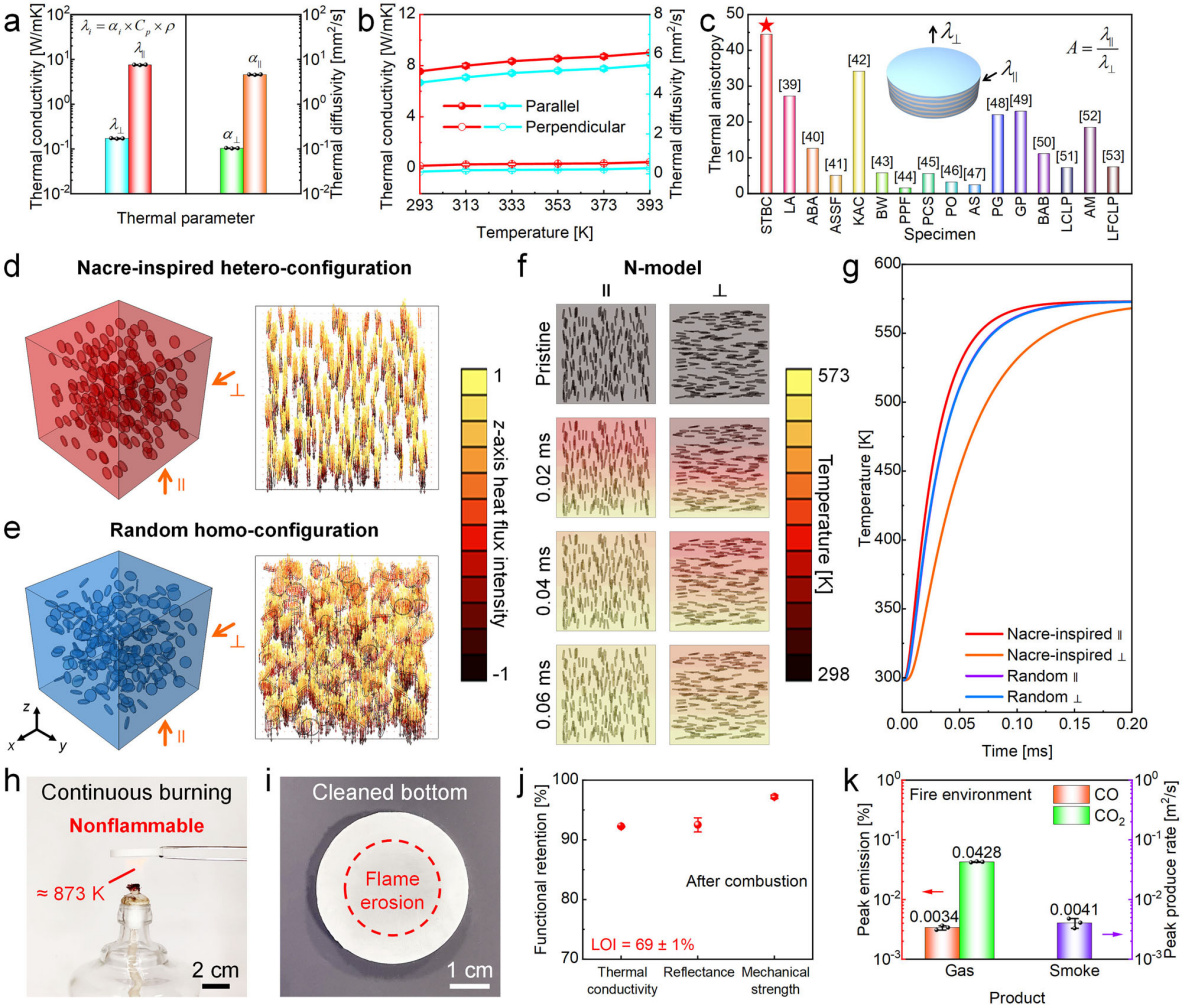
Thermal anisotropy and flame retardancy of STBC. **a** Thermal conductivity (*λ*_*i*_) and diffusivity (*α*_*i*_) of SSG-TA@BNNS-CNC (STBC) in the parallel (∥) and perpendicular (⊥) directions, and **b** their temperature-dependent profiles (*C*_p_: specific heat, *ρ*: density). **c** Thermal anisotropy (*A*, unitless) of STBC compared with reported thermal interface materials^39–53^. **d** Nacre-inspired hetero-configuration (N-model) and **e** random homo-configuration (R-model) models, with corresponding *z*-axis heat-flux intensity (unitless) maps at 0.01 ms. **f** Time-resolved temperature maps of the N-model in the parallel and perpendicular directions. **g** Temperature-time traces at differential elements for nacre-inspired and random structures in both directions. **h** Flame-impact resistance of STBC. **i** Bottom surface morphologies of STBC after combustion. **j** Retention of functional performance in STBC after flame impact. **k** Emission products of STBC under fire conditions. The error bars in (**a**, **b**, **j**, **k**) represent the standard deviations from three independent experiments. Source data are provided as a Source Data file.

In passive cooling, heat conduction was critical for efficiently transferring heat from overheated devices to the ambient environment. The heterogeneous architecture and multicomponent design imparted anisotropic thermal transport pathways. STBC exhibited a thermal conductivity of 7.56 W m^−1^ K^−1^ in the layer-parallel direction and 0.17 W m^−1^ K^−1^ in the layer-perpendicular direction (Fig. 2a and Supplementary Fig. 4). With increasing temperature, thermal conductivity in both directions slightly rose, enhancing interfacial heat dissipation (Fig. 2b). Differential scanning calorimetry (DSC) revealed no glass transition between 293 and 413 K, indicating operational stability at elevated temperatures (Supplementary Fig. 5)^26,54^. The coefficient of thermal expansion was negligible within this range, further confirming thermal stability under high-temperature conditions (Supplementary Fig. 6). Notably, the thermal anisotropy ratio of STBC was 44.47, surpassing many reported anisotropic composites and underscoring its suitability for thermal interface management (Fig. 2c and Supplementary Table 1).

To elucidate the origin of thermal anisotropy, we conducted finite element simulations based on heat-transfer principles. Two models with distinct brick orientations were analyzed using DC3D8 heat-conduction elements, with an isothermal boundary of 573 K applied to the bottom surface. In the nacre-inspired hetero-configuration model (N-model) (Fig. 2d), bricks were oriented within 5° of the *y*-*z* plane. In the random homo-configuration model (R-model) (Fig. 2e), brick orientations were arbitrary. Axes denoted nanosheet orientation: *x* was out-of-plane; *y* and *z* were in-plane. The color scale and arrows jointly indicated heat-flux magnitude and direction, respectively. Heat-flux maps showed disordered vectors in the R-model, whereas the N-model exhibited aligned vectors that facilitated direct heat transfer. The N-model further displayed a pronounced directional temperature gradient, confirming that architectural heterogeneity governed efficient heat transport (Fig. 2f). The ordered brick arrangement constructed robust phonon transport channels within the *y*-*z* plane, enhancing conduction along the *y*-axis while suppressing it along the *x*-axis, thereby yielding strong directional differences in thermal conductivity. By contrast, the R-model exhibited quasi-isotropic conduction (Supplementary Fig. 7). Temperature-time traces revealed that anisotropy substantially widened the bounds of heating rates between the two directions (Fig. 2g). Overall, the biomimetic ordered design formed an efficient percolative network rather than disordered agglomerates, enabling directional and high-efficiency heat transport.

We further evaluated thermal performance under extreme conditions. STBC withstood continuous exposure to an 873 K flame without structural compromise (Fig. 2h and Supplementary Fig. 8a–c). The top surface remained white (Supplementary Fig. 8d), and although the bottom surface exhibited slight burn marks, no melting, deformation, cracking, or structural damage was observed, confirming safety and reliability for long-term use (Fig. 2i). The limiting oxygen index reached 69%, and post-combustion STBC retained more than 90% of its thermal conductivity, reflectance, and mechanical strength, with no significant performance degradation (Fig. 2j). Thus, STBC not only maintained structural integrity under extreme thermal exposure but also preserved its core thermal-management and mechanical protection functions. Cone calorimeter further indicated exceptionally low carbon emissions under fire conditions (Fig. 2k, Supplementary Figs. 9 and 10). Together, experiments and simulations demonstrated that STBC exhibited excellent anisotropic thermal conductivity and thermal stability in extreme environments, rendering it well suited for efficient passive cooling under complex thermal conditions.

### Passive radiative cooling

**Fig. 3:**
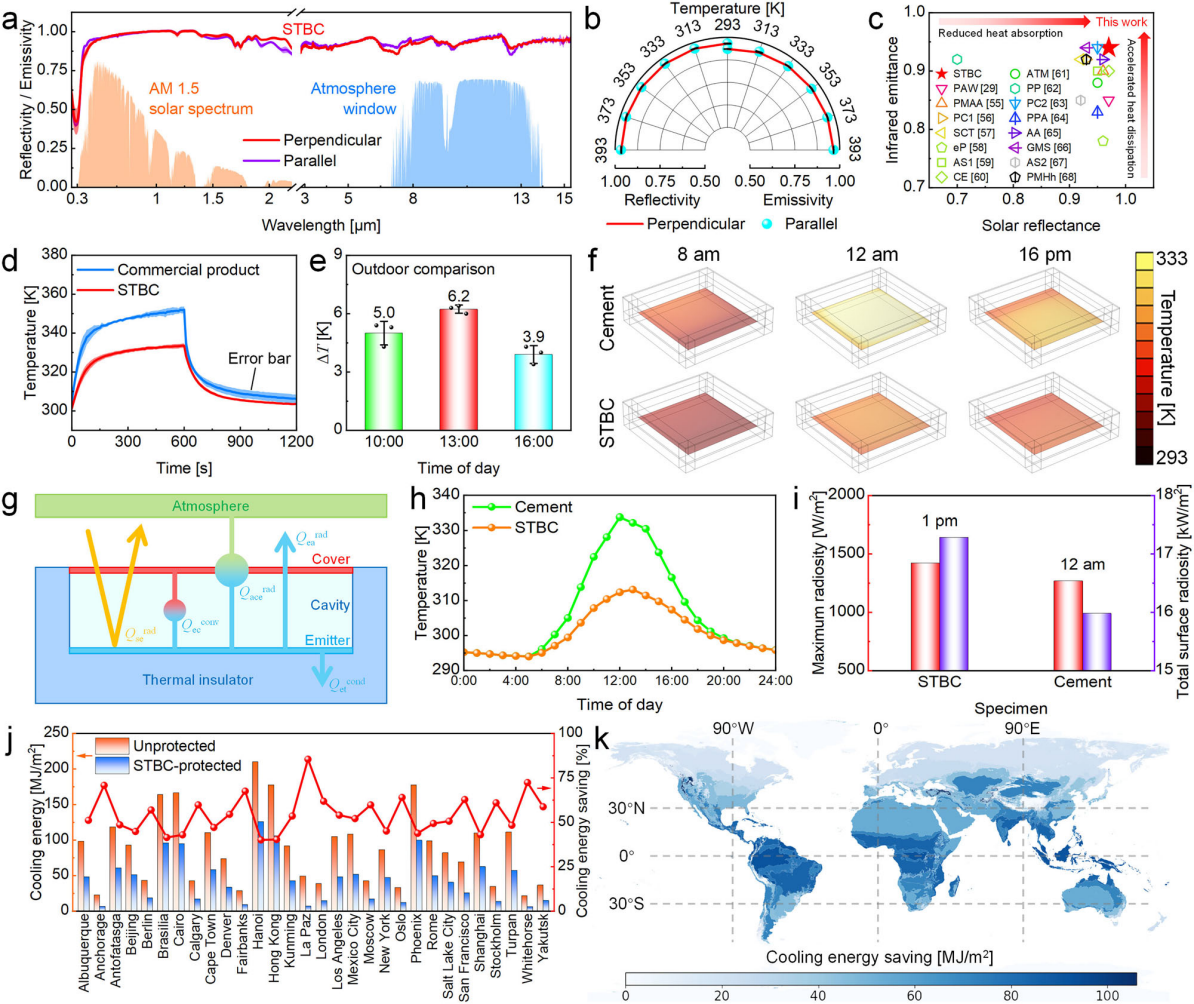
Radiative cooling and urban energy efficiency of STBC. **a** Reflectivity and emissivity of STBC measured in the parallel and perpendicular directions, and **b** their temperature-insensitive polar plots. **c** Solar reflectance and infrared emittance of STBC compared with reported radiative cooling composites^29,55–68^. Time-dependent temperature comparisons between a commercial radiative cooling product and STBC under **d** 1 sun exposure and **e** real-world outdoor sub-ambient conditions (Δ*T*: temperature change). **f** Finite-element temperature maps of conventional cement building material versus STBC. **g** Schematic of the radiative cooling energy balance. **h** Temperature, **i** maximum radiosity and total surface radiosity of cement and STBC over a simulated 1-day period in Hefei, China. **j** City-scale and **k** regional-scale energy-efficiency gains for STBC-protected building models worldwide. The error bars in (**a**, **b**, **d**, **e**) represent the standard deviations from three independent experiments. Source data are provided as a Source Data file.

Lowering the temperature of outdoor devices required both rejecting incoming solar radiation and releasing intrinsic heat^27,35^. We therefore investigated passive radiative cooling. STBC demonstrated high reflectivity across the solar spectrum and high emissivity in the mid-infrared in both parallel and perpendicular directions (Fig. 3a and Supplementary Fig. 11). Reflectivity and emissivity remained stable with increasing operating temperatures (Fig. 3b). At 393 K, the solar reflectance and infrared emittance both reached 0.97 in either orientation (Supplementary Discussion 1). This performance arose from the nacre-like hierarchical alignment of bricks, which maximized reflection in the UV-vis-NIR bands, while the mortar provided numerous dynamic crosslinking bonds with minimal vibrational absorption within the atmospheric transparency window (Supplementary Fig. 12a, b)^17,69,70^. The synergy yielded radiative cooling capacity surpassing many advanced materials (Fig. 3c, Supplementary Fig. 12c, d and Supplementary Table 2).

To assess practical cooling performance, we constructed an in-situ light-exposure temperature measurement platform (Supplementary Fig. 13). Solar irradiance was emulated using a xenon lamp that reproduced the spectral distribution and intensity of sunlight. A controller regulated irradiance, and an infrared camera tracked specimen temperatures in real time. Infrared imaging showed that a commercial radiative cooling product rapidly heated to 333 K within 1 min, whereas STBC warmed more gradually (Supplementary Fig. 14). Under standard 1 sun irradiation, the steady-state temperatures of STBC and the commercial product were 333.6 and 352.1 K, respectively (Fig. 3d). To capture real-world variability, we further evaluated sub-ambient radiative cooling outdoors under clear-sky conditions in Hefei, China, across forenoon, midday, and afternoon. STBC consistently maintained substantially lower temperatures than the commercial benchmark across changing solar incidence angles (Fig. 3e). Together, controlled and field tests indicated the superior cooling efficiency of STBC.

We complemented experiments with finite-element heat transfer simulations including surface-to-surface radiation. Using the diurnal solar-position schedule for Hefei, we computed temperature fields for conventional cement and STBC throughout the day (Fig. 3f, h). Surface temperatures of outdoor specimens scaled with solar irradiance, rising as irradiance increased, and the temperature difference between cement and STBC could reach up to 21.4 K. The cooling-plate balance (Fig. 3g) was expressed as:

1 $${Q}_{{{\rm{et}}}}^{{{\rm{cond}}}}+{Q}_{{{\rm{ec}}}}^{{{\rm{conv}}}}+{Q}_{{{\rm{ace}}}}^{{{\rm{rad}}}}+{Q}_{{{\rm{se}}}}^{{{\rm{rad}}}}+{Q}_{{{\rm{ea}}}}^{{{\rm{rad}}}}=0\,,$$

where *Q*_et_^cond^ was conductive heat exchange between the emitter and thermal insulator, *Q*_ec_^conv^ was convective heat exchange between the emitter and cover, *Q*_ace_^rad^ was net radiative exchange from the atmosphere and cover to the emitter, *Q*_se_^rad^ was solar radiative input to the emitter, and *Q*_ea_^rad^ was radiative emission from the emitter to the atmosphere. Enhancing radiative efficiency thus directly promoted heat dissipation. Supplementary Figs. 15 and 16 quantified surface radiosity of cement and STBC over 1 day. STBC achieved a maximum radiosity of 1420.20 W m^−2^ and a total daily radiosity of 17.28 kW m^−2^, exceeding cement by 152.20 W m^−2^ and 1.30 kW m^−2^, respectively (Fig. 3i). These results confirmed that STBC delivered superior cooling compared with both commercial radiative cooling materials and traditional building materials.

Because climate conditions varied globally, we conducted an energy-efficiency analysis across 30 cities using EnergyPlus (Supplementary Fig. 17 and Supplementary Discussion 2). Cooling-energy differences between unprotected and STBC-protected buildings reached 84.32 MJ m^−2^ in Hanoi, Vietnam, 77.88 MJ m^−2^ in Phoenix, USA, 71.66 MJ m^−2^ in Hong Kong, China, and 71.53 MJ m^−2^ in Cairo, Egypt (Fig. 3j and Supplementary Table 3). Importantly, STBC consistently delivered cooling-energy savings above 40%, with a maximum of 85.49% in La Paz, Bolivia. The global energy-saving map (Fig. 3k) showed higher radiative cooling efficiency near the equator, where STBC rejected more heat via passive radiation and thus yielded more pronounced energy savings. We also examined environmental robustness. TBC disintegrated within 90 s in water, whereas STBC maintained structural integrity after 48 h of full immersion with no visible disintegration or deformation (Supplementary Fig. 18a). After prolonged static immersion and cyclic wet-dry exposure, mass, dimensional, and mechanical retentions remained unchanged, confirming strong structural and performance stability under water exposure (Supplementary Fig. 18b). Furthermore, after dust deposition, simulated rainfall rapidly removed surface contaminants without residual fouling (Supplementary Fig. 18c). Combined with good hydrophilicity (Supplementary Fig. 18d), rainwater efficiently washed away particulates, preserving optical properties and cooling efficiency in complex outdoor environments. Overall, STBC exhibited outstanding passive cooling and self-cleaning, enabling uninterrupted around-the-clock operation and positioning it as a highly promising thermal-protection material for outdoor equipment.

### Kinetic impact defense

**Fig. 4:**
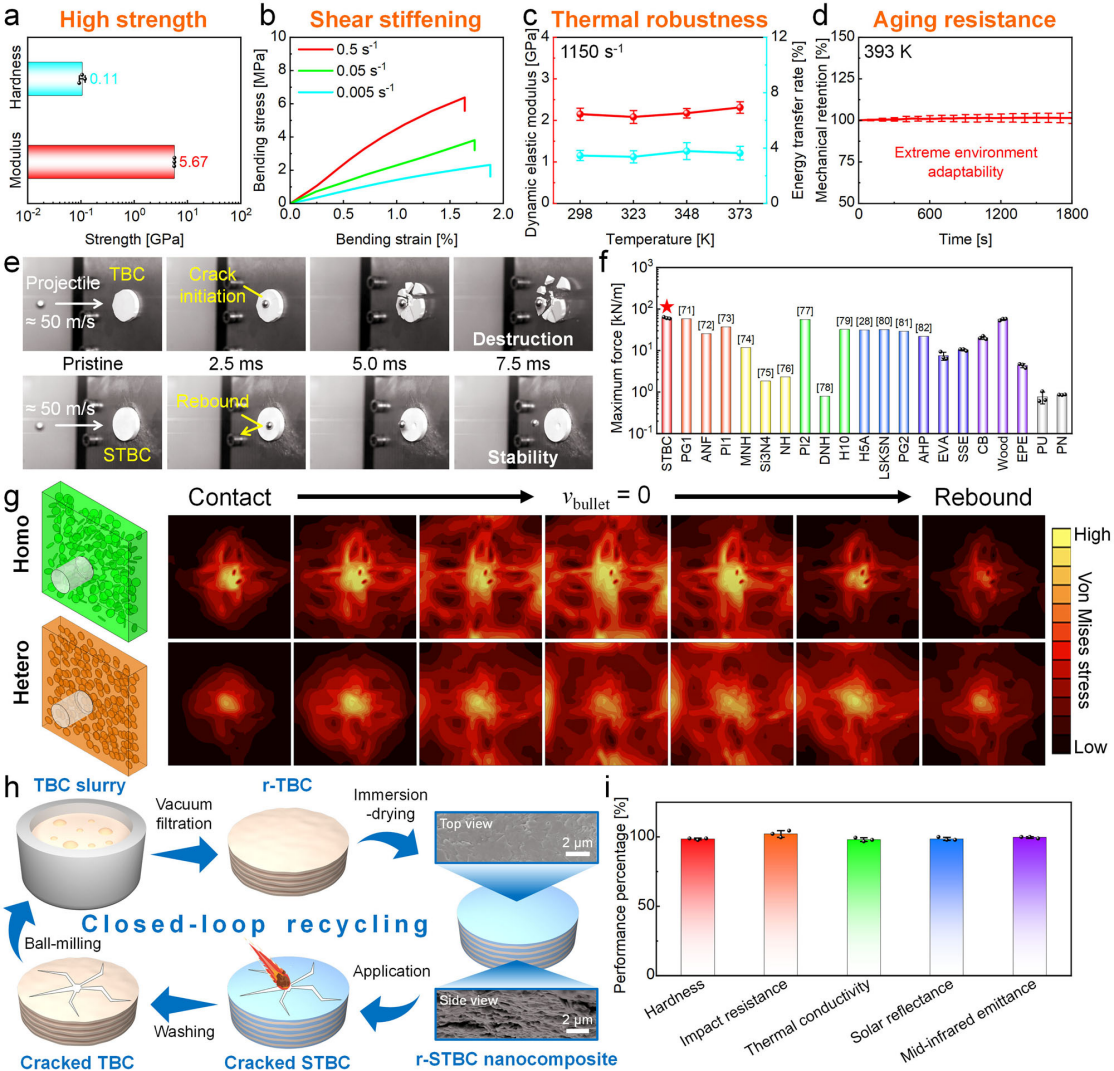
Dynamic impact resistance and closed-loop recycling of STBC. **a** High strength, **b** shear stiffening, **c** thermal robustness, and **d** aging resistance of STBC. **e** High-speed projectile impact processes of TBC and STBC. **f** Maximum force of STBC compared with various state-of-the-art^28,71–82^ and high-performance engineering materials in instrumented impact tests. **g** Homo- and hetero-configuration models with corresponding back-face von Mises stress distributions under high-velocity impact (*v*_bullet_: bullet velocity). **h** Schematic of the closed-loop recycling process for STBC. **i** Radar plot comparing the multi-functional performance of STBC and recyclable STBC (r-STBC). Each bar was normalized to the corresponding performance value of the pre-recycled STBC, which is defined as 100%. The images in (**h**) were created using 3ds Max 2022 and Microsoft PowerPoint 2016. The error bars in (**a**, **c**, **d**, **f**, **i**) represent the standard deviations from three independent experiments. Source data are provided as a Source Data file.

Historically, the design of passive cooling materials has emphasized optical performance to enable zero-energy cooling. In diverse and variable outdoor environments, however, such materials inevitably encountered kinetic impact threats. Impact resistance was therefore essential to ensure long-term stability, reliability, and practicality^81,83^. STBC exhibited a quasi-static hardness of 0.11 GPa and a modulus of 5.67 GPa, indicative of high strength (Fig. 4a and Supplementary Fig. 19). Three-point bending tests showed that bending stress increased with strain rates, evidencing rate-dependent stiffening (Fig. 4b). At a strain rate of 0.5 s^−1^, the flexural modulus and toughness were 420.96 MPa and 5.48 MJ m^−3^, respectively (Supplementary Fig. 20). High strain-rate performance was further evaluated using a split Hopkinson pressure bar (SHPB) system (Supplementary Discussion 3). At a strain rate of 1150 s^−1^, the dynamic elastic modulus remained essentially constant at ≈2.2 GPa from 298 to 373 K, demonstrating environmental robustness (Fig. 4c). The energy carried by a stress wave in the SHPB bars was computed using standard relations:

2 $${U}_{k}=\frac{{c}_{{{\rm{b}}}}{A}_{{{\rm{b}}}}}{2{E}_{{{\rm{b}}}}}{\int }_{0}^{{\!t}_{k}}{\sigma }_{k}^{2}(t){{\rm{d}}}t+\frac{{\rho }_{{{\rm{b}}}}{A}_{{{\rm{b}}}}{c}_{{{\rm{b}}}}^{3}}{2{E}_{{{\rm{b}}}}^{2}}{\int }_{0}^{{\!t}_{k}}{\sigma }_{k}^{2}(t){{\rm{d}}}t,k={{\rm{i}}},{{\rm{t}}}\,,$$

where *c*_b_ was the longitudinal wave velocity in the bar, *A*_b_ was the bar cross-sectional area, *E*_b_ was the bar modulus, *t*_*k*_ was the pulse duration, *σ*_*k*_(*t*) was the time-resolved stress and *ρ*_b_ was the bar density. The subscripts i and t denoted the incident and transmission bars, respectively. The energy transfer rate, representing the fraction of incident energy transmitted through specimens, was defined as:

3 $$\alpha=\frac{{U}_{{{\rm{t}}}}}{{U}_{{{\rm{i}}}}}.$$

At 373 K, the energy transfer rate for STBC was 3.64%, demonstrating that more than 95% of the high-speed impact energy was dissipated under extreme working conditions. During long-term thermal aging at 393 K, the storage modulus remained stable, indicating negligible thermal degradation (Fig. 4d). In composite cyclic aging tests simulating outdoor light, humidity, and temperature fluctuations, cycles of 365 nm UV irradiation (0–80 W m^−2^), 40–85% relative humidity, and 303–333 K temperature were applied (Supplementary Fig. 21a–c), and post-test storage modulus deviations were within 2% of initial values (Supplementary Fig. 21d).

To probe high-velocity penetration resistance, a ballistic impact platform was employed^17,71^. A spherical projectile (50 m s^−1^) was launched toward the specimen, and the event was captured by high-speed imaging. Brittle TBC exhibited crack initiation and catastrophic failure. In contrast, STBC rapidly deflected the projectile and preserved structural integrity via abrupt shear stiffening (Fig. 4e and Supplementary Fig. 22a). This resilience arose from the impact-hardening capability of SSG and synergistic interfacial interactions (Supplementary Fig. 22b, c). Upon impact, STBC formed load-bearing bridges within the interpenetrating 1D/2D network, suppressing crack initiation, while abundant interfacial hydrogen bonding and dynamic B-O crosslinking dissipated a substantial fraction of kinetic energy^33,84^. Following ISO 6603-2:2023, puncture behavior was quantified (Supplementary Fig. 23) using an instrumented drop-tower method:

4 $${E}_{j}={\int }_{\!\!\!\!\!0}^{{\!l}_{j}}F(l){{\rm{d}}}l,j={{\rm{m}}},{{\rm{p}}}\,,$$

where *F*(*l*) was the impact force as a function of displacement *l*, and *l*_*j*_ was the impact displacement. The subscripts m and p referred to maximum value and puncture points, respectively. At an impact height of 50 cm, STBC achieved an absorbed energy of 107.64 J m^−1^ and a maximum force of 61.10 kN m^−1^, outperforming state-of-the-art and high-performance engineering materials (Fig. 4f and Supplementary Table 4). To elucidate intrinsic protection mechanisms and assess stress-yield risks, explicit dynamic finite-element models of homogeneous and heterogeneous architectures were constructed (Fig. 4g). Von Mises stress maps indicated that heterogeneous architectures distributed and dissipated stress more uniformly, mitigating stress concentrations and enabling greater load tolerance^85,86^. Compared with the homogeneous model, the heterogeneous model exhibited a longer buffering duration (Supplementary Fig. 24). Parametric analyses indicated that greater platelet alignment, lower porosity, and smaller layer aspect ratios enhanced thermal transport, emissivity, and mechanical strength (Supplementary Fig. 25). These results highlighted the synergistic contributions of architecture and component to the superior impact protection of STBC under diverse dynamic stimuli.

In outdoor service, inevitable cracks and damage underscored the importance of recyclability. By selectively removing SSG and dissolving TBC, STBC could be reprocessed into recyclable STBC (r-STBC), enabling closed-loop recycling (Fig. 4h). Both r-STBC and STBC dissipated more than 80% of impact force in drop-tower tests (Supplementary Fig. 26). Importantly, the relative differences between r-STBC and STBC in hardness, impact resistance, thermal conductivity, solar reflectance and mid-infrared emittance were all within 5% (Fig. 4i and Supplementary Table 5). This closed-loop strategy demonstrated strong sustainability and environmental compatibility by diverting waste and reducing raw-material demand.

### Thermally and mechanically resistant sandwich paradigm

**Fig. 5:**
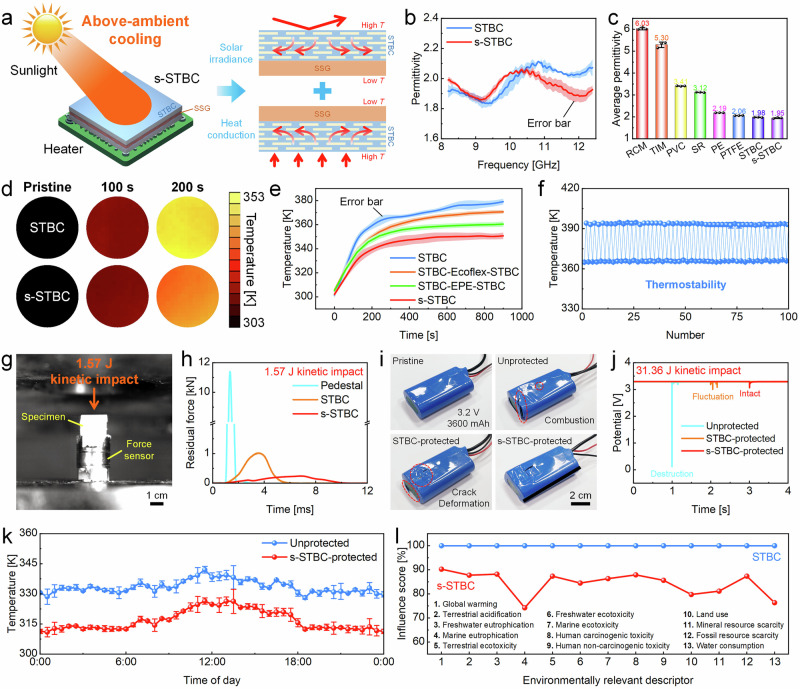
Collaborative passive cooling and enhanced impact protection of sandwich-structural STBC (s-STBC). **a** Above-ambient passive cooling mechanism of s-STBC (*T*: temperature). **b** Frequency-dependent permittivity of STBC and s-STBC. **c** Average permittivity of representative dielectric materials. **d** Infrared images, **e** temporal temperature profiles, and **f** thermal stability of heater-integrated passive insulators under 1 sun irradiation. **g** Drop-tower system and **h** residual forces recorded for pedestal, STBC and s-STBC under a 3.2 kg impact. **i** Damage states and **j** potential-time curves of unprotected, STBC-protected, and s-STBC-protected batteries upon 31.36 J impact energy. **k** Temperature evolution of unprotected and s-STBC-protected heaters during outdoor passive cooling in Hefei, China. **l** Environmental influence scores across relevant categories for STBC and s-STBC. The error bars in (**b**, **c**, **e**, **f**, **k**) represent the standard deviations from three independent experiments. Source data are provided as a Source Data file.

With rising power densities and harsh operation environments, heat dissipation and impact buffering often competed for design priority^28,87,88^, yet both were essential for ensuring device stability in extreme conditions. We therefore developed an intelligent thermo-mechanically resilient sandwich STBC (s-STBC) configuration, comprising STBC-SSG-STBC laminates (Fig. 5a). Its above-ambient passive cooling combined high solar reflectance in the top layer with rapid heat conduction in the bottom layer. When subjected to external (sunlight) and internal (device) heat sources, the nacre-mimetic hierarchy established efficient in-plane heat-conduction channels. Rapid lateral diffusion increased the effective dissipation area, thereby facilitating heat removal. The central SSG layer, which was thermally insulating and high heat storage, decoupled the two STBC sections, increased the temperature gradient across the stack, and promoted outward heat rejection by the outer STBC surfaces. Balancing maximum temperature, residual force transmission, overall thickness, and creep constraints, a 5-mm-thick SSG interlayer was selected for thermal insulation and impact resistance^17,89^ (Supplementary Fig. 27). STBC also exhibited practical adhesion across diverse substrates, enhancing thermal-interface management (Supplementary Fig. 28). Compared with an aluminum interface, the STBC-based interface reduced interfacial thermal resistance by 50.86%, substantially improving interfacial heat-transfer efficiency (Supplementary Fig. 29). Moreover, most devices required thermally conductive interfaces that were good electrical insulators to prevent short circuits and avoid interference with signal transmission^3,26^. The volume resistivities of STBC and SSG were 7.62 × 10^9^ and 6.23 × 10^11^ Ω, respectively, satisfying electrical insulation requirements (Supplementary Fig. 30). s-STBC exhibited a low dielectric constant and high breakdown resistance, outperforming many commercial materials (Fig. 5b, c and Supplementary Table 6). It enhanced electromagnetic transparency relative to STBC and minimized interference with wireless communication, allowing signals to traverse the material with negligible attenuation (Supplementary Fig. 31). s-STBC was suitable for scenarios requiring mechanical protection without hindering device connectivity.

To further interrogate passive cooling in the sandwich architecture, we constructed a photothermal coupling platform. Equal-volume specimens were mounted on resistive heaters and irradiated with a xenon lamp. Real-time temperatures were recorded via infrared imaging. At 100 s, STBC and s-STBC showed similar temperatures, but at 200 s, a clear divergence emerged (Fig. 5d). The steady-state temperature difference reached ≈30 K (Fig. 5e). In bulk STBC, radiative cooling and conductive coupling to the heater partially counteracted each other, limiting net sub-ambient performance. In s-STBC, the highly insulating heat-capacitive SSG layer attenuated heat transfer, enabling both STBC faces to deliver enhanced radiative cooling. The architecture thus achieved passive thermal insulation coupled with synergistic cooling (Supplementary Discussion 4). After 100 load-unload cycles of the heat source, s-STBC maintained temperature stability, evidencing robust thermal durability and long service life under variable extreme environments (Fig. 5f).

We next evaluated dynamic mechanical impact protection. In a drop-tower experiment with a 3.2 kg blunt hammerhead, the specimen experienced a contact-rebound-secondary contact sequence within 150 ms (Fig. 5g and Supplementary Fig. 32). A force sensor recorded the transmitted residual force at first contact. Without protection, the residual impact force was 11.40 kN. With STBC and s-STBC, only 8.95% and 2.11% of the input load were transmitted, respectively (Fig. 5h). Given the criticality of battery safety, we performed a 31.36 J dynamic impact test: The unprotected battery short-circuited and ignited; the STBC-protected battery exhibited cracking and deformation; the s-STBC-protected battery showed no significant damage (Fig. 5i and Supplementary Fig. 33a). Potential-time traces revealed destructive, fluctuating, and intact responses for unprotected, STBC-protected, and s-STBC-protected batteries, respectively, highlighting the superior buffering capacity of s-STBC (Fig. 5j and Supplementary Fig. 33b, c).

To assess practical performance and sustainability, we conducted outdoor high-temperature experiments. s-STBC was attached to a heated device (353 K) under direct sunlight, and two thermohygrometers monitored unprotected and STBC-protected surfaces over 1 day in Hefei, China. The maximum solar irradiance reached 1.06 kW m^−2^, with average humidity ≈40% (Supplementary Fig. 34). Surface temperatures without s-STBC were consistently higher than with s-STBC, with differences exceeding 11.9 K and peaking at 20.5 K at night (Fig. 5k). During the day, s-STBC resisted solar loading and dissipated heat through convection and radiation. At night, as the device-ambient temperature differential increased, s-STBC continued dissipating heat via passive radiative cooling.

Beyond operational energy savings, carbon-emission reductions should be evaluated over the full life cycle^30,90,91^. Conventional cooling materials typically reduced emissions only during service, whereas extraction, production, and end-of-life disposal phases were often under-accounted^92,93^. Using 1 kg of material as the functional unit, we quantified a range of environmental influence categories (Supplementary Fig. 35, Supplementary Discussion 5, Supplementary Tables 7 and 8)^94,95^. s-STBC reduced influences across all categories compared with STBC, confirming its low-carbon and environmentally friendly profile (Fig. 5l, Supplementary Tables 9 and 10). Industrial deployment via continuous processing, centralized raw-material procurement, and minimized process losses was expected to further reduce energy demand and unit cost.

In summary, intelligent s-STBC delivered combined passive cooling, impact resistance and environmental sustainability, making it a promising multi-functional safeguard for outdoor devices. The brick-and-mortar architecture afforded substantial design latitude and provided a compelling pathway for further performance enhancement. Future iterations might enable autonomous tuning of anisotropic thermal conductivity through mechanically reconfigurable designs that permitted controlled deformation, allowing reversible switching between thermally conductive and insulating states. Moreover, judicious selection of brick-and-mortar constituents, coupled with targeted channel architectures, could precisely tailor properties and realize multifunctionality. By synergistically controlling the composition and microstructure of the brick and mortar, comprehensive improvements in mechanical performance and functional adaptability could be achieved. These considerations motivated continued exploration of biomimetic brick-and-mortar composites and would facilitate their deployment in practical settings.

## Discussion

As a result, we proposed a nacre-mimetic design strategy that integrated passive cooling with impact mitigation. The as-developed SSG-TA@BNNS-CNC (STBC) nanocomposite exhibited a thermal anisotropy ratio of 44.47 and remained intact after 1 h of direct flame exposure, evidencing exceptional environmental robustness. At 393 K, STBC achieves a solar reflectance of 0.97 and a mid-infrared emittance of 0.97, enabling efficient passive cooling even at elevated operating temperatures. In urban-building simulations, it delivered cooling energy savings exceeding 40%, with peak reductions up to 84.32 MJ m^−2^. Mechanically, STBC showed a modulus of 5.67 GPa and maintained a stable energy transfer rate of approximately 3.78% under 1150 s^−1^ strain-rate loading across temperatures, indicating that more than 95% of incident kinetic energy was dissipated. It withstood 50 m s^−1^ projectile impacts and, if damaged, could be fully recovered through a closed-loop recycling process. Collectively, STBC surpassed many advanced high-performance materials in both passive cooling and impact protection. Building on this platform, we developed a thermally and mechanically resilient sandwich STBC (s-STBC) architecture comprising STBC-SSG-STBC laminates. s-STBC provided stronger cooling and buffering, lowering temperature by about 30 K in photothermal tests and transmitting only 2.11% of the incident load in drop-tower experiments, a 76.42% reduction relative to a single STBC. Outdoor trials and life cycle assessment confirmed its practicality and sustainability, establishing s-STBC as a compelling candidate for thermal management and protection of outdoor devices operating under harsh environmental conditions.

## Methods

### Materials

BN was purchased from Minnesota Mining and Manufacturing (3M) Company, Minnesota, USA. TA was obtained from Macklin Biochemical Co., Ltd., Shanghai, China. CNC was acquired from ScienceK Co., Ltd., Zhejiang, China. BA and OA were supplied by Sinopharm Chemical Reagent Co., Ltd., Shanghai, China. HSO was sourced from Fenghuang Chemical Technology Co., Ltd., Guangdong, China. Ethanol was procured from Titan Scientific Co., Ltd., Shanghai, China. Commercial radiative cooling material (RCM) and thermal interface material (TIM) were purchased from Coinno Intelligent Technology Co. Ltd., Hainan, China and Yumeiren thermal Conductive Material Co. Ltd., Guangdong, China, respectively.

### Preparation of TA@BN nanosheet (BNNS)-CNC (TBC) aqueous slurries

5 g BN and 0.125 g TA were dispersed in deionized water, and ball-milled at 353 K for 24 h to exfoliate BN into TA-functionalized BNNS (TA@BNNS). TA adhered to BNNS through π-π interactions, enhancing BNNS hydrophilicity and dispersibility and promoting the formation of an ordered architecture. 0.25 g CNC was then added to the TA@BNNS dispersion, and ball milling was continued for an additional 24 h to yield the TA@BNNS-CNC (TBC) aqueous slurry.

### Fabrication of hierarchical TBC monoliths

The TBC slurry was subjected to vacuum filtration at 0.04 MPa for 12 h. Under gravity and vacuum-assisted flow, the 2D TA@BNNS stacked and self-assembled into a hierarchical structure that interpenetrated with the 1D CNC network. The resulting bulk was annealed in an oven at 353 K for 12 h to relieve residual stress and remove moisture, thereby improving mechanical strength. The TBC monolith was obtained after natural cooling.

### Synthesis of shear-stiffening gels (SSGs)

1 g BA was uniformly dispersed in 30 g HSO and stirred at 453 K for 2 h to promote the formation of dynamic boron-oxygen (B-O) crosslinks. 75 μL OA was subsequently added and the mixture stirred for an additional 1 h to further catalyze the reaction. The hybrid was then placed in a drying oven at 298 K for 6 h to obtain SSG exhibiting rate-dependent stiffening behavior.

### Preparation of nacre-mimetic SSG-TA@BNNS-CNC (STBC) nanocomposites

30 g SSG was dissolved in ethanol by sonication for 3 h. The TBC monolith was impregnated with the SSG-ethanol solution under a 0.04 MPa vacuum. The SSG-treated monolith was dried at 353 K to stabilize hydrogen bonding and dynamic crosslinks. After natural cooling, a nacre-mimetic brick-and-mortar STBC nanocomposite was obtained, in which TBC served as the brick and SSG as the mortar.

### Closed-loop of recyclable STBC (r-STBC) composites

A cracked STBC specimen was washed with ethanol to remove SSG, yielding a cracked TBC skeleton. The TBC was ball-milled in deionized water at 353 K for 48 h to produce a TBC slurry. A recyclable TBC (r-TBC) monolith was then prepared through vacuum filtration. Using the same impregnation, drying, and curing protocol as for the original SSG integration, an r-STBC composite was successfully fabricated.

### Design of sandwich-structural STBC (s-STBC) composites

Two STBC monoliths and one SSG layer were cut to identical dimensions. Leveraging the intrinsic adhesive properties of SSG, the three components were laminated into a tri-layer s-STBC composite, with SSG as the core and STBC as the top and bottom layers.

### Characterization

The microstructures of the materials were examined by scanning electron microscopes (SEM, GenimiSEM 500, Zeiss, UK, and DB500, Ciqtek, China). Anisotropic thermal conductivities of STBC specimens (30 × 30 × 10 mm^3^) at various temperatures were measured using a thermal constant analyzer (TPS 2500S, Hot Disk, Sweden) and a laser thermal conductivity meter (LFA 467, Netzsch, Germany). Thermal resistance (25 × 25 × 1 mm^3^) was assessed with an interface material thermal resistance and heat conduction coefficient instrument (LW-9389MD, Longwin, China) at 137.90 kPa (20 psi). Coefficients of thermal expansion (25 × 8 × 8 mm^3^) and heat flow were determined using a thermal dilatometer (DIL402, Netzsch, Germany) and a differential scanning calorimeter (DSC 204F1, Netzsch, Germany), respectively, over 293–413 K. Heat release and carbon emissions under fire conditions were measured using a cone calorimeter (6810, Vouch, China) over a burn duration of 1800 s. Hydrophobicity and electrical insulation were characterized with a water droplet angle tester (CHD-JCJ180A-1, Chengding Technology, China) and an electrometer (6517B, Keithley, USA). Dielectric properties were measured with a vector network analyzer (3671E, Ceyear Technologies, China). The BNNS mass fraction was fixed at 62 wt% for all test specimens.

The hardness and modulus of STBC (20 × 20 × 5 mm^3^) were measured by a nanoindentation tester (NHT2, Anton Paar, Austria). The shear-stiffening response of SSG (0.1–100 Hz), as well as the aging resistance and mechanical strength retention of STBC at 1 Hz and 393 K (diameter: 20 mm, thickness: 2 mm), were evaluated using a rotational rheometer (Physica MCR 302, Anton Paar, Austria) with an oscillatory shear strain of 0.1%. Three-point bending tests on STBC (50 × 50 × 5 mm^3^) were performed at strain rates of 0.5, 0.05, and 0.005 s^−1^, and storage moduli (diameter: 20 mm, thickness: 2 mm) were measured over 25–50 Hz using a dynamic mechanical analyzer (DMA, ElectroForce 3200, TA instruments, USA). Single-lap shear tests (adhesion area: 20 × 20 mm^2^) were conducted on a universal testing machine (Criterion model 43, MTS Systems, USA). High-speed compression tests (diameter: 14 mm, thickness: 1 mm) were carried out using a SHPB apparatus comprising a striker, incident, transmission, and absorber bars. Striker velocity was measured by a photogate, and stresses in the incident and transmission bars were recorded using four strain gauges (BX120-3AA, Xianmisi, China). Stress-wave signals were captured with a high dynamic strain indicator (CS-1D, Xinheng Electronic Technology, China) and an oscilloscope (DPO2014B, Tektronix, USA). Impact resistance of commercial materials (diameter: 40 mm), as well as three-layer STBC and s-STBC (single-layer diameter: 40 mm, thickness: 5 mm), was characterized on a drop-tower impact tester (ZCJ1302-A, MTS Systems, USA) using an 800 g impactor with a 5 mm diameter rod for commercial materials, and a 3.2 kg ball-peen impactor for the STBC configurations. In ballistic experiments on STBC (diameter: 40 mm, thickness: 5 mm), projectiles were propelled by nitrogen gas, velocity was measured via an oscilloscope, and the impact process was recorded with a high-speed camera (Phantom v2512, Vision Research, USA) at 20,000 fps.

Temperature-dependent reflectance and emittance (50 × 50 × 5 mm^3^) were characterized using a UV-vis-NIR spectrophotometer (0.2–2.5 μm, UV 3600, Shimadzu, Japan) and a Fourier-transform infrared spectrometer (2.5–15.3 μm, Nicolet iS50, Thermo Fisher Scientific, USA). Specimen temperatures were monitored with an infrared camera (865, Testo, Germany). A xenon lamp (BXU116, Xingchuang Electronics, China) simulated standard sunlight, and solar intensity was modulated with a solar power meter (1333, TES Electrical Electronic, China). A UV lamp (365 nm, Haozhuo lighting, China) and a humidifier (DEM-F570, Deerma, China) provided 365 nm irradiation and controlled humidity, respectively. Heaters requiring cooling (JCDZ, Epistar, China) and lithium iron phosphate batteries (3.2 V, 3600 mAh, Zhongli Energy technology, China) were positioned beneath STBC and s-STBC for radiative cooling and impact resistance testing. Light intensity was recorded by a solar total radiation detector (YGY-TBQ, Chengyun Technology, China), and temperature and humidity were monitored with a hygrothermograph (DYF60A, Dayufeng Technology, China). Quantitative data were presented as the mean ± SD from *n* = 3 independent samples.

### Reporting summary

Further information on research design is available in the Nature Portfolio Reporting Summary linked to this article.

## Supplementary information

Supplementary Information

Reporting Summary

Transparent Peer Review file

## Source data

Source Data

## Data Availability

All relevant data generated from this study are available from the article and Supplementary Information. Source data are provided with this paper.
