# Supplementary material for: Collaborative passive cooling of impact-hardening interfaces enabled by nacre-mimetic design
Source: Nat Commun. 2026 Jun 22;17:7823. doi: 10.1038/s41467-026-74755-w (PMC13439421; doi:10.1038/s41467-026-74755-w)
Supplement: Supplementary file 1 [file 41467_2026_74755_MOESM1_ESM.pdf]

Supplementary Information

Collaborative passive cooling of impact-hardening interfaces enabled by nacre-mimetic design

Zimu Li[1], Sheng Wang[1]*, Shuai Liu[1], Jianpeng Wu[1], Wenhui Wang[1], Zhentao Zhang[1], Shilong Duan[1], Liangyuan Qi[2], Yuan Hu[2] & Xinglong Gong[1,2]*

[1]*CAS Key Laboratory of Mechanical Behavior and Design of Materials, Department of Modern Mechanics, University of Science and Technology of China, Hefei, Anhui, 230027, PR China*

[2]*State Key Laboratory of Fire Science, University of Science and Technology of China, Hefei, Anhui, 230026, PR China*

**Corresponding authors: wsh160@ustc.edu.cn (S. Wang), gongxl@ustc.edu.cn (X. L. Gong)*

Contents

Supplementary Discussions 1 to 5

Supplementary Figures 1 to 35

Supplementary Tables 1 to 10

Supplementary References

Supplementary Discussion 1 | Solar reflectance and infrared emittance calculation.

Solar reflectance was defined as:

$$\overline{R}_{\mathrm{sol}}=\frac{\int_{0.3}^{2.5} I_{\mathrm{sol}}(\lambda)\cdot R_{\mathrm{sol}}(\lambda,T)\,\mathrm{d}\lambda}{\int_{0.3}^{2.5} I_{\mathrm{sol}}(\lambda)\,\mathrm{d}\lambda}, \tag{1}$$

where $I_{\mathrm{sol}}(\lambda)$ was the ASTM G173 global solar spectrum at wavelength λ, and $R_{\mathrm{sol}}(\lambda, T)$ was the spectral reflectance at wavelength λ and temperature T. Solar reflectance over 0.3-2.5 μm was measured using a Shimadzu UV-3600 UV-vis-NIR spectrophotometer. Prior to testing, the instrument was calibrated with a high-reflectance barium sulfate reference to minimize errors arising from ambient light and baseline drift.

The 8-13 μm atmospheric transparency window lied in the mid-infrared region where atmospheric absorption was minimal and transmittance was high, permitting thermal radiation to pass to outer space with negligible attenuation. Passive radiative cooling exploited materials that emitted strongly within this window to effectively reject heat to the sky while minimizing reabsorption and back-reflection by the atmosphere. Infrared emittance was defined as:

$$\overline{E}_{\mathrm{inf}}=\frac{\int_{8.0}^{13.0} I_{\mathrm{bla}}(\lambda,T)\cdot E_{\mathrm{inf}}(\lambda,T)\,\mathrm{d}\lambda}{\int_{8.0}^{13.0} I_{\mathrm{bla}}(\lambda,T)\,\mathrm{d}\lambda}, \tag{2}$$

where $I_{\mathrm{bla}}(\lambda, T)$ and $E_{\mathrm{inf}}(\lambda, T)$ were the blackbody emittance spectrum and the spectral hemispherical emittance at wavelength λ and temperature T, respectively, following Planck's blackbody radiation law:

$$I_{\mathrm{bla}}(\lambda,T)=\frac{2hc^2}{\lambda^5}\cdot\frac{1}{e^{\frac{hc}{\lambda kT}}-1}, \tag{3}$$

where h was Planck's constant, c was the speed of light, e was the natural constant, and k was the Stefan-Boltzmann constant. Mid-infrared emissivity in the 8-13 μm window was measured using a Thermo Fisher Nicolet iS50 Fourier-transform infrared

spectrometer, calibrated against a high-reflectance gold mirror to ensure accurate emissivity determination.

For variable-temperature measurements, both instruments were equipped with temperature-controlled chambers. The measurement system was preheated and stabilized for 30 min before data acquisition. Spectral scanning commenced only after the specimen reached thermal equilibrium, with the temperature deviation strictly maintained within ±1 K to avoid artifacts due to thermal fluctuations.

Supplementary Discussion 2 | Energy efficiency simulation for various global cities.

To quantify the cooling energy savings attributable to STBC, building energy simulations were performed using EnergyPlus 23.2.0. The building model was constructed in SketchUp (Supplementary Fig. 17). First, cooling energy consumption was calculated for the model without STBC as the outermost building material. STBC was then applied as the exterior layer under identical boundary conditions, and the cooling energy consumption was recalculated. Comparative analyses for buildings with and without STBC were conducted for multiple global cities using climate data downloaded from the EnergyPlus website (https://energyplus.net/weather).

Supplementary Discussion 3 | Split Hopkinson pressure bar (SHPB) system calculation.

SHPB analyses assumed 1D wave propagation and stress equilibrium, described as:

$$\sigma_{\mathrm{i}} = \sigma_{\mathrm{t}} + \sigma_{\mathrm{r}}, \tag{4}$$

where σ_i, σ_t and σ_r were the incident, transmitted, and reflected stress waves, respectively. Based on 1D stress-wave theory, the specimen's dynamic stress, strain, and strain rate could be expressed as followed:

$$\sigma_{\mathrm{s}} = \frac{E_{\mathrm{b}} A_{\mathrm{b}}}{A_{\mathrm{s}}} \varepsilon_{\mathrm{t}}, \tag{5}$$

$$\varepsilon_{\mathrm{s}} = \frac{2c_{\mathrm{b}}}{l_{\mathrm{s}}}\int_0^t \left(\varepsilon_{\mathrm{i}} - \varepsilon_{\mathrm{t}}\right)\mathrm{d}t\,, \tag{6}$$

$$\dot{\varepsilon}_{\mathrm{s}} = \frac{2c_{\mathrm{b}}}{l_{\mathrm{s}}}\left(\varepsilon_{\mathrm{i}} - \varepsilon_{\mathrm{t}}\right), \tag{7}$$

where E_b was the bar modulus, A_b and A_s were the cross-sectional areas of the bar and specimen, respectively, ε_i and ε_t were the strains measured on the incident and transmitted bars, respectively, and c_b was the bar wave velocity, t was the pulse duration, and l_s was the specimen thickness. These relations were used to compute the mechanical response of the specimens from the recorded strain signals.

Supplementary Discussion 4 | Passive insulator control experiment.

Commercial Ecoflex and EPE foam were selected as passive interlayers with matched thickness to replace SSG, yielding STBC-Ecoflex-STBC and STBC-EPE-STBC specimens, respectively. Under identical irradiance, the steady-state temperatures of STBC, STBC-Ecoflex-STBC, STBC-EPE-STBC, and s-STBC were 379.1, 370.7, 360.4, and 350.7 K, respectively (Fig. 5e).

For passive thermal insulation, interfacial thermal resistance between dissimilar materials slowed heat transfer compared with monolithic STBC, confirming that passive insulators could lower STBC temperature by limiting heat input. For active synergistic cooling, both the specific heat and emissivity of SSG exceeded those of Ecoflex. Materials with higher specific heat required greater heat input to achieve the same temperature rise, thereby delaying the system's thermal response. Moreover, higher emissivity enhanced radiative heat loss and established an effective radiative heat sink. The s-STBC architecture simultaneously suppressed heat conduction and promoted thermal dissipation to the surroundings, producing a synergistic inhibition of heat flow. In addition, the thermal insulation performance of SSG as an interlayer surpassed that of commercial foam, further validating the advantages of the s-STBC

design. The superiority of s-STBC exceeded that attainable with a simple "insulation layer & STBC" configuration, demonstrating a genuine synergy.

Supplementary Discussion 5 | Life cycle assessment (LCA) simulation.

LCA evaluated the environmental influences and potential influences of all inputs and outputs associated with a product system throughout its life cycle, in accordance with ISO 14040 and ISO 14044. In this work, SimaPro 10.1 was used for modeling. Using 1 kg of material as the functional unit, the life cycle environmental influences of STBC and s-STBC were compared. The system boundary and life cycle inventory data for STBC and s-STBC were provided in Supplementary Fig. 35, Supplementary Tables 7 and 8.

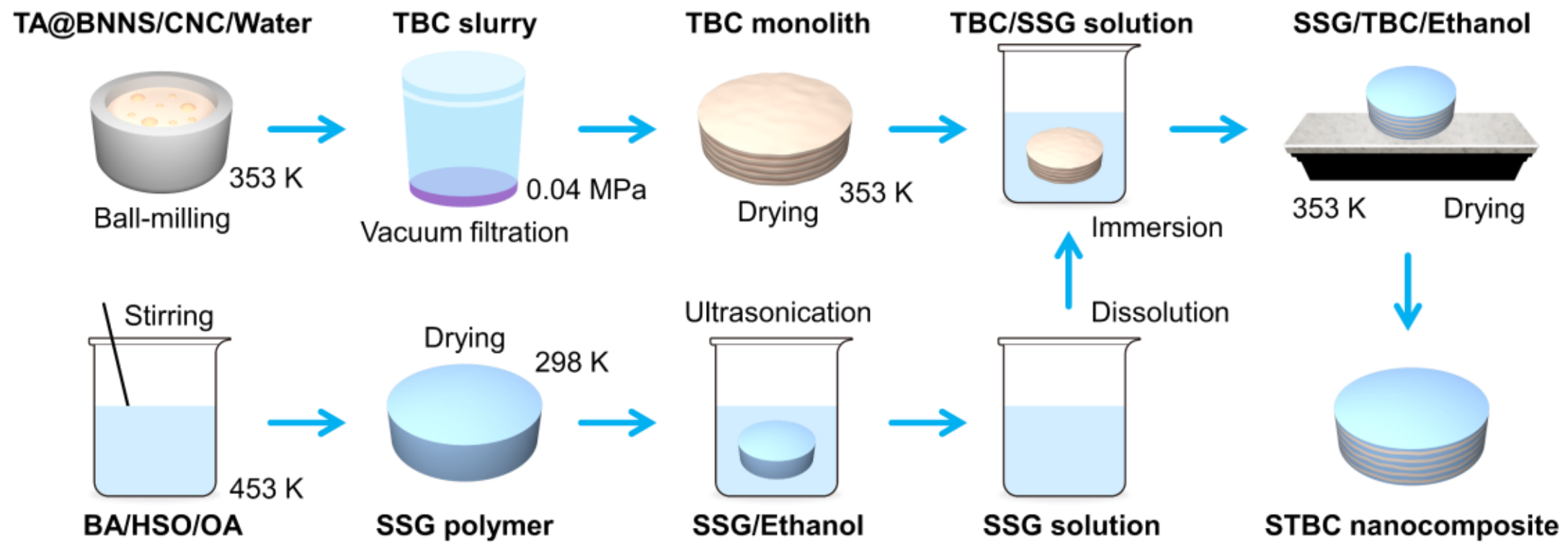

Supplementary Fig. 1 | Detailed flowchart of the STBC preparation procedure. Dissolution: SSG was uniformly dissolved in ethanol via ultrasonication to form a stable SSG-ethanol solution. Immersion: The TBC framework was immersed in this solution and fully permeated under vacuum, ensuring intimate bonding between SSG and TBC. Drying: The impregnated composite block was heated in an oven at 353 K to remove ethanol, yielding STBC nanocomposite with a brick-and-mortar architecture. The images were created using 3ds Max 2022 and Microsoft PowerPoint 2016.

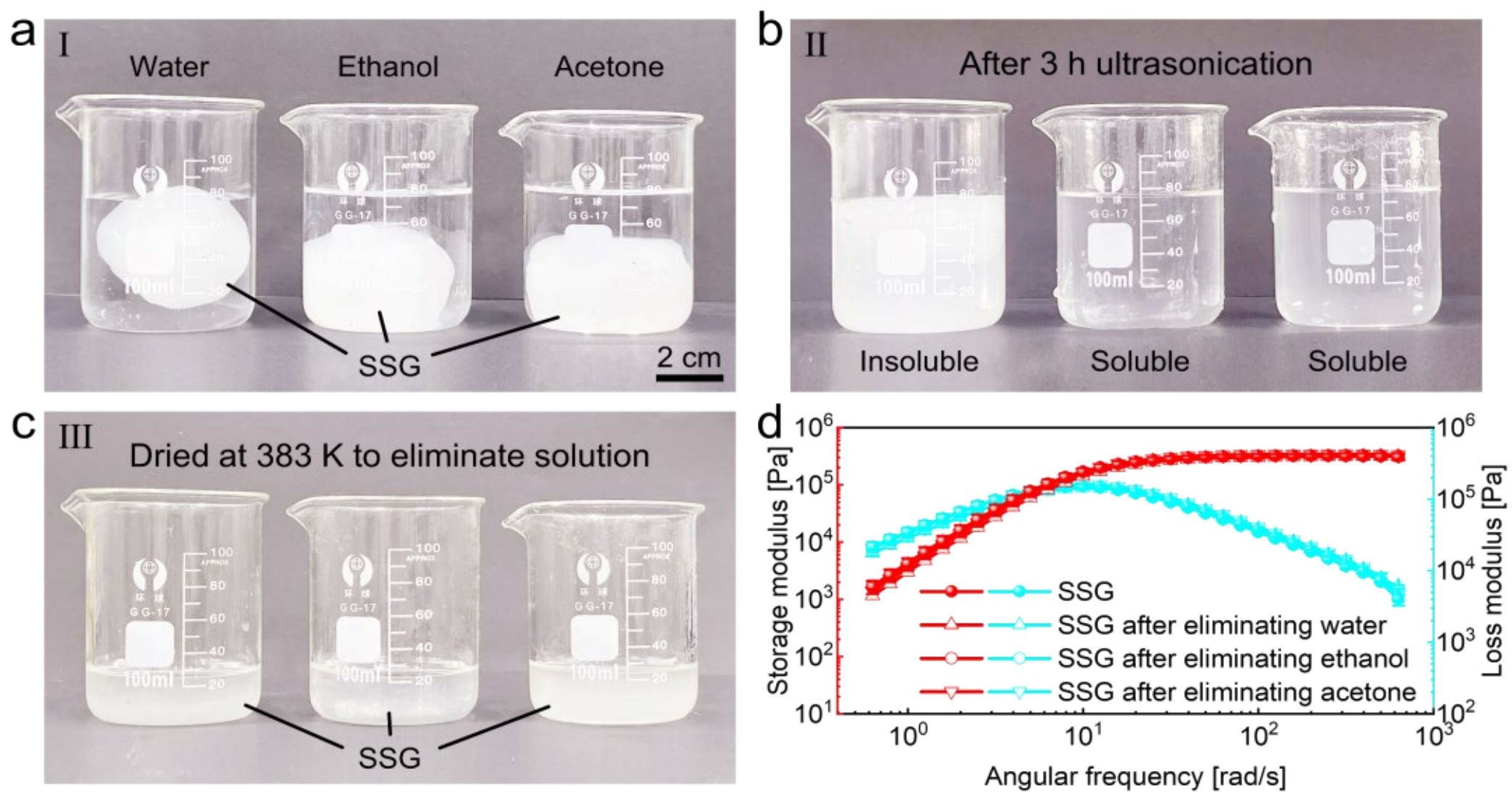

Supplementary Fig. 2 | Stability of SSG in humid and polar environments. a Pristine state. **b** After 3 h of ultrasonication. **c** Dried to remove solvent. **d** Corresponding storage and loss moduli. Rheological comparisons before and after processing indicated that the storage and loss moduli remained essentially unchanged, demonstrating performance consistency. The error bars in (**d**) represent the standard deviations from three independent experiments. Source data are provided as a Source Data file.

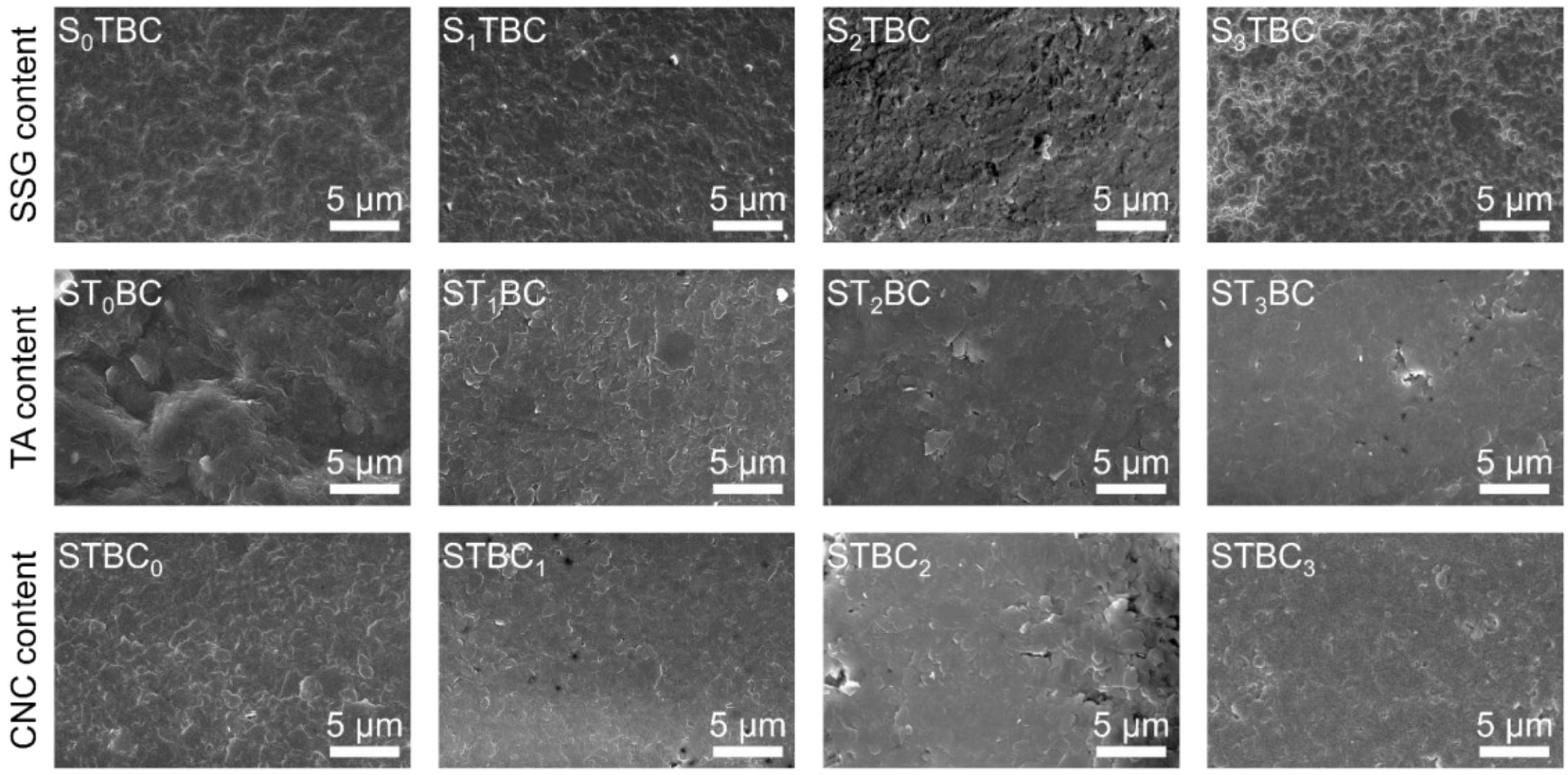

Supplementary Fig. 3 | Top-view microstructures of STBC as a function of material parameters. Specimens with BNNS-to-SSG mass ratios of 1:0, 4:1, 2:1 and 1:1 were designated S_iTBC (i = 0-3); those with TA contents of 0, 0.0078, 0.0155, and 0.0310 wt% are denoted ST_jBC (j = 0-3); and those with CNC contents of 0, 0.0155, 0.0310, and 0.0620 wt% are denoted $STBC_k$ (k = 0-3). All specimens prepared under these conditions exhibited well-ordered stacked microstructures, highlighting the versatility and robustness of the preparation method.

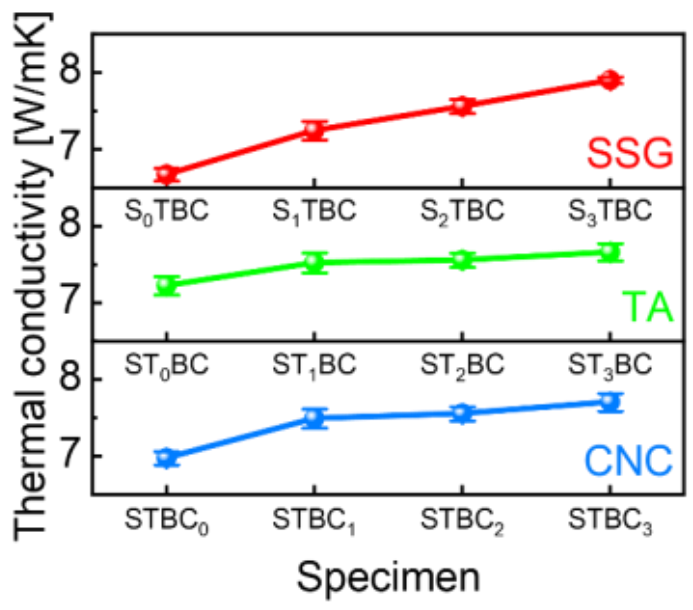

Supplementary Fig. 4 | Thermal conductivity of STBC as a function of material parameters. The error bars represent the standard deviations from three independent experiments. Source data are provided as a Source Data file.

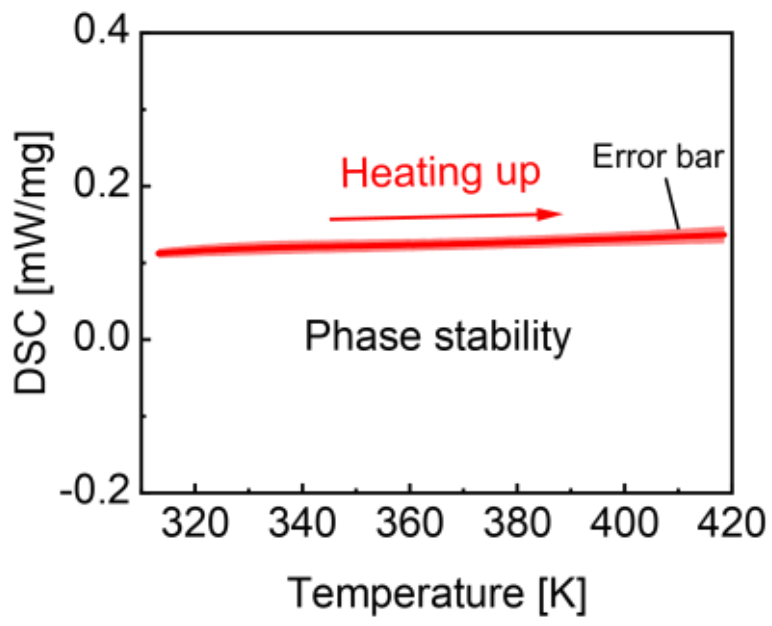

Supplementary Fig. 5 | Temperature-dependent differential scanning calorimetry (DSC) profile of STBC. The error bar represents the standard deviations from three independent experiments. Source data are provided as a Source Data file.

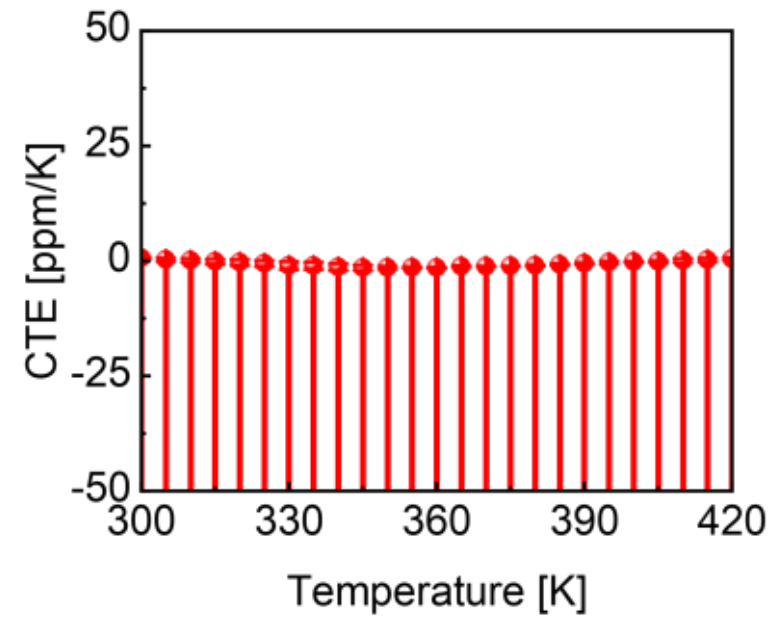

Supplementary Fig. 6 | Coefficients of thermal expansion (CTE) for STBC from 300 to 420 K. The error bars represent the standard deviations from three independent experiments. Source data are provided as a Source Data file.

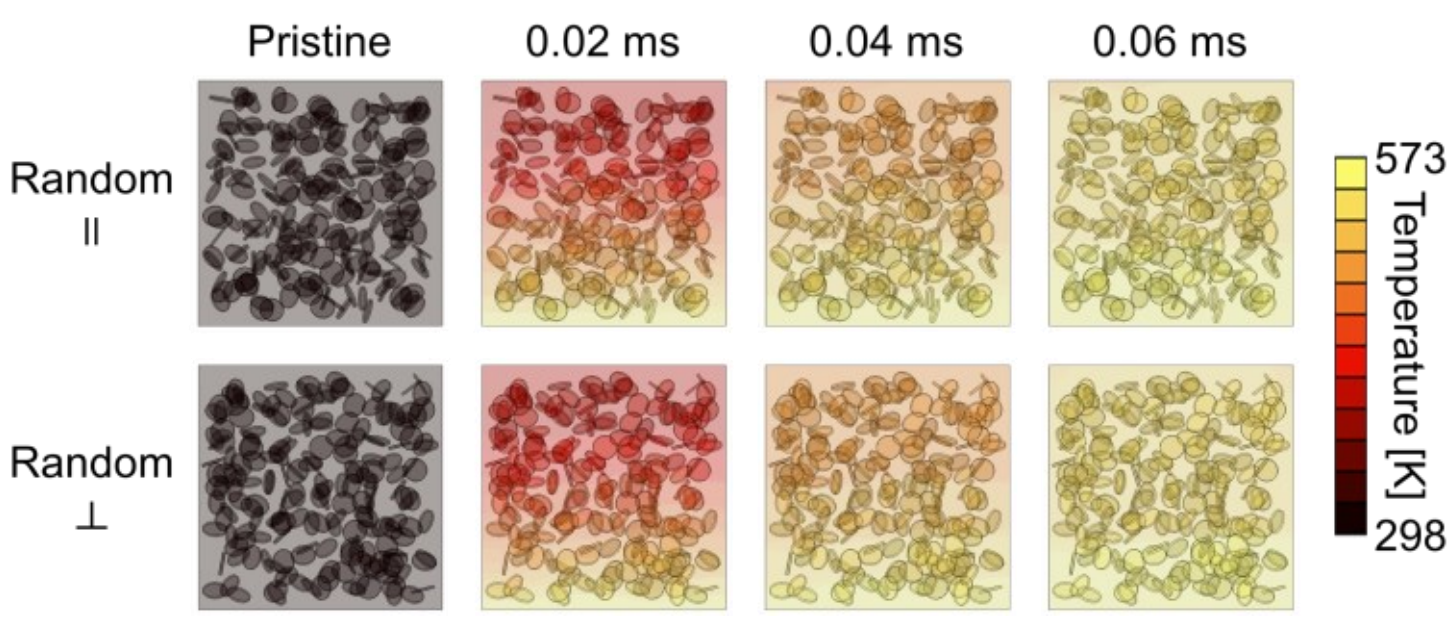

Supplementary Fig. 7 | Temporal temperature maps of the R-model in the parallel and perpendicular directions. All material parameters used in the Abaqus 2021 simulations were sourced from the literature[1,2].

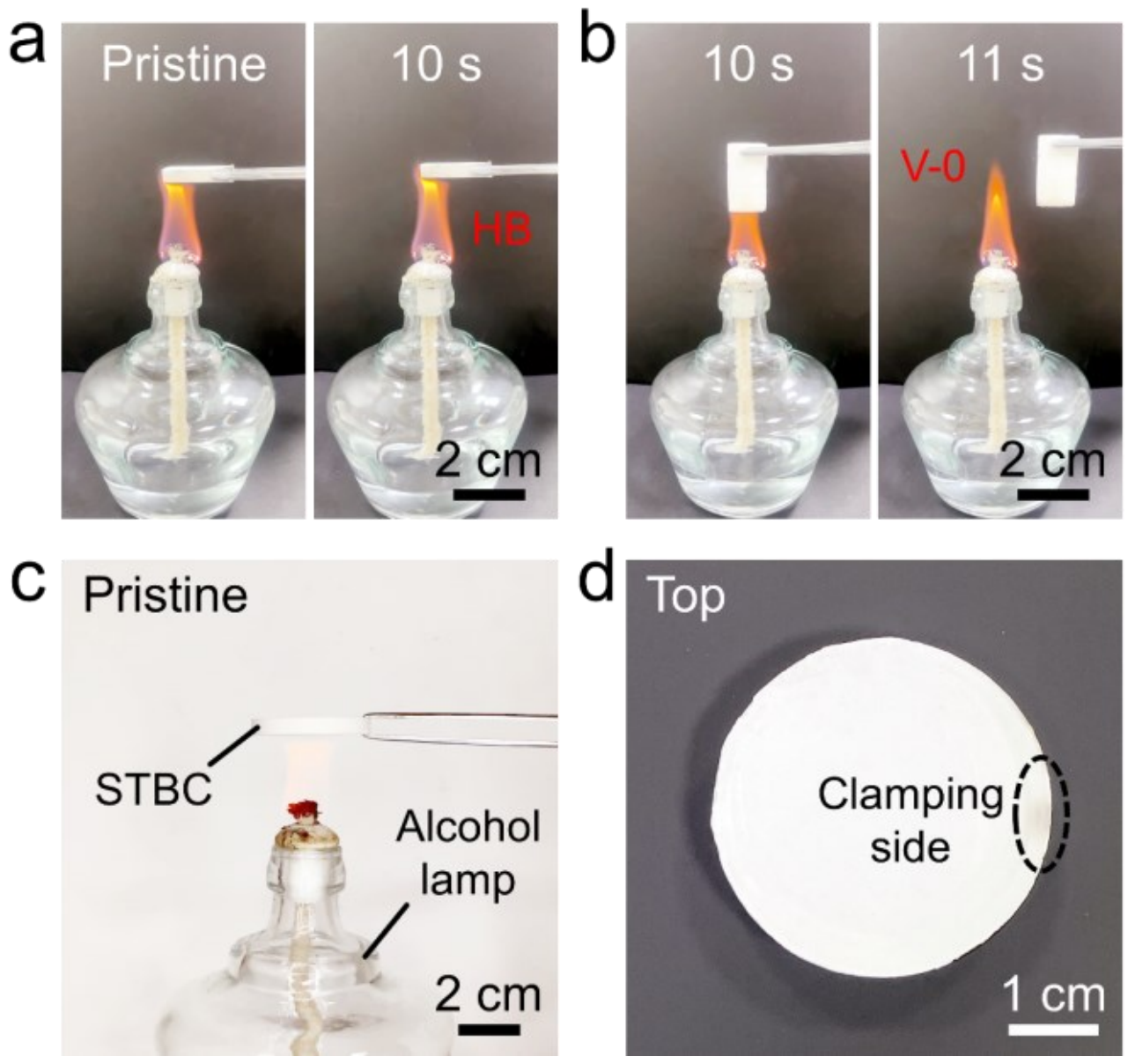

Supplementary Fig. 8 | Flame-retardant behavior and post-combustion morphology of STBC. a Horizontal and **b** vertical burning of STBC. In the horizontal burning test, STBC exhibited no observable combustion. In the vertical test, STBC rapidly self-extinguished after two 10 s ignition events without melt dripping. **c** Flame-impact resistance of STBC. **d** Top-surface morphology of STBC post-combustion.

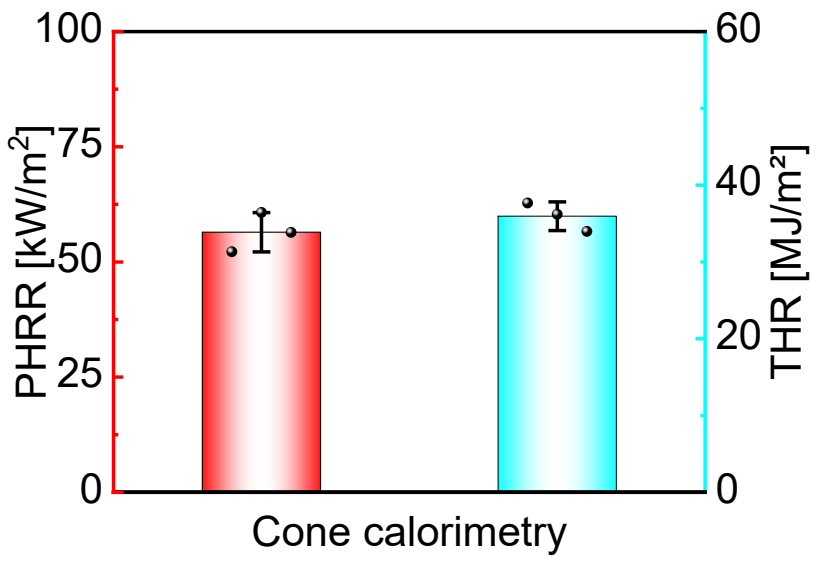

Supplementary Fig. 9 | Peak heat release rate and total heat release of STBC under fire exposure. The error bars represent the standard deviations from three independent experiments. Source data are provided as a Source Data file.

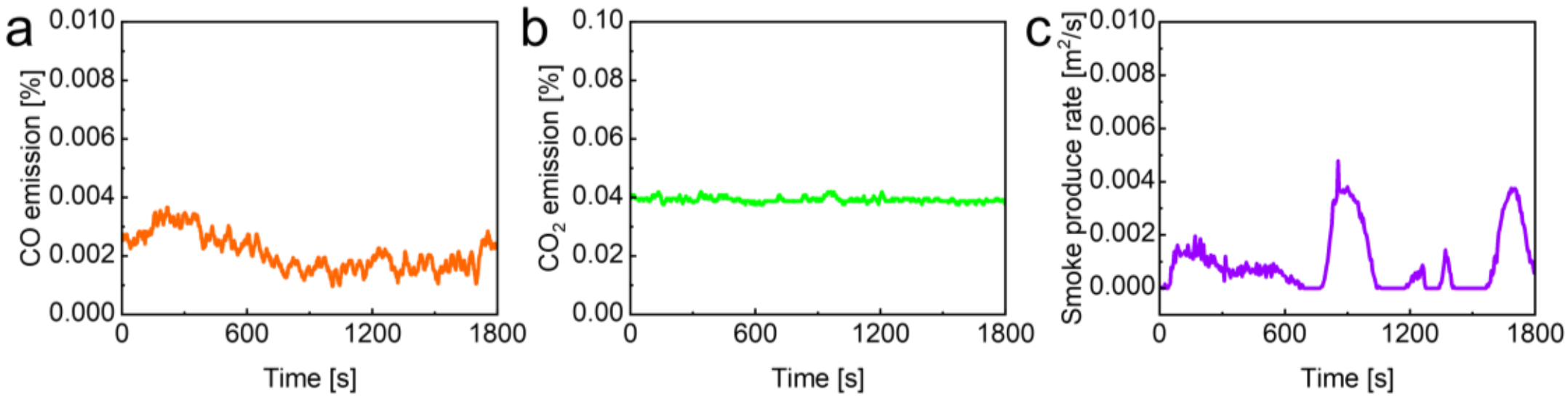

Supplementary Fig. 10 | Fire-emission behavior of STBC under combustion. Real-time **a** CO and **b** CO_2 emissions and **c** smoke produce rate of STBC during fire exposure. Source data are provided as a Source Data file.

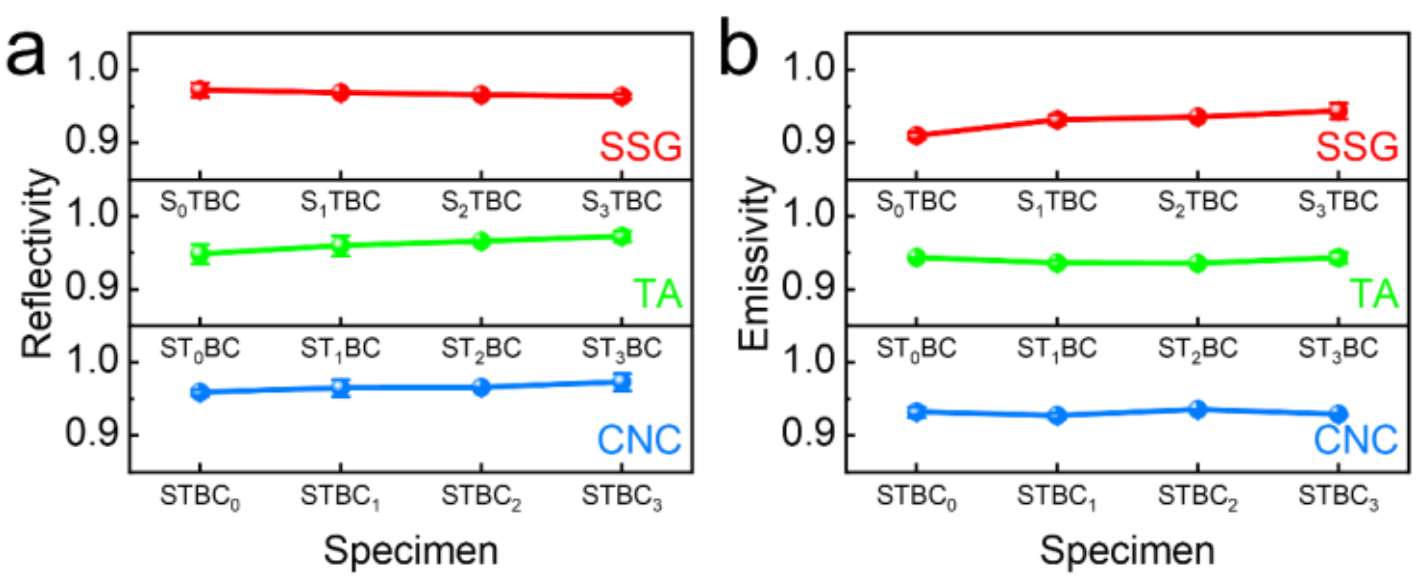

Supplementary Fig. 11 | Optical radiative-cooling properties of STBC with different material parameters. a Reflectivity and **b** emissivity of STBC as a function of material parameters. The error bars represent the standard deviations from three independent experiments. Source data are provided as a Source Data file.

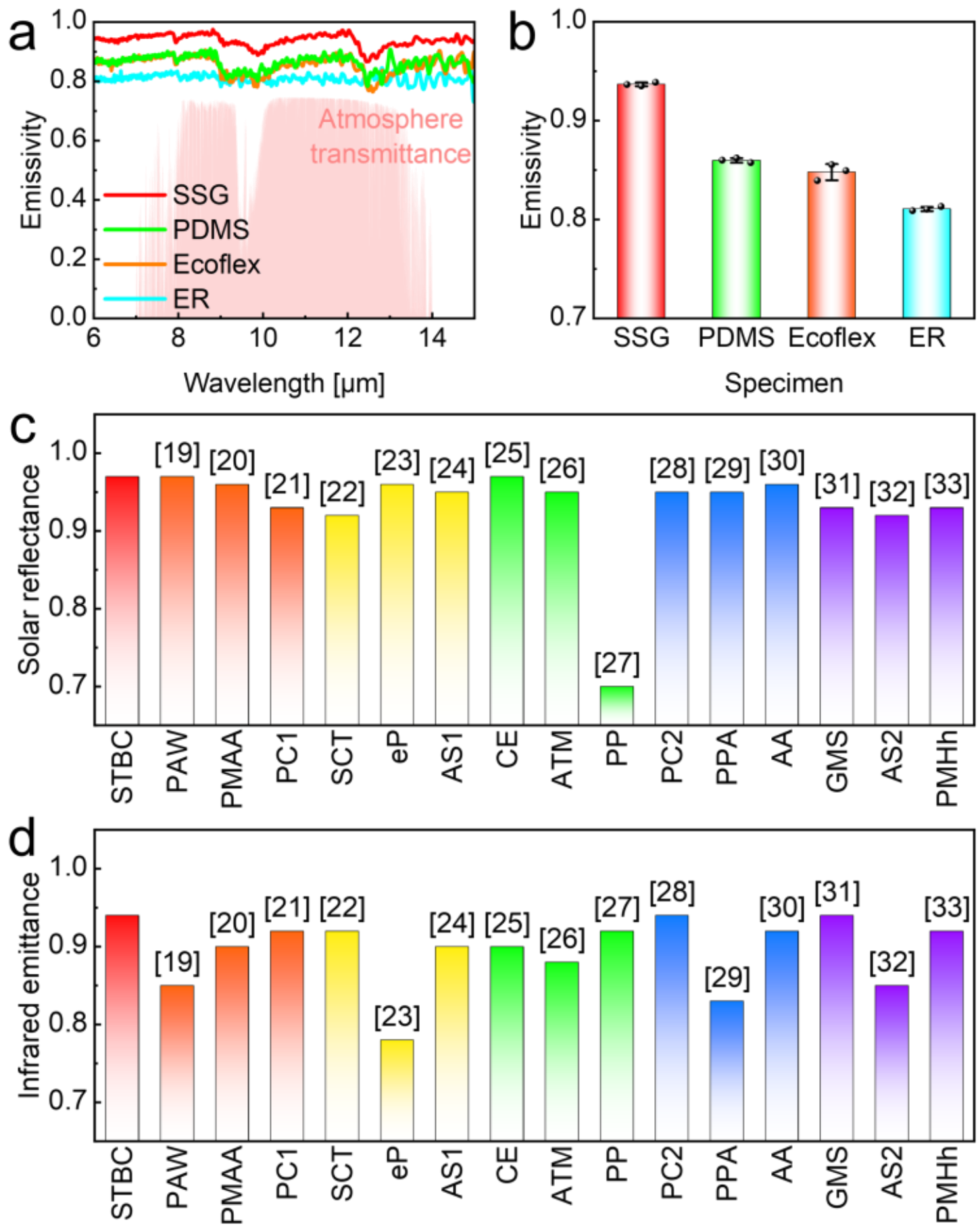

Supplementary Fig. 12 | Radiative-cooling performance of SSG and STBC compared with representative materials. a Emissivity-wavelength spectra and **b** emissivity comparison of SSG versus high-molecular-weight PDMS, Ecoflex, and epoxy resin (ER) engineering polymers. **c** Solar reflectance and **d** infrared emittance of STBC compared with various radiative cooling composites[19-33]. The error bars in (**b**) represent the standard deviations from three independent experiments. Source data are provided as a Source Data file.

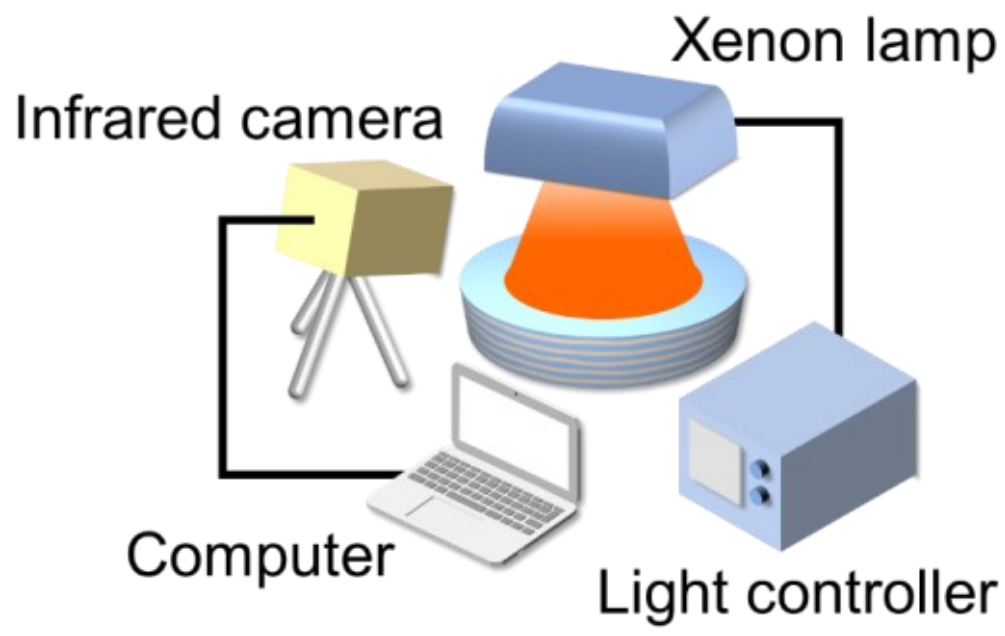

Supplementary Fig. 13 | Schematic of the in-situ light-exposure temperature measurement platform. The images were created using 3ds Max 2022 and Microsoft PowerPoint 2016.

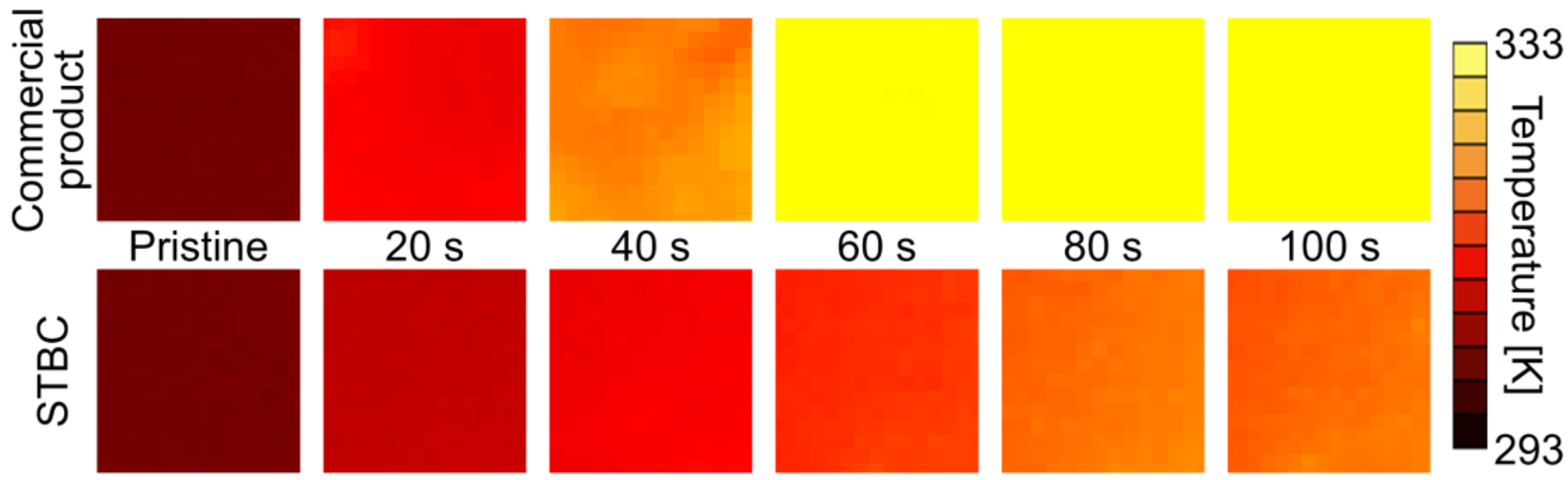

Supplementary Fig. 14 | Infrared images of a commercial radiative cooling product and STBC under 1 sun exposure.

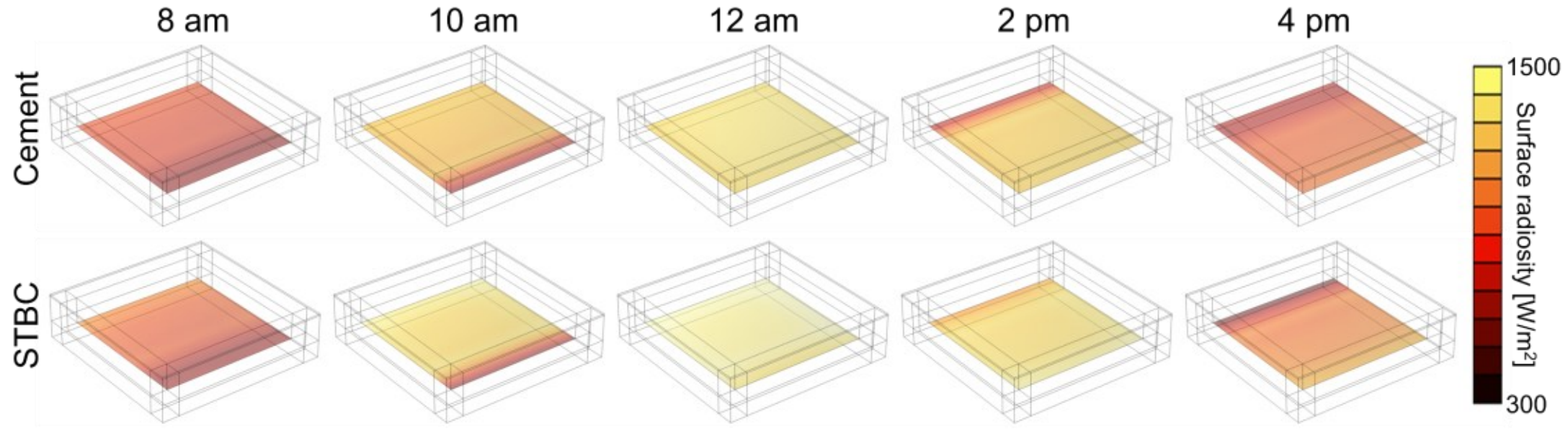

Supplementary Fig. 15 | Surface radiosity patterns of commercial cement and STBC obtained via finite element simulation with surface-to-surface radiation heat transfer.

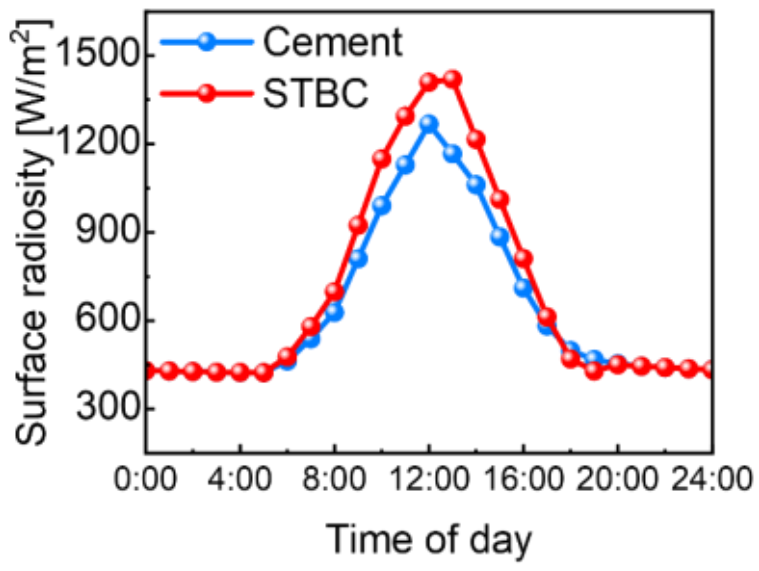

Supplementary Fig. 16 | Surface radiosity of cement and STBC models over a 1-day cycle. Source data are provided as a Source Data file.

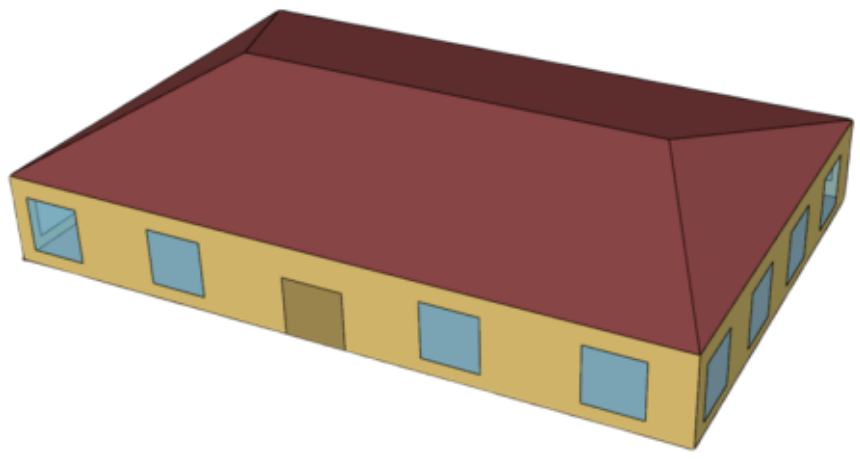

Supplementary Fig. 17 | Schematic of the STBC-integrated building model used for EnergyPlus simulations. The image was created using 3ds Max 2022 and Microsoft PowerPoint 2016.

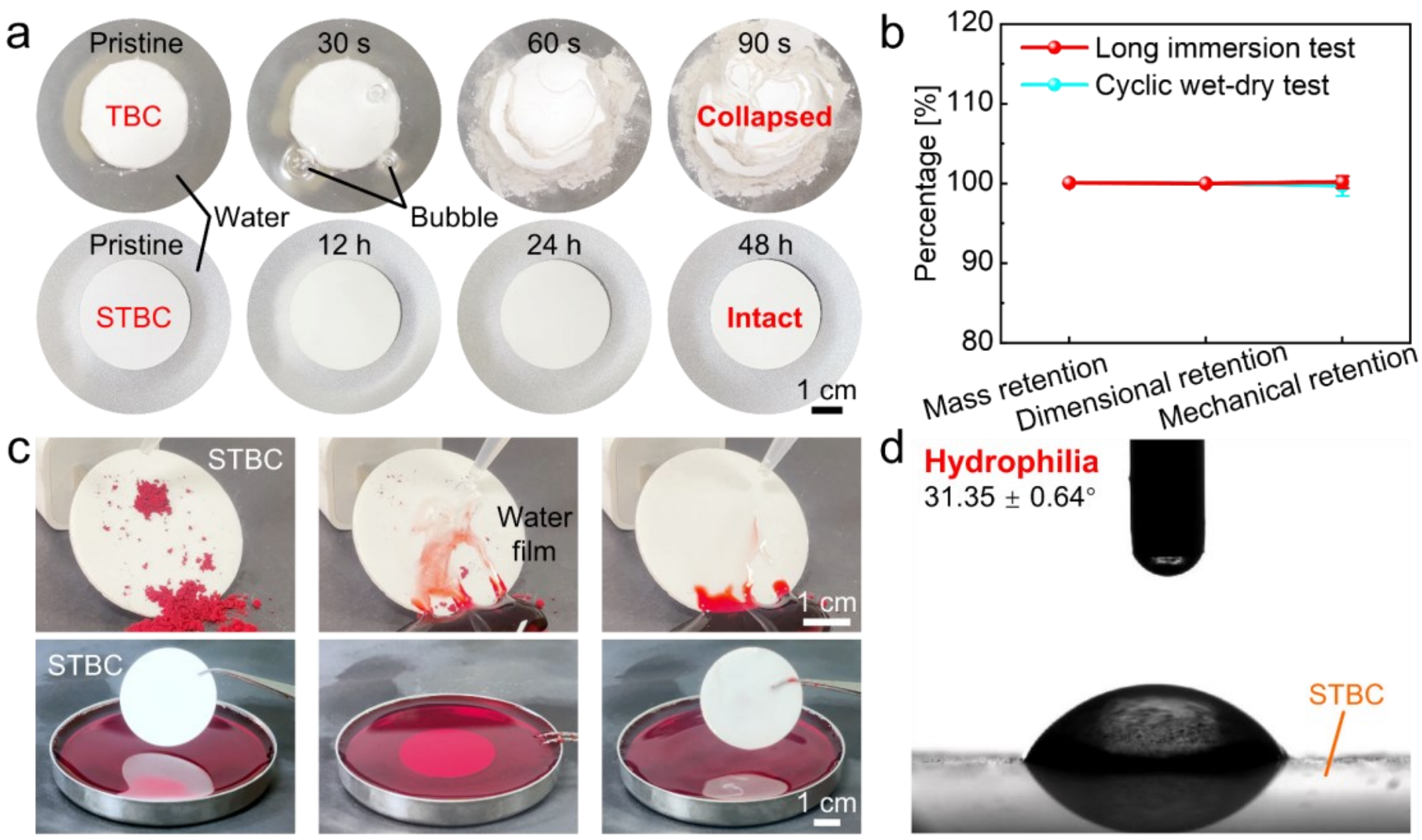

Supplementary Fig. 18 | Environmental durability and surface wettability of STBC. a Water resistance of TBC and STBC. **b** Mass, dimensional, and mechanical retention of STBC after prolonged immersion and cyclic wet-dry tests. STBC underwent 10 wet-dry cycles consisting of 5 min immersion in water followed by 5 min drying at 353 K. **c** Self-cleaning and **d** hydrophilic properties of STBC. The error bars in (**b**) represent the standard deviations from three independent experiments. Source data are provided as a Source Data file.

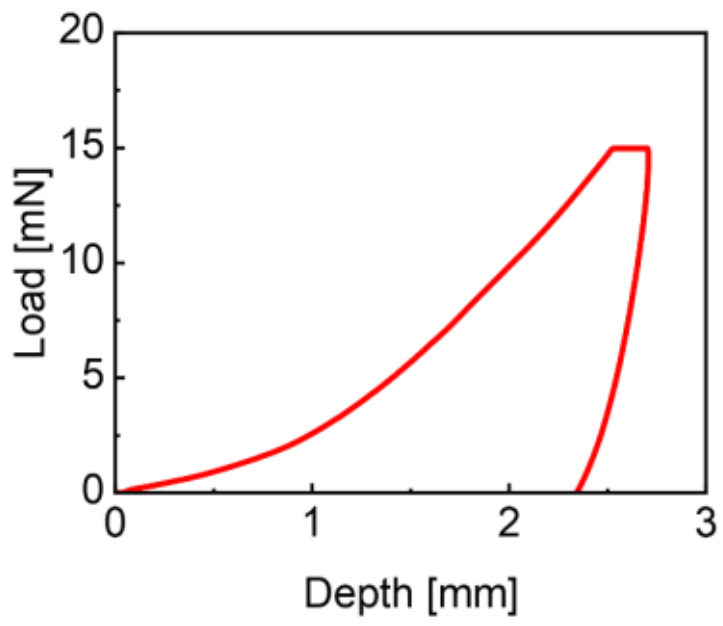

Supplementary Fig. 19 | Load-depth curve of STBC in the nanoindentation test. Source data are provided as a Source Data file.

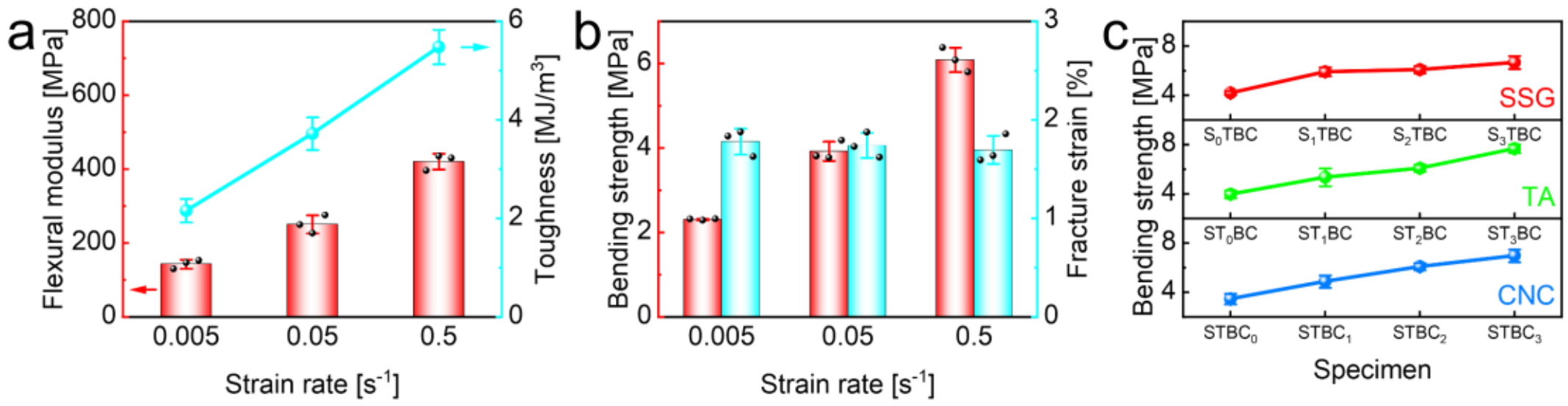

Supplementary Fig. 20 | Strain-rate-dependent bending properties of STBC. a Flexural moduli and toughness, **b** bending strengths and fracture strains of STBC at strain rates of 0.005, 0.05 and 0.5 s^{-1}. **c** Bending strength of STBC as a function of material parameters. The error bars represent the standard deviations from three independent experiments. Source data are provided as a Source Data file.

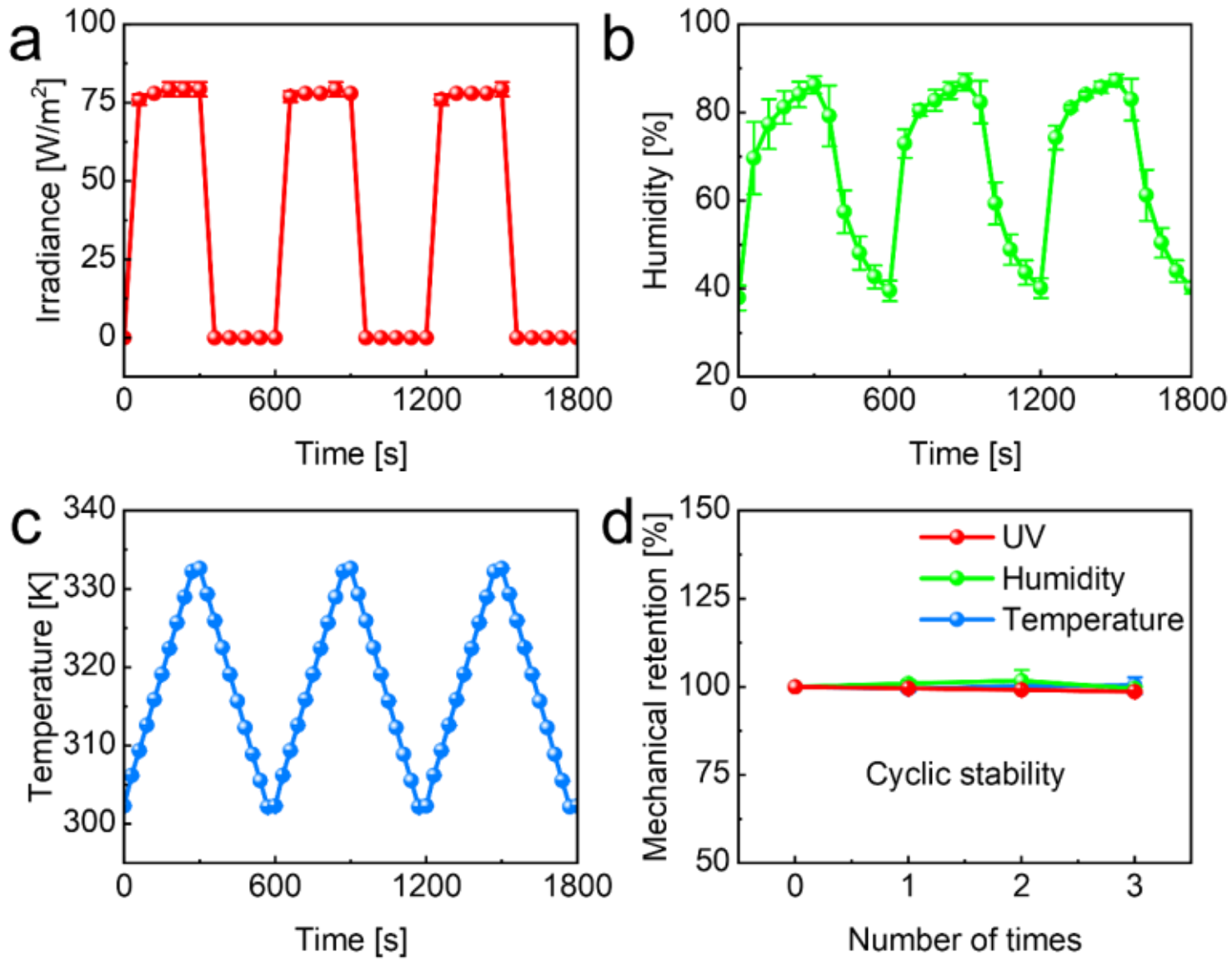

Supplementary Fig. 21 | Environmental stability and mechanical retention of STBC. a UV, **b** humidity, and **c** temperature cycling tests, and **d** corresponding mechanical retention of STBC. The error bars represent the standard deviations from three independent experiments. Source data are provided as a Source Data file.

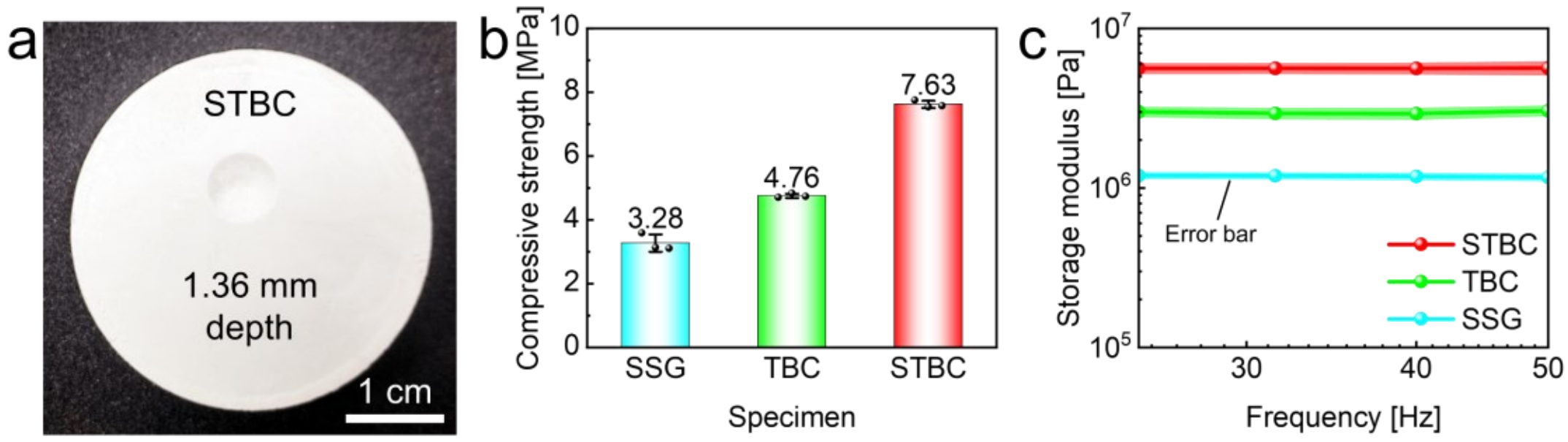

Supplementary Fig. 22 | Impact-hardening behavior and dynamic mechanical properties of STBC. a Surface morphology of STBC after projectile impact. **b** Compressive strength of SSG, TBC, and STBC at 2000 s^{-1} from SHPB tests. **c** Storage modulus of SSG, TBC, and STBC from DMA. The compressive strength and storage modulus of STBC were both higher than those of the individual subcomponents. These results indicated that incorporating SSG to form STBC enhanced mechanical performance compared with TBC, conferring greater rigidity and impact resistance under high-rate loading[1,3]. The error bars in (**b**) and (**c**) represent the standard deviations from three independent experiments. Source data are provided as a Source Data file.

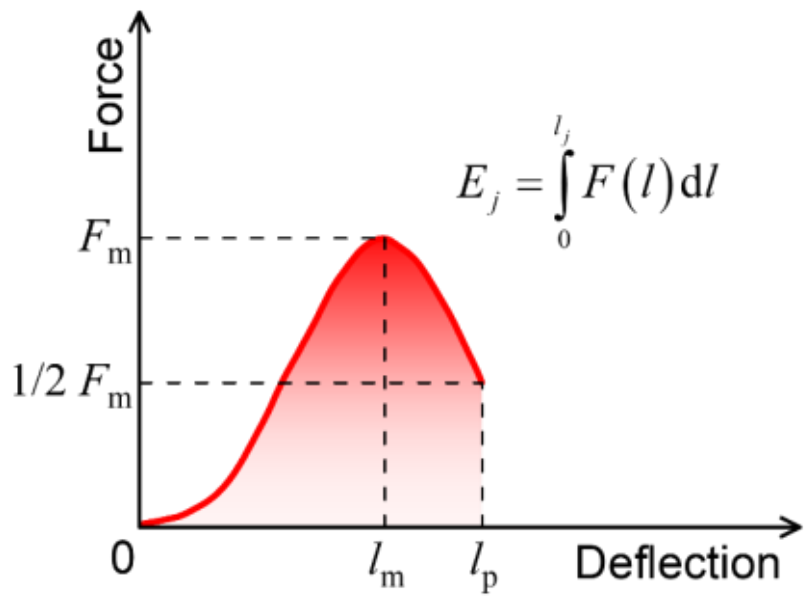

Supplementary Fig. 23 | Schematic of force-deflection curves under puncture loading.

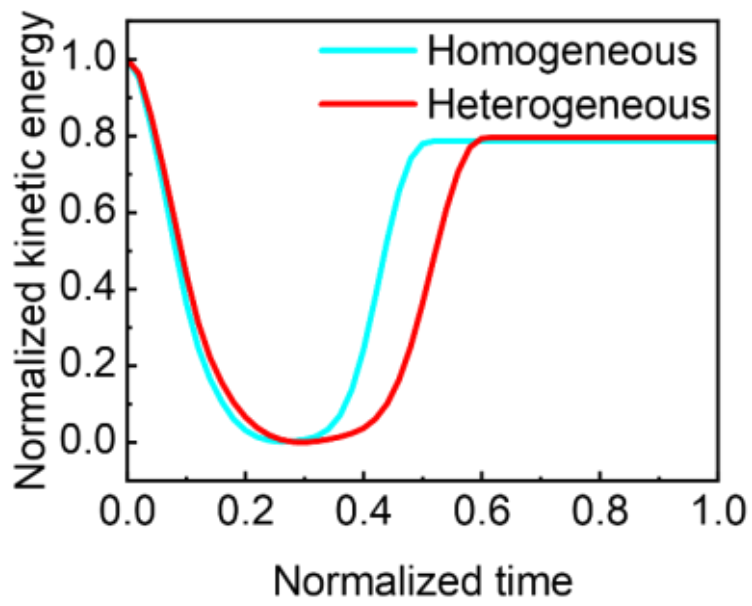

Supplementary Fig. 24 | Normalized kinetic energies of homogeneous and heterogeneous structures obtained via explicit dynamic finite element analysis. Source data are provided as a Source Data file.

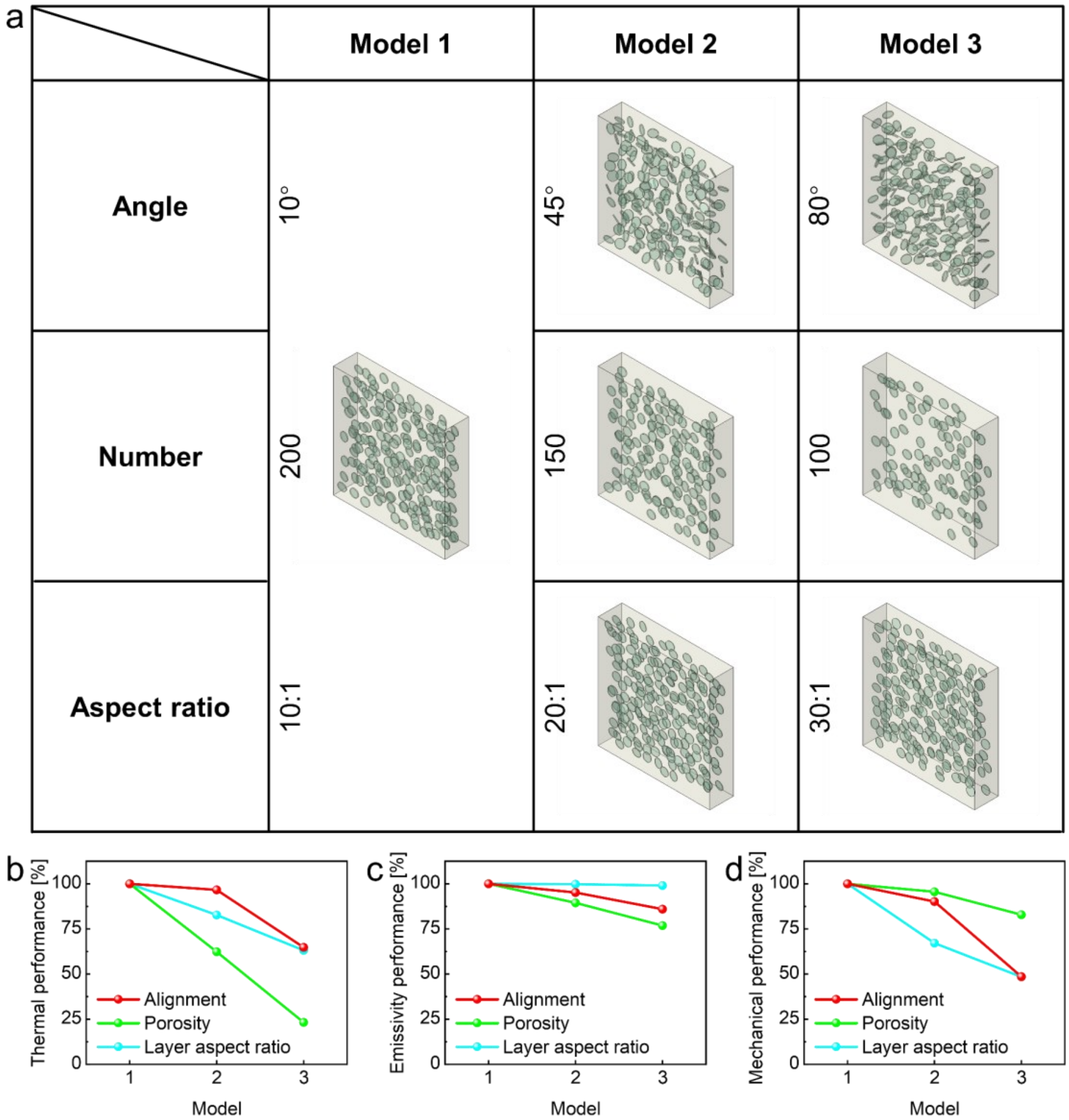

Supplementary Fig. 25 | Parametric modeling and performance optimization of STBC. a Parametric modeling of STBC. **b** Thermal, **c** emissivity, and **d** mechanical performance as a function of model parameters. Source data are provided as a Source Data file.

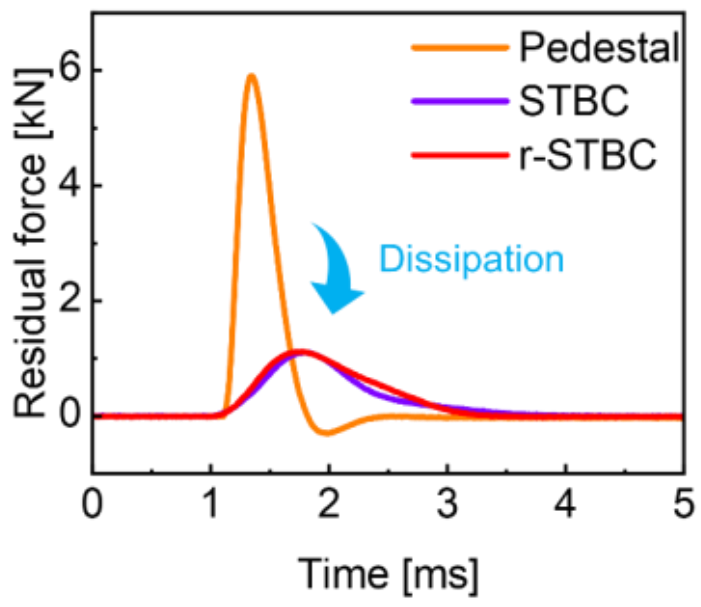

Supplementary Fig. 26 | Residual force profiles of the pedestal, STBC and r-STBC in drop-tower experiments. Source data are provided as a Source Data file.

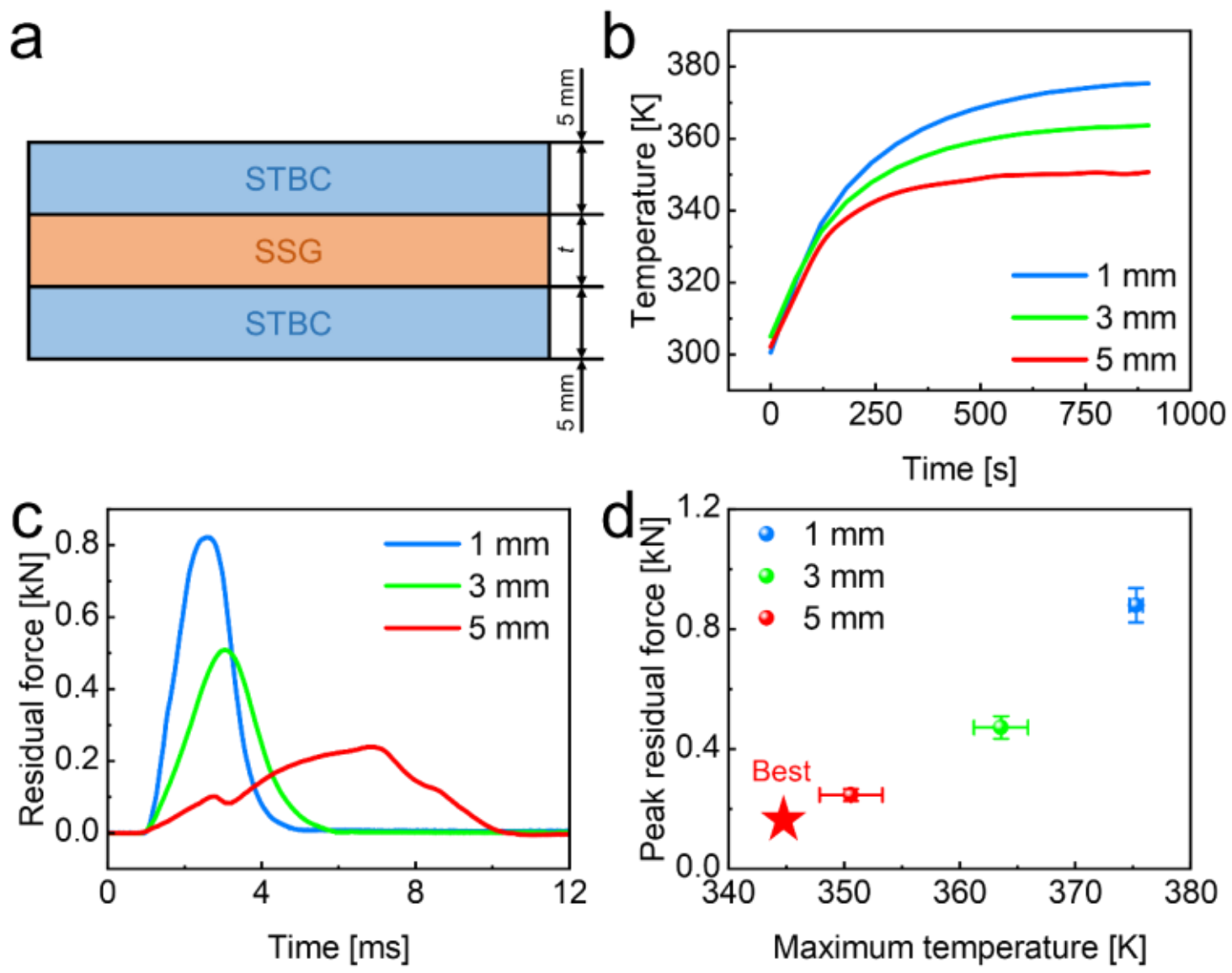

Supplementary Fig. 27 | Structural design and thermo-mechanical performance of s-STBC with different interlayer thicknesses. **a** s-STBC structural design schematic. **b** Temperature-time curves, **c** residual force-time curves, and **d** thermo-mechanical comparison of s-STBC with SSG interlayer thickness of 1, 3, and 5 mm. These results confirmed that interlayer thickness was pivotal for regulating the multifunctional properties of s-STBC. The error bars in (**d**) represent the standard deviations from three independent experiments. Source data are provided as a Source Data file.

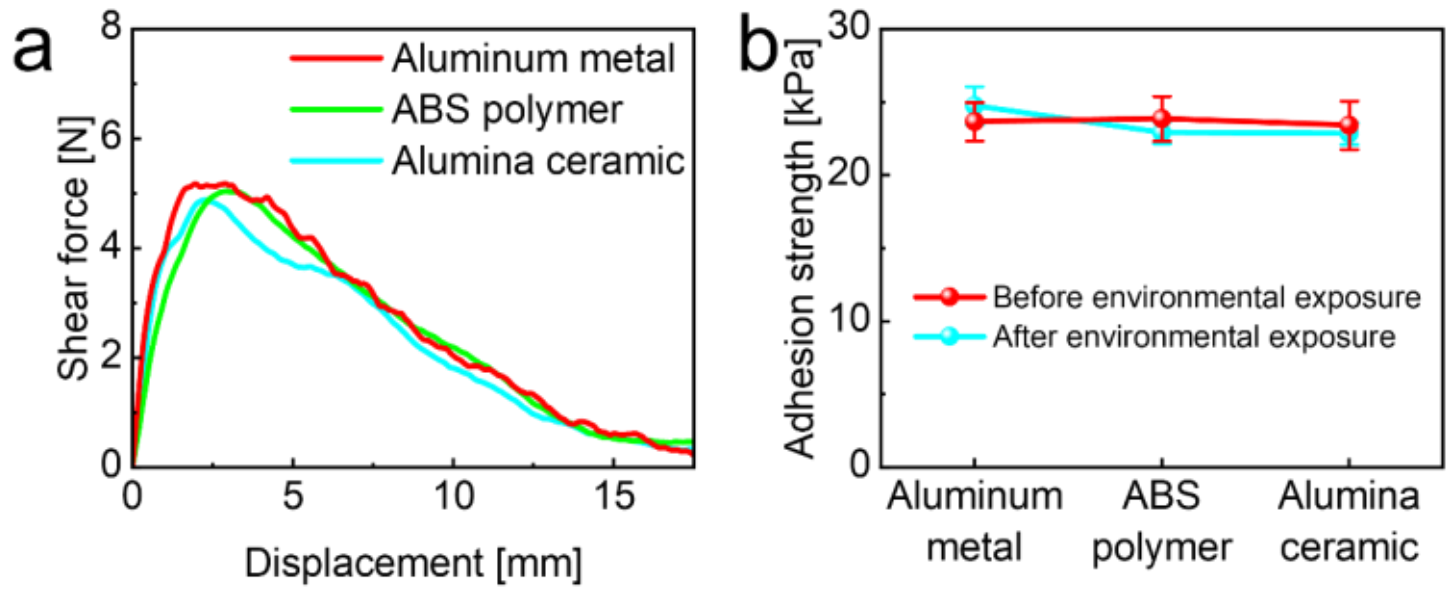

Supplementary Fig. 28 | Adhesion behavior and environmental robustness of STBC on representative substrates. a Shear force-displacement of STBC adhered to representative substrates, and **b** adhesion strength before and after environmental exposure under single-lap shear testing. The error bars in (**b**) represent the standard deviations from three independent experiments. Source data are provided as a Source Data file.

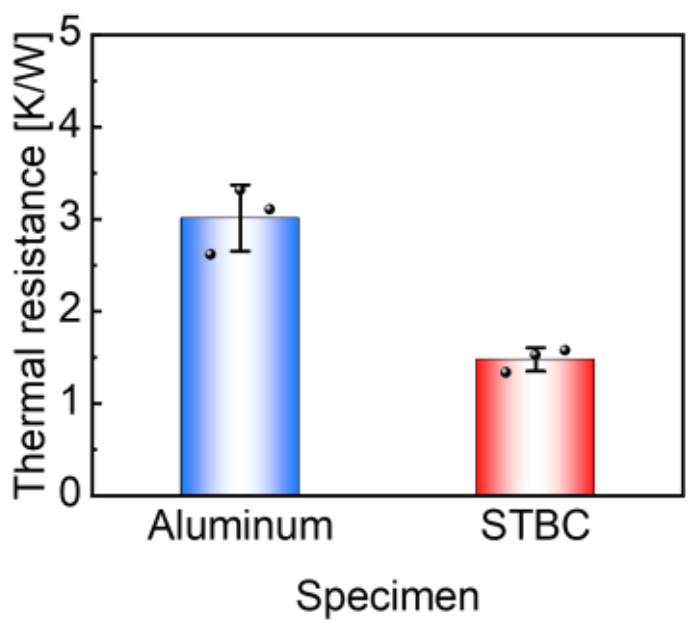

Supplementary Fig. 29 | Comparison of thermal resistance for realistic device stacks employing aluminum versus STBC per ASTM D 5470. STBC was better suited for device-level thermal interface management than aluminum, validating its potential in thermal conduction and heat-dissipation components. The error bars represent the standard deviations from three independent experiments. Source data are provided as a Source Data file.

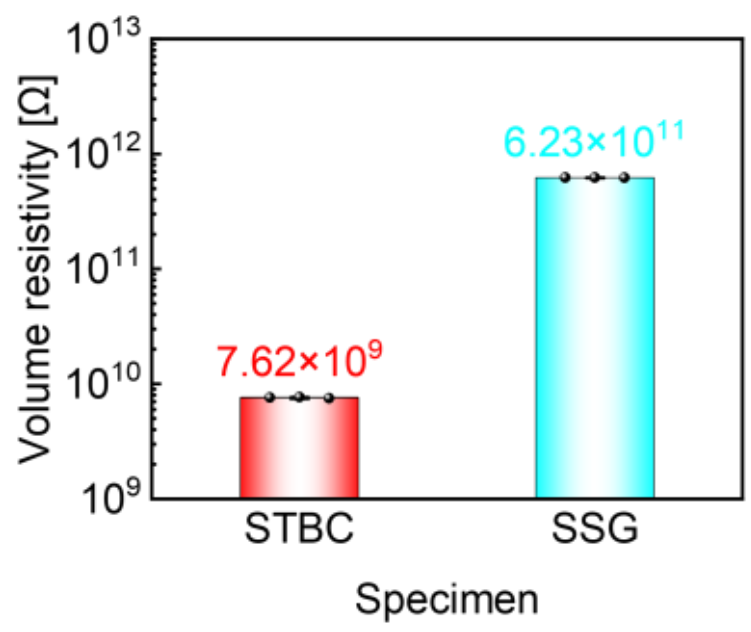

Supplementary Fig. 30 | Volume resistivities of STBC and SSG. The error bars represent the standard deviations from three independent experiments. Source data are provided as a Source Data file.

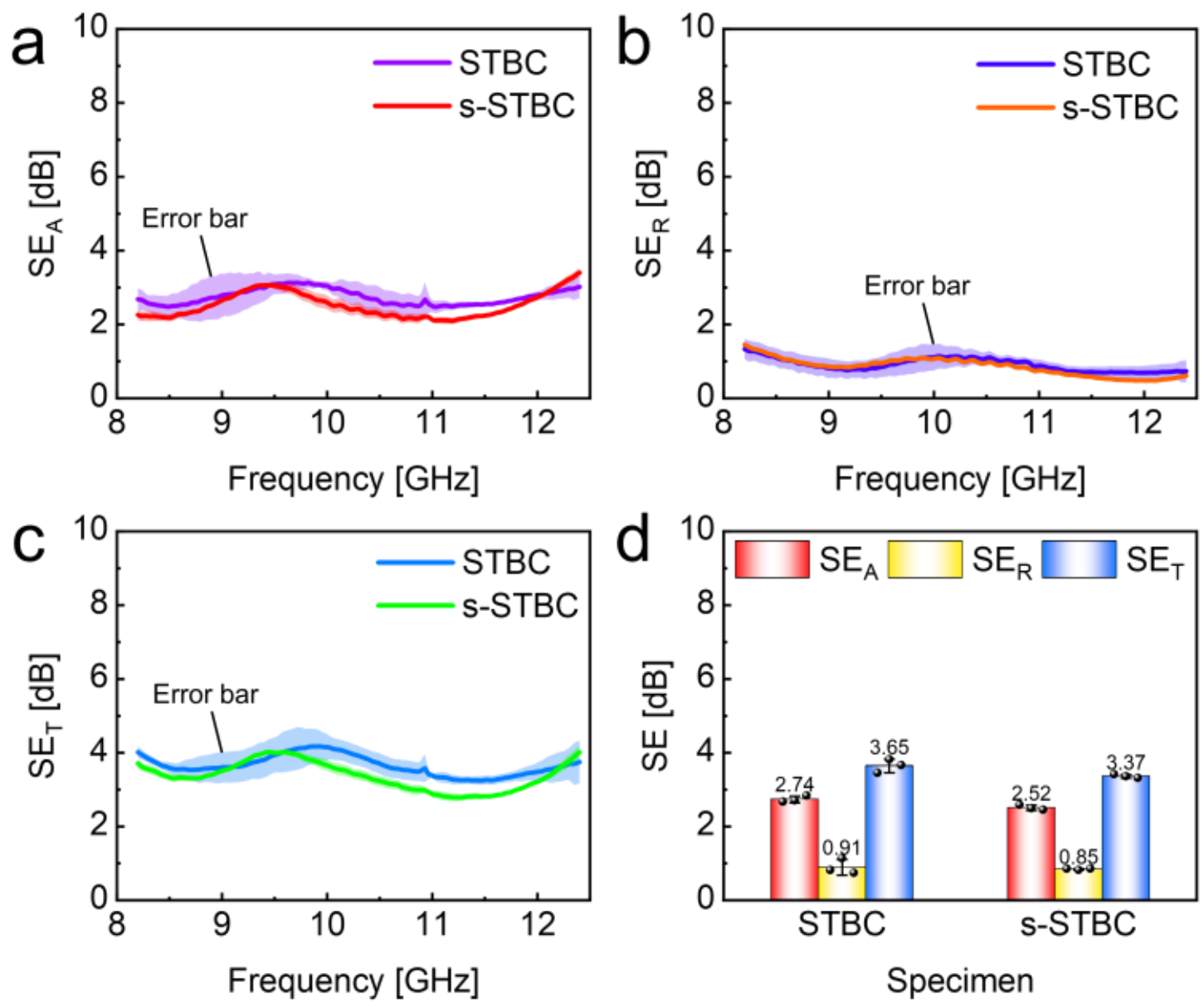

Supplementary Fig. 31 | Electromagnetic interference (EMI) shielding performance of STBC and s-STBC. a Absorption shielding effectiveness (SE_A), **b** reflection shielding effectiveness (SE_R), and **c** total shielding effectiveness (SE_T) of s-STBC. **d** Comparison of EMI shielding performance between STBC and s-STBC. The error bars represent the standard deviations from three independent experiments. Source data are provided as a Source Data file.

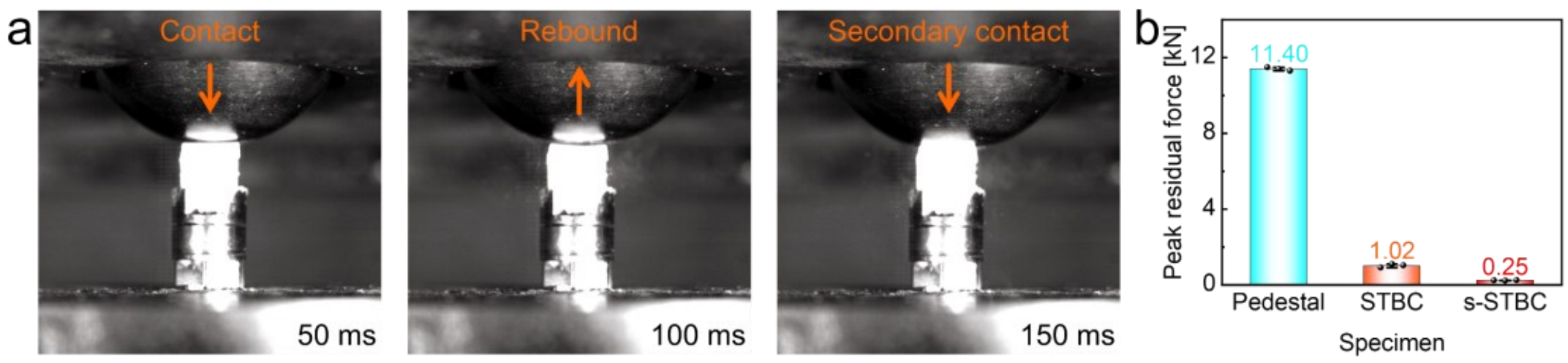

Supplementary Fig. 32 | Heavy-impact resistance of STBC and s-STBC. a 3.2 kg impact sequence captured by high-speed imaging and **b** peak residual force of the pedestal, STBC, and s-STBC. Source data are provided as a Source Data file.

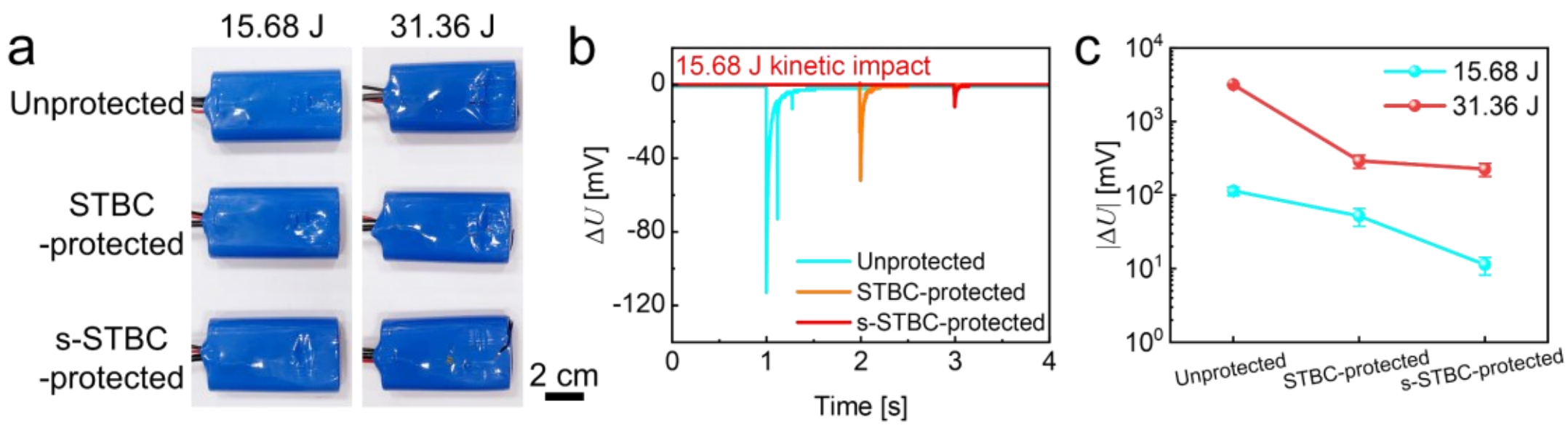

Supplementary Fig. 33 | Battery-protection performance of STBC and s-STBC under impact loading. a Damaged states, **b** temporal voltage traces, and **c** comparisons of voltage change (ΔU) for unprotected, STBC-protected, and s-STBC-protected batteries. The error bars in (**c**) represent the standard deviations from three independent experiments. Source data are provided as a Source Data file.

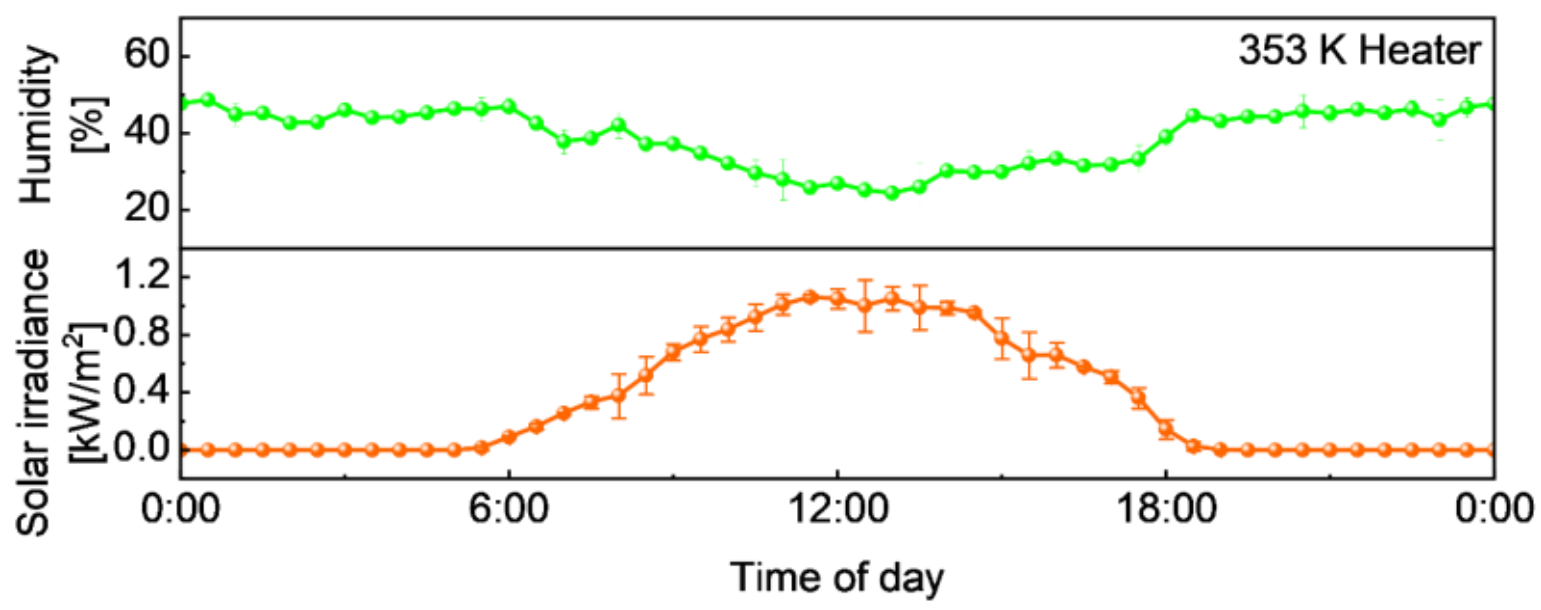

Supplementary Fig. 34 | Diurnal profiles of solar irradiance and ambient humidity in Hefei, China. The error bars represent the standard deviations from three independent experiments. Source data are provided as a Source Data file.

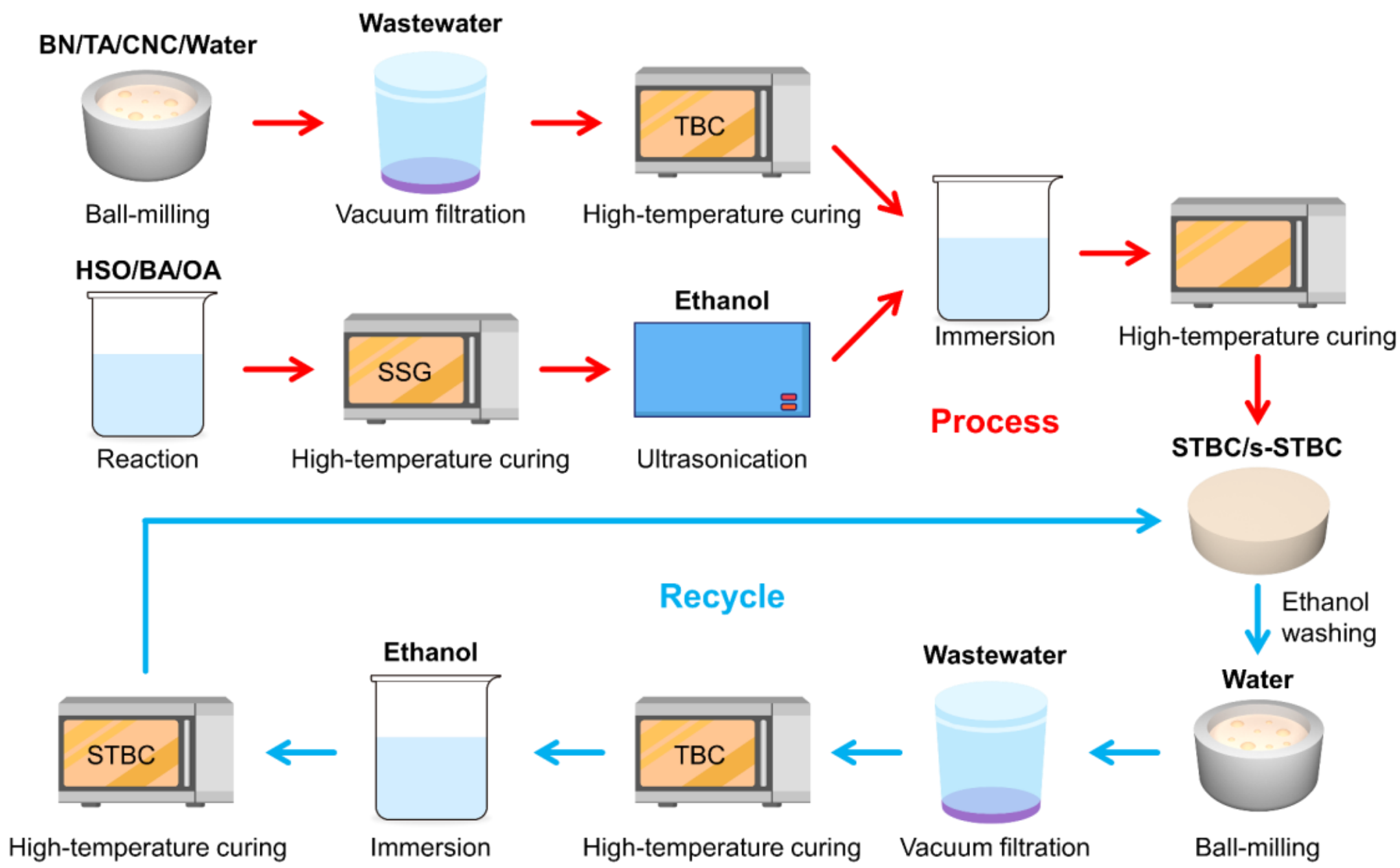

Supplementary Fig. 35 | System boundary of STBC and s-STBC. The images were created using 3ds Max 2022 and Microsoft PowerPoint 2016.

Supplementary Table 1 | Thermal anisotropy of STBC and reported thermal interface materials.

Specimen	Thermal anisotropy	Reference
**STBC (This work)**	**44.47**	**This work**
LM/ABS (LA)	27.2	4
AgNWs@BNNS/ANF (ABA)	12.59	5
A-SiC@SiO_2-FAs (ASSF)	5.08	6
Kaolin/Al/CNF (KAC)	34.19	7
BNNS/WPU (BW)	5.8	8
PBA-PDMS/FGf (PPF)	1.57	9
FCS	5.6	10
PAN/OSZ (PO)	3.2	11
AH-SSCSNWA (AS)	2.5	12
PVA/Graphite (PG)	22.00	13
GN/PVA (GP)	23.00	14
(BNNS/ANF)-(AgNWs@BNNS)-(BNNS/ANF) (BAB)	11.14	15
LC-CNT/LC-PI (LCLP)	7.31	16
AP/MBLM (AM)	18.55	17
LC-(FG@CNT)/LC-PI (LFCLP)	7.45	18

LM: Low-melting-point metal; ABS: Acrylonitrile butadiene styrene; CNF: Cellulose nanofiber; WPU: Waterborne polyurethane; PBA: 2-[[(butylamino)carbonyl] oxy]ethyl ester; PDMS: Polydimethylsiloxane; FGf: Folded graphene film; PAN: Polyacrylonitrile copolymer; OSZ: Oligosilazane; PVA: Polyvinyl alcoho; GN: Graphene@naphthalenesulfonate; BNNS: Boron nitride nanosheets; ANF: Aramid nanofiber; LC: Liquid crystal; CNT: Carbon nanotube; PI: Polyimid; AP: Aramid nanofiber/polyvinyl alcohol; MBLM: MXene-bridging-liquid metal; FG: Fluorinated graphene; PI: Polyimide.

Supplementary Table 2 | Radiative cooling performance of STBC and various reported advanced cooling composites.

Specimen	Solar reflectance	Infrared emittance	Reference
**STBC (This work)**	**0.97**	**0.94**	**This work**
PMP/AgNWs/Wool (PAW)	0.97	0.85	19
PMAA	0.96	0.9	20
PMMA/CPC (PC1)	0.93	0.92	21
$SrAl_2O_4$/CNC@Textile (SCT)	0.92	0.92	22
es-PEO (eP)	0.96	0.78	23
Al_2O_3/Silk (AS1)	0.95	0.9	24
CNC-EC (CE)	0.97	0.9	25
ANF/TiO_2-Mica (ATM)	0.95	0.88	26
PDMS/PAM (PP)	0.70	0.92	27
PVA-$CaCl_2$ (PC2)	0.95	0.94	28
POM-PTFE-Al (PPA)	0.95	0.83	29
Al_2O_3/Al (AA)	0.96	0.92	30
GG/MF/SiO_2 (GMS)	0.93	0.94	31
AgNWs/SEBS (AS2)	0.92	0.85	32
PDMS/MCI/HDI/h-BN (PMHh)	0.93	0.92	33

PMP: Polymethylpentene; CPC: Colloidal photonic crystal; CNC: Cellulose nanocrystal; PEO: Polyethylene oxide; EC: Ethylcellulose; ANF: Aramid nanofiber; PDMS: Polydimethylsiloxane; PAM: Polyacrylamide; PVA: Poly(vinyl alcohol); POM: Polyformaldehyde; PTFE: Polytetrafluoroethylene; GG: Gellan gum; MF: Melamine-formaldehyde; AgNWs: Silver nanowires; SEBS: Polystyrene-block-poly(ethylene-ran-butylene)-block-polystyrene; BN: Boron nitride; MCI: Methylene bis(cyclohexylisocyanate); HDI: Hexamethylene diisocyanate.

Supplementary Table 3 | Energy-efficiency outcomes for global cities with and without STBC protection, evaluated using EnergyPlus simulations.

City	Unprotected cooling energy [MJ m^{-2}]	STBC-protected cooling energy [MJ m^{-2}]	Cooling energy saving [%]
Albuquerque	98.32	48.05	51.13
Anchorage	22.85	6.67	70.81
Antofatasga	118.45	60.87	48.61
Beijing	92.87	51.18	44.89
Berlin	43.37	18.67	56.95
Brasilia	164.03	96.00	41.47
Cairo	166.43	94.90	42.98
Calgary	42.68	17.15	59.82
Cape Town	110.62	58.47	47.14
Denver	73.87	33.60	54.51
Fairbanks	28.82	9.35	67.56
Hanoi	210.15	125.83	40.12
Hong Kong	177.63	105.97	40.34
Kunming	92.05	42.77	53.54
La Paz	49.47	7.18	85.49
London	38.82	14.80	61.88
Los Angeles	105.08	48.30	54.04
Mexico City	108.50	51.95	52.12
Moscow	42.75	17.18	59.81
New York	86.53	47.50	45.11
Oslo	33.50	12.05	64.03
Phoenix	177.58	99.70	43.86
Rome	98.85	50.05	49.37
Salt Lake City	82.32	40.63	50.64
San Francisco	69.40	25.78	62.85
Shanghai	110.15	62.77	43.01
Stockholm	34.98	13.65	60.98
Turpan	111.17	57.37	48.39
Whitehorse	21.92	6.05	72.40
Yakutsk	36.68	15.13	58.75

Supplementary Table 4 | Impact resistance of STBC compared with state-of-the-art and high-performance engineering materials.

Specimen	Maximum force [kN m^{-1}]	Specific puncture energy [J m^{-1}]	Reference
**STBC**	**61.10**	**107.64**	**This work**
PVA/GO (PG1)	58.25	/	34
ANF	25.43	98.28	35
PPGTD-IPDA (PI1)	37.25	/	36
MNH	11.7	10.8	37
Si_3N_4	1.84	/	38
NH	2.3	0.55	39
PDO-IP2.5 (PI2)	≈56	/	40
DNH	0.8	0.53	41
HPDU10 (H10)	32.13	/	42
Hex[5A] (H5A)	31.25	37.5	43
Leather/SSG/Kevlar/ STF/NWF (LSKSN)	32	/	44
PL-Gel (PG2)	28.95	2.6	45
AA/HEMA/PEG (AHP)	22.1	/	46
EVA	7.51	100.30	The data were derived from this work.
SSE	10.45	93.81	
CB	20.40	89.12	
Wood	56.61	75.12	
EPE	4.37	34.56	
PU	0.76	8.02	
PVC/NBR (PN)	0.86	4.74	

Both maximum force and specific puncture energy were normalized by specimen thickness. PVA: Poly(vinyl alcohol); GO: Graphene oxide; ANF: Aramid nanofiber; PPGTD: PPG, tolylene 2,4-diisocyanate terminated; IPDA: Isophoronediamine; MNH: Microsphere-reinforced nanocomposite hydrogel; NH: Nanocomposite hydrogel; PDO: 2, 4-pentanedione dioxime; DNH: Double network hydrogel; HPDU: Hyperbranched polyurethane; SSG: Shear-stiffening gel; STF: Shear-thickening fluid; NWF: Non-woven fabric; AA: Acrylic acid; HEMA: Hydroxyethyl methacrylate; PEG: Polyethylene glycol; EVA: Ethylene vinyl acetate copolymer; SSE: Shear-stiffening elastomer[47]; CB: Corrugated board; EPE: Expanded polyethylene; PU: Polyurethane; PVC: Polyvinyl chloride; NBR: Nitrile butadiene rubber. The engineering specimens used for comparison were commercially available products.

Supplementary Table 5 | Comparison of the multi-functional properties of STBC and r-STBC.

Parameter	r-STBC	Performance percentage
Hardness	89.67 ± 0.58 HA	98.53 ± 0.63%
Impact resistance	83.15 ± 1.94%	102.17 ± 2.39%
Thermal conductivity	7.41 ± 0.10 W m^{-1} K^{-1}	98.06 ± 1.33%
Solar reflectance	0.96 ± 0.01	98.63 ± 1.19%
Mid-infrared emittance	0.94 ± 0.01	99.65 ± 0.61%

Supplementary Table 6 | Comparative dielectric performance of s-STBC and various electrical insulation materials.

Specimen	Average permittivity
**s-STBC (This work)**	**1.9519 ± 0.0217**
**STBC (This work)**	**1.9806 ± 0.0083**
PTFE	2.0594 ± 0.0011
PE	2.1913 ± 0.0029
SR	3.1193 ± 0.0105
PVC	3.4053 ± 0.010
TIM	5.2972 ± 0.1273
RCM	6.0344 ± 0.0550

PTFE: Polytetrafluoroethylene; PE: Polyethylene; SR: Silicone rubber; PVC: Polyvinyl chloride; TIM: Thermal interface material; RCM: Radiative cooling material. PTFE, PE, SR, PVC were commercially available products.

Supplementary Table 7 | Life cycle inventory for the production of STBC.

Section	Material	Amount	Unit	Database
Input (Process)	BN	620.16	g	48
	TA	15.50	g	49
	CNC	31.01	g	50
	HSO	321.87	g	
	BA	10.73	g	
	OA	0.73	g	
	Ethanol	333.33	g	
	Water	3720.93	g	
	Electricity (Ball milling)	0.20	kWh	Ecoinvent v3.10
	Electricity (Vacuum filtration)	0.36	kWh	
	Electricity (High-temperature curing)	0.36	kWh	
	Electricity (Ultrasonication)	0.06	kWh	
Output (Process)	STBC	1000.00	g	
	Wastewater	3720.93	g	Ecoinvent v3.10
Input (Recycle)	STBC	1000.00	g	
	Ethanol	2000.00	g	
	Water	3720.93	g	
	Electricity (Ball milling)	0.20	kWh	Ecoinvent v3.10
	Electricity (Vacuum filtration)	0.36	kWh	
	Electricity (High-temperature curing)	0.36	kWh	
Output (Recycle)	STBC	1000	g	
	Wastewater	3720.93	g	Ecoinvent v3.10

Electricity consumption for ball milling, vacuum filtration, and high-temperature curing was estimated from the rated power and operating time of each instrument. Initial ethanol inputs were 333.33 g and 2000.00 g, respectively. Assuming 20% consumption (66.66 g and 100.00 g), the remainder was recovered for reuse.

Supplementary Table 8 | Life cycle inventory for the production of s-STBC.

Section	Material	Amount	Unit	Database
Input (Process)	BN	449.19	g	48
	TA	11.23	g	49
	CNC	22.46	g	50
	HSO	499.34	g	
	BA	16.64	g	
	OA	1.14	g	
	Ethanol	241.44	g	
	Water	2695.16	g	
	Electricity (Ball milling)	0.14	kWh	Ecoinvent v3.10
	Electricity (Vacuum filtration)	0.26	kWh	
	Electricity (High-temperature curing)	0.28	kWh	
	Electricity (Ultrasonication)	0.04	kWh	
Output (Process)	STBC	1000	g	
	Wastewater	2695.16	g	Ecoinvent v3.10
Input (Recycle)	STBC	1000	g	
	Ethanol	1448.65	g	
	Water	2695.16	g	
	Electricity (Ball milling)	0.14	kWh	Ecoinvent v3.10
	Electricity (Vacuum filtration)	0.26	kWh	
	Electricity (High-temperature curing)	0.26	kWh	
Output (Recycle)	STBC	1000	g	
	Wastewater	2695.16	g	Ecoinvent v3.10

Electricity consumption for ball milling, vacuum filtration, and high-temperature curing was estimated from the rated power and operating time of each instrument. Initial ethanol inputs were 241.44 g and 1448.65 g, respectively. Assuming a 20% consumption (48.28 g and 289.73 g), the remainder was recovered for reuse.

Supplementary Table 9 | Life cycle environmental influences of STBC and s-STBC.

Influence category	Unit	STBC	**s-STBC**
Global warming	kg CO_2 eq	1.54E+01	**1.39E+01**
Terrestrial acidification	kg SO_2 eq	6.46E-02	**5.67E-02**
Freshwater eutrophication	kg P eq	5.50E-03	**4.85E-03**
Marine eutrophication	kg N eq	2.91E-03	**2.16E-03**
Terrestrial ecotoxicity	kg 1,4-DCB	4.98E+02	**4.35E+02**
Freshwater ecotoxicity	kg 1,4-DCB	7.48E-01	**6.32E-01**
Marine ecotoxicity	kg 1,4-DCB	1.39E+00	**1.20E+00**
Human carcinogenic toxicity	kg 1,4-DCB	2.15E+00	**1.89E+00**
Human non-carcinogenic toxicity	kg 1,4-DCB	1.67E+01	**1.43E+01**
Land use	m^2a crop eq	1.20E+00	**9.57E-01**
Mineral resource scarcity	kg Cu eq	7.90E-02	**6.41E-02**
Fossil resource scarcity	kg oil eq	4.35E+00	**3.80E+00**
Water consumption	m^3	4.61E-01	**3.52E-01**

Functional unit: 1 kg of material.

Supplementary Table 10 | Life cycle environmental influences of raw materials for STBC and s-STBC.

Influence category	Section	Carbon footprint of STBC [kg CO_2 eq]	Water footprint of STBC [m^3]	Carbon footprint of s-STBC [kg CO_2 eq]	Water footprint of s-STBC [m^3]
BN	Input (Process)	7.90E+00	2.21E-01	5.72E+00	1.60E-01
TA		8.80E-03	2.91E-04	6.38E-03	2.11E-04
CNC		2.75E+00	2.12E-01	2.00E+00	1.53E-01
HSO		3.26E+00	2.22E-02	5.05E+00	3.44E-02
BA		7.99E-03	1.19E-04	1.24E-02	1.85E-04
OA		1.09E-03	9.79E-06	1.70E-03	1.52E-05
Ethanol		8.02E-02	3.51E-04	5.81E-02	2.54E-04
Water		9.59E-04	1.93E-03	6.95E-04	1.40E-03
Electricity		4.76E-01	1.25E-03	3.47E-01	9.09E-04
Wastewater	Output (Process)	5.70E-04	-1.86E-03	4.13E-04	-1.35E-03
Ethanol	Input (Recycle)	4.81E-01	2.11E-03	3.48E-01	1.53E-03
Water		9.59E-04	1.93E-03	6.95E-04	1.40E-03
Electricity		4.44E-01	1.16E-03	3.22E-01	8.42E-04
Wastewater	Output (Recycle)	5.70E-04	-1.86E-03	4.13E-04	-1.35E-03
**Total**		**1.54E+01**	**4.61E-01**	**1.39E+01**	**3.52E-01**

Carbon and water footprints were quantified using the global warming and water consumption indicators, respectively.

Supplementary References

1. Li, Z. et al. Bioinspired flame-retardant and impact-resistant aramid composites via nacre-mimetic self-assembly for firefighting applications. *Adv. Mater.* **37**, 2508606 (2025).
2. Wu, N. et al. A binder-free ice template method for vertically aligned 3D boron nitride polymer composites towards thermal management. *J. Colloid Interf. Sci.* **647**, 43-51 (2023).
3. Wu, J. et al. A lightweight aramid-based structural composite with ultralow thermal conductivity and high-impact force dissipation. *Matter* **5**, 2265-2284 (2022).
4. Yu, D. et al. Volume-metallization 3D-printed polymer composites. *Adv. Mater.* **36**, 2403088 (2024).
5. Han, Y., Ruan, K. & Gu, J. Multifunctional thermally conductive composite films based on fungal tree-like heterostructured silver nanowires@boron nitride nanosheets and aramid nanofibers. *Angew. Chem. Int. Edit.* **62**, e202216093 (2022).
6. Zhang, Z. et al. Highly oriented SiC@SiO_2 ceramic fiber aerogels with good anisotropy of the thermal conductivity and high-temperature resistance. *Adv. Sci.* **12**, 2416740 (2025).
7. Zhang, Z. B., Gao, H. L., Chen, S. M. & Yu, S. H. Anisotropically thermal-protective porous ceramics enabled by nacre-like framework. *Adv. Mater.* **37**, 2506308 (2025).
8. Chan, K.-Y. et al. Scalable anisotropic cooling aerogels by additive freeze-casting. *Nat. Commun.* **13**, 5553 (2022).
9. Yu, H. et al. Highly thermally conductive polymer/graphene composites with rapid room-temperature self-healing capacity. *Nano-Micro Lett.* **14**, 135 (2022).
10. Wang, Z. Y. et al. Functional carbon springs enabled dynamic tunable microwave absorption and thermal insulation. *Adv. Mater.* **36**, 2412605 (2024).
11. Liao, X. et al. Extremely low thermal conductivity and high electrical conductivity of sustainable carbon-ceramic electrospun nonwoven materials. *Sci. Adv.* **9**, eade6066 (2023).
12. Su, L. et al. Anisotropic and hierarchical SiC@SiO_2 nanowire aerogel with exceptional stiffness and stability for thermal superinsulation. *Sci. Adv.* **6**, eaay6689 (2020).
13. Zhou, Y. et al. Defects vibrations engineering for enhancing interfacial thermal transport in polymer composites. *Sci. Adv.* **11**, eadp6516 (2025).
14. Zhuang, Y. et al. Flexible graphene nanocomposites with simultaneous highly anisotropic thermal and electrical conductivities prepared by engineered graphene with flat morphology. *ACS Nano* **14**, 11733-11742 (2020).
15. Han, Y. et al. Highly thermally conductive aramid nanofiber composite films with synchronous visible/infrared camouflages and information encryption. *Angew. Chem. Int. Edit.* **63**, e202401538 (2024).
16. Ruan, K., Shi, X., Zhang, Y., Guo, Y., Zhong, X. & Gu, J. Electric-field-induced

alignment of functionalized carbon nanotubes inside thermally conductive liquid crystalline polyimide composite films. *Angew. Chem. Int. Edit.* **62**, e202309010 (2023).
17. Sun, Y., Su, Y., Chai, Z., Jiang, L. & Heng, L. Flexible solid-liquid bi-continuous electrically and thermally conductive nanocomposite for electromagnetic interference shielding and heat dissipation. *Nat. Commun.* **15**, 7290 (2024).
18. Ruan, K. et al. Molecular brush-grafted liquid crystalline hetero-structured fillers for boosting thermal conductivity of polyimide composite films. *Adv. Funct. Mater.* **35**, 2506563 (2025).
19. Wu, R. et al. Spectrally engineered textile for radiative cooling against urban heat islands. *Science* **384**, 1203-1212 (2024).
20. Gao, W., Lei, Z., Wu, K. & Chen, Y. Reconfigurable and renewable nano-micro-structured plastics for radiative cooling. *Adv. Funct. Mater.* **31**, 2100535 (2021).
21. Hou, X. et al. Brilliant colorful daytime radiative cooling coating mimicking scarab beetle. *Matter* **8**, 101898 (2025).
22. Zhou, Y., Lu, C. & Xiong, R. Hierarchical nanocellulose photonic design for synergistic colored radiative cooling. *ACS Nano* **19**, 5029-5039 (2025).
23. Li, D. et al. Scalable and hierarchically designed polymer film as a selective thermal emitter for high-performance all-day radiative cooling. *Nat. Nanotechnol.* **16**, 153-158 (2020).
24. Zhu, B. et al. Subambient daytime radiative cooling textile based on nanoprocessed silk. *Nat. Nanotechnol.* **16**, 1342-1348 (2021).
25. Zhu, W. et al. Structurally colored radiative cooling cellulosic films. *Adv. Sci.* **9**, 2202061 (2022).
26. Xiong, L., Wei, Y., Chen, C., Chen, X., Fu, Q. & Deng, H. Thin lamellar films with enhanced mechanical properties for durable radiative cooling. *Nat. Commun.* **14**, 6129 (2023).
27. Li, J. et al. A photosynthetically active radiative cooling film. *Nat. Sustain.* **7**, 786-795 (2024).
28. Li, J. et al. A tandem radiative/evaporative cooler for weather-insensitive and high-performance daytime passive cooling. *Sci. Adv.* **8**, eabq0411 (2022).
29. Wu, X. et al. A dual-selective thermal emitter with enhanced subambient radiative cooling performance. *Nat. Commun.* **15**, 815 (2024).
30. Xiao, C. et al. Ultrabroadband and band-selective thermal meta-emitters by machine learning. *Nature* **643**, 80-88 (2025).
31. Peng, Z. C. et al. Scalable low-carbon ambient-dried foam-like aerogels for radiative cooling with extreme environmental resistance. *Adv. Mater.* **37**, 2505224 (2025).
32. Xu, Y. et al. Multiscale porous elastomer substrates for multifunctional on-skin electronics with passive-cooling capabilities. *P. Natl. Acad. Sci.* **117**, 205-213 (2019).
33. Guo, C. et al. Crack-resistant and self-healable passive radiative cooling silicone

compounds. *Adv. Mater.* **37**, 2500738 (2025).
34. Liang, X. et al. Impact-resistant hydrogels by harnessing 2D hierarchical structures. *Adv. Mater.* **35**, 2207587 (2022).
35. Wu, J. et al. Acid-assisted toughening aramid aerogel monoliths with ultralow thermal conductivity and superior tensile toughness. *Adv. Funct. Mater.* **34**, 2307072 (2023).
36. Zhang, Z., Qian, L., Cheng, J., Xie, Q., Ma, C. & Zhang, G. Room-temperature self-healing polyurea with high puncture and impact resistances. *Chem. of Mater.* **35**, 1806-1817 (2023).
37. Tan, Y. et al. Bioinspired hierarchical hydrogels engineered with extreme impact resistance. *Adv. Funct. Mater.* **35**, e08034 (2025).
38. Li, M. et al. Resilient and antipuncturing Si_3N_4 nanofiber sponge. *Nano Lett.* **23**, 1289-1297 (2023).
39. Rauner, N., Meuris, M., Zoric, M. & Tiller, J. C. Enzymatic mineralization generates ultrastiff and tough hydrogels with tunable mechanics. *Nature* **543**, 407-410 (2017).
40. Liu, X. et al. Engineered self-healable elastomer with giant strength and toughness via phase regulation and mechano-responsive self-reinforcing. *Chem. Eng. J.* **410**, 128300 (2021).
41. Zhang, H. J. et al. Tough physical double-network hydrogels based on amphiphilic triblock copolymers. *Adv. Mater.* **28**, 4884-4890 (2016).
42. Chen, X. et al. Extremely tough, puncture-resistant, transparent, and photoluminescent polyurethane elastomers for crack self-diagnose and healing tracking. *ACS Appl. Mater. Interfaces* **12**, 30847-30855 (2020).
43. Yin, Z., Hannard, F. & Barthelat, F. Impact-resistant nacre-like transparent materials. *Science* **364**, 1260-1263 (2019).
44. Fan, Z. et al. Synchronous enhancement of safety protection and impact perception in intelligent leather. *Adv. Compos. Hybrid Ma.* **8**, 146 (2025).
45. Zhang, Z. et al. In-situ localised alignment assisted salting-out enhanced ionogels with high strength, toughness and impact resistance. *Nat. Commun.* **16**, 7683 (2025).
46. Chen, G., Wu, J., Wang, Z., Zhu, H., Zhu, S. & Zhang, Q. Armored polymer-fluid gels with integrated damping and impact protection across broad temperatures. *Sci. Adv.* **11**, eadv5292 (2025).
47. Yang, J., Zhao, C., Lai, S., Wang, D. & Gong, X. Hybrid additive manufacturing of shear-stiffening elastomer composites for enhanced mechanical properties and intelligent wearable applications. *Adv. Mater.* **37**, 2419096 (2025).
48. https://epb.zibo.gov.cn/art/2019/5/16/art_2210_1669107.html.
49. Chen, H., Jiang, K., Wu, Q., Zhang, X., Qian, C. & Fan, D. Mussel byssus cuticle-inspired low-carbon footprint soybean meal adhesives with high-strength and anti-mildew performance. *Ind. Crop. Prod.* **222**, 119433 (2024).
50. Malovrh Rebec, K., Turk, J. & Kunaver, M. Quantifying the environmental

implication of cotton-fiber-based nanocrystalline cellulose: A life-cycle assessment. *Sustain. Prod. Consump.* **45**, 192-202 (2024).
