# Supplementary material for: Collaborative passive cooling of impact-hardening interfaces enabled by nacre-mimetic design
Source: Nat Commun. 2026 Jun 22;17:7823. doi: 10.1038/s41467-026-74755-w (PMC13439421; doi:10.1038/s41467-026-74755-w)
Supplement: Supplementary file 2 [file 41467_2026_74755_MOESM2_ESM.pdf]

nature portfolio

Corresponding author(s): Sheng Wang, Xinglong Gong

Last updated by author(s): May 18, 2026

Reporting Summary

Nature Portfolio wishes to improve the reproducibility of the work that we publish. This form provides structure for consistency and transparency in reporting. For further information on Nature Portfolio policies, see our Editorial Policies and the Editorial Policy Checklist.

Statistics

For all statistical analyses, confirm that the following items are present in the figure legend, table legend, main text, or Methods section.

n/a	Confirmed	
☐	☒	The exact sample size (*n*) for each experimental group/condition, given as a discrete number and unit of measurement
☐	☒	A statement on whether measurements were taken from distinct samples or whether the same sample was measured repeatedly
☒	☐	The statistical test(s) used AND whether they are one- or two-sided<br>*Only common tests should be described solely by name; describe more complex techniques in the Methods section.*
☒	☐	A description of all covariates tested
☒	☐	A description of any assumptions or corrections, such as tests of normality and adjustment for multiple comparisons
☐	☒	A full description of the statistical parameters including central tendency (e.g. means) or other basic estimates (e.g. regression coefficient) AND variation (e.g. standard deviation) or associated estimates of uncertainty (e.g. confidence intervals)
☒	☐	For null hypothesis testing, the test statistic (e.g. *F*, *t*, *r*) with confidence intervals, effect sizes, degrees of freedom and *P* value noted<br>*Give P values as exact values whenever suitable.*
☒	☐	For Bayesian analysis, information on the choice of priors and Markov chain Monte Carlo settings
☒	☐	For hierarchical and complex designs, identification of the appropriate level for tests and full reporting of outcomes
☒	☐	Estimates of effect sizes (e.g. Cohen's *d*, Pearson's *r*), indicating how they were calculated

Our web collection on statistics for biologists contains articles on many of the points above.

Software and code

Policy information about availability of computer code

Data collection	Experimental data were collected using the manufacturer-provided acquisition software integrated with the corresponding instruments. No custom code or custom algorithms were used for data collection.
Data analysis	Finite-element simulations were performed using Abaqus 2021. Building energy simulations were performed using EnergyPlus 23.2.0. Life-cycle assessment was performed using SimaPro 10.1 with the Ecoinvent 3.10 database. No unpublished custom code or algorithms were used for data analysis.

For manuscripts utilizing custom algorithms or software that are central to the research but not yet described in published literature, software must be made available to editors and reviewers. We strongly encourage code deposition in a community repository (e.g. GitHub). See the Nature Portfolio guidelines for submitting code & software for further information.

Data

Policy information about availability of data

All manuscripts must include a data availability statement. This statement should provide the following information, where applicable:

- Accession codes, unique identifiers, or web links for publicly available datasets
- A description of any restrictions on data availability
- For clinical datasets or third party data, please ensure that the statement adheres to our policy

All relevant data generated from this study are available from the article and Supplementary Information. Source data are provided with this paper.

Research involving human participants, their data, or biological material

Policy information about studies with human participants or human data. See also policy information about sex, gender (identity/presentation), and sexual orientation and race, ethnicity and racism.

Reporting on sex and gender	This study did not involve human participants, human data or human biological materials. Therefore, sex- and gender-related information was not collected or analyzed.
Reporting on race, ethnicity, or other socially relevant groupings	This study did not involve human participants, human data or human biological materials. Therefore, race, ethnicity or other socially relevant groupings were not collected or analyzed.
Population characteristics	No human participants were included. Therefore, participant-level characteristics were not collected or analyzed.
Recruitment	No human participants were recruited for this materials science study.
Ethics oversight	Ethics approval for human participant research was not required because no human participants, human data or human biological materials were involved.

Note that full information on the approval of the study protocol must also be provided in the manuscript.

Field-specific reporting

Please select the one below that is the best fit for your research. If you are not sure, read the appropriate sections before making your selection.

☐ Life sciences ☐ Behavioural & social sciences ☒ Ecological, evolutionary & environmental sciences

For a reference copy of the document with all sections, see nature.com/documents/nr-reporting-summary-flat.pdf

Ecological, evolutionary & environmental sciences study design

All studies must disclose on these points even when the disclosure is negative.

Study description	This is a materials science study evaluating laboratory-prepared nacre-mimetic composites for passive cooling and impact protection. Quantitative data were obtained by comparing predefined material groups, structural configurations and testing conditions, including composition, architecture, temperature, strain rate, irradiation and impact loading. The design was mainly comparative rather than factorial or hierarchical. Experimental units were independently prepared specimens or protected device assemblies. Where applicable, three independent specimens were tested per condition and data are reported as mean ± s.d.
Research sample	Samples were laboratory-prepared STBC and s-STBC composites, together with relevant control, recycled, commercial comparison specimens and protected device assemblies. These samples were selected to evaluate whether nacre-mimetic and sandwich structural designs can integrate passive cooling, thermal/electrical insulation, environmental durability and impact protection for outdoor devices. They represent laboratory-fabricated protective materials and device-level configurations, not biological or ecological populations. No organisms, biological specimens or environmental samples were collected or manipulated. Organism taxa, source, sex and age are not applicable. Existing datasets included publicly available EnergyPlus climate data, literature-derived comparison data and Ecoinvent life-cycle inventory data.
Sampling strategy	Samples were assigned to predefined composition, structural-design or testing-condition groups according to the experimental design. For quantitative measurements, three independently prepared specimens were tested where applicable and data are reported as mean ± s.d., unless otherwise stated. No statistical sample-size calculation was performed because this materials study did not involve biological or ecological populations, nor hypothesis testing requiring power analysis. The sample sizes were considered sufficient because they follow standard practice in materials characterization and enabled assessment of experimental reproducibility across independently prepared specimens.
Data collection	Data were recorded by trained authors using instrument-integrated acquisition software on the corresponding materials-characterization, mechanical-testing, thermal-analysis, optical-spectroscopy, electrical-testing and infrared-imaging instruments. Simulation data were generated using finite-element, EnergyPlus and life-cycle assessment tools. Outdoor passive-cooling data were collected from July 2, 2025 to July 3, 2025 in Hefei, China, with temperature recording during the test period. No separate sample cohorts or collection gaps were involved.
Timing and spatial scale	Most experiments were performed at the laboratory specimen or device scale. Outdoor passive-cooling measurements were conducted from July 2, 2025 to July 3, 2025 in Hefei, China. No ecological field sites, habitats, populations or environmental sampling transects were involved.
Data exclusions	No data were excluded from the analyses.
Reproducibility	Key experiments were repeated using independently prepared specimens under the same protocols where applicable. Quantitative measurements were generally performed on three independent samples and reported as mean ± s.d., unless otherwise stated. The repeated tests showed consistent trends across independent specimens, supporting the reproducibility of the experimental findings.

	All attempts to repeat the reported experiments were successful, and no repeat experiments were excluded.
Randomization	Randomization was not relevant because specimens were assigned to predefined composition, structural-design or testing-condition groups. No ecological populations, organisms or field samples were allocated to treatment groups.
Blinding	Blinding was not used because the study relied on instrument-based materials characterization, testing and simulation under predefined protocols. No subjective ecological or biological outcomes were assessed.

Did the study involve field work? ☒ Yes ☐ No

Field work, collection and transport

Field conditions	Outdoor device-level passive-cooling measurements were conducted under direct sunlight in Hefei, China. An s-STBC sample was attached to a heated device maintained at 353 K, and surface temperatures were monitored from July 2, 2025 to July 3, 2025 using thermohygrometers. The maximum solar irradiance was 1.06 kW m^-2, and the average relative humidity was approximately 40%. No ecological or biological sampling was involved.
Location	The outdoor experiment was conducted in Hefei, Anhui, China, in an open-air device-level testing setting. No ecological field site, protected area, habitat, water body or biological sampling location was involved.
Access & import/export	No habitats, organisms, biological specimens or environmental samples were accessed, collected, transported, imported or exported. The outdoor test only involved laboratory-prepared specimens and a heated device. Therefore, no field collection or import/export permit was required.
Disturbance	The study caused no disturbance to habitats, organisms or ecological systems. The outdoor test involved only temporary placement of laboratory-prepared specimens, a heated device and sensors, all of which were removed after measurement.

Reporting for specific materials, systems and methods

We require information from authors about some types of materials, experimental systems and methods used in many studies. Here, indicate whether each material, system or method listed is relevant to your study. If you are not sure if a list item applies to your research, read the appropriate section before selecting a response.

Materials & experimental systems

n/a	Involved in the study	
☒	☐	Antibodies
☒	☐	Eukaryotic cell lines
☒	☐	Palaeontology and archaeology
☒	☐	Animals and other organisms
☒	☐	Clinical data
☒	☐	Dual use research of concern
☒	☐	Plants

Methods

n/a	Involved in the study	
☒	☐	ChIP-seq
☒	☐	Flow cytometry
☒	☐	MRI-based neuroimaging

Plants

Seed stocks	No seed stocks or plant specimens were used. Cellulose nanocrystals were used only as chemically prepared material components and were not treated as plant specimens.
Novel plant genotypes	No novel plant genotypes, transgenic plants, gene-edited plants, mutant lines or hybrid plant lines were generated or used.
Authentication	No seed stocks or plant genotypes were used. Therefore, authentication of plant materials or genotypes was not required.
