# Supplementary material for: Collaborative passive cooling of impact-hardening interfaces enabled by nacre-mimetic design
Source: Nat Commun. 2026 Jun 22;17:7823. doi: 10.1038/s41467-026-74755-w (PMC13439421; doi:10.1038/s41467-026-74755-w)
Supplement: Supplementary file 3 [file 41467_2026_74755_MOESM3_ESM.pdf]

nature portfolio

Peer Review File

Collaborative passive cooling of impact-hardening interfaces enabled by nacre-mimetic design

Corresponding Author: Professor Xinglong Gong

This file contains all reviewer reports in order by version, followed by all author rebuttals in order by version.

Version 0:

Reviewer comments:

Reviewer #1

(Remarks to the Author)
This manuscript presents a highly innovative and meticulously researched study on the development of a nacre-mimetic composite (STBC and its sandwich derivative, s-STBC) that synergistically integrates passive radiative cooling with exceptional impact resistance. The work is ambitious in its scope, addressing a critical challenge in outdoor device protection. The data presented is extensive and generally supports the authors' claims. The concept of combining these two functionalities is very novel and of significant potential interest to the fields of materials science, thermal management, and protective structures. However, the manuscript in its current form requires mandatory revisions to meet the high standards of Nature Communications after addressing the following comments in a convincing way.

1. The manuscript does a reasonable job of comparing its thermal anisotropy and radiative cooling performance to other advanced materials in Supplementary Tables 1 and 2. However, the comparison for impact resistance is inadequate. Please provide a direct, quantitative comparison of impact resistance metrics (specific puncture energy, maximum force) with state-of-the-art materials from the literature in the main text or a supplementary figure.
2. The high-speed impact results are impressive but the direct evidence linking the performance to the "dynamic crosslinking" within the SSG mortar is unclear. A quantitative analysis, such as comparing the storage modulus (G') of the pure SSG and the STBC composite at high strain rates (e.g., using the SHPB data or dynamic mechanical analysis at high frequencies), would strengthen this causal link.
3. The claim that the s-STBC design enhances cooling by creating thermal isolation is interesting. However, the temperature difference between STBC and s-STBC (Fig. 5d,e) should be more rigorously explained. Is the ~30°C difference primarily due to the insulating SSG layer reducing heat flow from the heater, or is there a genuine enhancement in the radiative cooling efficiency of the top STBC layer? A control experiment with a passive insulator of similar thickness instead of SSG would help decouple these effects and prove true synergy.
4. The LCA (Fig. 5l) is a valuable result but the methodology description in Supplementary Discussion 4 is insufficient. The authors shall provide more details in the main text or methods: What are the system boundaries? How were the energy inputs for ball milling, vacuum filtration, and high-temperature curing accounted for? A brief summary of key assumptions is necessary for the reader to assess the validity of the environmental claims.
5. Figure 4i and Supplementary Table 5 show that recycled STBC (r-STBC) retains >95% of original properties. This is excellent but the recycling process involves ball-milling the TBC scaffold for 48 hours. The authors should comment on whether this intense mechanical process causes degradation of the BNNS or CNC, and if not, why the structure remains intact. An SEM image of the r-STBC showing the preserved "brick-and-mortar" morphology would be very convincing.

Reviewer #2

(Remarks to the Author)
Overall Assessment and Recommendation
Recommendation: Major Revision
This manuscript reports a nacre-inspired multifunctional composite (BNNS/CNC scaffold with a shear-stiffening gel matrix) that combines high thermal conductivity, radiative-cooling capability, and impact protection. The integration is creative, and the experimental work is comprehensive. The study presents practical demonstrations such as energy-saving simulations, battery-protection tests, and recyclability analysis.
While the concept of combining heat-spreading, radiative-cooling, and mechanical protection in a single material is

appealing, the scientific novelty and mechanistic depth currently fall short of the level expected for Nature Communications. Most observed performances arise from architectural integration of known components rather than discovery of a new physical mechanism or generalizable design principle. Nevertheless, the data quality, sustainability focus, and multifunctionality merit serious consideration after significant revision.
Here are the detailed comments below:
Major Comments
1. Novelty and Conceptual Depth
- The integration of nacre-like alignment, radiative-cooling design, and shear-stiffening gel (SSG) is interesting but not conceptually groundbreaking. Each subcomponent has rich precedent. The manuscript should clearly state what fundamental insight or transferable principle emerges from their integration.
- A quantitative or predictive model correlating microstructure (alignment, porosity, or layer aspect ratio) to multifunctional performance ($\kappa_{\parallel}$, $\varepsilon_{8\text{–}13}$ μm, and rate-dependent modulus) would greatly enhance scientific value.
- Many studies have already used BN-based fillers to achieve high thermal conductivity; here the performance appears largely due to high BN loading, not network efficiency. Please clarify whether the composite achieves enhanced conduction via percolation pathways or simply filler crowding.
- The rationale for using boronic acid (BA) as the dynamic crosslinking unit in the SSG should also be strengthened. Other chemistries (e.g., disulfide, boronic ester variants) have shown excellent performance. Explain why BA chemistry is chosen and what unique advantages it confers.
2. Thermal Characterization and Benchmarking
- The reported anisotropic thermal conductivity (8.33 $W \cdot m^{-1} \cdot K^{-1}$, anisotropy ≈ 49) is impressive, but TPS/Hot Disk measurements may overestimate in-plane thermal conductivity for layered anisotropic structures. Validation using laser-flash or 3ω techniques is recommended.
- Include thermal resistance (°C W^{-1}) in a realistic device stack to show applicability for heat-spreading or cooling devices.
- Specify the BNNS content (wt.% or vol.%) in all tested samples and ensure comparisons of anisotropy are made at similar filler loadings. Clarify whether the nacre-like alignment remains stable across varying BNNS contents.
- Compare radiative-cooling performance under standard outdoor sub-ambient tests (midday, clear-sky, angular dependence) with leading RC coatings or composites.
3. Mechanical and Impact Response
- The impact and drop tests are persuasive visually, but remain qualitative. Provide standardized metrics such as transmitted-force, absorbed-energy, or resilience following ISO/ASTM/NIJ standards.
- Include rate-dependent modulus data (DMA or SHPB) to distinguish contributions from the SSG's shear-stiffening versus the nacre-like architecture.
- Clarify long-term SSG stability: Fig. 1d shows strain-rate sweeps at multiple intervals (1–24 months), but the text lacks explanation. Please state that periodic tests confirm temporal stability of the dynamic B–O network, showing negligible modulus change and consistent stiffening behavior.
4. Durability, Flammability, and Recyclability
- For Fig. 2h, include quantitative flammability metrics (UL-94, LOI, or cone calorimetry: PHRR/THR/TTI) to benchmark fire resistance.
- The statement that STBC remains intact after 1 h at 600 °C should be supported by post-flame performance tests (thermal conductivity, reflectance, mechanical strength) to verify functional retention beyond visual appearance.
- The recyclability claim (Fig. 4h) suggests the SSG dissolves in ethanol, implying borosiloxane matrix lability to polar solvents. Clarify whether this recyclability derives from intentional dynamic bond reversibility or solvent-induced degradation. Provide quantitative stability data in polar and humid environments.
- The reported "3 h water stability" is short for outdoor applications; include mass retention, swelling ratio, and mechanical retention after longer immersion (≥24 h) and cyclic wet-dry testing.
- For Supplementary Figs. 11–13, add data for repeated immersion–drying cycles and quantitative metrics (weight change, dimensional change, mechanical strength variation).
5. Durability and Environmental Stability
- Demonstrate resistance to UV, humidity, and temperature cycling, as these are critical for outdoor applications.
- Quantify adhesion to representative substrates (metal, polymer, ceramic) before and after environmental exposure.
6. Life-Cycle Assessment (LCA) and Scalability
- The current functional unit ("1 m^{-3} (W m^{-1} $K^{-1})^{-1}$") is unnecessarily complex. Recalculate or at least normalize results using practical units (e.g., "1 m^2 of coating" or "1 kg of material") for clarity.
- Expand the LCA to report carbon and water footprints of key raw materials and summarize in a supplementary table.
- If feasible, include a service-life estimate (accelerated aging or FMEA) to strengthen the durability argument.
- For industrial feasibility, discuss scalability of the fabrication method (e.g., sheet size, yield, energy consumption). A simple production-scale simulation (~1 ton yr^{-1}) and associated cost or energy estimates would add practical relevance.
Minor Comments:
• Define the atmospheric transparency window (8–13 μm) and emphasize why emissivity in this range is crucial for passive radiative cooling.
• Clarify how optical spectra were measured at 120 °C (instrumentation, calibration).
• Add uncertainty bars for all thermal and mechanical plots.
• Streamline redundant figure captions in the Supplementary Information.
• Correct typographical errors (e.g., "brick-and-motar" → "brick-and-mortar").
• Briefly explain in Fig. 3j why equatorial regions show stronger simulated cooling; consider adding rainfall/dust-condition simulations to support robustness claims.

Reviewer #3

(Remarks to the Author)
Some revised suggestion for this manuscript as following.
1. Information of the same category (temperature quantification metrics) should theoretically adhere to a unified format standard.
2. The enlarged image on the right side of Figure 1c has lower clarity compared to the surrounding images. Please optimize it.
3、Figure 1a contains several points that may be subject to some debate. Please be advised.
(1) Can arrows be drawn in the section from the “heat source” to the “conductive” interface to indicate heat transfer from the heat source to the transfer layer?
(2) The model on the right is not closely connected to the actual environment shown on the left. Could a more intuitive connection method be used, such as a magnifying glass focused on the roof?
(3) This regulation reflects my personal perspective and represents what I consider the most contentious aspect: The right diagram in Fig. 1a visually depicts solar radiation reflection and thermal conduction on the uppermost surface. However, the schematic in the right diagram of Fig. 5a illustrates the material's top-layer reflection, intermediate thermal insulation, and lateral thermal conduction properties. Regarding the “heat radiation surface” section, I personally believe the author's visual continuity in the diagrams is incomplete. In fact, a thorough reading reveals the author intends to convey that their material can reflect sunlight and radiate heat from any surface. However, viewing the images in isolation may confuse readers about which specific surface constitutes the “heat radiation surface.”
4. Regarding the s-STBC structural design: The thickness of the SSG layer directly impacts the balance between thermal insulation and impact resistance. Could you provide “comparative experimental data on the maximum cooling temperature difference and residual impact force of s-STBC under different SSG thicknesses (e.g., 1mm, 3mm, 5mm)”? Please also explain the core considerations for the current thickness selection. Neither the main text nor the references section of this article addresses research on SSG layer thickness.
5. The English need to improve if possible.

Reviewer #4

(Remarks to the Author)
The manuscript by Li et al. designed and fabricated a nacre-inspired composites with passive cooling and impact resistance properties. There is no doubt that the nacre-mimetic materials could exhibit excellent mechanical properties. The anisotropy of the materials is also logic due to the unique structure of nacre-like structure. What make the materials with excellent overall properties compared with other nacre-like materials could be the unique composition. I think this is one of the main contribution and novelty of the work. Overall, the manuscript is well-written with a lot of data, but the design principles and synthesis/fabrication part should be enhanced with more control experiments. I would suggest major revision of manuscripts and the following concerns should be addressed in the revised manuscript.

1. The authors showed that they can fabricate the composites with nacre-mimetic structure, but the influence of different parameters on the structure and properties have not been investigated. For example, the authors claimed that “Cellulose nanocrystals (CNCs), prepared through the hydrolysis of cellulose, were incorporated to enhance the overall thermo-mechanical performance.”, however, they did not provide any evidence. The authors should vary the amount of CNCs and TA, the content of BNNS and SSG polymer and see how would the structure and properties (both thermal behavior and mechanical properties) change with them.
2. What is the role of ethyl alcohol? What does dissolution/immersion/drying mean in the Figure 1e. The authors should clear explain the procedures since it is one of the main figure in the article and tell the readers the design principles.
3. Furthermore, there are numerous similar procedures to fabricate nacre-mimetic composites materials. What makes this one unique compared with those reported in literature? For example, Nat. Commun. 2017, 8 (1), 287. https://doi.org/10.1038/s41467-017-00392-z.
4. Figure 2d-e, heat flux mappings at 0.01 ms. What is the X and Y axis of the mapping? And what does the color and arrow mean in the figure? Is there any scale in this figure? Furthermore, the detailed measurement methods should be provided.
5. Figure 3, the authors only showed the properties of one sample. Again, is this reproducible? What is the influence of different parameters on these properties?
6. In the design strategy, Figure 1b, the authors compared the thermal conduction of amorphous materials and crystalline materials. How does this mechanism contribute to the design of the composites? Could the author provide examples showing similar composites made of amorphous materials that exhibited lower thermal properties?
7. In the design strategy, Figure 1d, the authors demonstrate the possible intelligent adjustable stiffness. How would this contribute to the overall function of the materials? It would be good if the authors could demonstrate the “intelligent” function of the final composites.
8. It would be good if the authors could further elaborate on the future direction to improve the properties of these kind of materials. For example, by changing the composition of brick and mortar to which materials could potentially be better?

Version 1:

Reviewer comments:

Reviewer #1

(Remarks to the Author)
The authors carefully revised and improved the manuscript. I recommend acceptance.

Reviewer #2

(Remarks to the Author)
The authors have comprehensively addressed the major concerns raised in the previous review. In particular, the revised manuscript now provides (i) strengthened conceptual framing of the dynamic-response, multi-physics design principle, (ii) quantitative microstructure–property correlations supported by parametric modeling, (iii) independent validation of thermal conductivity via laser flash analysis, (iv) standardized impact metrics in accordance with ISO protocols, (v) detailed durability assessments including water, UV, and environmental cycling tests, and (vi) expanded life-cycle analysis with clarified system boundaries and practical functional units.

The additional experimental controls, benchmarking against state-of-the-art materials, and improved mechanistic explanations significantly enhance the scientific rigor and clarity of the work. The multifunctional integration is now convincingly demonstrated rather than descriptively presented, and the translational relevance is substantially strengthened.

In my opinion, the manuscript now meets the standards of Nature Communications in terms of novelty, depth, and technical completeness. I recommend acceptance.

Reviewer #4

(Remarks to the Author)
The authors have addressed all my concerns and I suggested acceptance of the manuscript.

Reviewer #5

(Remarks to the Author)
This manuscript presents a highly innovative and meticulously researched study on the development of a nacre-mimetic composite (STBC and its sandwich derivative, s-STBC) that synergistically integrates passive radiative cooling with kinetic impact defense. The integration of a dynamic chemical network with an ordered biomimetic architecture successfully overcomes the performance trade-offs typical of conventional composites. The authors have done an excellent job addressing the previous rounds of peer review, making the paper absolutely worth accepting. A decision of Minor Revision is recommended to allow the authors to finalize the formatting of their newly added figures and standardize the nomenclature before publication.

1. The revised manuscript and the accompanying response letter clearly articulate the fundamental principle of "dynamic-response and interface-mediated multi-physics synergistic regulation". By coupling a functional boron nitride nanosheet/cellulose nanocrystal (BNNS/CNC) skeleton with an environmentally responsive shear-stiffening gel (SSG) matrix, the authors have established a transferable design paradigm for polymer engineering that moves far beyond simple performance stacking.

2. The authors comprehensively addressed concerns regarding the causal link between dynamic crosslinking and impact resistance. The inclusion of Split Hopkinson Pressure Bar (SHPB) tests at a high strain rate of 2000 s^-1 and dynamic mechanical analysis (DMA) provides definitive quantitative evidence of the impact-hardening effect. The updated comparisons confirm that STBC significantly outperforms numerous state-of-the-art engineering materials in both maximum force and specific puncture energy.

3. The revisions highlighted in blue demonstrate exceptional methodological rigor. Validating the initial thermal conductivity measurements with laser-flash analysis (yielding 7.56 W m-1 K^-1 and confirming a thermal anisotropy ratio of 44.47 solidifies the claims regarding directional heat dissipation. Furthermore, the decoupling of passive thermal insulation from active synergistic cooling using Ecoflex and EPE foam control experiments elegantly proves the true synergy of the s-STBC design.

4. The extensive life-cycle assessment (LCA) boundary clarifications , alongside the newly added quantitative flammability metrics (e.g., an LOI of 69%), significantly elevate the practical relevance of this work. The outdoor cyclic aging tests encompassing UV, humidity, and temperature variations confirm the material's viability for protecting outdoor devices in extreme, prolonged environmental conditions.

5. While the scientific merit is now exceptionally solid, the authors should ensure that all newly replaced high-resolution SEM images and the standardized SI temperature metrics (Kelvin) are perfectly formatted and free of any residual typographical errors in the final galley proofs.

6. The authors should consider adding references related to passive radiation cooling materials to provide a broader context for their work. Some relevant references include:

https://doi.org/10.1016/j.energy.2025.136840
https://doi.org/10.1016/j.jobe.2025.113382

https://doi.org/10.1002/adsu.202500734
https://doi.org/10.1016/j.nanoen.2020.105600
https://doi.org/10.1021/acsami.5c14931

Reviewer #6

(Remarks to the Author)
The author has very well addressed all the questions raised by reviewer #3. Regarding the radiation cooling section, the current revised manuscript is basically free from any issues. Besides, it is to refer to the recent articles published in Nature Communications related to radiation cooling, such as Nature Communications, 2025, 16, 10533. Nature Communications, 2024, 15, 3076.

Open Access This Peer Review File is licensed under a Creative Commons Attribution 4.0 International License, which permits use, sharing, adaptation, distribution and reproduction in any medium or format, as long as you give appropriate credit to the original author(s) and the source, provide a link to the Creative Commons license, and indicate if changes were made.
In cases where reviewers are anonymous, credit should be given to 'Anonymous Referee' and the source.
The images or other third party material in this Peer Review File are included in the article's Creative Commons license, unless indicated otherwise in a credit line to the material. If material is not included in the article's Creative Commons license and your intended use is not permitted by statutory regulation or exceeds the permitted use, you will need to obtain permission directly from the copyright holder.
To view a copy of this license, visit https://creativecommons.org/licenses/by/4.0/

Reply to Reviewer #1

Comment: This manuscript presents a highly innovative and meticulously researched study on the development of a nacre-mimetic composite (STBC and its sandwich derivative, s-STBC) that synergistically integrates passive radiative cooling with exceptional impact resistance. The work is ambitious in its scope, addressing a critical challenge in outdoor device protection. The data presented is extensive and generally supports the authors' claims. The concept of combining these two functionalities is very novel and of significant potential interest to the fields of materials science, thermal management, and protective structures. However, the manuscript in its current form requires mandatory revisions to meet the high standards of Nature Communications after addressing the following comments in a convincing way.

Our reply: Thank you for your positive assessment and thoughtful questions regarding this work. Your insights have provided crucial guidance for enhancing the scientific rigor and precision of the manuscript. In response to the core issues you raised, we have undertaken systematic revisions and enhancements. All modifications are highlighted in blue and have been integrated into both the manuscript and supplementary information. Through additional experiments, strengthened mechanistic analysis, and standardized data presentation, we have substantially improved the manuscript to meet the rigorous standards of *Nature Communications*. We appreciate your meticulous review, as these revisions have significantly strengthened the work's credibility and practical relevance.

Question 1: The manuscript does a reasonable job of comparing its thermal anisotropy and radiative cooling performance to other advanced materials in Supplementary Tables 1 and 2. However, the comparison for impact resistance is inadequate. Please provide a direct, quantitative comparison of impact resistance metrics (specific puncture energy, maximum force) with state-of-the-art materials from the literature in the main text or a supplementary figure.

Our reply: We appreciate your insightful suggestion. To address the concern of

insufficient impact resistance benchmarking, we have supplemented a direct and quantitative comparison of key impact metrics (maximum force and specific puncture energy) between STBC and state-of-the-art materials reported in the literature (Supplementary Table 4 and Fig. 4f). STBC exhibits superior impact resistance, outperforming state-of-the-art and high-performance engineering materials in both maximum force and specific puncture energy.

Supplementary Table 4 | Impact resistance of STBC compared with state-of-the-art and high-performance engineering materials.

Specimen	Maximum force [kN m^{-1}]	Specific puncture energy [J m^{-1}]	Reference
**STBC**	**61.10**	**107.64**	**This work**
PVA/GO (PG1)	58.25	/	R1
ANF	25.43	98.28	R2
PPGTD-IPDA (PI1)	37.25	/	R3
MNH	11.7	10.8	R4
Si_3N_4	1.84	/	R5
NH	2.3	0.55	R6
PDO-IP2.5 (PI2)	≈56	/	R7
DNH	0.8	0.53	R8
HPDU10 (H10)	32.13	/	R9
Hex[5A] (H5A)	31.25	37.5	R10
Leather/SSG/Kevlar/STF/NWF (LSKSN)	32	/	R11
PL-Gel (PG2)	28.95	2.6	R12
AA/HEMA/PEG (AHP)	22.1	/	R13
EVA	7.51	100.30	The data are derived from this work.
SSE	10.45	93.81	
CB	20.40	89.12	
Wood	56.61	75.12	

Specimen	Maximum force [kN m^{-1}]	Specific puncture energy [J m^{-1}]	Reference
EPE	4.37	34.56	
PU	0.76	8.02	
PVC/NBR (PN)	0.86	4.74	

Both maximum force and specific puncture energy are normalized by specimen thickness. PVA: Poly(vinyl alcohol); GO: Graphene oxide; ANF: Aramid nanofiber; PPGTD: PPG, tolylene 2,4-diisocyanate terminated; IPDA: Isophoronediamine; MNH: Microsphere-reinforced nanocomposite hydrogel; NH: Nanocomposite hydrogel; PDO: 2, 4-pentanedione dioxime; DNH: Double network hydrogel; HPDU: Hyperbranched polyurethane; SSG: Shear-stiffening gel; STF: Shear-thickening fluid; NWF: Non-woven fabric; AA: Acrylic acid; HEMA: Hydroxyethyl methacrylate; PEG: Polyethylene glycol; EVA: Ethylene vinyl acetate copolymer; SSE: Shear-stiffening elastomer; CB: Corrugated board; EPE: Expanded polyethylene; PU: Polyurethane; PVC: Polyvinyl chloride; NBR: Nitrile butadiene rubber. SSE is formulated following work[R14], and the engineering specimens used for comparison are commercially available products.

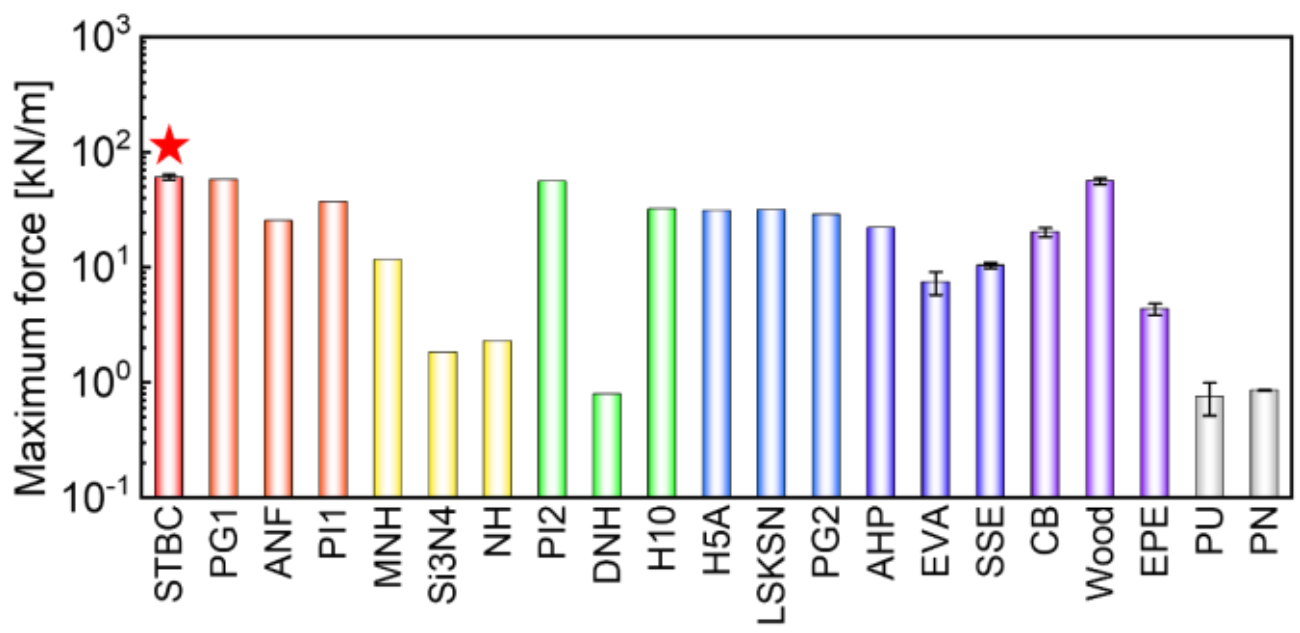

Fig. 4f | Maximum force of STBC compared with various state-of-the-art and high-performance engineering materials in instrumented impact tests.

The corresponding discussion has been incorporated into the revised manuscript and supplementary information. (Page 15, line 319; Page 16 lines 320-321; Page 17, lines 368-370; Page S24, lines 293-305)

Question 2: The high-speed impact results are impressive but the direct evidence linking the performance to the "dynamic crosslinking" within the SSG mortar is unclear.

A quantitative analysis, such as comparing the storage modulus (G') of the pure SSG and the STBC composite at high strain rates (e.g., using the SHPB data or dynamic mechanical analysis at high frequencies), would strengthen this causal link.

Our reply: Thank you for suggesting that our argument would benefit from additional quantitative evidence to more directly link dynamic crosslinking with impact resistance. High-speed impact tests show that incorporating SSG markedly improves the overall impact resistance of the composites, which is attributed to dynamic crosslinking (Fig. 4e). However, quantitative analysis is needed to substantiate this causal link.

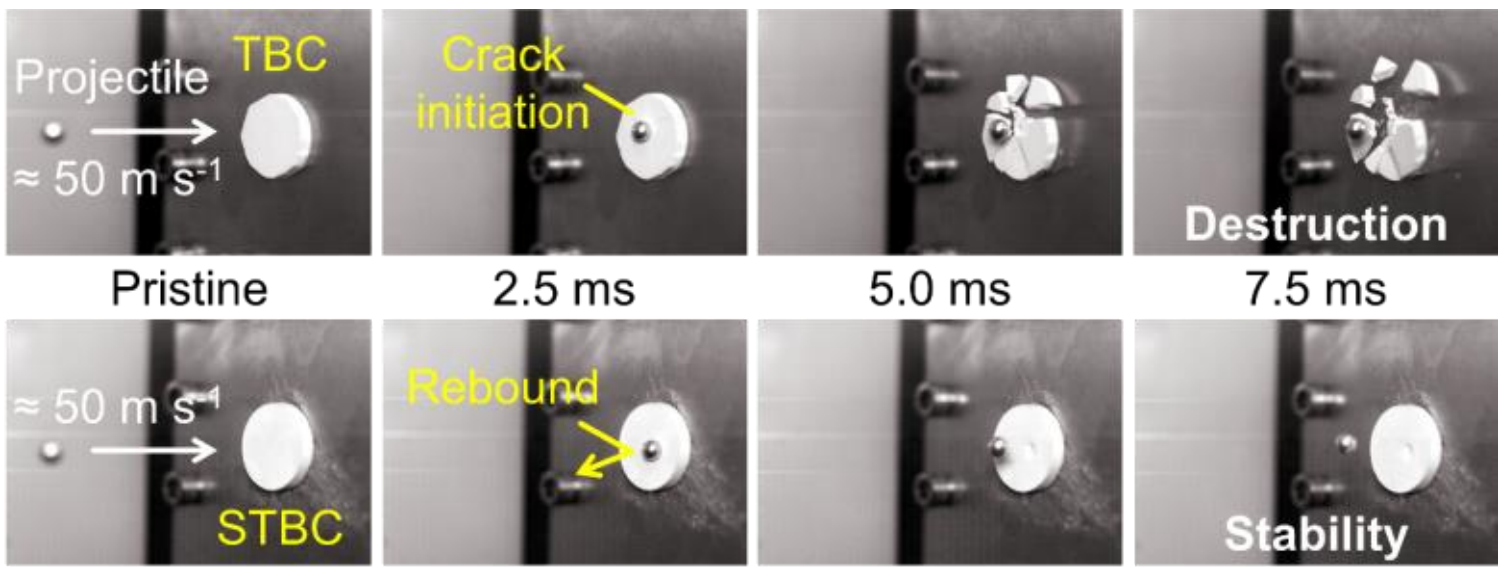

Fig. 4e | High-speed projectile impact processes of TBC and STBC.

Following your recommendation, we have included SHPB data at a strain rate of 2000 s^{-1} (Supplementary Fig. 22b) and dynamic mechanical analysis (DMA) at frequencies from 25 to 50 Hz (Supplementary Fig. 22c) for pure SSG mortar, TBC skeleton, and STBC composite to simulate high-speed impact loading conditions. The dynamic compressive strength and storage modulus of STBC are both higher than those of the individual subcomponents (SSG mortar and TBC skeleton). These results indicate that incorporating SSG to form STBC substantially enhances mechanical performance compared with TBC, conferring greater rigidity and impact resistance under high-rate loading[R15,R16].

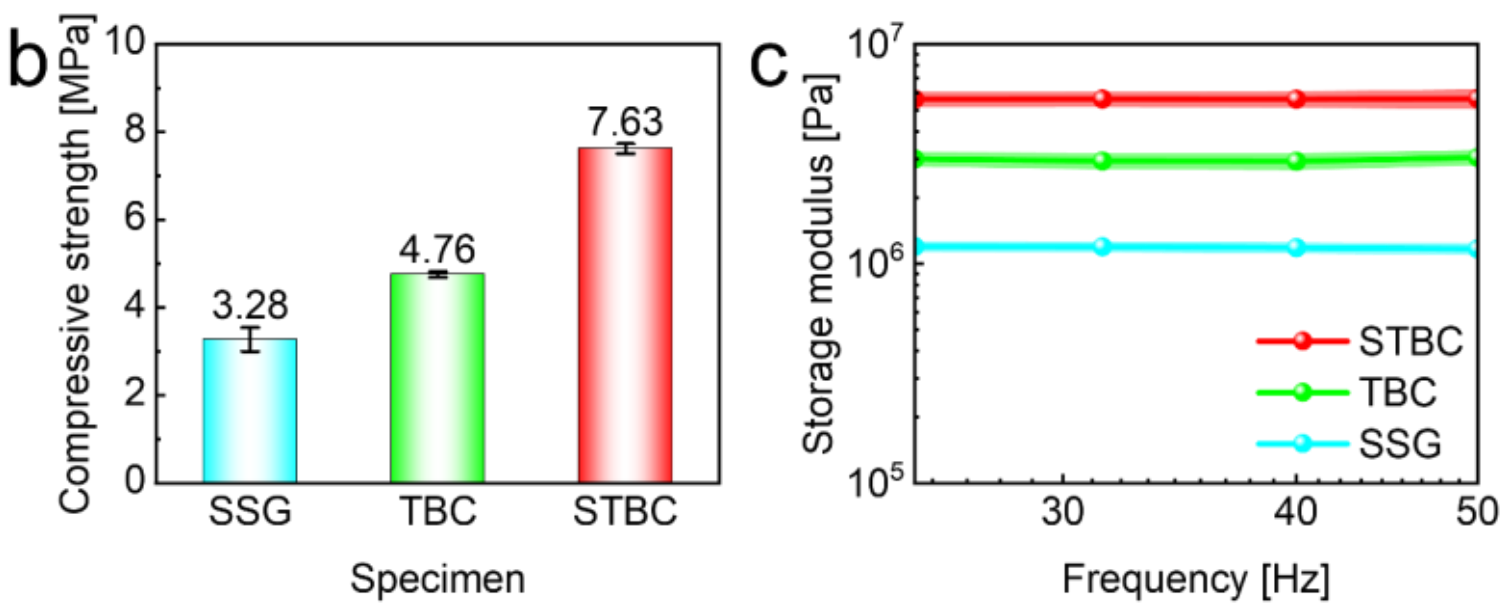

Supplementary Fig. 22 | b Compressive strength of SSG, TBC, and STBC at 2000 s^{-1}

from SHPB tests. **c** Storage modulus of SSG, TBC, and STBC from DMA.

The corresponding experiments and discussion have been incorporated into the revised manuscript and supplementary information. (Page 15, line 319; Page 17, lines 354-363; Page 26, lines 599-600; Page 27, lines 603-604; Page S14, lines 206-213)

Question 3: The claim that the s-STBC design enhances cooling by creating thermal isolation is interesting. However, the temperature difference between STBC and s-STBC (Fig. 5d,e) should be more rigorously explained. Is the ~30°C difference primarily due to the insulating SSG layer reducing heat flow from the heater, or is there a genuine enhancement in the radiative cooling efficiency of the top STBC layer? A control experiment with a passive insulator of similar thickness instead of SSG would help decouple these effects and prove true synergy.

Our reply: Thank you for this insightful perspective. It motivates us to rigorously differentiate passive thermal insulation from active synergistic cooling through controlled comparative experiments. We select commercial Ecoflex with thermal conductivity comparable to that of SSG (0.19 W/mK), and thermally insulating EPE foam as passive insulators with matched thickness to replace SSG, yielding STBC-Ecoflex-STBC and STBC-EPE-STBC specimens, respectively. Under identical irradiance, the steady-state temperatures of STBC, STBC-Ecoflex-STBC, STBC-EPE-STBC, and s-STBC are 379.1, 370.7, 360.4, and 350.7 K, respectively (Fig. 5e).

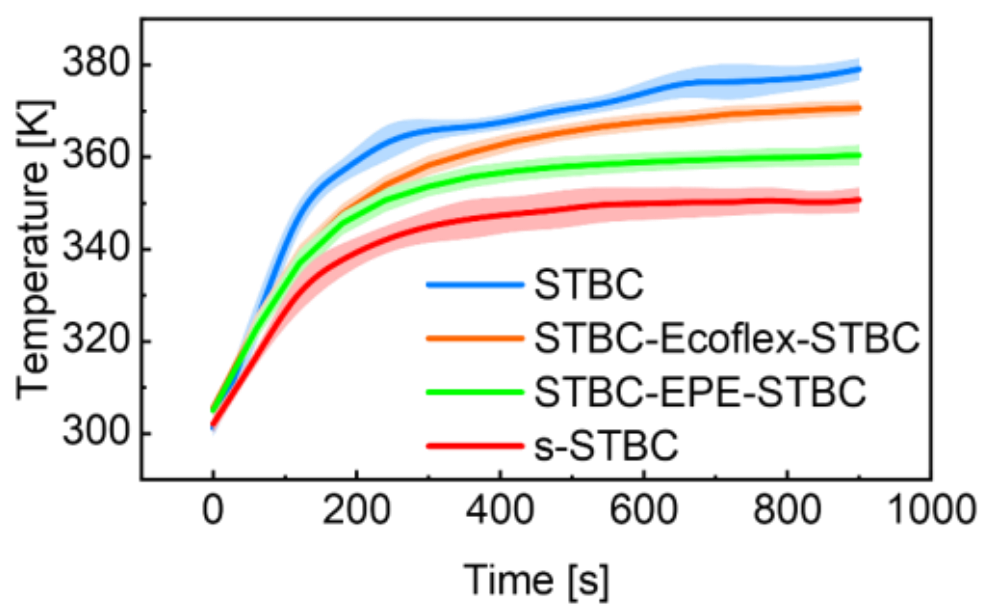

Fig. 5e | Temporal temperature profiles of heater-integrated passive insulators under 1 sun irradiation.

(1) **Passive thermal insulation:** Interfacial thermal resistance between dissimilar materials slows heat transfer compared with monolithic STBC, confirming that passive insulators can lower STBC temperature by limiting heat input.

(2) **Active synergistic cooling:** The specific heat and emissivity of SSG are 1.54 MJ m^{-3} K^{-1} and 0.94, respectively, both exceeding those of Ecoflex, which are 1.35 MJ MJ m^{-3} K^{-1} and 0.85. Materials with higher specific heat require greater heat input to achieve the same temperature rise, thereby delaying the system's thermal response. Moreover, higher emissivity enhances radiative heat loss and establishes an effective radiative heat sink. The s-STBC architecture simultaneously suppresses heat conduction and promotes thermal dissipation to the surroundings, producing a synergistic inhibition of heat flow. In addition, the thermal insulation performance of SSG as an interlayer surpasses that of commercial foam, further validating the advantages of the s-STBC design. The superiority of s-STBC exceeds that attainable with a simple "insulation layer & STBC" configuration, demonstrating a genuine synergy.

In summary, the SSG layer blocks heat transfer and enables the STBC layers on both sides to deliver enhanced cooling performance, achieving both passive thermal insulation and active synergistic cooling (Fig. 5a).

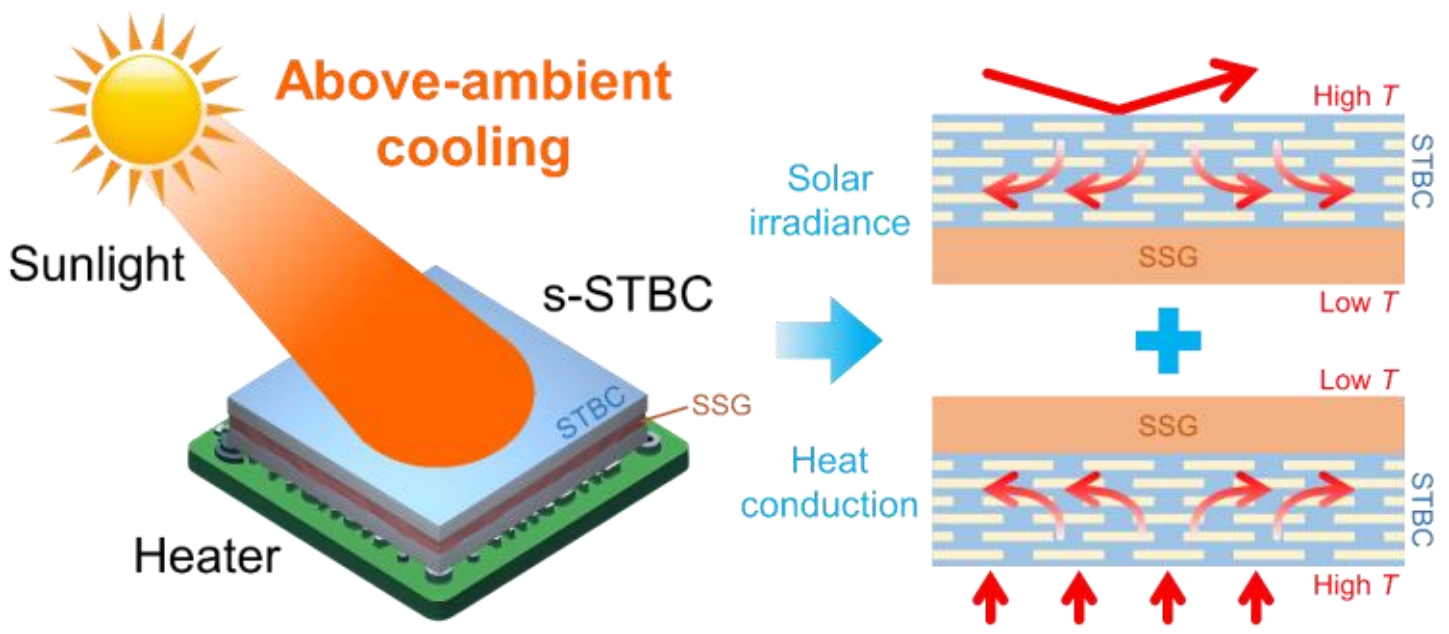

Fig. 5a | Above-ambient passive cooling mechanism of s-STBC.

Relevant figures and discussion have been added in the revision. (Page 19, lines 395, 397-398; Page 21, lines 443-445; Page S4, lines 74-90; Page S5, lines 91-92)

Question 4: The LCA (Fig. 5l) is a valuable result but the methodology description in

Supplementary Discussion 4 is insufficient. The authors shall provide more details in the main text or methods: What are the system boundaries? How were the energy inputs for ball milling, vacuum filtration, and high-temperature curing accounted for? A brief summary of key assumptions is necessary for the reader to assess the validity of the environmental claims.

Our reply: Thank you for your professional advice. Regarding the LCA system boundary, we have prepared a detailed schematic that clearly delineates the methodological scope and enables intuitive understanding of the assessment coverage (Supplementary Fig. 35). Energy inputs for ball milling, vacuum filtration, and high-temperature curing are calculated based on the rated power of the equipment used and their recorded operating durations. Detailed data have been provided in Supplementary Tables 7 and 8 to ensure accurate energy consumption estimates. We also include a concise summary of key assumptions in the supplementary information. The summary encompasses the criteria for data source selection and the simplification principles applied during the calculation, providing readers with a sound basis for evaluating the validity of the environmental claims. (Page 22, lines 474-476; Page S5, lines 100-102; Page S20, lines 268-269; Page S27, lines 316-319; Page S28, lines 321-324)

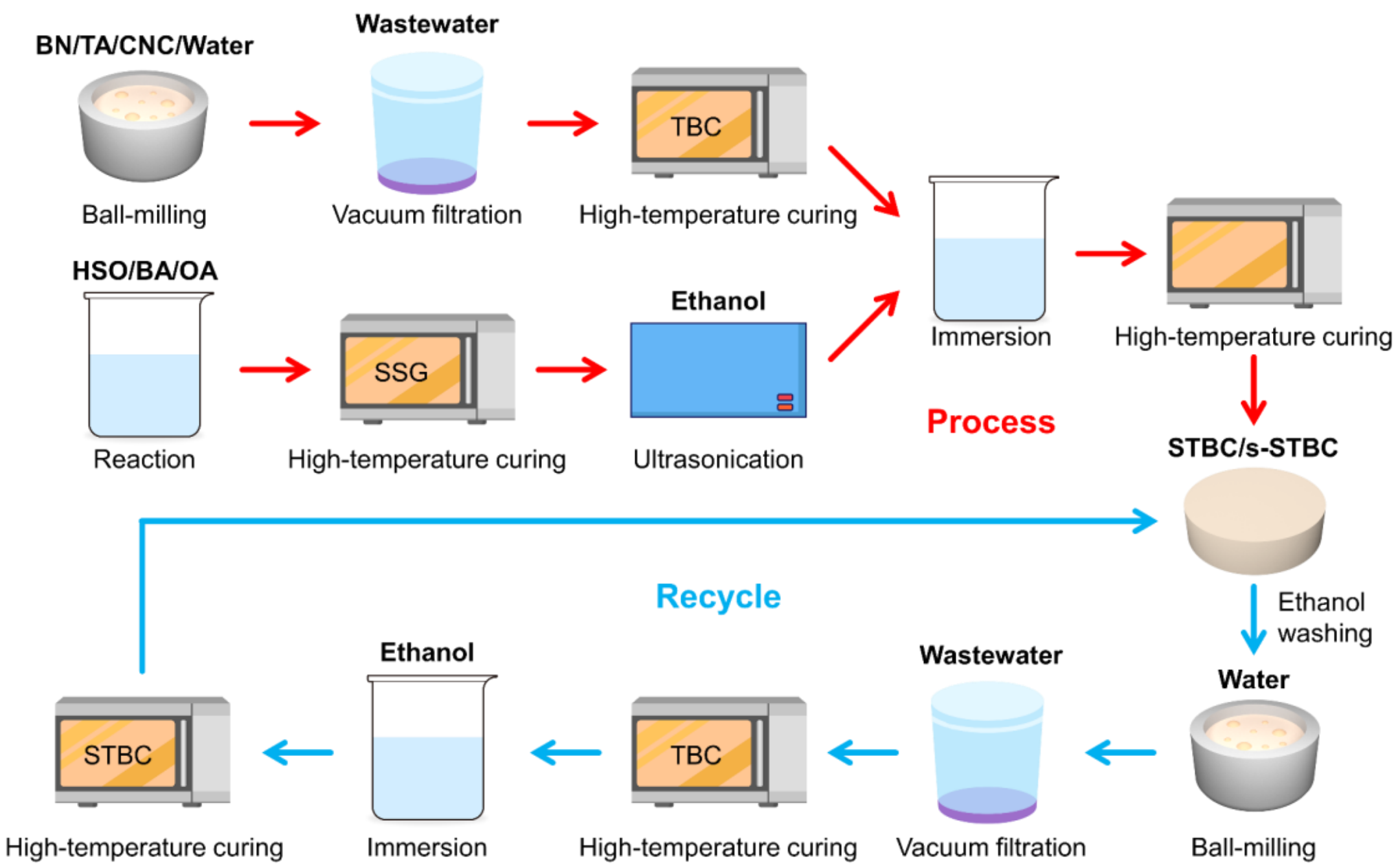

Supplementary Fig. 35 | System boundary of STBC and s-STBC.

Supplementary Table 7 | Life cycle inventory for the production of STBC.

Section	Material	Amount	Unit	Database
Input (Process)	BN	620.16	g	R17
	TA	15.50	g	R18
	CNC	31.01	g	R19
	HSO	321.87	g	
	BA	10.73	g	
	OA	0.73	g	
	Ethanol	333.33	g	
	Water	3720.93	g	
	Electricity (Ball milling)	0.20	kWh	Ecoinvent v3.10
	Electricity (Vacuum filtration)	0.36	kWh	
	Electricity (High-temperature curing)	0.36	kWh	
	Electricity (Ultrasonication)	0.06	kWh	
Output (Process)	STBC	1000.00	g	
	Wastewater	3720.93	g	Ecoinvent v3.10
Input (Recycle)	STBC	1000.00	g	
	Ethanol	2000.00	g	
	Water	3720.93	g	
	Electricity (Ball milling)	0.20	kWh	Ecoinvent v3.10
	Electricity (Vacuum filtration)	0.36	kWh	
	Electricity (High-temperature curing)	0.36	kWh	
Output (Recycle)	STBC	1000	g	
	Wastewater	3720.93	g	Ecoinvent v3.10

Electricity consumption for ball milling, vacuum filtration, and high-temperature curing is estimated from the rated power and operating time of each instrument. Initial ethanol inputs are 333.33 g and 2000.00 g, respectively. Assuming 20% consumption (66.66 g and 100.00 g), the remainder is recovered for reuse.

Supplementary Table 8 | Life cycle inventory for the production of s-STBC.

Section	Material	Amount	Unit	Database
Input (Process)	BN	449.19	g	R17
	TA	11.23	g	R18
	CNC	22.46	g	R19
	HSO	499.34	g	
	BA	16.64	g	
	OA	1.14	g	
	Ethanol	241.44	g	
	Water	2695.16	g	
	Electricity (Ball milling)	0.14	kWh	Ecoinvent v3.10
	Electricity (Vacuum filtration)	0.26	kWh	
	Electricity (High-temperature curing)	0.28	kWh	
	Electricity (Ultrasonication)	0.04	kWh	
Output (Process)	STBC	1000	g	
	Wastewater	2695.16	g	Ecoinvent v3.10
Input (Recycle)	STBC	1000	g	
	Ethanol	1448.65	g	
	Water	2695.16	g	
	Electricity (Ball milling)	0.14	kWh	Ecoinvent v3.10
	Electricity (Vacuum filtration)	0.26	kWh	
	Electricity (High-temperature curing)	0.26	kWh	
Output (Recycle)	STBC	1000	g	
	Wastewater	2695.16	g	Ecoinvent v3.10

Electricity consumption for ball milling, vacuum filtration, and high-temperature curing is estimated from the rated power and operating time of each instrument. Initial ethanol inputs are 241.44 g and 1448.65 g, respectively. Assuming 20% consumption (48.28 g and 289.73 g), the remainder is recovered for reuse.

Question 5: Figure 4i and Supplementary Table 5 show that recycled STBC (r-STBC)

retains >95% of original properties. This is excellent but the recycling process involves ball-milling the TBC scaffold for 48 hours. The authors should comment on whether this intense mechanical process causes degradation of the BNNS or CNC, and if not, why the structure remains intact. An SEM image of the r-STBC showing the preserved "brick-and-mortar" morphology would be very convincing.

Our reply: Thank you for your professional suggestion. Comparative performance analysis of r-STBC and STBC (Fig. 4i) indicates that 48 h ball milling does not degrade the performance of BNNS or CNC. This outcome can be attributed to two primary factors: First, the mild ball-milling conditions (low rotational speed and small media diameter) mitigate high-intensity mechanical damage to the material structure. Second, BNNS possesses a robust covalently bonded crystalline lattice, while CNC is a highly crystalline cellulose nanomaterial characterized by a dense internal hydrogen-bonding network. Both constituents therefore exhibit high mechanical stability, ensuring structural integrity under these milling conditions[R20,R21]. To further substantiate this conclusion, we have provided SEM micrographs of r-STBC (Fig. 4h). Compared with Fig. 1e, these images confirm the preservation of the intact brick-and-mortar hierarchical morphology, providing direct visual evidence of structural stability during recycling and supporting the observation that r-STBC retains over 95% of its original properties.

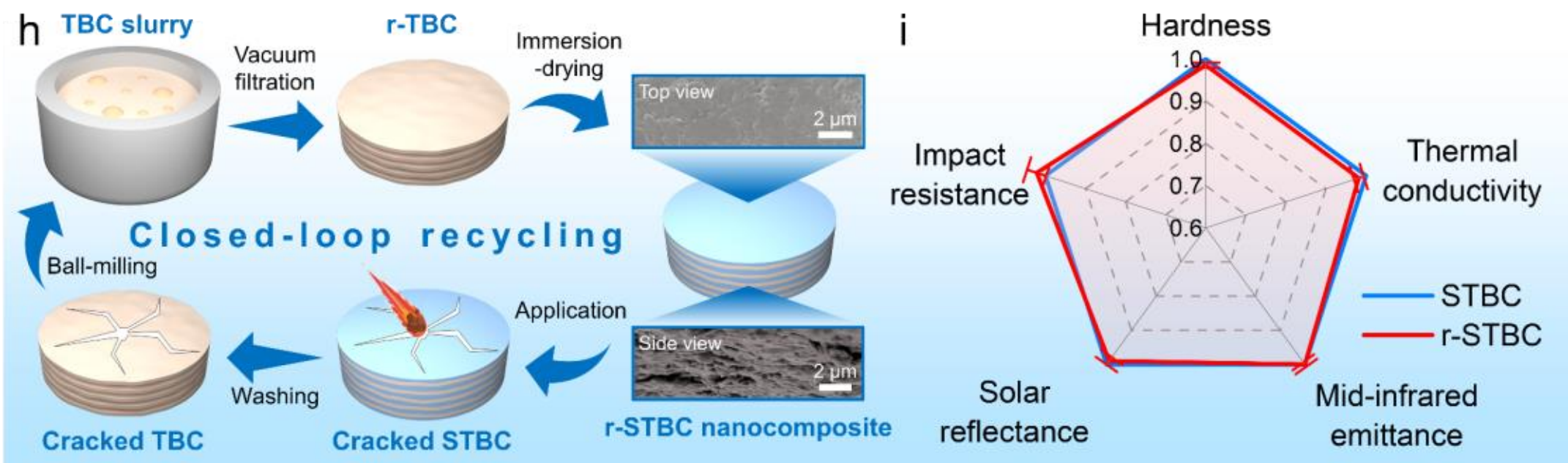

Fig. 4 | h Schematic of the closed-loop recycling process for STBC. **i** Radar plot comparing the multi-functional performance of STBC and r-STBC.

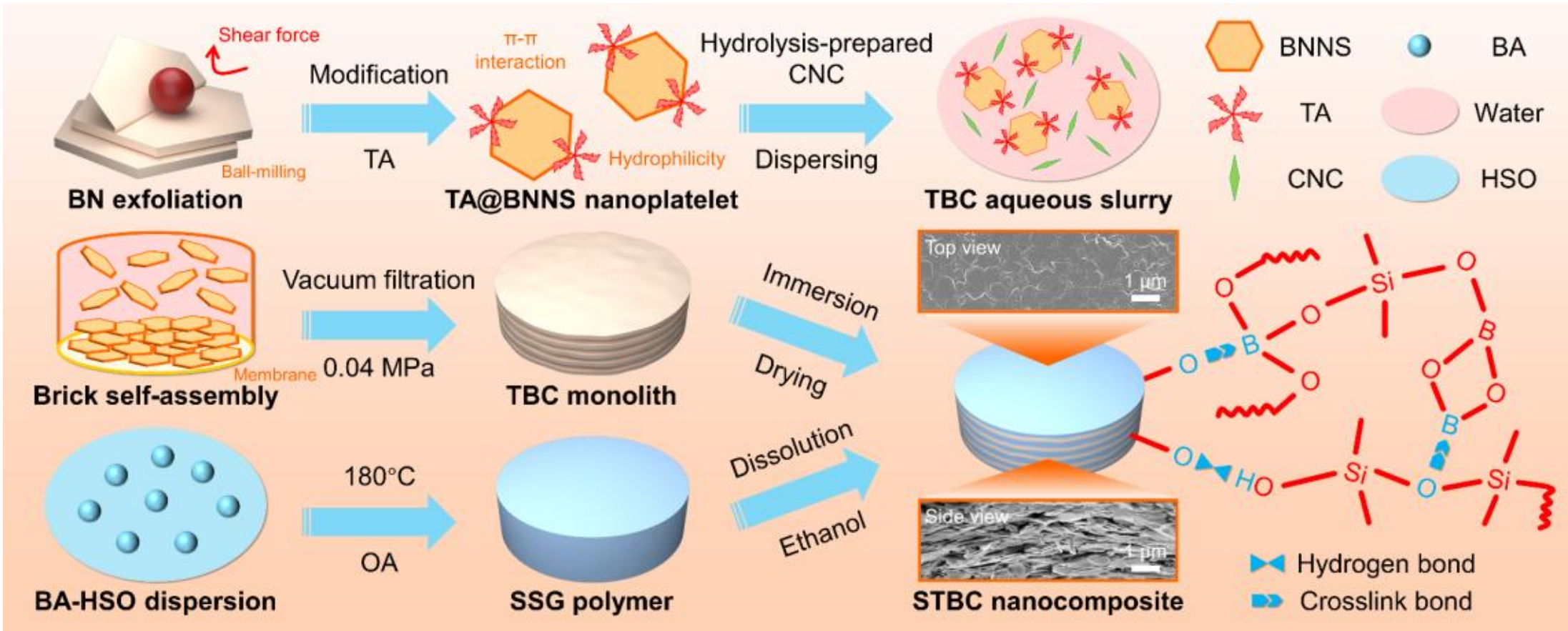

Fig. 1e | Preparation of STBC nanocomposites based on a nacre-mimetic design.

The relevant data have been added to the revised manuscript and supplementary information. (Page 6, line 113; Page 16, lines 322-324)

Reply to Reviewer #2

Comment: Overall Assessment and Recommendation

Recommendation: Major Revision

This manuscript reports a nacre-inspired multifunctional composite (BNNS/CNC scaffold with a shear-stiffening gel matrix) that combines high thermal conductivity, radiative-cooling capability, and impact protection. The integration is creative, and the experimental work is comprehensive. The study presents practical demonstrations such as energy-saving simulations, battery-protection tests, and recyclability analysis.

While the concept of combining heat-spreading, radiative-cooling, and mechanical protection in a single material is appealing, the scientific novelty and mechanistic depth currently fall short of the level expected for Nature Communications. Most observed performances arise from architectural integration of known components rather than discovery of a new physical mechanism or generalizable design principle. Nevertheless, the data quality, sustainability focus, and multifunctionality merit serious consideration after significant revision.

Our reply: Thank you for acknowledging the work's concept of multifunctional integration, experimental rigor, and practical significance, and for providing valuable suggestions to enhance its scientific novelty and mechanistic depth.

The core innovation of this work lies in overcoming the intrinsic limitations of traditional biomimetic materials, which typically prioritize optimization of a single function. We establish a multifunctional synergistic design paradigm that integrates thermal conduction, radiative cooling, and impact resistance. This integration transcends simple performance stacking. By coupling a dynamic chemical network with an ordered nacre-inspired architecture, we enable mutual reinforcement and adaptive synergy among these three core properties under complex and extreme outdoor conditions. The multi-dimensional performance encompassing high strength, anisotropic thermal transport, passive radiative cooling, and kinetic impact protection (Fig. R1), surpasses the performance envelopes of many state-of-the-art and high-performance engineering materials. These advances result from deep innovation

through the co-integration of structure, chemistry, and function.

In the point-by-point response below, we elaborate on the scientific novelty and mechanistic depth of this work and have systematically integrated the relevant content into the revised manuscript to meet the high standards of *Nature Communications*. We appreciate your careful review and constructive guidance, which have substantially enhanced the paper's academic rigor, scientific value, and practical utility.

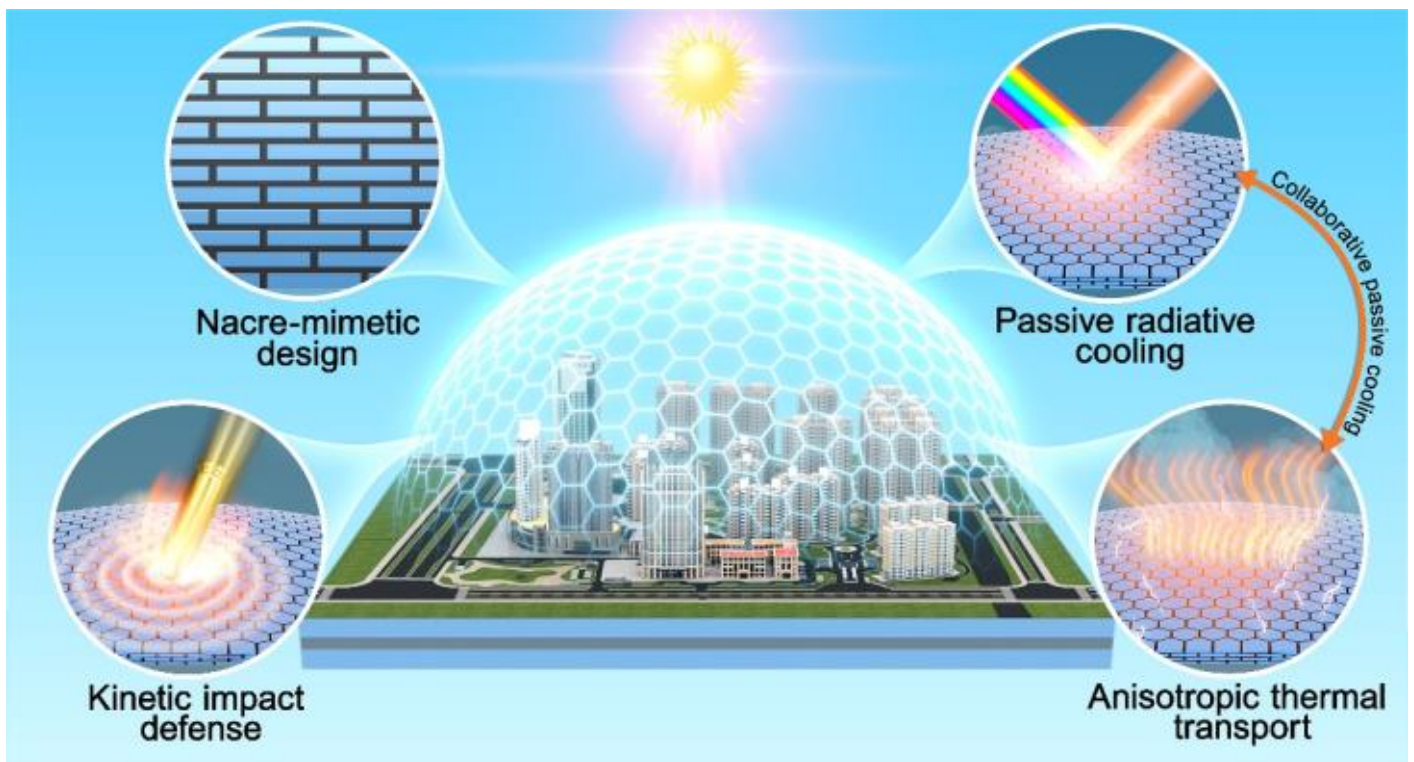

Fig. R1 | Nacre-mimetic STBC exhibiting advanced anisotropic thermal transport, passive radiative cooling, and kinetic impact defense.

Here are the detailed comments below:

Major Comments

1. Novelty and Conceptual Depth

Question 1: The integration of nacre-like alignment, radiative-cooling design, and shear-stiffening gel (SSG) is interesting but not conceptually groundbreaking. Each subcomponent has rich precedent. The manuscript should clearly state what fundamental insight or transferable principle emerges from their integration.

Our reply: Thank you for raising a question that allows us to more clearly articulate the work's core scientific contributions and conceptual advances. The innovation here does not lie in designing a single subcomponent in isolation, but in establishing a transferable fundamental principle of "dynamic-response and interface-mediated multi-physics synergistic regulation" through integrative design. This principle addresses the inherent limitations of traditional composites, which often suffer from functional fragmentation and performance trade-offs.

(1) **Fundamental insight:** Conventional composites are largely static and insensitive to strain rate and thermal response, offering limited functionality and therefore failing to concurrently satisfy the dual requirements of mechanical protection and thermal management. Moreover, their properties are fixed upon fabrication and cannot dynamically respond to environmental changes. Our key advance is the incorporation of a dynamic B-O crosslinking network into a nacre-like structure, thereby constructing an intelligent interface that is activatable by external physical fields. Mechanically, the impact-stiffening behavior of this dynamic interface is precisely triggered under high-strain-rate stress fields. Through reversible bond scission and reformation, it dissipates impact energy beyond the linear superposition expected from its constituents, rather than merely transmitting stress. Thermally, STBC achieves anisotropic thermal conductivity through micro- and nano-scale biomimetic design, thereby satisfying direction-specific thermal requirements. This integrative design delivers synergistic multifunctionality, encompassing high strength, anisotropic thermal transport, passive radiative cooling, and kinetic impact protection, thereby enhancing adaptability in mechanical and thermal performance beyond that of conventional materials.

(2) **Transferable principle:** Building on this fundamental understanding, we propose a bionic integration paradigm of "functional skeleton & dynamically responsive matrix" as a transferable design principle. By selecting ordered and function-oriented skeletons and pairing them with environmental-field-responsive dynamic matrices, one can rationally design intelligent material systems that actively adapt to complex multi-physics environments. This paradigm is not confined to the mechanics-thermodynamics synergy. It can be generalized to scenarios involving extreme temperatures and electromagnetic interference, thereby providing clear and transferable design logic for next-generation multifunctional composites.

We have reinforced this core principle in the revised manuscript, emphasizing that the contribution of this work lies in distilling and validating the fundamental logic of cross-functional integration, rather than merely reporting another high-performance composite, with the aim of offering an inspirational design framework for related fields.

(Page 3, lines 55-59; Page 8, lines 165-167; Page 22, lines 483-492; Page 23, lines 493-494)

Question 2: A quantitative or predictive model correlating microstructure (alignment, porosity, or layer aspect ratio) to multifunctional performance ($\kappa_{//}$, ε_{8-13} μm, and rate-dependent modulus) would greatly enhance scientific value.

Our reply: Thank you for your professional inquiry. To rigorously correlate microstructure with multifunctional properties, we perform thermal and mechanical simulations using finite-element analysis software.

We parameterized alignment by varying the angle between the nanoplatelets and the in-plane direction (10°, 45°, 80°), and controlled porosity by varying the number of nanoplatelets (200, 150, 100). The layer aspect ratio is defined as the diameter-to-thickness ratio of the nanoplatelets (10:1, 20:1, 30:1) (Supplementary Fig. 25a). By systematically combining these parameters to construct 27 distinct conditions, we have identified clear trends: higher alignment degree, lower porosity, and smaller layer aspect ratio substantially enhance thermal performance, emissivity, and mechanical properties (Supplementary Fig. 25b-d). The corresponding discussion has been added to supplementary information (Page 18, lines 377-381; Page S16, lines 222-224).

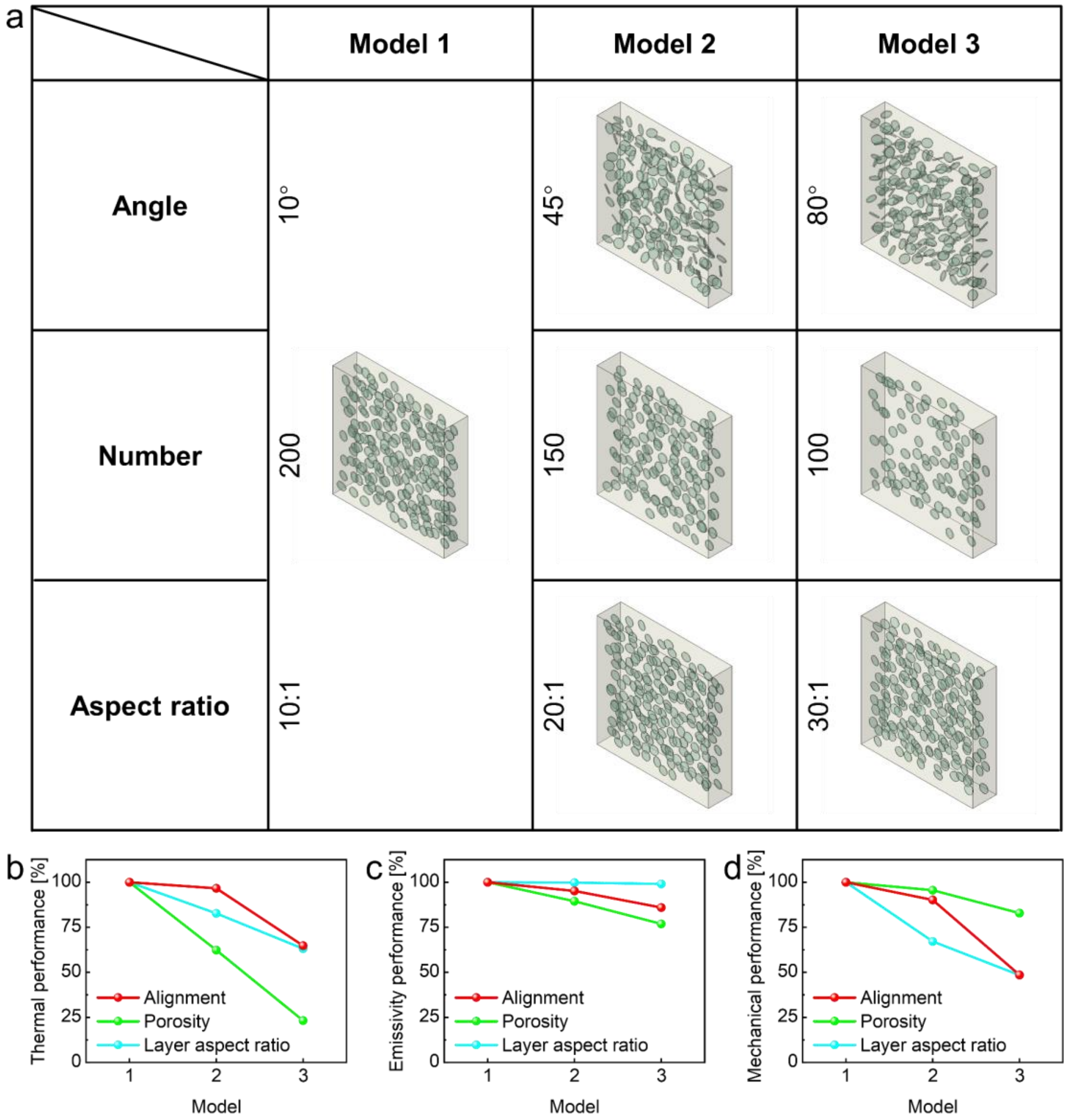

Supplementary Fig. 25 | a Parametric modeling of STBC. **b** Thermal, **c** emissivity, and **d** mechanical performance as a function of the model parameters.

We appreciate your insightful comments. Moving forward, we will undertake a more in-depth investigation into the regulatory mechanisms through which specific microstructural parameters influence multifunctional behavior. This foundation will guide subsequent research and the dissemination of related findings.

Question 3: Many studies have already used BN-based fillers to achieve high thermal conductivity; here the performance appears largely due to high BN loading, not network efficiency. Please clarify whether the composite achieves enhanced conduction via

percolation pathways or simply filler crowding.

Our reply: Thank you for raising this critical question regarding the thermal conduction mechanism. The high thermal conductivity of STBC arises not from simply increasing the loading of BN-based fillers, but from efficient heat transport along oriented and continuous conduction pathways established by a 1D/2D interpenetrating network.

First, while BN-based fillers are widely used in thermal management materials, the innovation of this work is to challenge the conventional assumption that "higher loading yields higher conductivity". Through a biomimetic ordered design, the fillers form an efficient percolative network rather than disordered agglomerates. Traditional high-BN-loading strategies often suffer from filler aggregation, elevated interfacial thermal resistance, and compromised mechanical properties. By contrast, the 1D/2D interpenetrating network in STBC drives the self-assembly and alignment of 2D BNNS, creating continuous and directionally percolating heat-transfer pathways. This orientation design is supported by finite-element thermal analyses (Fig. 2d-g). The anisotropic thermal pathways enabled by the heterostructure are central to the enhanced conductivity and substantially exceed the anisotropy typically observed in conventional high-BN composites. These results directly demonstrate that the directionality and efficiency of heat transport originate from network percolation rather than disordered packing. Finally, compared with reported composites that solely rely on high BN content, STBC exhibits a thermal anisotropy of 44.47, markedly surpassing existing materials (Fig. 2c and Supplementary Table 1).

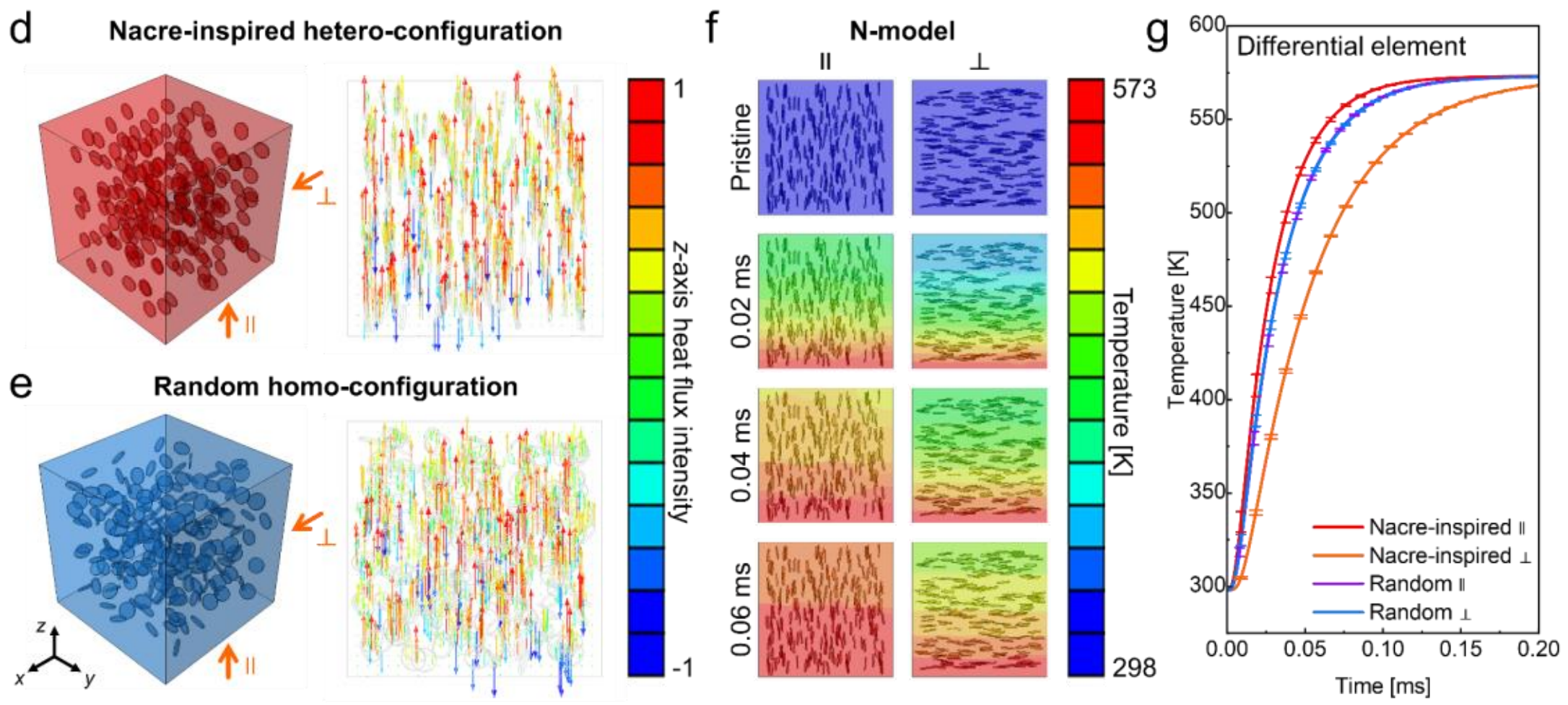

Fig. 2 | d Nacre-inspired hetero-configuration and **e** random homo-configuration models, with corresponding *y-z* plane heat-flux maps at 0.01 ms. **f** Time-resolved temperature maps of the N-model in the parallel and perpendicular directions. **g** Temperature-time traces at differential elements for nacre-inspired and random structures in both directions.

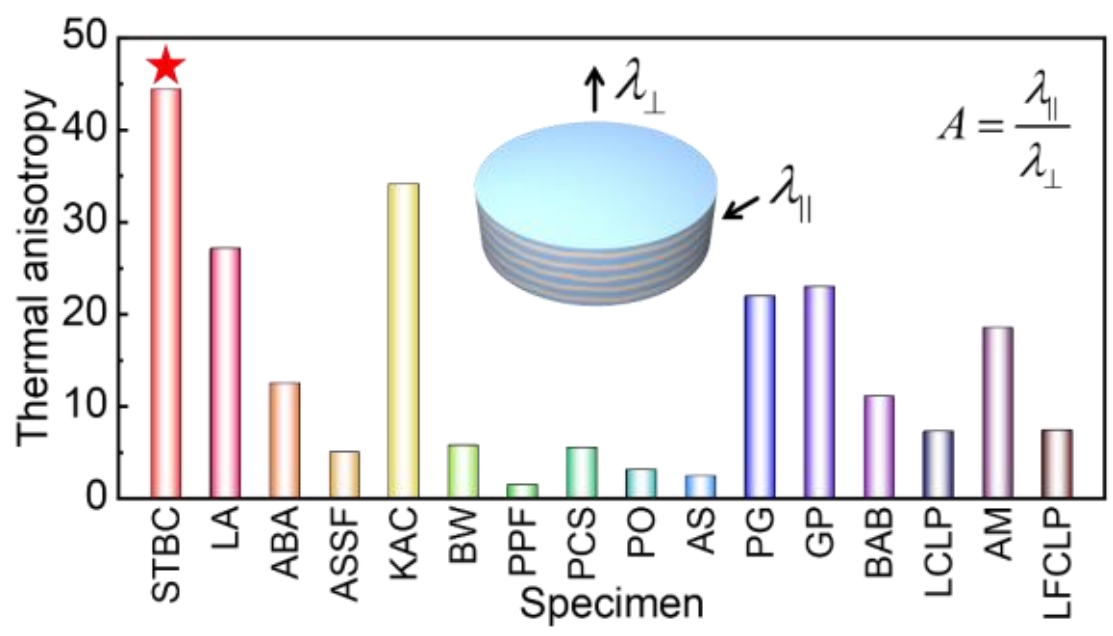

Fig. 2c | Thermal anisotropy of STBC compared with reported thermal interface materials.

Supplementary Table 1 | Thermal anisotropy of STBC and reported thermal interface materials.

Specimen	Thermal anisotropy	Reference
**STBC (This work)**	**44.47**	**This work**
LM/ABS (LA)	27.2	R22
AgNWs@BNNS/ANF (ABA)	12.59	R23

Specimen	Thermal anisotropy	Reference
A-SiC@SiO_2-FAs (ASSF)	5.08	R24
Kaolin/Al/CNF (KAC)	34.19	R25
BNNS/WPU (BW)	5.8	R26
PBA-PDMS/FGf (PPF)	1.57	R27
FCS	5.6	R28
PAN/OSZ (PO)	3.2	R29
AH-SSCSNWA (AS)	2.5	R30
PVA/Graphite (PG)	22.00	R31
GN/PVA (GP)	23.00	R32
(BNNS/ANF)-(AgNWs@BNNS)-(BNNS/ANF) (BAB)	11.14	R33
LC-CNT/LC-PI (LCLP)	7.31	R34
AP/MBLM (AM)	18.55	R35
LC-(FG@CNT)/LC-PI (LFCLP)	7.45	R36

LM: Low-melting-point metal; ABS: Acrylonitrile butadiene styrene; CNF: Cellulose nanofiber; WPU: Waterborne polyurethane; PBA: 2-[[(butylamino)carbonyl] oxy]ethyl ester; PDMS: Polydimethylsiloxane; FGf: Folded graphene film; PAN: Polyacrylonitrile copolymer; OSZ: Oligosilazane; PVA: Polyvinyl alcoho; GN: Graphene@naphthalenesulfonate; BNNS: Boron nitride nanosheets; ANF: Aramid nanofiber; LC: Liquid crystal; CNT: Carbon nanotube; PI: Polyimid; AP: Aramid nanofiber/polyvinyl alcohol; MBLM: MXene-bridging-liquid metal; FG: Fluorinated graphene; PI: Polyimide.

In summary, the high thermal conductivity in this work stems from directional conduction pathways constructed by the biomimetic ordered network, rather than from high BN filler loading indeed. This approach not only addresses the inherent shortcomings of traditional high-loading strategies but also provides a structure-

directed route to thermal enhancement for efficient thermal management material design. The relevant discussion has been added in the revised manuscript. (Page 9, lines 173-177; Page 10, lines 194-196, 198-214; Page 11, lines 215-217)

Question 4: The rationale for using boronic acid (BA) as the dynamic crosslinking unit in the SSG should also be strengthened. Other chemistries (e.g., disulfide, boronic ester variants) have shown excellent performance. Explain why BA chemistry is chosen and what unique advantages it confers.

Our reply: Thank you for your question regarding the selection of the dynamic chemical system. We choose a BA-based SSG because it uniquely combines temperature and stress stability, meeting the thermo-mechanical synergistic requirements of extreme outdoor environments. This combination of properties is difficult to achieve simultaneously with alternative chemistries such as disulfide or boronic ester systems.

In the SSG system, boron is incorporated into organosilicon precursors to form poly(borosiloxane) networks. Owing to the electron deficiency of boron (empty p orbitals) and the presence of lone pairs on oxygen, B-O bonds are established, generating a dynamically crosslinked structural network[R37,R38]. Consequently, SSG remains compliant under slow loading but exhibits high fracture strength at high-strain-rate stretching. The Si-O backbone is comparatively robust and engages in reactions with boron only after bond scission at elevated temperatures. By contrast, disulfide linkages and borate species undergo thermal decomposition at high temperatures. Although disulfide bonds and boronic ester species can form dynamic crosslinks with certain substrates, they display limited compatibility toward silicone oils that fail to synthesize shear-stiffening polymers. Moreover, BA-based SSG retains the core feature of reversible dynamic bond scission and reformation (Fig. 4e) while providing excellent thermal stability[R39,R40] and water resistance (Supplementary Fig. 18a), thereby establishing a robust chemical foundation for multi-performance synergy. Moreover, the B-O crosslinks formed by BA exhibit favorable spectral non-absorption characteristics, which is a prerequisite for efficient passive cooling that neither disulfide

nor boronic ester variants can meet (Supplementary Fig. 12).

The selection of BA also aligns with the work's emphasis on simple and sustainable processing and closed-loop recycling. BA-based SSG can be conveniently incorporated using common carriers such as ethanol or acetone (Supplementary Fig. 2). Our 24-month aging tests confirm consistent performance stability, further validating its practical applicability (Fig. 1d).

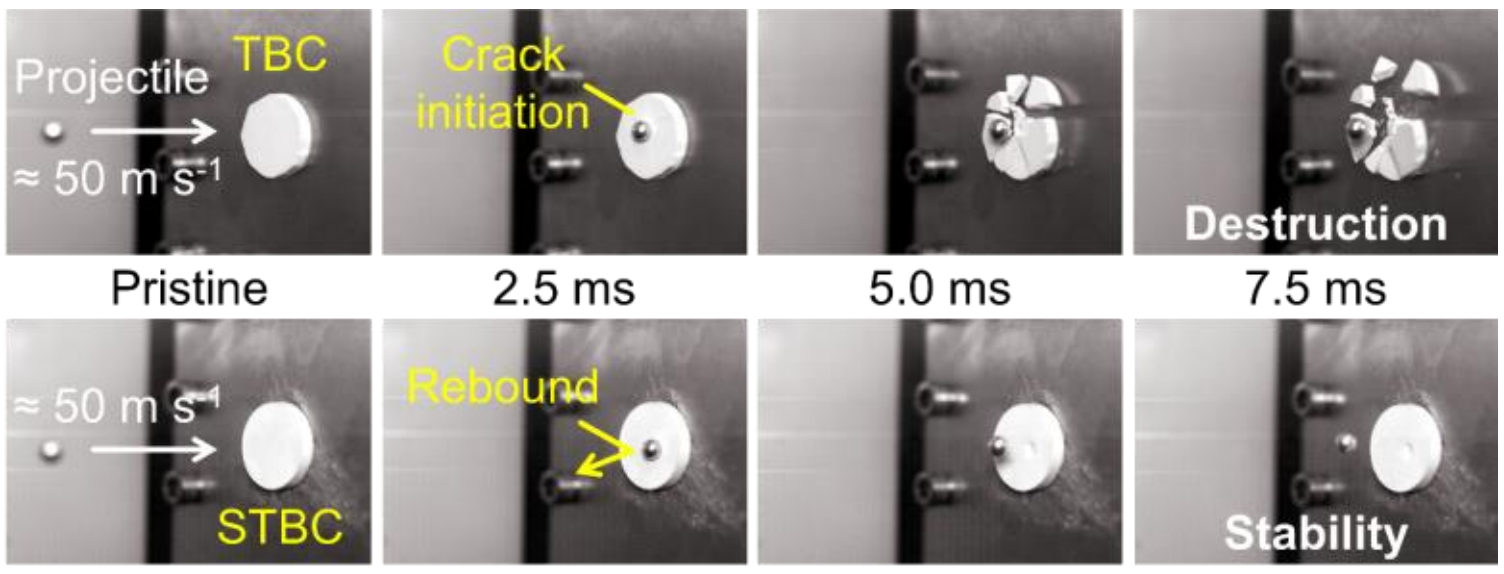

Fig. 4e | High-speed projectile impact processes of TBC and STBC.

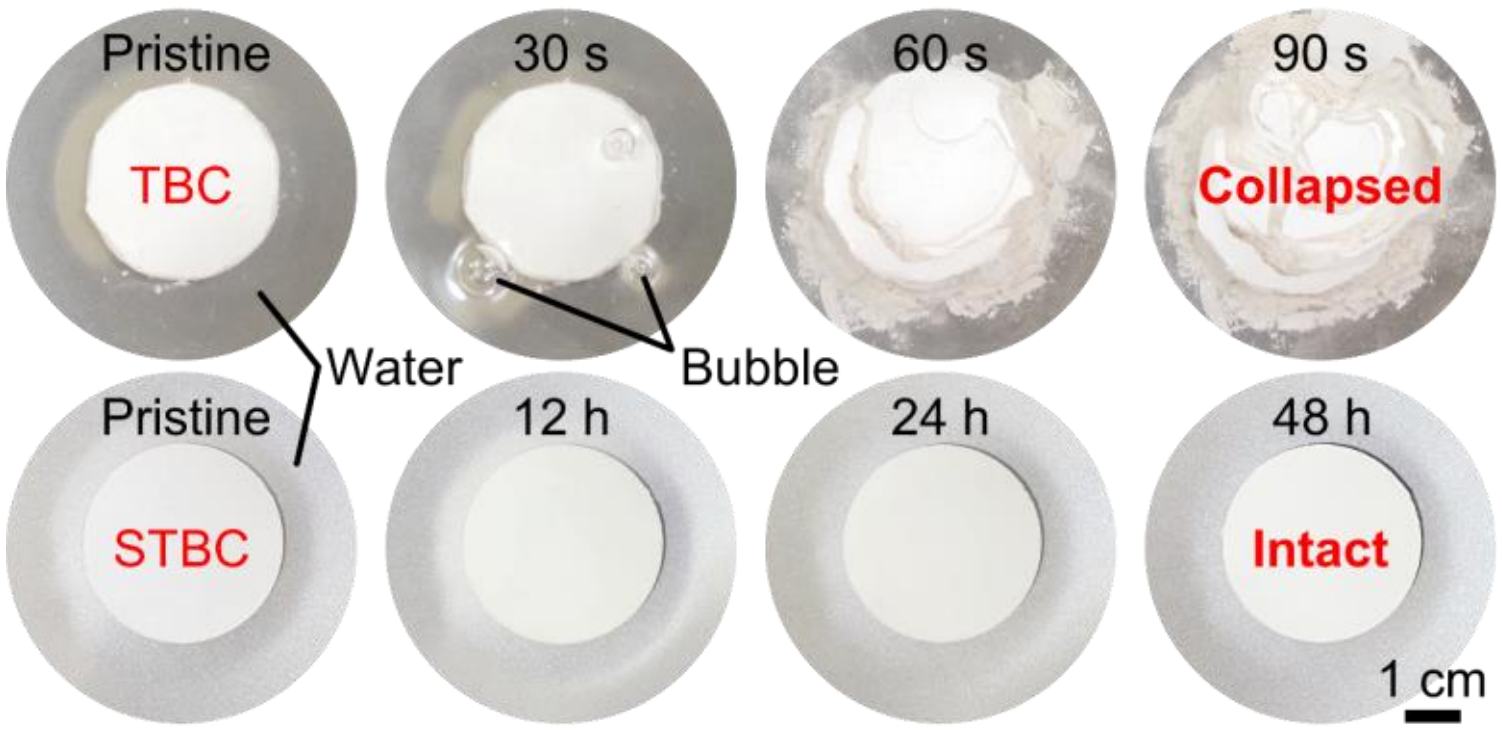

Supplementary Fig. 18a | Water resistance of TBC and STBC.

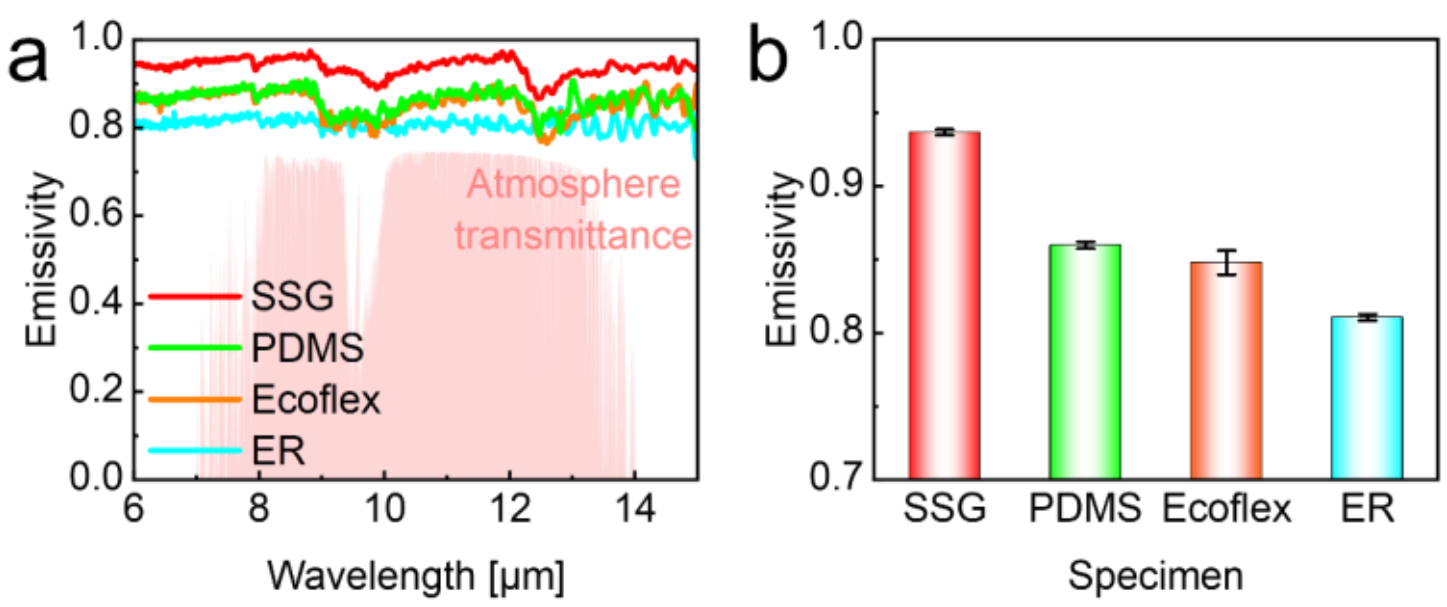

Supplementary Fig. 12 | a Emissivity-wavelength spectra and **b** emissivity comparisons for SSG versus high-molecular-weight PDMS, Ecoflex, and epoxy resin (ER) engineering polymers.

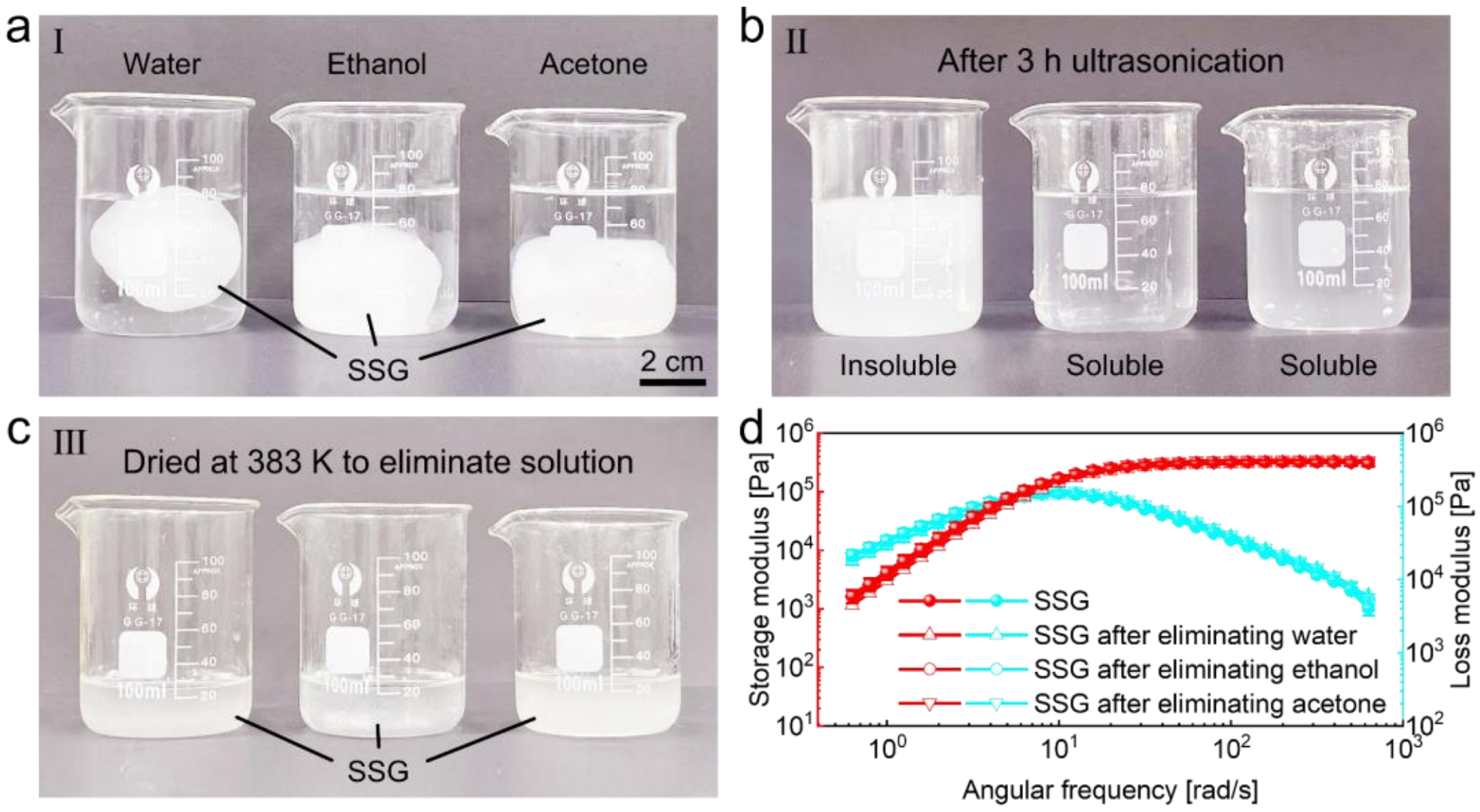

Supplementary Fig. 2 | Stability of SSG in humid and polar environments: **a** Pristine state. **b** After 3 h of ultrasonication. **c** Dried to remove solvent. **d** Corresponding storage and loss moduli.

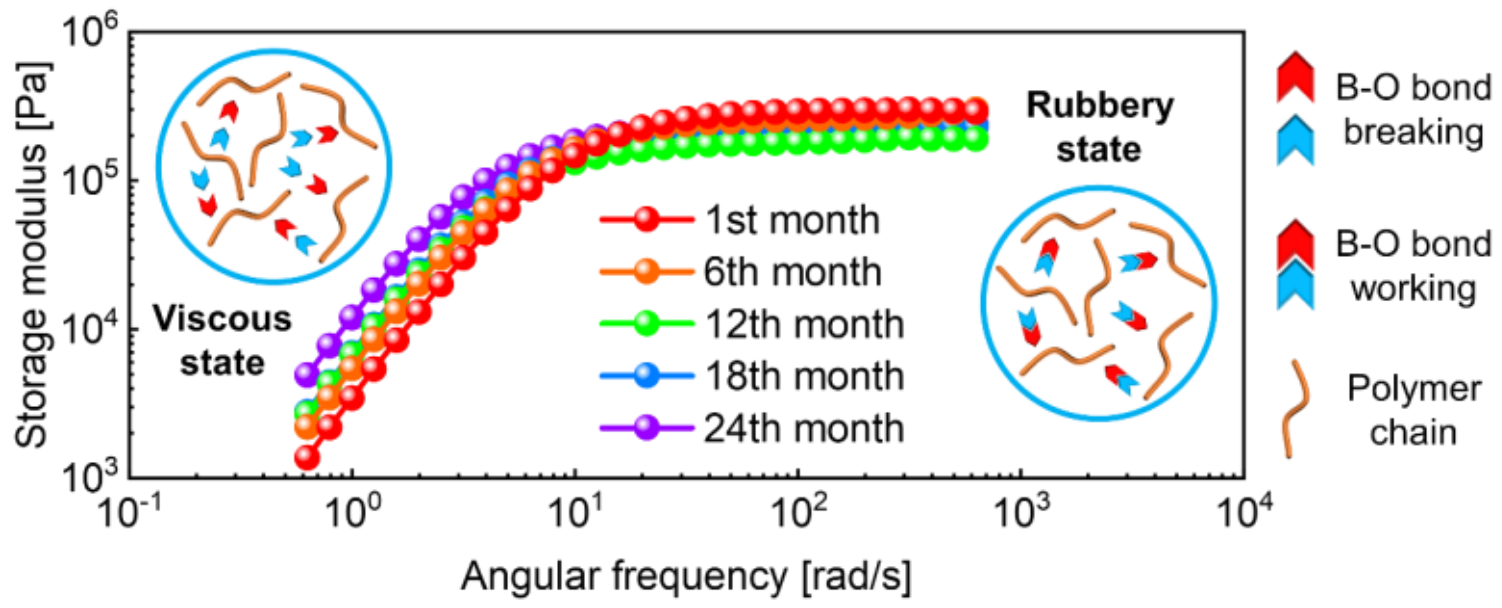

Fig. 1d | Intelligent tunable stiffness and long-term stability of SSG.

However, the primary goal of this work is not merely to improve SSG impact resistance. Rather, it is to realize multifunctional synergistic enhancement by engineering a bioinspired micro-interfacial architecture in tandem with a judiciously selected BA chemistry. The distinctive advantages of the BA system provide essential chemical support for achieving simultaneous improvements across multiple performance metrics. We have made this rationale explicit in the manuscript. (Page 6, line 112; Page 7, lines 140-142; Page 8, lines 159-160; Page 13, lines 256-259; Page 15, line 319; Page 17, lines 354-359; Page S6, lines 111-116; Page S10, lines 162-165; Page S12, lines 187-188)

2. Thermal Characterization and Benchmarking

Question 5: The reported anisotropic thermal conductivity (8.33 W·m^{-1}·K^{-1}, anisotropy ≈ 49) is impressive, but TPS/Hot Disk measurements may overestimate in-plane thermal conductivity for layered anisotropic structures. Validation using laser-flash or 3ω techniques is recommended.

Our reply: Thank you for highlighting this important methodological consideration. Independent cross-validation is indeed essential for ensuring data reliability. Following your suggestion, we verify thermal conductivity using laser-flash analysis (LFA 467, Netzsch, Germany). The measured thermal conductivity of STBC is 7.56 W m^{-1} K^{-1}, which is slightly lower than the initial value obtained via TPS/Hot Disk. We have re-evaluated all parameters related to thermal conductivity to ensure rigor (Fig. 2a-c). The thermal anisotropy of STBC remains at 44.47, exceeding many reported anisotropic composites and supporting its suitability for thermal interface management.

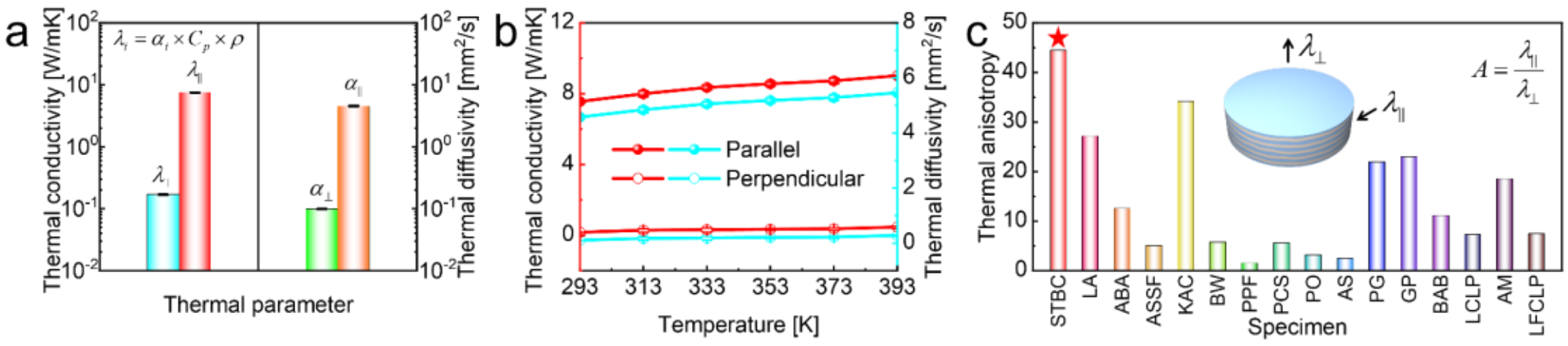

Fig. 2 | a Thermal conductivity and diffusivity of STBC in the parallel and perpendicular directions and **b** their temperature-dependent profiles. **c** Thermal anisotropy of STBC compared with reported thermal interface materials.

Relevant data and methods have been revised accordingly in the revision. (Page 9, lines 171-173, 186; Page 10, lines 187-188, 194-196; Page 26, lines 577-580)

Question 6: Include thermal resistance (°C W^{-1}) in a realistic device stack to show applicability for heat-spreading or cooling devices.

Our reply: Thank you for your suggestion. To evaluate the practical applicability of STBC, we fabricate two realistic device stacked configurations and conduct a systematic comparison of thermal resistance between STBC and a typical heat-

dissipation substrate (aluminum) according to ASTM D 5470 standard. The STBC-based interface exhibits a 50.86% lower interfacial thermal resistance than aluminum, demonstrating substantially enhanced interfacial heat transfer efficiency (Supplementary Fig. 29). These results indicate that STBC is better suited for device-level thermal interface management than aluminum and validate its application potential in thermal conduction and heat-dissipation components.

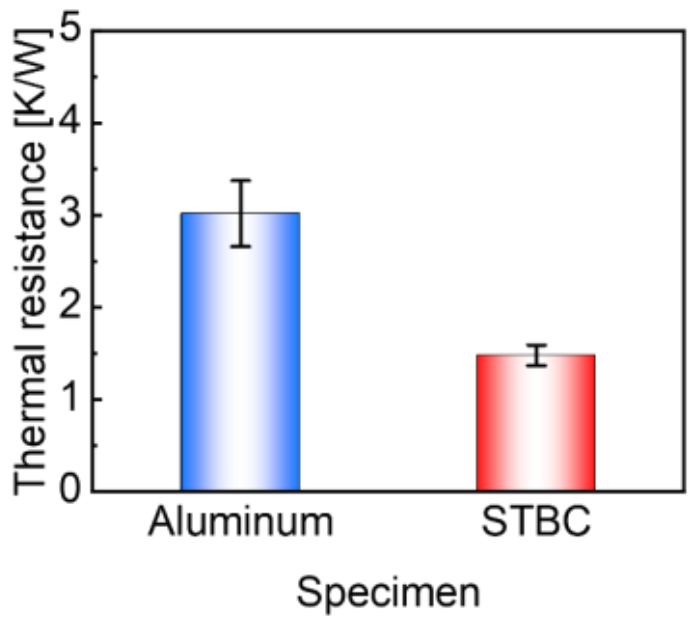

Supplementary Fig. 29 | Comparison of thermal resistance for realistic device stacks employing aluminum versus STBC per ASTM D 5470.

Experimental details have been added to the relevant sections of the manuscript and supplementary information to strengthen the practical relevance and evidentiary rigor of our work. (Page 20, lines 423-425; Page 26, lines 580-583; Page S18, lines 241-245)

Question 7: Specify the BNNS content (wt.% or vol.%) in all tested samples and ensure comparisons of anisotropy are made at similar filler loadings. Clarify whether the nacre-like alignment remains stable across varying BNNS contents.

Our reply: Thank you for highlighting the importance of standardizing material formulations and ensuring rigorous experimental comparisons. As this work focuses on BNNS-based ceramics with a nacre-inspired brick-and-mortar microstructure, the BNNS mass fraction is consistently set at 62 wt.% for all test specimens. This core formulation parameter is explicitly detailed in the Methods section. In addition, the material parameters for all comparison specimens are thoroughly documented in the corresponding experimental sections and supplementary information, ensuring that performance comparisons are conducted at comparable filler loadings and thereby

preserving the objectivity and comparability of the results.

Regarding the stability of nacre-mimetic alignment across varying BNNS contents, the directional vacuum-filtration self-assembly process employed in this work enables BNNS to maintain a robust and ordered alignment. During filtration, 2D nanoplatelets progressively accumulate on the membrane surface and undergo constrained and directional alignment. Owing to their planar geometry, nanoplatelets preferentially orient horizontally, stacking with their planes parallel to the filter surface under the combined influence of fluid shear and pressure normal to the membrane. This behavior accords with prior report[R41], further validating the reliability and generality of our structural design.

We have supplemented the manuscript with these data annotations and experimental details to ensure transparency in material formulations and rigor in performance comparisons, thereby providing stronger support for our conclusions. (Page 8, lines 152-155; Page 26, lines 591-592)

Question 8: Compare radiative-cooling performance under standard outdoor sub-ambient tests (midday, clear-sky, angular dependence) with leading RC coatings or composites.

Our reply: We appreciate your valuable suggestion, which is essential for positioning the performance of our work. We compare the radiative cooling performance with a leading commercial RC composite (Coinno Intelligent Technology Co. Ltd., Hainan, China). Under standard 1 solar irradiation, the saturated steady-state temperatures of STBC and the commercial product are 333.6 and 352.1 K, respectively (Fig. 3d). To account for real-world variability, we further evaluate the angular dependence of sub-ambient radiative cooling outdoors under clear-sky conditions in Hefei, China. Across forenoon, midday, and afternoon testing, STBC consistently exhibit substantially lower temperatures than the commercial product, maintaining robust cooling under different solar incidence angles (Fig. 3e). Together, the controlled and field evaluations indicate that STBC delivers superior cooling efficiency compared with the commercial benchmark.

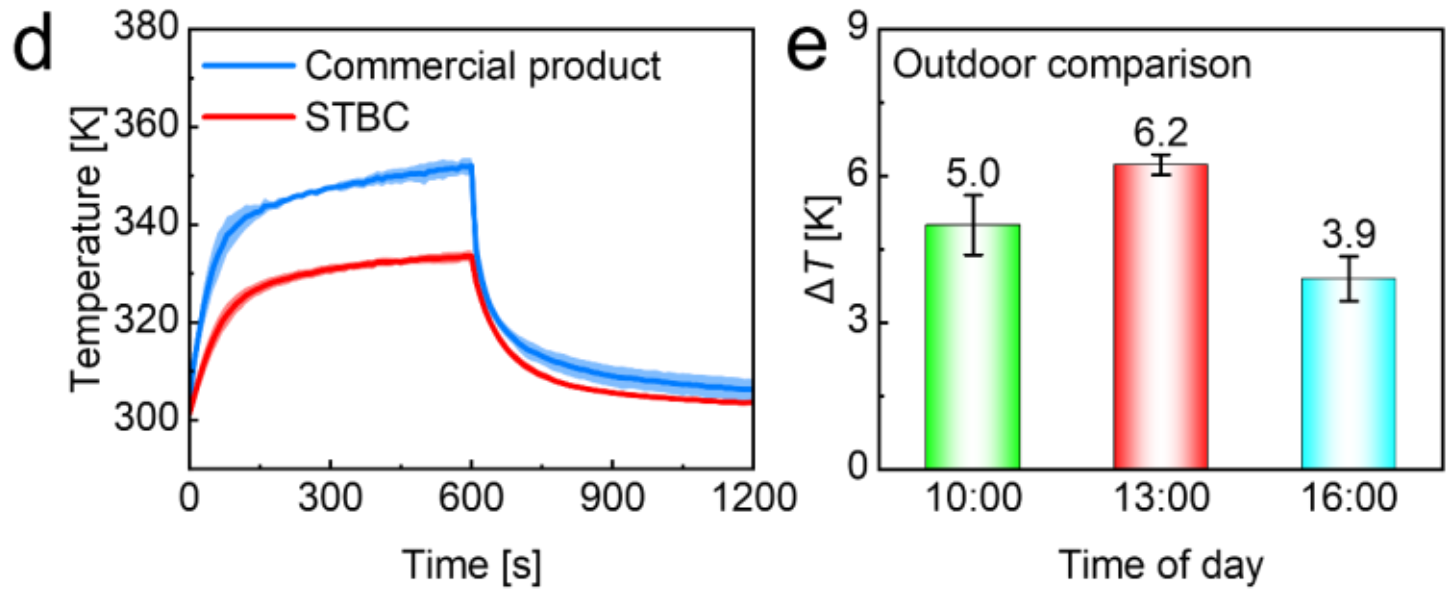

Fig. 3 | Time-dependent temperature comparisons between a commercial radiative cooling product and STBC under **d** 1 sun exposure and **e** real-world outdoor sub-ambient conditions.

The comparative data and corresponding discussion have been incorporated into the revised manuscript. (Page 12, lines 240-241; Page 13, lines 267-274)

3. Mechanical and Impact Response

Question 9: The impact and drop tests are persuasive visually, but remain qualitative. Provide standardized metrics such as transmitted-force, absorbed-energy, or resilience following ISO/ASTM/NIJ standards.

Our reply: Thank you for the valuable suggestion. To enhance the objectivity and rigor of the impact-resistance assessment, we supplemented quantitative testing and analysis in accordance with ISO 6603-2:2023, which prescribes standardized procedures for evaluating the puncture impact behavior of rigid composites. We employ an instrumented drop-tower impact method to quantify puncture behavior via the force-displacement response during impact (Fig. 4f), described as follows:

$$E_j = \int_0^{l_j} F(l)\,\mathrm{d}l,\ j = \mathrm{m}, \mathrm{p}, \tag{1}$$

where $F(l)$ is the impact force as a function of displacement l, and l_j is the impact displacement. The subscripts m and p refer to the maximum value and puncture points, respectively. At an impact height of 50 cm, STBC achieves an absorbed-energy of 107.64 J m^{-1} and a maximum force of 61.10 kN m^{-1}. To further highlight STBC's advantages, we systematically benchmark its performance against multiple state-of-the-

art and high-performance engineering materials. The results show that STBC significantly outperforms these materials in both specific puncture energy and maximum impact force (Supplementary Table 4), fully validating its superior impact protection.

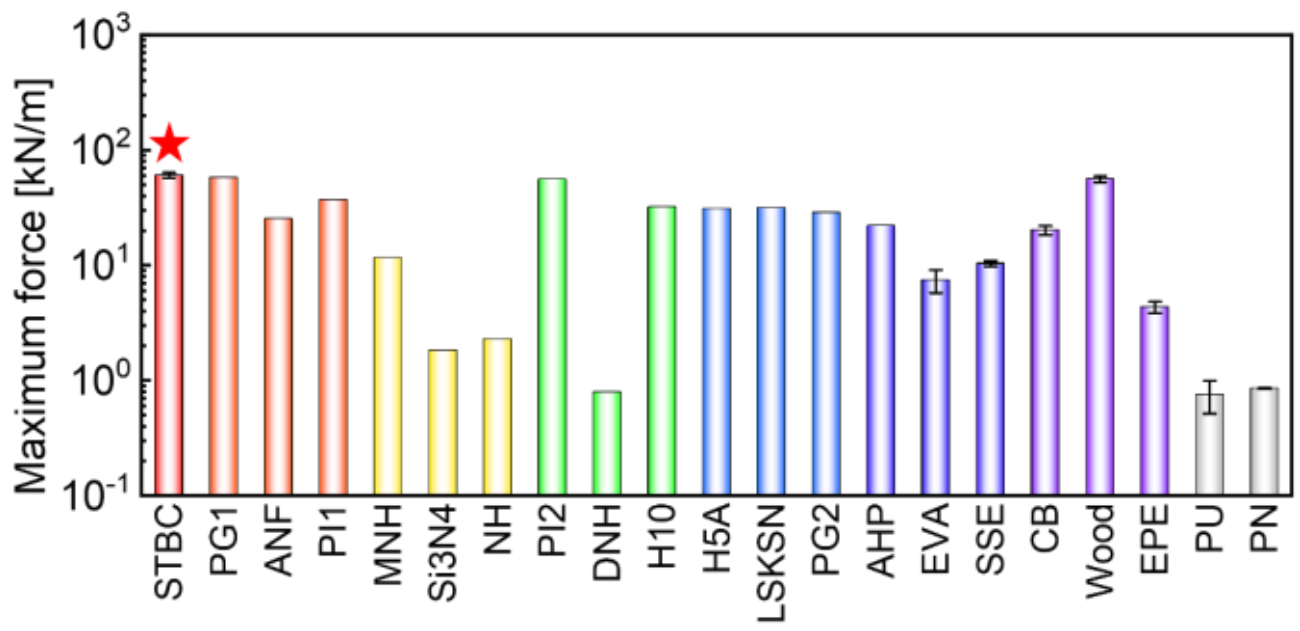

Fig. 4f | Maximum force of STBC compared with various state-of-the-art and high-performance engineering materials in instrumented impact tests.

Supplementary Table 4 | Impact resistance of STBC compared with state-of-the-art and high-performance engineering materials.

Specimen	Maximum force [kN m^{-1}]	Specific puncture energy [J m^{-1}]	Reference
**STBC**	**61.10**	**107.64**	**This work**
PVA/GO (PG1)	58.25	/	R1
ANF	25.43	98.28	R2
PPGTD-IPDA (PI1)	37.25	/	R3
MNH	11.7	10.8	R4
Si_3N_4	1.84	/	R5
NH	2.3	0.55	R6
PDO-IP2.5 (PI2)	≈56	/	R7
DNH	0.8	0.53	R8
HPDU10 (H10)	32.13	/	R9
Hex[5A] (H5A)	31.25	37.5	R10
Leather/SSG/Kevlar/ STF/NWF (LSKSN)	32	/	R11
PL-Gel (PG2)	28.95	2.6	R12

Specimen	Maximum force [kN m^{-1}]	Specific puncture energy [J m^{-1}]	Reference
AA/HEMA/PEG (AHP)	22.1	/	R13
EVA	7.51	100.30	The data are derived from this work.
SSE	10.45	93.81	
CB	20.40	89.12	
Wood	56.61	75.12	
EPE	4.37	34.56	
PU	0.76	8.02	
PVC/NBR (PN)	0.86	4.74	

Both maximum force and specific puncture energy are normalized by specimen thickness. PVA: Poly(vinyl alcohol); GO: Graphene oxide; ANF: Aramid nanofiber; PPGTD: PPG, tolylene 2,4-diisocyanate terminated; IPDA: Isophoronediamine; MNH: Microsphere-reinforced nanocomposite hydrogel; NH: Nanocomposite hydrogel; PDO: 2, 4-pentanedione dioxime; DNH: Double network hydrogel; HPDU: Hyperbranched polyurethane; SSG: Shear-stiffening gel; STF: Shear-thickening fluid; NWF: Non-woven fabric; AA: Acrylic acid; HEMA: Hydroxyethyl methacrylate; PEG: Polyethylene glycol; EVA: Ethylene vinyl acetate copolymer; SSE: Shear-stiffening elastomer; CB: Corrugated board; EPE: Expanded polyethylene; PU: Polyurethane; PVC: Polyvinyl chloride; NBR: Nitrile butadiene rubber. SSE is developed based on prior work[R14], while the engineering specimens used for comparison are commercially available products.

The relevant experiments and analyses have been incorporated into the revised manuscript. (Page 17, lines 363-364, 367-370)

Question 10: Include rate-dependent modulus data (DMA or SHPB) to distinguish contributions from the SSG's shear-stiffening versus the nacre-like architecture.

Our reply: Thank you for this highly insightful question. Following your recommendation, we conduct SHPB tests at a strain rate of 2000 s^{-1} (Supplementary Fig. 22b) and DMA at frequencies from 25 to 50 Hz (Supplementary Fig. 22c) on the SSG matrix, the nacre-like TBC architecture, and the STBC composite, to delineate the

contribution of each subcomponent to impact resistance. The dynamic compressive strength and storage modulus of STBC exceed those of both individual subcomponents (SSG matrix and nacre-like TBC). The shear-stiffening behavior of SSG and the nacre-like architecture synergistically enhance the composite's mechanical performance, yielding an effectively additive improvement[R15,R16].

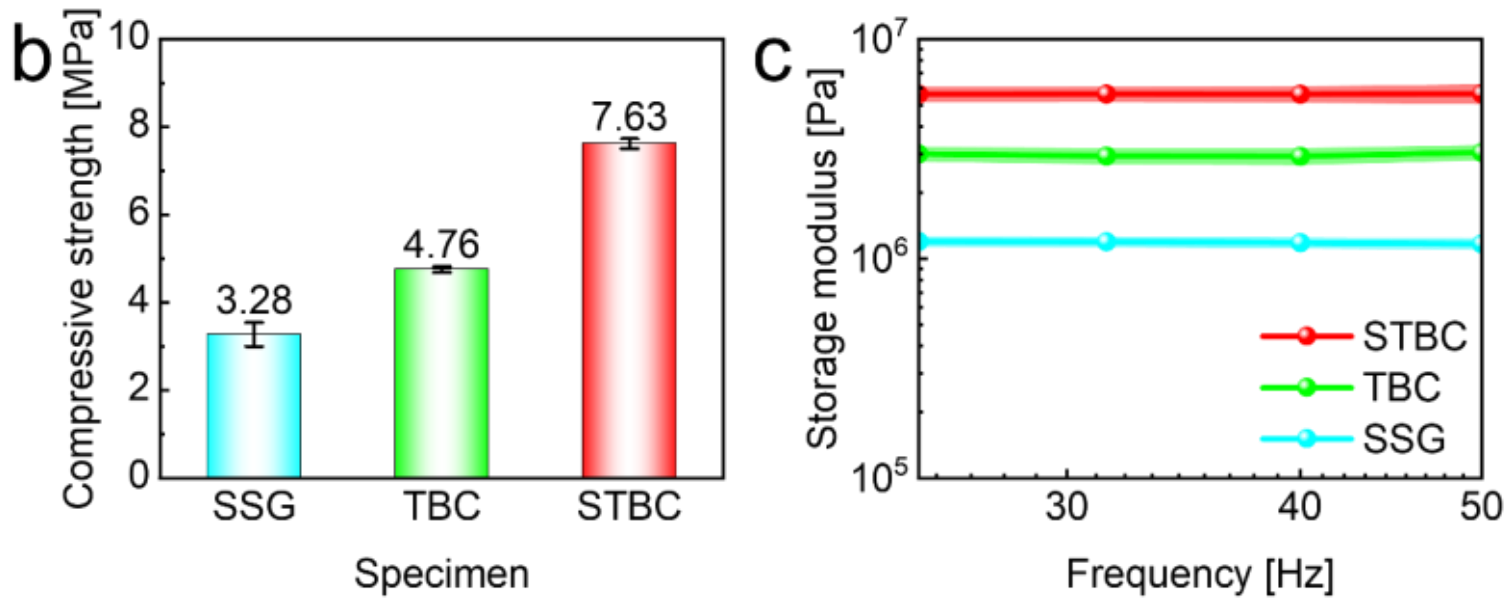

Supplementary Fig. 24 | b Compressive strength of SSG, TBC, and STBC at 2000 s^{-1} from SHPB tests. **c** Storage modulus of SSG, TBC, and STBC from DMA.

The corresponding experiments and discussion have been added to the revised manuscript and supplementary information. (Page 17, line 360; Page 26, lines 599-600; Page 27, lines 603-604; Page S14, lines 206-213)

Question 11: Clarify long-term SSG stability: Fig. 1d shows strain-rate sweeps at multiple intervals (1–24 months), but the text lacks explanation. Please state that periodic tests confirm temporal stability of the dynamic B–O network, showing negligible modulus change and consistent stiffening behavior.

Our reply: Thank you for pointing out the need for a more precise interpretation of the long-term stability data (Fig. 1d). Accurate interpretation of these results is crucial for demonstrating the durability of the dynamic B-O network. Based on your suggestion, we revise the text for clarity and coherence as follows: "24-month periodic strain-rate sweep tests confirmed the temporal stability of the dynamic B-O network, showing negligible modulus change and consistent stiffening behavior." (Page 7, lines 140-142)

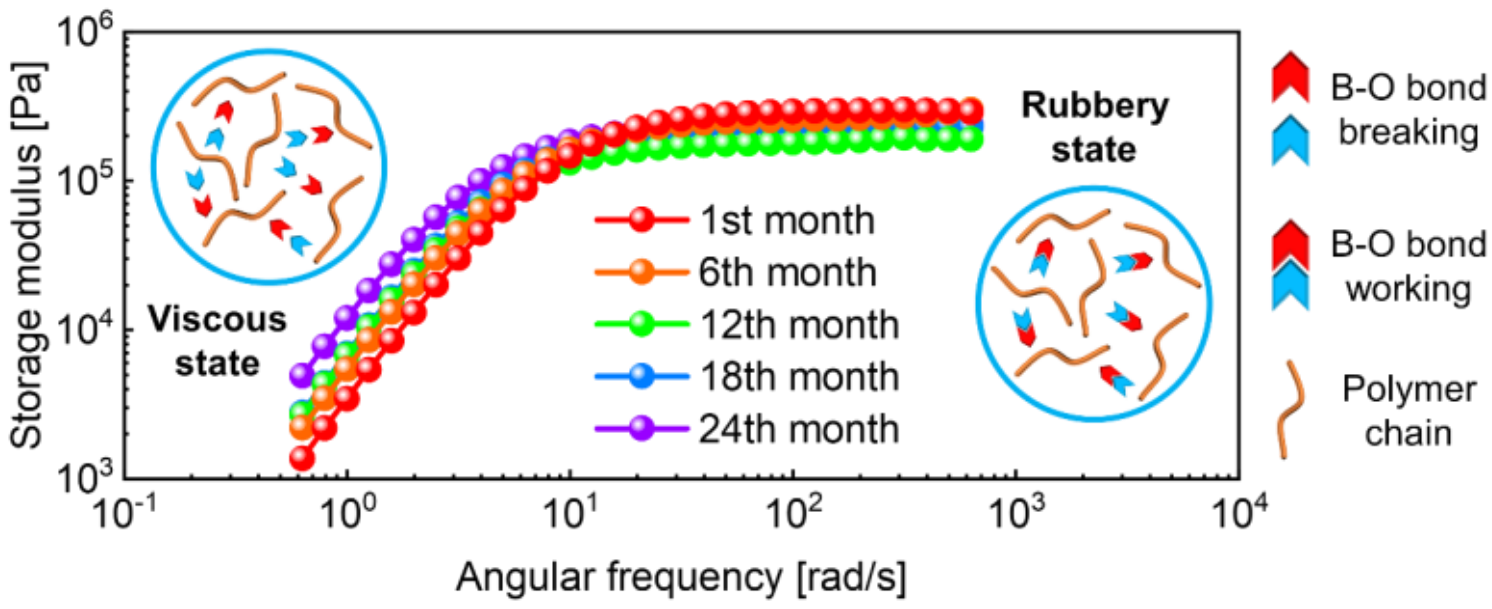

Fig. 1d | Intelligent tunable stiffness and long-term stability of SSG.

4. Durability, Flammability, and Recyclability

Question 12: For Fig. 2h, include quantitative flammability metrics (UL-94, LOI, or cone calorimetry: PHRR/THR/TTI) to benchmark fire resistance.

Our reply: Thank you for your valuable suggestion. Fig. 2h highlights the exceptional flame-retardant performance of STBC. To strengthen this section with quantitative flammability metrics, we conduct horizontal and vertical burning tests, LOI measurements, and cone calorimetry characterization. In the horizontal burning test, STBC exhibits no observable combustion (Supplementary Fig. 8a). In the vertical burning test, STBC rapidly self-extinguishes after two 10 s ignitions without generating dripping particles (Supplementary Fig. 8b). Its LOI reaches 69%, and post-combustion STBC retains over 90% of its thermal conductivity, reflectance, and mechanical strength (Fig. 2j). Cone calorimeter results further indicate a low PHRR and THR (Supplementary Fig. 9).

h

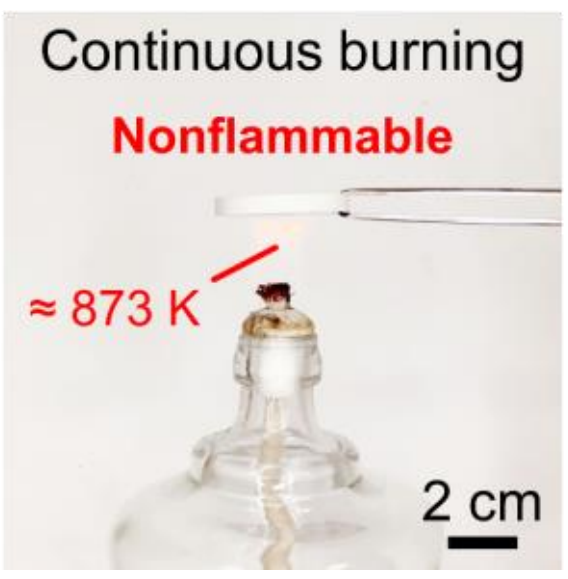

Fig. 2h | Flame impact resistance of STBC.

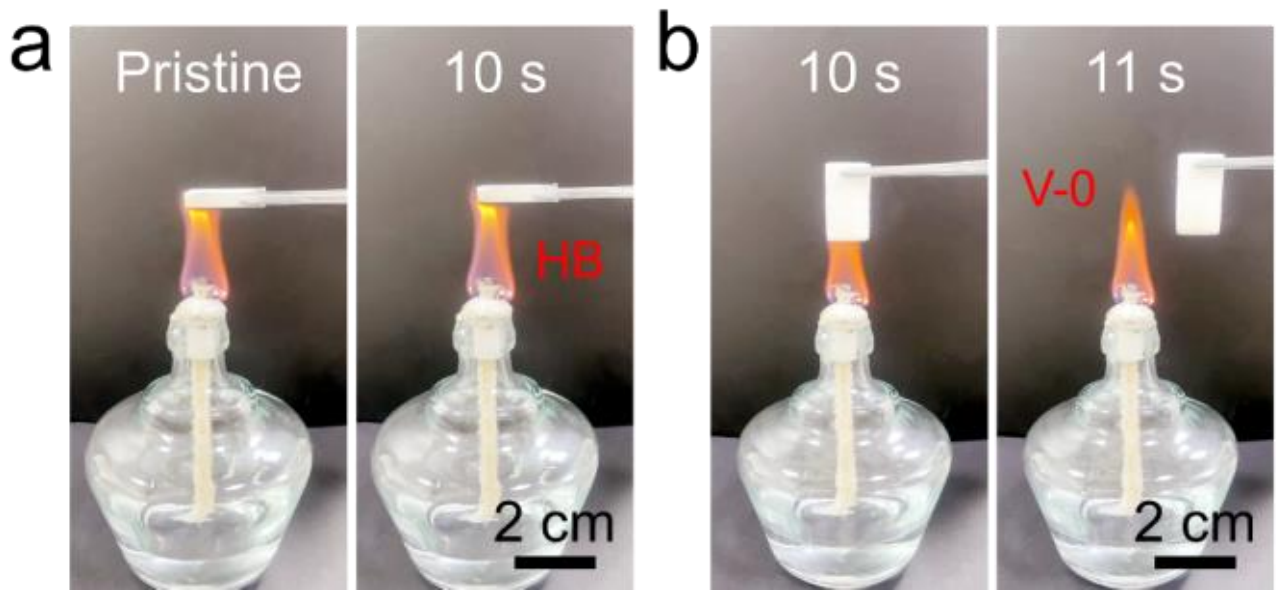

Supplementary Fig. 8 | a Horizontal and **b** vertical burning of STBC.

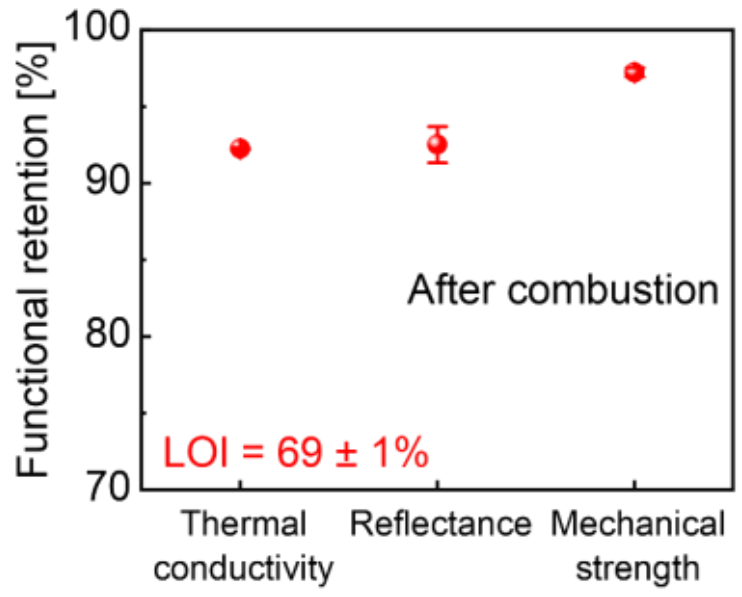

Fig. 2j | Retention of functional performance in STBC after flame impact.

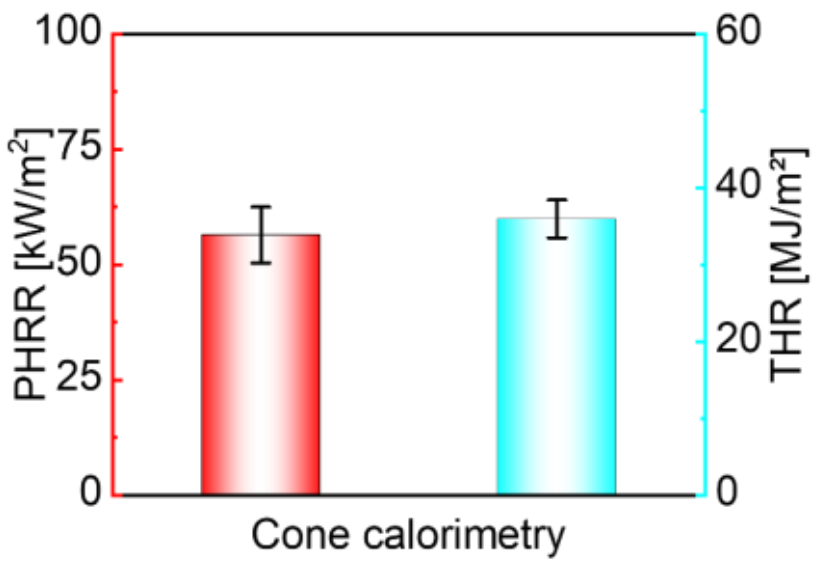

Supplementary Fig. 9 | Peak heat release rate and total heat release of STBC under fire exposure.

Collectively, these comprehensive quantitative tests establish a rigorous benchmark for the fire resistance of STBC. The corresponding quantitative data have been incorporated into the revised manuscript. (Page 9, lines 179-180; Page 11, lines 218-229; Page S9, lines 143-147, 149-151)

Question 13: The statement that STBC remains intact after 1 h at 600 °C should be supported by post-flame performance tests (thermal conductivity, reflectance,

mechanical strength) to verify functional retention beyond visual appearance.

Our reply: We appreciate your valuable suggestions, which enable a more comprehensive validation of functional stability under extreme high-temperature exposure. We systematically measure the thermal conductivity, reflectance, and mechanical strength of STBC after flame impact. Mechanical strength is assessed via oscillatory shear rheology at 0.1% strain and 1 Hz. The results show that post-burn STBC retains over 90% of its thermal conductivity, reflectance, and mechanical strength, with no significant performance degradation (Fig. 2j). These findings demonstrate that STBC not only maintains structural integrity after extreme thermal exposure but also effectively preserves its core thermal-management and mechanical protection functions.

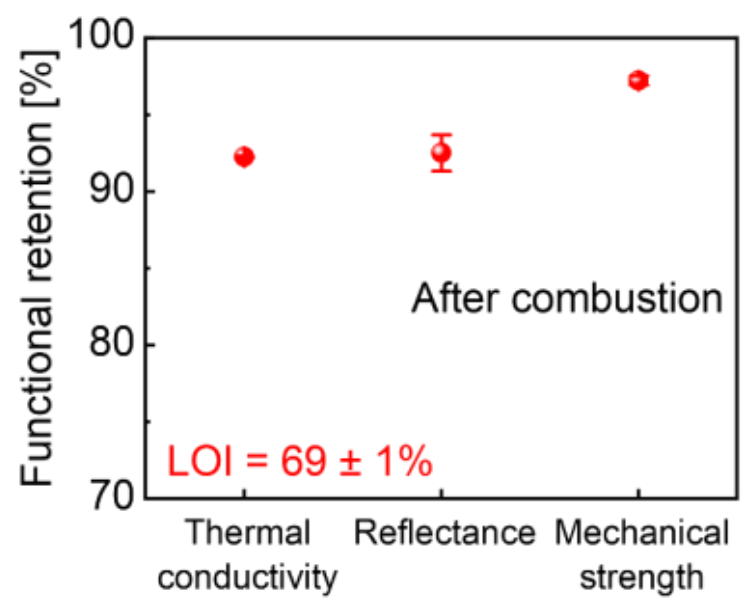

Fig. 2j | Retention of functional performance in STBC after flame impact.

The corresponding experiments and discussion have been added to the manuscript. (Page 9, lines 179-180; Page 11, lines 224-228)

Question 14: The recyclability claim (Fig. 4h) suggests the SSG dissolves in ethanol, implying borosiloxane matrix lability to polar solvents. Clarify whether this recyclability derives from intentional dynamic bond reversibility or solvent-induced degradation. Provide quantitative stability data in polar and humid environments.

Our reply: Thank you for your insightful questions regarding the recyclability mechanism and environmental stability of SSG in Fig. 4h. The recyclability of SSG derives from intentionally engineered dynamic bond reversibility[R15,R16]. To verify this, we perform performance-recovery tests by immersing SSG in water (humid

environment), ethanol, and acetone (polar environment) (Supplementary Fig. 2a). After 3 h of ultrasonication, SSG dissolves in ethanol and acetone yet remains insoluble in water (Supplementary Fig. 2b). Subsequently, all samples are dried at 383 K to remove solvent. In each case, the original SSG is reconstituted, confirming reversibility (Supplementary Fig. 2c). Rheological comparisons before and after processing show that the storage and loss moduli are essentially unchanged, demonstrating performance consistency (Supplementary Fig. 2d). Quantitative analysis confirms that the performance of SSG is robust in both humid and polar environments.

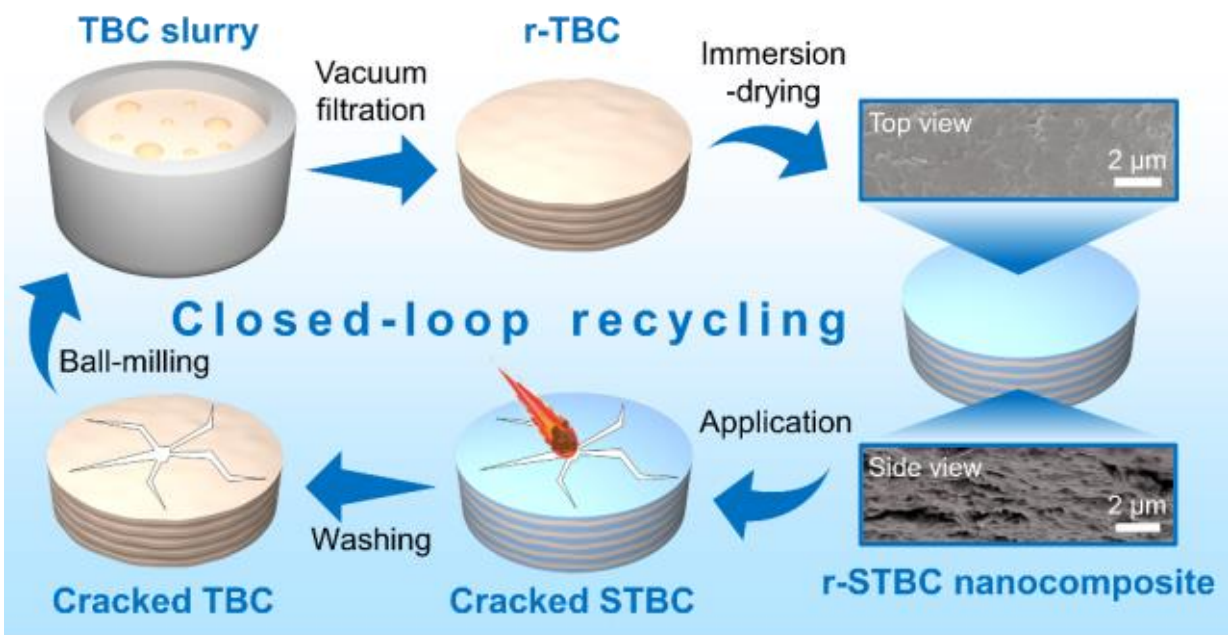

Fig. 4h | Schematic of the closed-loop recycling process for STBC.

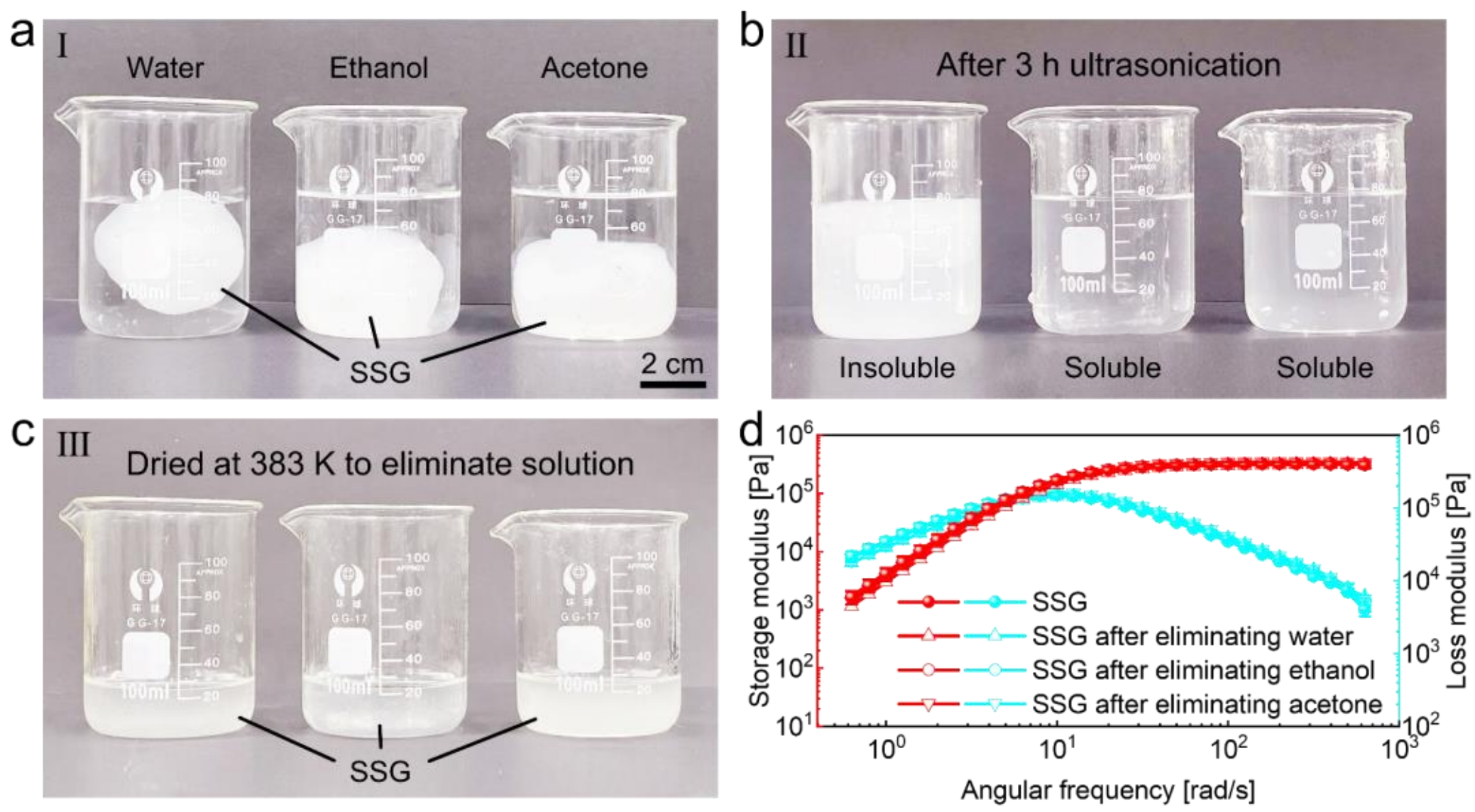

Supplementary Fig. 2 | Stability of SSG in humid and polar environments: **a** Pristine state. **b** After 3 h of ultrasonication. **c** Dried to remove solvent. **d** Corresponding storage and loss moduli.

Relevant experiments and data have been incorporated into the revised manuscript

and supplementary information. (Page 8, lines 159-160; Page S6, lines 111-116)

Question 15: The reported "3 h water stability" is short for outdoor applications; include mass retention, swelling ratio, and mechanical retention after longer immersion (≥24 h) and cyclic wet-dry testing.

Our reply: Thank you for your inquiry regarding the duration of our pervious water-stability test. To better assess outdoor applicability, we extend the immersion period from 3 h to 48 h. TBC disintegrates within 90 s in water, whereas STBC maintains structural integrity after 48 h of full immersion, with no visible signs of disintegration or deformation (Supplementary Fig. 18a). In addition, STBC is subjected to 10 wet-dry cycles consisting of 5 min immersion in water followed by 5 min drying at 353 K. Mechanical retention is evaluated via oscillatory shear rheology at 0.1% strain and 1 Hz. After prolonged immersion and cyclic wet-dry exposure, mass, dimensional, and mechanical retentions remain within 99-101%, confirming robust structural and performance stability under water exposure (Supplementary Fig. 18b). These results provide a solid experimental foundation for outdoor use in environments with moisture and rainfall.

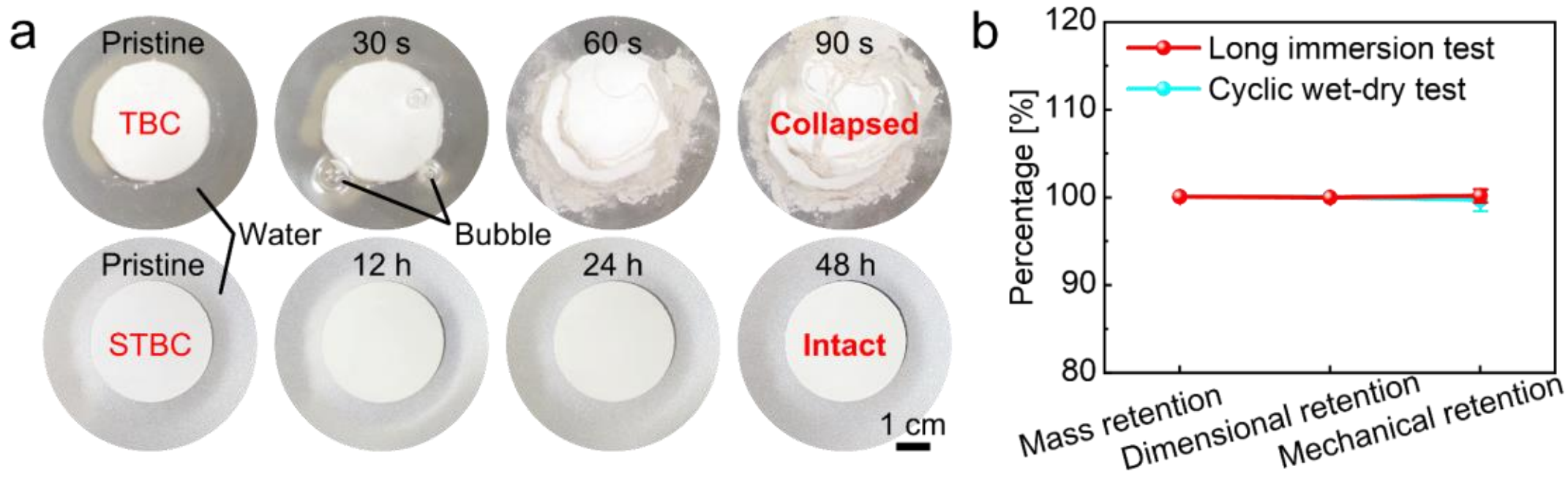

Supplementary Fig. 18 | a Water resistance of TBC and STBC. **b** Mass, dimensional, and mechanical retention of STBC after prolonged immersion and cyclic wet-dry tests.

The corresponding experiments and discussion have been added to the revised manuscript and supplementary information. (Page 14, lines 301-306; Page S12, lines 187-189; Page S13, lines 190-191)

Question 16: For Supplementary Figs. 11–13, add data for repeated immersion–drying cycles and quantitative metrics (weight change, dimensional change, mechanical strength variation).

Our reply: Thank you for requesting additional quantitative data under repeated immersion-dry cycles. For coherence, the previous Supplementary Figs. 11-13 have been reorganized into a single Supplementary Fig. 18 to improve the continuity of description and interpretation. We supplement the repeated immersion-drying experiments and quantified changes in weight, dimension, and mechanical strength. Mechanical variations are assessed by measuring the storage modulus of STBC before and after cycling using oscillatory shear rheology at 0.1% strain and 1 Hz. All metrics exhibit high stability, with deviations within 1% compared to pristine STBC, validating its robustness under cyclic immersion-drying conditions.

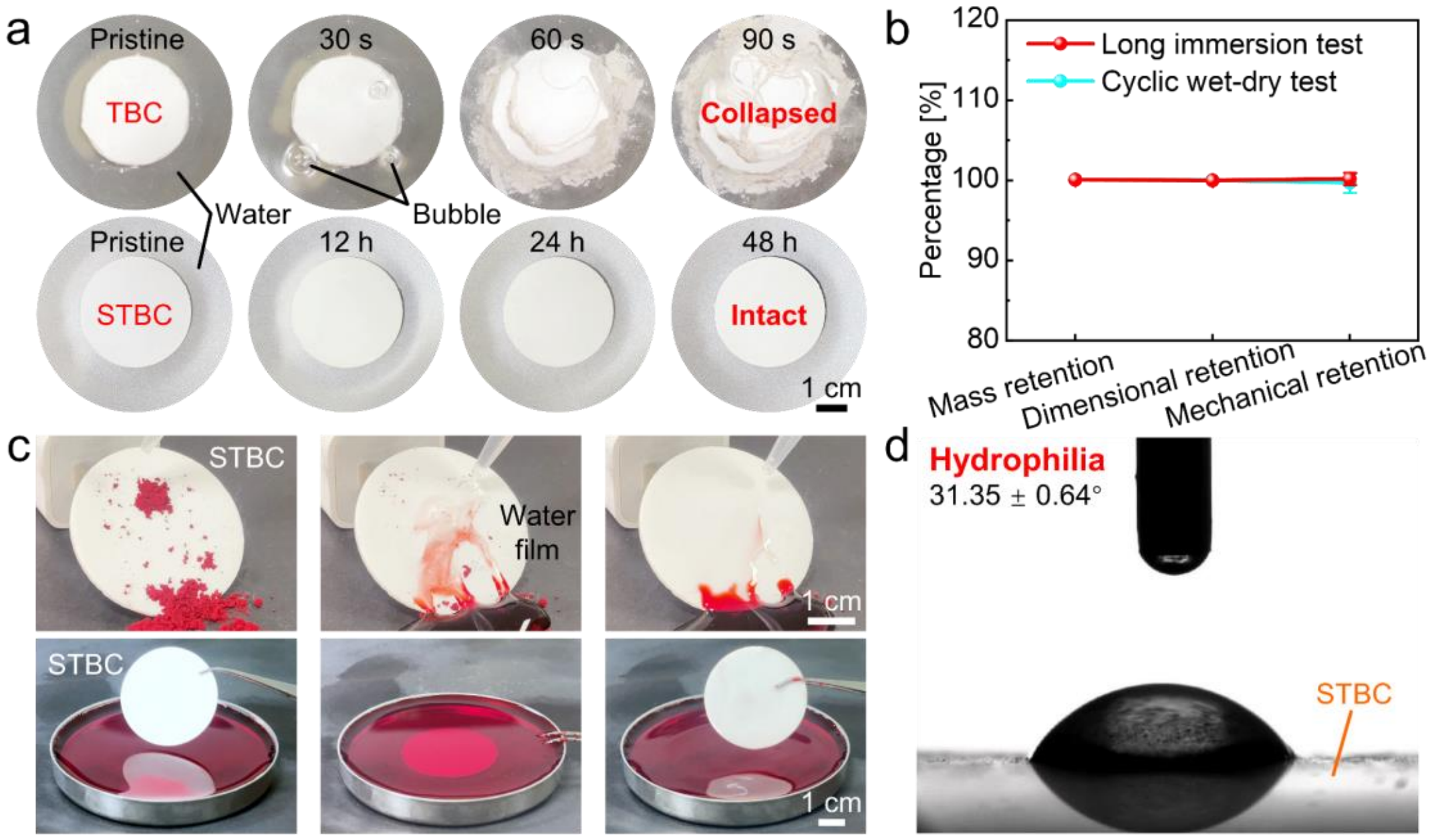

Supplementary Fig. 18 | a Water resistance of TBC and STBC. **b** Mass, dimensional, and mechanical retentions of STBC after prolonged immersion and cyclic wet-dry tests. **c** Self-cleaning and **d** hydrophilic properties of STBC.

Relevant figures and discussion have been incorporated into the revised manuscript and supplementary information. (Page 14, lines 301-306; Page 15, lines 307-310; Page S12, lines 187-189; Page S13, lines 190-191)

5. Durability and Environmental Stability

Question 17: Demonstrate resistance to UV, humidity, and temperature cycling, as these are critical for outdoor applications.

Our reply: Thank you for highlighting this critical point, which is directly relevant to the practical outdoor deployment. Demonstrating resistance to UV, humidity, and temperature cycling is essential for establishing durability. We conducted UV (365 nm, 0-80 W m^{-2} irradiance), humidity (40-85% relative humidity), and temperature (303-333 K) cycling experiments to comprehensively evaluate environmental durability (Supplementary Fig. 21a-c). Mechanical retention is assessed via storage modulus measured in oscillatory shear at 0.1% strain and 1 Hz. Under all cyclic conditions, STBC retains more than 98% of its mechanical performance, irrespective of UV exposure, humidity, or temperature variations, demonstrating stability suitable for long-term reliable outdoor service (Supplementary Fig. 21d).

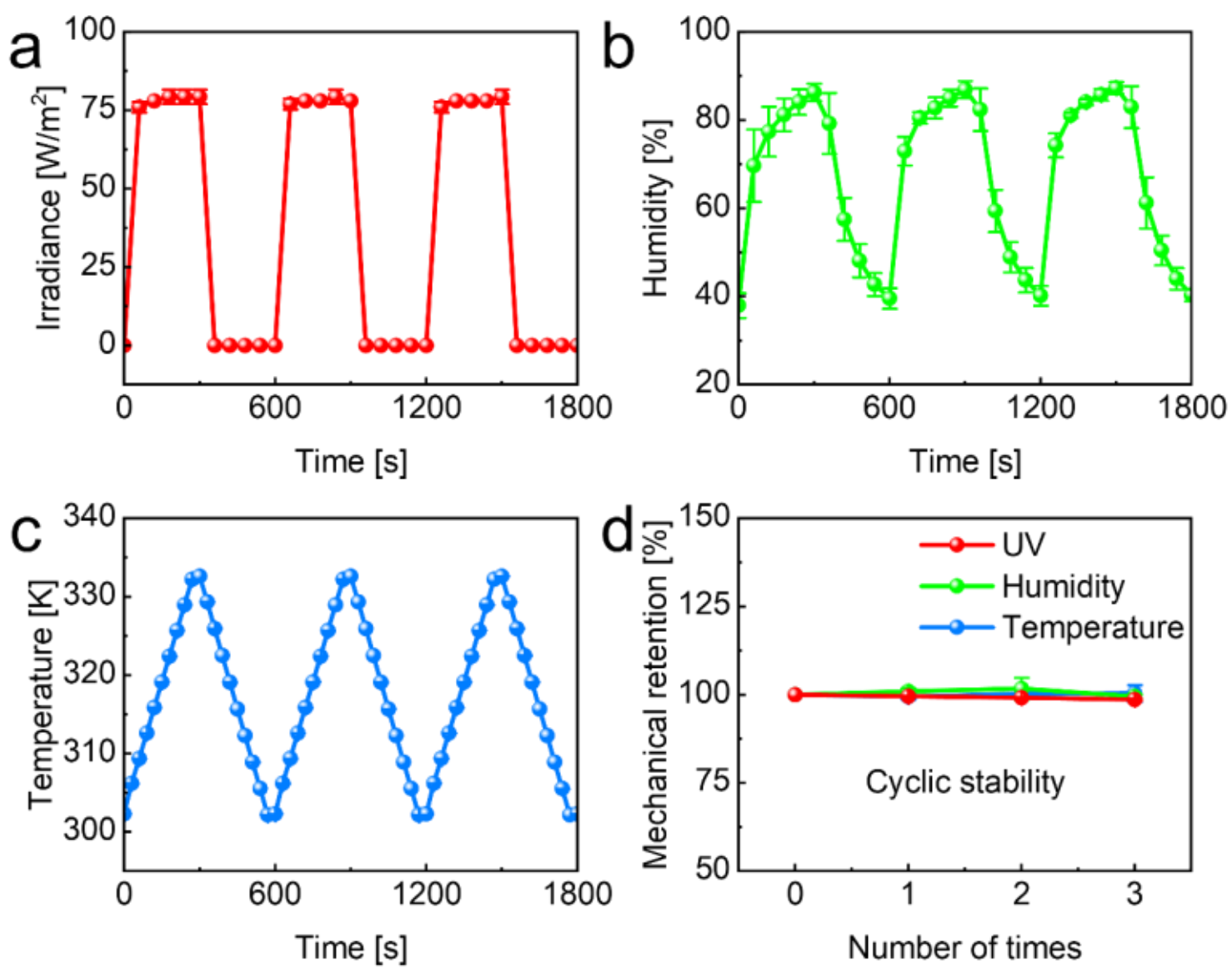

Supplementary Fig. 21 | a UV, **b** humidity, and **c** temperature cycling tests, and **d** corresponding mechanical retention of STBC.

These experiments have been added to the revised manuscript and supplementary information. (Page 17, lines 349-353; Page S14, lines 202-204)

Question 18: Quantify adhesion to representative substrates (metal, polymer, ceramic) before and after environmental exposure.

Our reply: Thank you for your valuable input on quantifying adhesion strength and environmental reliability. We perform single-lap shear tests on STBC bonded to representative substrates, including aluminum metal, ABS polymer, and alumina ceramic. The shear force increases with displacement up to the failure point and then decreases (Supplementary Fig. 28a). Comparing specimens before and after 1 day of outdoor environmental exposure, the adhesion strength of STBC varies by no more than 5% across all substrates (Supplementary Fig. 28b). This consistency reflects the preserved adhesive properties of the SSG after environmental exposure and the predominance of cohesive failure within the SSG, indicating robust interfacial bonding. STBC demonstrates good adhesion across diverse substrates, supporting effective thermal interface management. Additionally, the application of vacuum-grade silicone grease at the interface can further enhance interfacial adhesion.

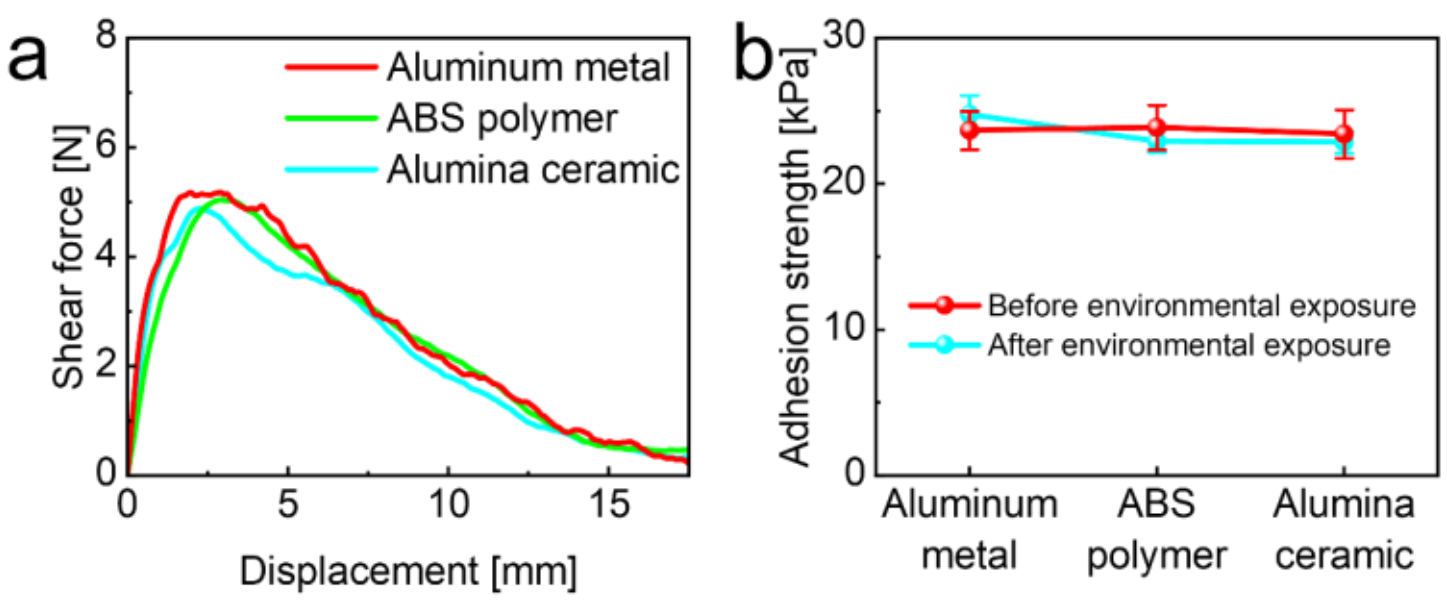

Supplementary Fig. 28 | a Shear force-displacement of STBC adhered to representative substrates, and **b** adhesion strength before and after environmental exposure under single-lap shear testing.

Relevant discussion has been included in the revised manuscript. (Page 20, lines 421-422; Page 26, lines 601-602; Page 27, line 603; Page S17, lines 236-239)

6. Life-Cycle Assessment (LCA) and Scalability

Question 19: The current functional unit ("1 m^{-3} (W m^{-1} K^{-1})$^{-1}$") is unnecessarily complex. Recalculate or at least normalize results using practical units (e.g., "1 m^2 of

coating" or "1 kg of material") for clarity.

Our reply: Thank you for your valuable suggestion. The previously used functional unit (1 m^{-3} (W m^{-1} $K^{-1})^{-1}$) is overly complex and may have impeded intuitive interpretation. To improve clarity and practical relevance, we recalculate and standardize the performance metrics using a benchmark of 1 kg of material. This approach yields concise units aligned with real-world usage. The updated standardized data are reported in Supplementary Table 9 and Fig. 51.

Supplementary Table 9 | Life cycle environmental influences of STBC and s-STBC.

Influence category	Unit	STBC	**s-STBC**
Global warming	kg CO_2 eq	1.54E+01	**1.39E+01**
Terrestrial acidification	kg SO_2 eq	6.46E-02	**5.67E-02**
Freshwater eutrophication	kg P eq	5.50E-03	**4.85E-03**
Marine eutrophication	kg N eq	2.91E-03	**2.16E-03**
Terrestrial ecotoxicity	kg 1,4-DCB	4.98E+02	**4.35E+02**
Freshwater ecotoxicity	kg 1,4-DCB	7.48E-01	**6.32E-01**
Marine ecotoxicity	kg 1,4-DCB	1.39E+00	**1.20E+00**
Human carcinogenic toxicity	kg 1,4-DCB	2.15E+00	**1.89E+00**
Human non-carcinogenic toxicity	kg 1,4-DCB	1.67E+01	**1.43E+01**
Land use	m^2a crop eq	1.20E+00	**9.57E-01**
Mineral resource scarcity	kg Cu eq	7.90E-02	**6.41E-02**
Fossil resource scarcity	kg oil eq	4.35E+00	**3.80E+00**
Water consumption	m^3	4.61E-01	**3.52E-01**

Functional unit: 1 kg of material.

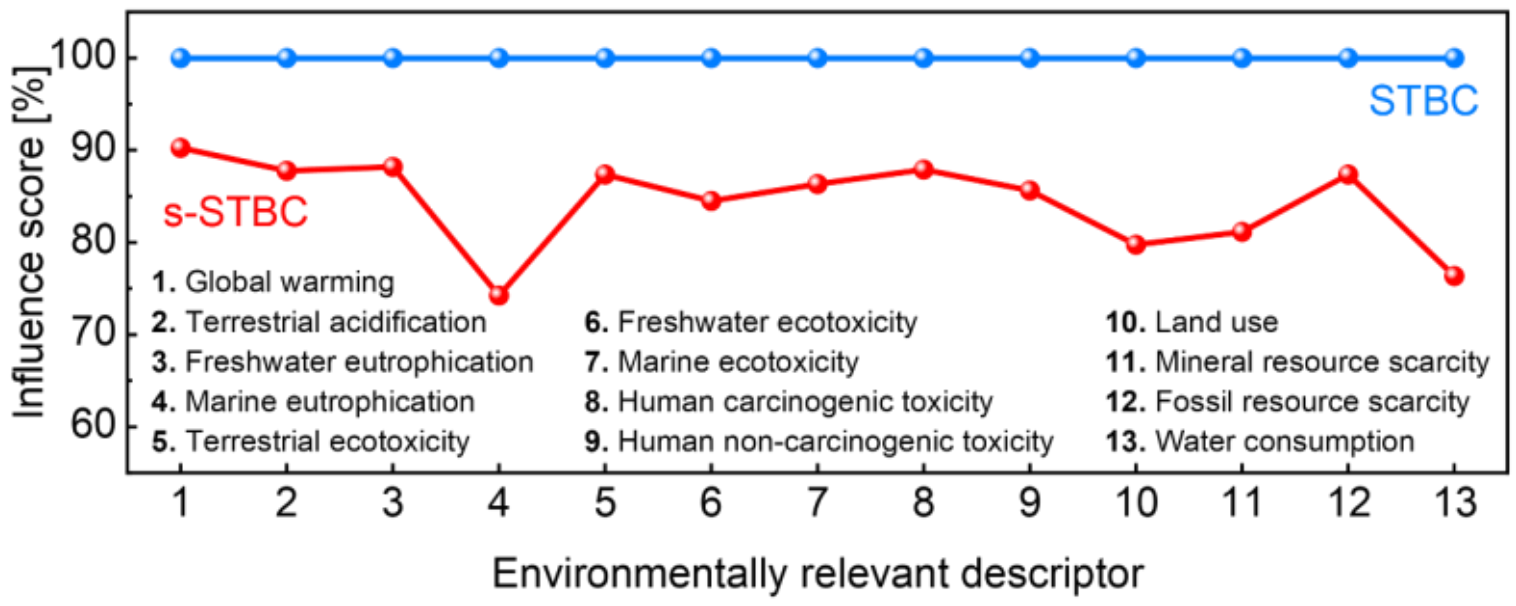

Fig. 5l | Influence scores for all environmentally relevant descriptors in STBC and s-STBC.

Relevant discussion has been incorporated into the revisions. (Page 22, line 478; Page S29, lines 325-326)

Question 20: Expand the LCA to report carbon and water footprints of key raw materials and summarize in a supplementary table.

Our reply: Thank you for your constructive recommendation. To more comprehensively present the material's sustainability profile, we expand the LCA to include both carbon and water footprints for all raw materials. The carbon footprint is quantified using the global warming metric, and the water footprint is evaluated using the water consumption indicator. The expanded LCA data are compiled in Supplementary Table 10.

Supplementary Table 10 | Life cycle environmental influences of raw materials for STBC and s-STBC.

Influence category	Section	Carbon footprint of STBC [kg CO_2 eq]	Water footprint of STBC [m^3]	Carbon footprint of s-STBC [kg CO_2 eq]	Water footprint of s-STBC [m^3]
BN	Input (Process)	7.90E+00	2.21E-01	5.72E+00	1.60E-01
TA		8.80E-03	2.91E-04	6.38E-03	2.11E-04
CNC		2.75E+00	2.12E-01	2.00E+00	1.53E-01

Influence category	Section	Carbon footprint of STBC [kg CO_2 eq]	Water footprint of STBC [m^3]	Carbon footprint of s-STBC [kg CO_2 eq]	Water footprint of s-STBC [m^3]
HSO		3.26E+00	2.22E-02	5.05E+00	3.44E-02
BA		7.99E-03	1.19E-04	1.24E-02	1.85E-04
OA		1.09E-03	9.79E-06	1.70E-03	1.52E-05
Ethanol		8.02E-02	3.51E-04	5.81E-02	2.54E-04
Water		9.59E-04	1.93E-03	6.95E-04	1.40E-03
Electricity		4.76E-01	1.25E-03	3.47E-01	9.09E-04
Wastewater	Output (Process)	5.70E-04	-1.86E-03	4.13E-04	-1.35E-03
Ethanol	Input (Recycle)	4.81E-01	2.11E-03	3.48E-01	1.53E-03
Water		9.59E-04	1.93E-03	6.95E-04	1.40E-03
Electricity		4.44E-01	1.16E-03	3.22E-01	8.42E-04
Wastewater	Output (Recycle)	5.70E-04	-1.86E-03	4.13E-04	-1.35E-03
**Total**		**1.54E+01**	**4.61E-01**	**1.39E+01**	**3.52E-01**

Carbon and water footprints are quantified using the global warming and water consumption indicators, respectively.

The corresponding discussion has been added to the revised manuscript and supplementary information. (Page 22, line 478; Page S30, lines 327-330)

Question 21: If feasible, include a service-life estimate (accelerated aging or FMEA) to strengthen the durability argument.

Our reply: Thank you for your valuable suggestion, which strengthens our durability assessment. To rigorously support the material's reliability in practical applications, we supplement our work with accelerated aging under diverse physical stimuli, systematically simulating complex outdoor extreme environments.

In water-stability tests, STBC retains 99-101% of its mass, dimensional, and mechanical properties after up to 48 h of static immersion and under cyclic immersion-drying (Supplementary Fig. 18b). In composite cyclic aging tests simulating outdoor light, humidity, and temperature fluctuations, we apply cycles of 365 nm UV irradiation (0-80 W m^{-2}), 40-85% relative humidity, and temperatures from 303-333 K (Supplementary Fig. 21a-c). Post-test storage modulus deviations are within 2% of initial values (Supplementary Fig. 21d). During long-term high-temperature aging at 393 K, STBC's storage modulus remains stable, indicating negligible thermal degradation (Fig. 4d). Furthermore, 24-month natural aging confirms no significant degradation of the SSG matrix's shear-stiffening core properties (Fig. 1d). Across more than 100 thermal cycling tests simulating real-world heat-dissipation scenarios, s-STBC exhibited no measurable decline in cooling efficiency (Fig. 5f).

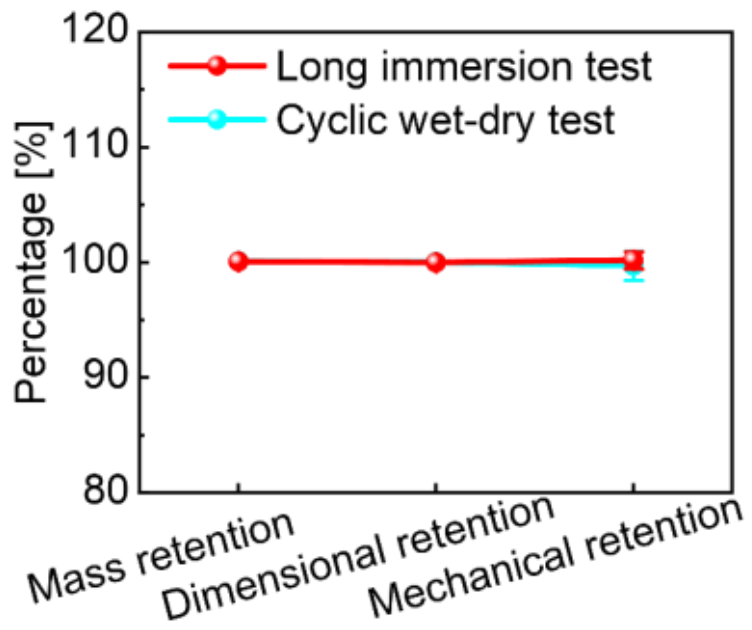

Supplementary Fig. 18b | Mass, dimensional, and mechanical retention of STBC after prolonged immersion and cyclic wet-dry tests.

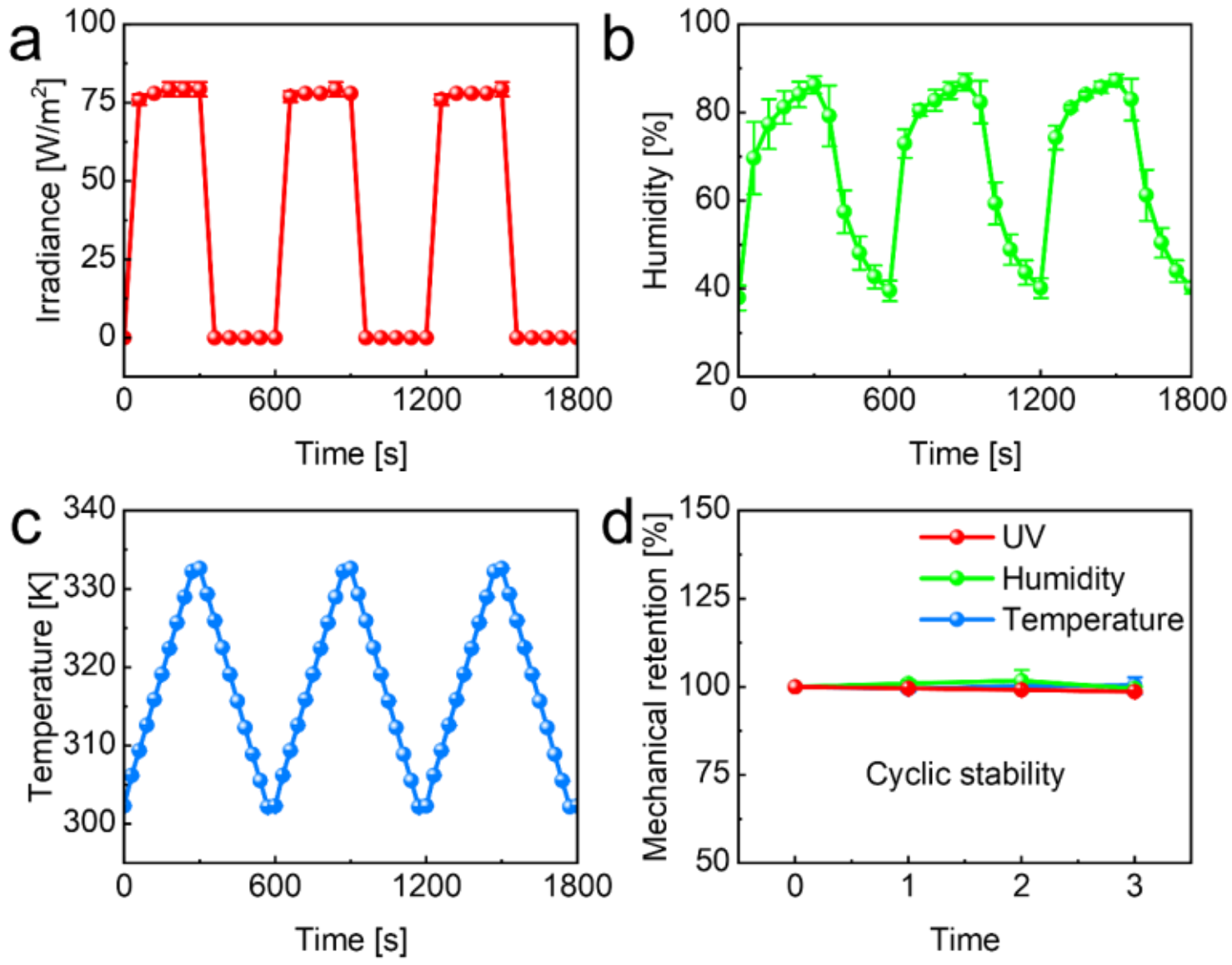

Supplementary Fig. 21 | a UV, **b** humidity, and **c** temperature cycling tests, and **d** corresponding mechanical retention of STBC.

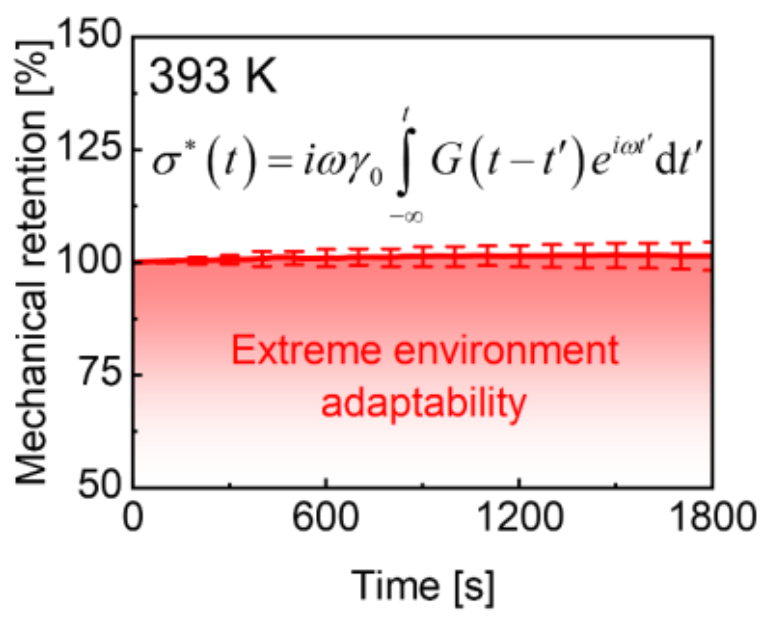

Fig. 4d | Aging resistance of STBC.

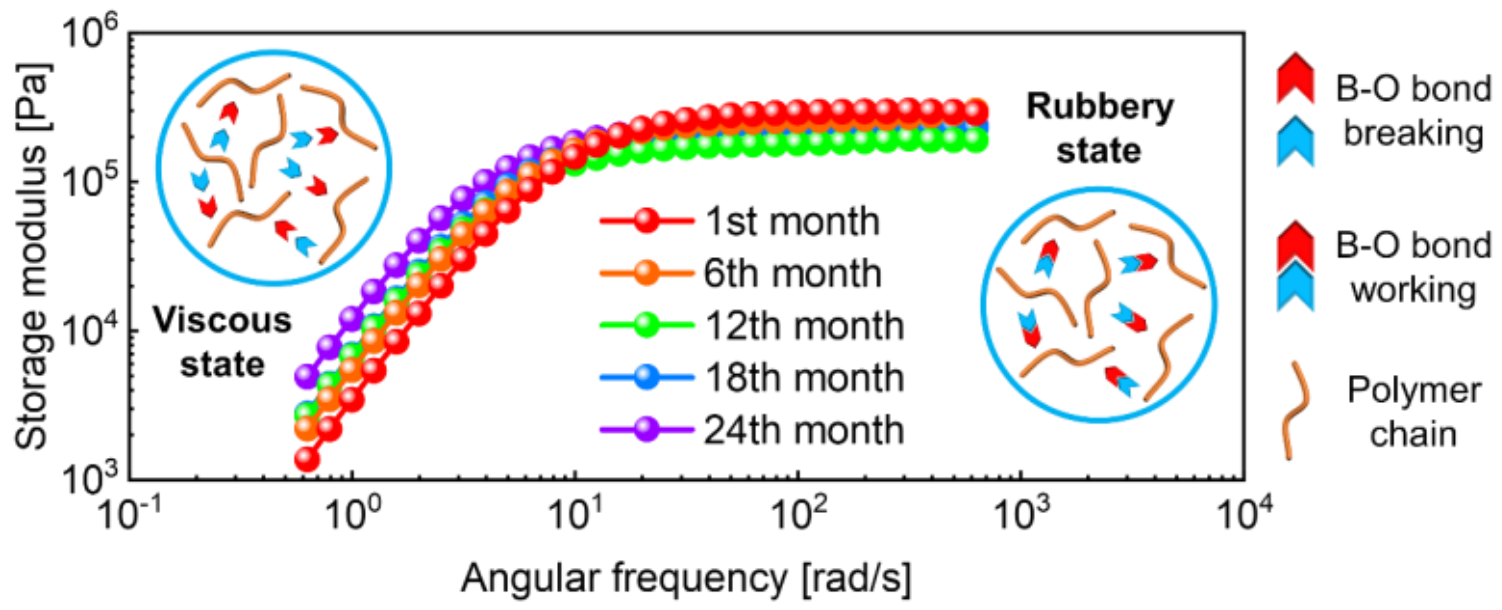

Fig. 1d | Intelligent tunable stiffness and long-term stability of SSG.

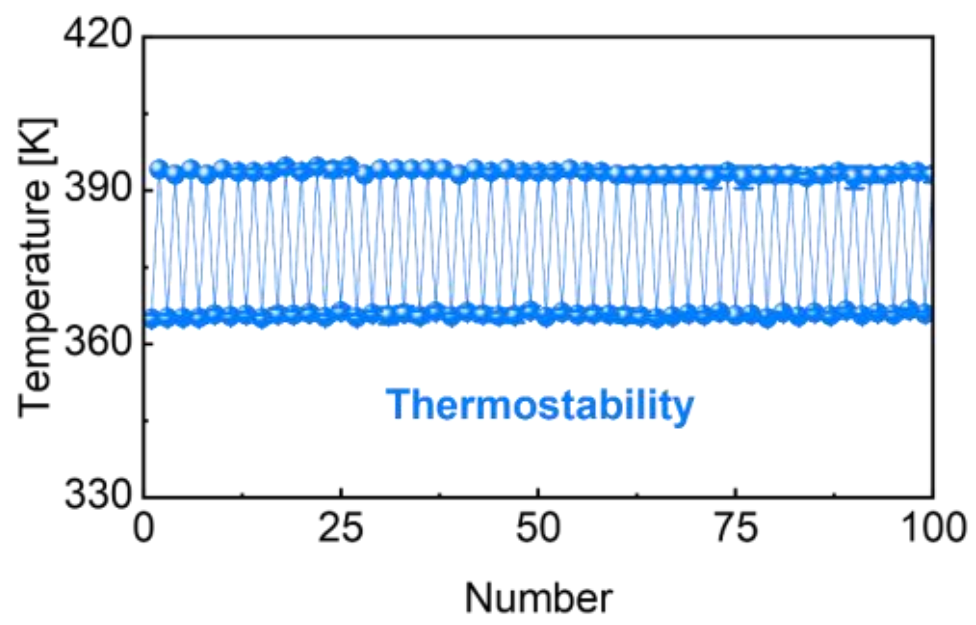

Fig. 5f | Thermal stability of heater-integrated passive insulators under 1 sun irradiation.

These multidimensional accelerated aging and long-term stability evaluations comprehensively validate the outstanding durability of STBC under humidity, light exposure, temperature cycling, prolonged high temperatures, and repeated use, providing strong experimental support for long-term outdoor service.

Detailed experimental parameters, procedures, and performance-change curves for each accelerated aging test are provided in the revised manuscript and supplementary information. (Page 6, line 112; Page 7, lines 140-142; Page 14, lines 303-306; Page 17, lines 348-353; Page 19, lines 397-398; Page 21, lines 445-447)

Question 22: For industrial feasibility, discuss scalability of the fabrication method (e.g., sheet size, yield, energy consumption). A simple production-scale simulation (~1 ton yr^{-1}) and associated cost or energy estimates would add practical relevance.

Our reply: Thank you for your valuable suggestion regarding industrial applicability. The aqueous-phase directed filtration self-assembly process used in this work is inherently amenable to large-scale manufacturing. By enlarging the filtration mold (e.g., scalable to 2 m × 2 m) and increasing the preparation volumes of SSG and filtration slurry, batch yields can exceed 1kg. Based on laboratory-scale techno-economic estimates, producing 1 ton of STBC requires approximately 1000 kWh of energy and incurs a cost of about USD 40000. In industrial practice, transitioning to continuous processing, centralizing raw-material procurement, and minimizing process losses are expected to further reduce both energy demand and unit cost.

Relevant details have been added to the supplementary information to underscore the

technology's practical value. (Page 22, lines 478-480)

Minor Comments:

Question 23: Define the atmospheric transparency window (8–13 μm) and emphasize why emissivity in this range is crucial for passive radiative cooling.

Our reply: Thank you for your valuable suggestions on clarifying the atmospheric transparency window and its role in passive radiative cooling. The atmospheric transparency window at 8-13 μm is the mid-infrared range in which the atmosphere exhibits minimal absorption and very high transmittance, allowing thermal radiation to pass through to outer space with negligible attenuation. Passive radiative cooling relies on materials that emit strongly within this window to efficiently reject heat to the sky while minimizing reabsorption and back-reflection by the atmosphere. The high infrared emissivity of STBC in this band satisfies this fundamental requirement.

We have supplemented the manuscript with the relevant definitions and mechanistic explanations. (Page S2, lines 32-36)

Question 24: Clarify how optical spectra were measured at 120 °C (instrumentation, calibration).

Our reply: Thank you for highlighting the need for detailed spectral measurement protocols. Solar reflectance over 0.3-2.5 μm is measured using a Shimadzu UV-3600 UV-vis-NIR spectrophotometer. Prior to testing, the instrument is calibrated with a high-reflectance barium sulfate standard to eliminate errors from ambient light and baseline drift. Mid-infrared emissivity in the 8-13 μm atmospheric window is measured using a Thermo Fisher Nicolet iS50 Fourier-transform infrared spectrometer, calibrated against a high-reflectance gold mirror to ensure accurate emissivity determination. For measurements at 393 K, both instruments are equipped with temperature-controlled chambers. The system is preheated to 393 K and stabilized for 30 min before measurements. Spectral scanning commences only after the sample reaches thermal equilibrium, with temperature deviation strictly controlled within ±1 K to preclude thermal fluctuation artifacts. All procedures and calibrations conform to relevant

academic standards.

Detailed instrument parameters and calibration steps have been included in the supplementary information to facilitate replication and verification. (Page S2, lines 28-31, 42-43; Page S3, lines 44-50)

Question 25: Add uncertainty bars for all thermal and mechanical plots.

Our reply: Thank you for highlighting the importance of data presentation standards. To improve rigor, we have added uncertainty bars to all relevant plots throughout the article and Supplementary Information. Data are presented as the mean ± SD from n = 3 independent samples. In addition, we perform error analyses for all supplementary experiments to ensure reproducibility. These updates enhance the scientific robustness of our data presentation and enable readers to more accurately assess result reliability.

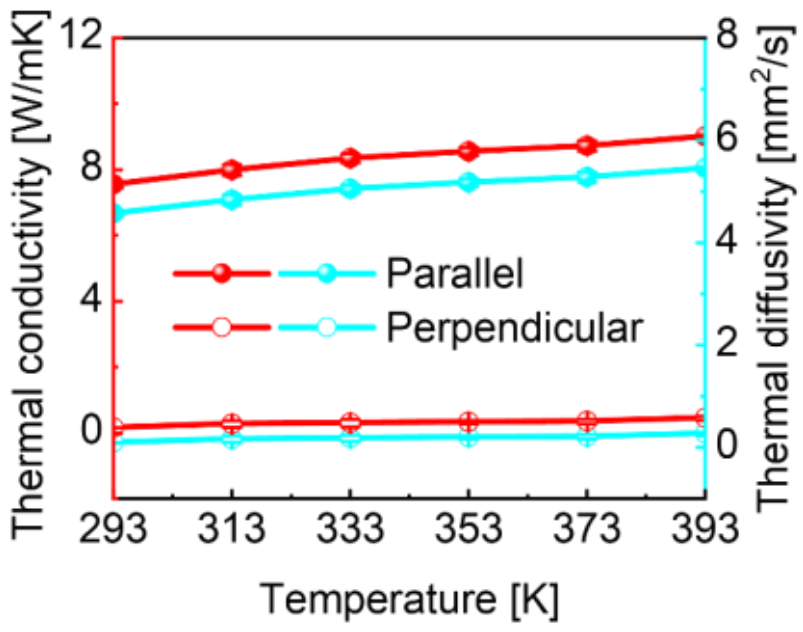

Fig. 2b | Temperature-dependent profiles of STBC in the parallel and perpendicular directions.

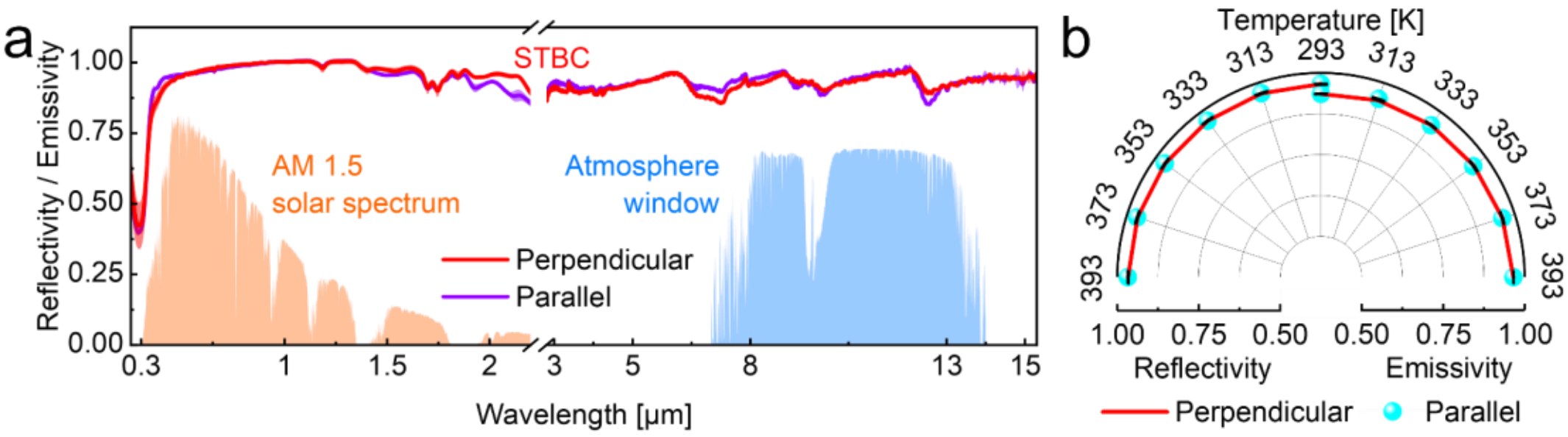

Fig. 3 | a Reflectivity and emissivity of STBC measured in the parallel and perpendicular directions, and **b** their temperature-insensitive polar plots.

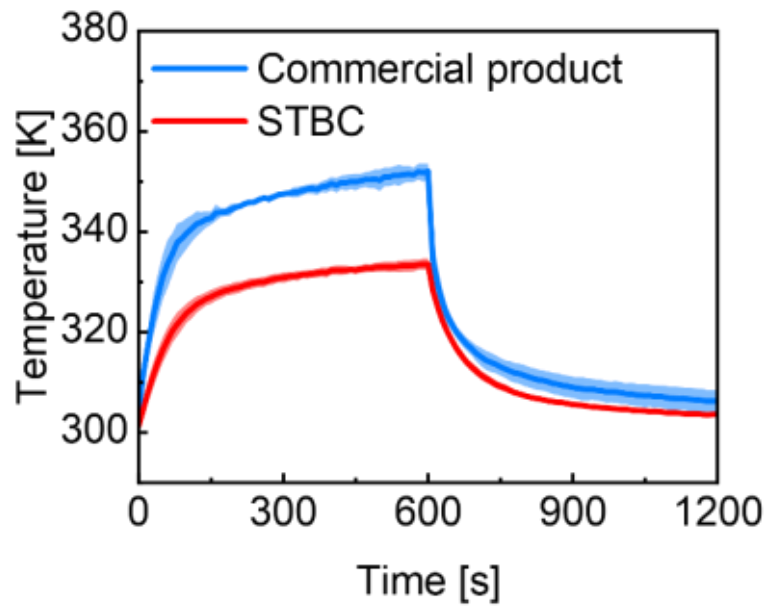

Fig. 3d | Time-dependent temperature comparisons between a commercial radiative cooling product and STBC under 1 sun exposure.

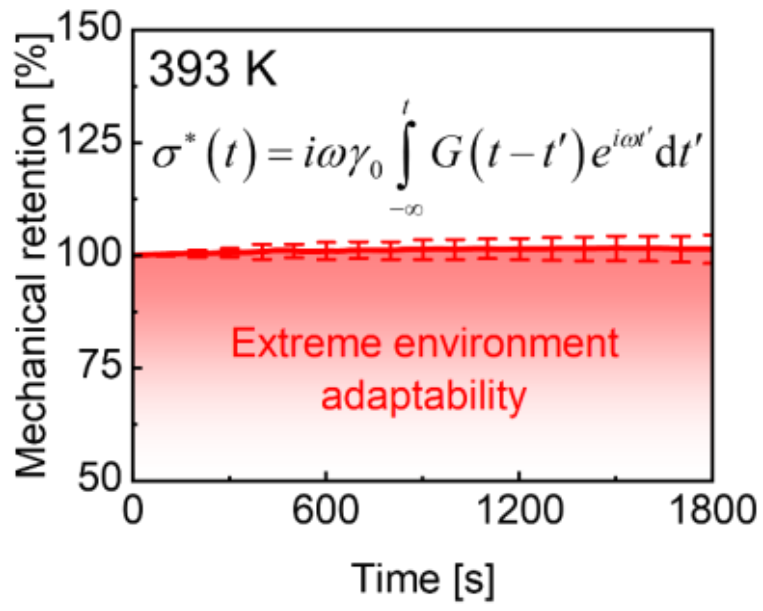

Fig. 4d | Aging resistance of STBC.

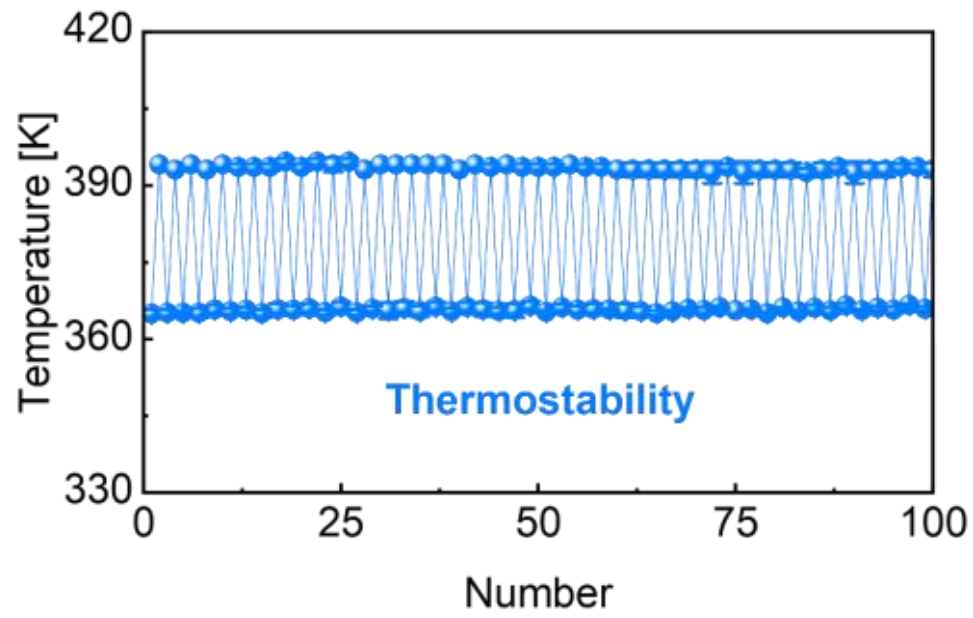

Fig. 5f | Thermal stability of heater-integrated passive insulators under 1 sun irradiation.

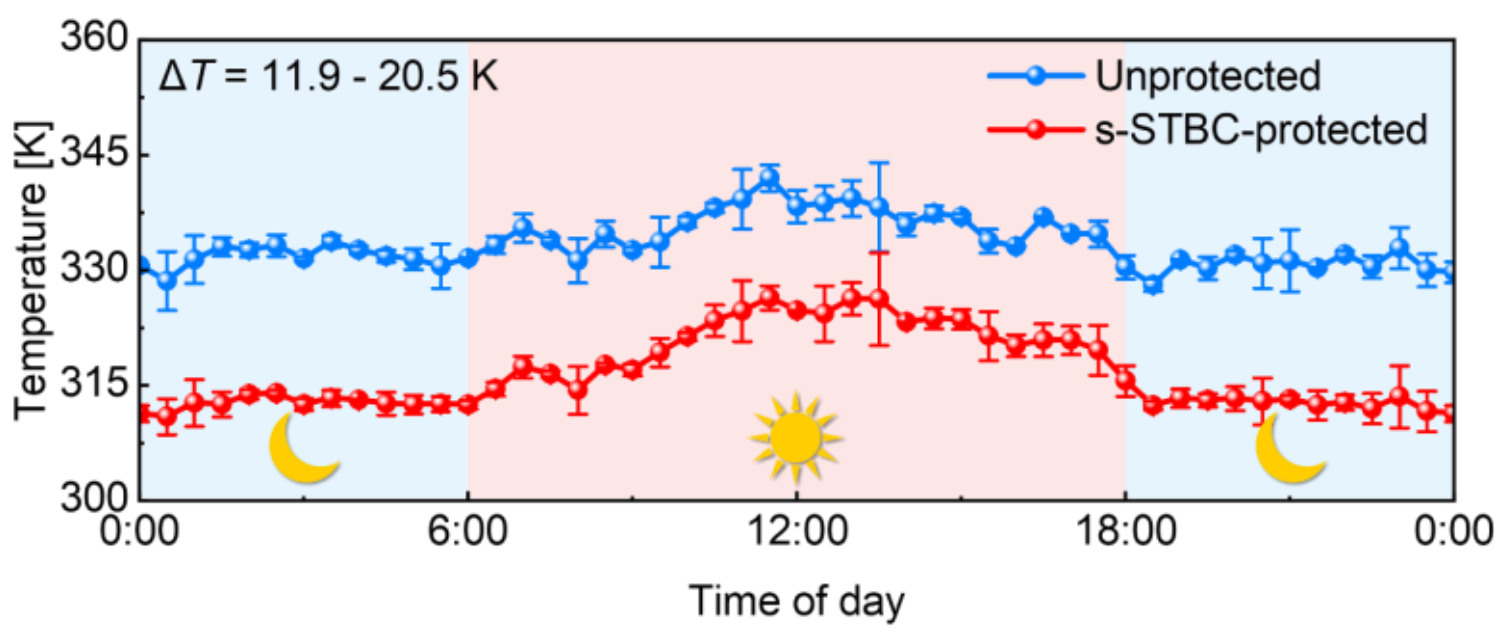

Fig. 5k | Temperature evolution of unprotected and s-STBC-protected heaters during

outdoor passive cooling in Hefei, China.

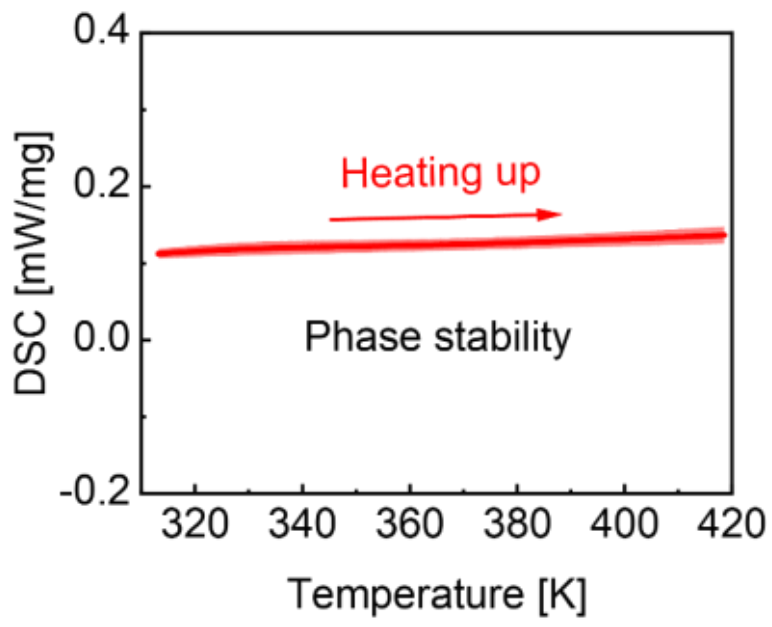

Supplementary Fig. 5 | Temperature-dependent DSC profile of STBC.

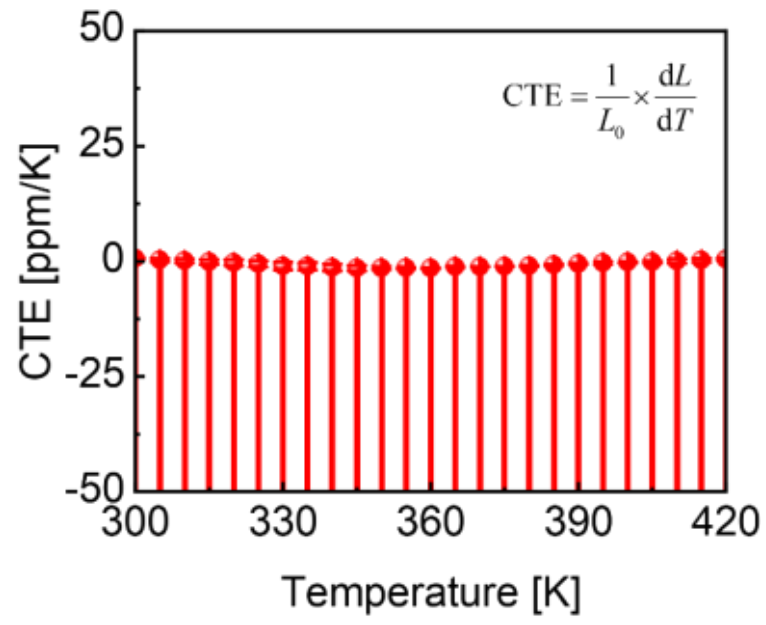

Supplementary Fig. 6 | Coefficients of thermal expansion for STBC from 300 to 420 K.

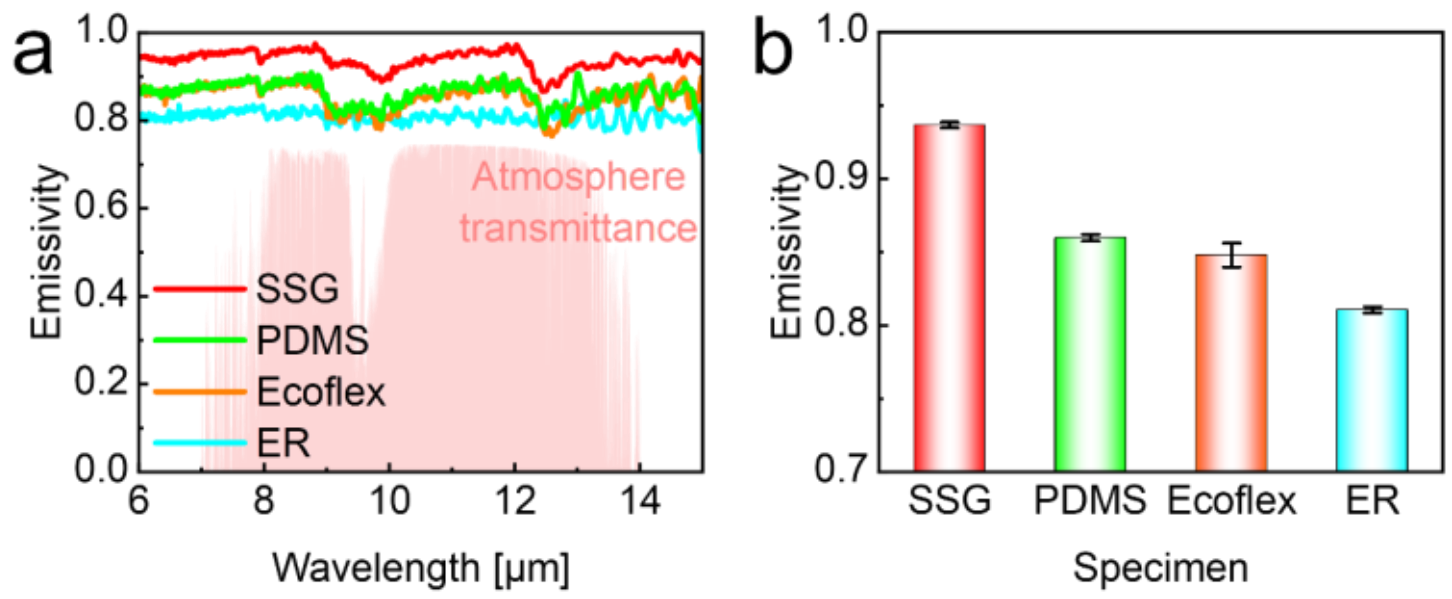

Supplementary Fig. 12 | a Emissivity-wavelength spectra and **b** emissivity comparison of SSG versus high-molecular-weight PDMS, Ecoflex, and epoxy resin (ER) engineering polymers.

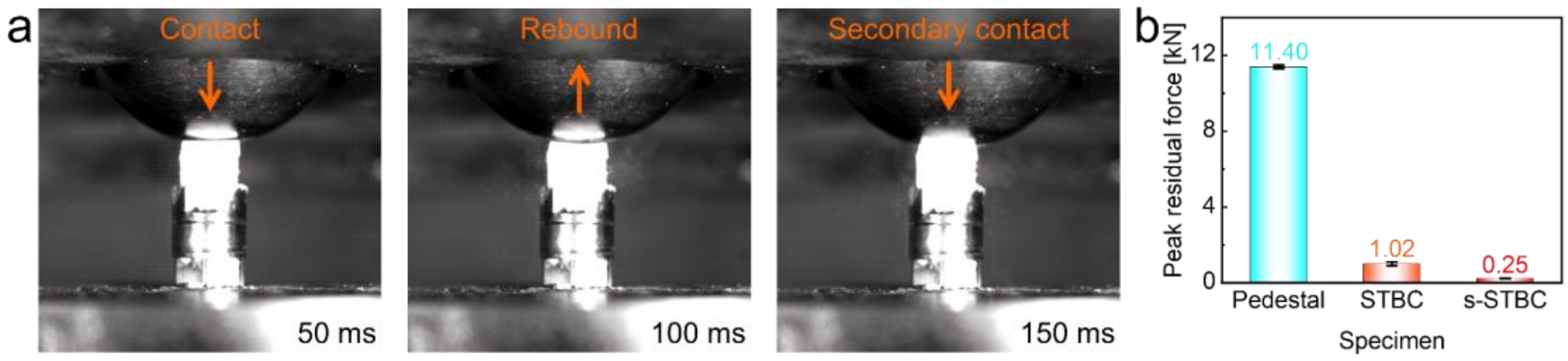

Supplementary Fig. 32 | a 3.2 kg impact sequence captured by high-speed imaging and **b** peak residual force of the pedestal, STBC, and s-STBC.

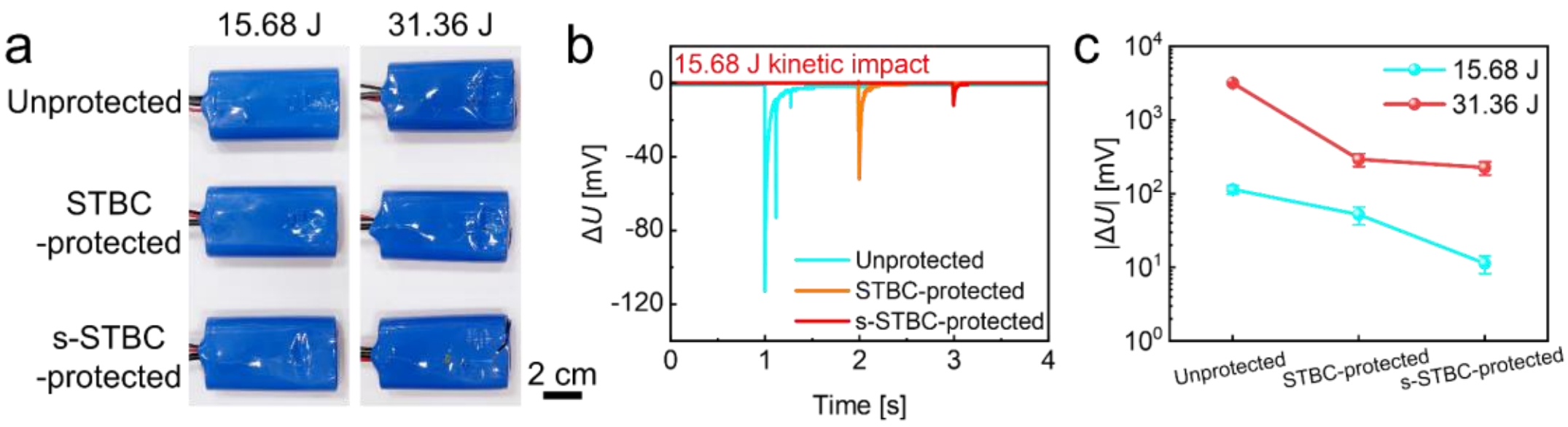

Supplementary Fig. 33 | a Damaged states, **b** temporal voltage traces, and **c** comparisons of voltage change for unprotected, STBC-protected, and s-STBC-protected batteries.

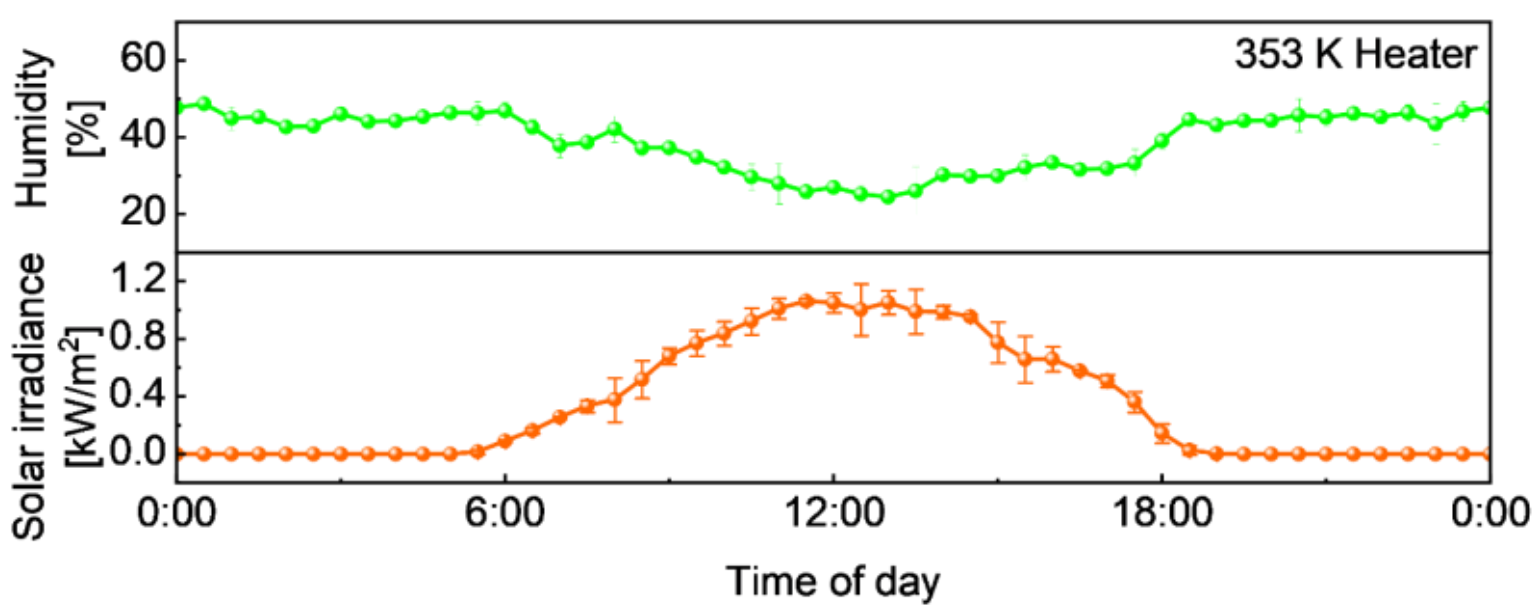

Supplementary Fig. 34 | Diurnal profiles of solar irradiance and ambient humidity in Hefei, China.

Question 26: Streamline redundant figure captions in the Supplementary Information.

Our reply: Thank you for identifying the redundancy in the supplementary information figure captions. We have comprehensively revised and streamlined all captions to emphasize key information and improve readability.

Specifically, redundant and nonessential descriptive text has been removed, and the general description of error analysis has been consolidated into the Methods section for a unified presentation (Page 28, line 632). In accordance with *Nature Communications* formatting, repeated descriptions of specimens, experimental conditions, and other recurring elements have been standardized and simplified.

After revision, each caption concisely and accurately summarizes the core content of its corresponding figure, substantially enhancing readability.

Question 27: Correct typographical errors (e.g., "brick-and-motar" → "brick-and-mortar").

Our reply: We appreciate your careful review of typographical details. We have thoroughly checked and corrected spelling and grammar across the manuscript and figure labels. All corrections are now incorporated into the revised version.

Question 28: Briefly explain in Fig. 3j why equatorial regions show stronger simulated cooling; consider adding rainfall/dust-condition simulations to support robustness claims.

Our reply: Thank you for your interest in cooling efficiency. The equatorial belt in Fig. 3j shows stronger cooling energy savings primarily because of substantially higher solar irradiance in that region. Under these conditions, STBC can reject more heat via passive radiative cooling, yielding more pronounced energy savings (Supplementary Table 3). To assess robustness for outdoor applications, we conduct tests under simulated rainfall and dust exposure. Following dust deposition, simulated rainfall rapidly removes surface contaminants without residual fouling (Supplementary Fig. 18c). Combined with the material's strong hydrophilicity evidenced by a water contact angle of 31.35° (Supplementary Fig. 18d), rainwater efficiently washes away particulates, preserving optical properties and cooling efficiency in complex outdoor environments.

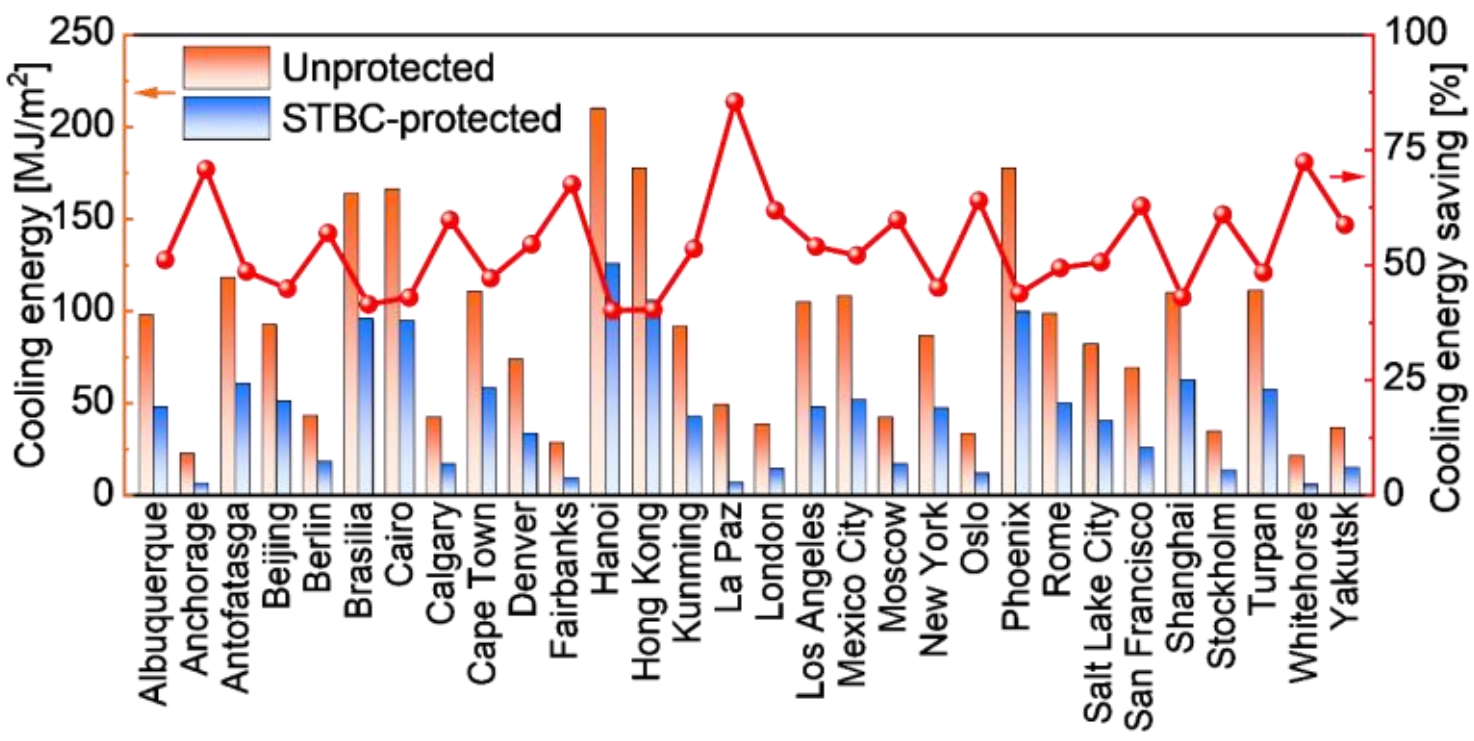

Fig. 3j | City-scale energy-efficiency gains for STBC-protected building models worldwide.

Supplementary Table 3 | Energy-efficiency outcomes for global cities with and without STBC protection, evaluated using EnergyPlus simulations.

City	Unprotected cooling energy [MJ m^{-2}]	STBC-protected cooling energy [MJ m^{-2}]	Cooling energy saving [%]
Albuquerque	98.32	48.05	51.13
Anchorage	22.85	6.67	70.81
Antofatasga	118.45	60.87	48.61
Beijing	92.87	51.18	44.89
Berlin	43.37	18.67	56.95
Brasilia	164.03	96.00	41.47
Cairo	166.43	94.90	42.98
Calgary	42.68	17.15	59.82
Cape Town	110.62	58.47	47.14
Denver	73.87	33.60	54.51
Fairbanks	28.82	9.35	67.56
Hanoi	210.15	125.83	40.12
Hong Kong	177.63	105.97	40.34
Kunming	92.05	42.77	53.54
La Paz	49.47	7.18	85.49
London	38.82	14.80	61.88

City	Unprotected cooling energy [MJ m^{-2}]	STBC-protected cooling energy [MJ m^{-2}]	Cooling energy saving [%]
Los Angeles	105.08	48.30	54.04
Mexico City	108.50	51.95	52.12
Moscow	42.75	17.18	59.81
New York	86.53	47.50	45.11
Oslo	33.50	12.05	64.03
Phoenix	177.58	99.70	43.86
Rome	98.85	50.05	49.37
Salt Lake City	82.32	40.63	50.64
San Francisco	69.40	25.78	62.85
Shanghai	110.15	62.77	43.01
Stockholm	34.98	13.65	60.98
Turpan	111.17	57.37	48.39
Whitehorse	21.92	6.05	72.40
Yakutsk	36.68	15.13	58.75

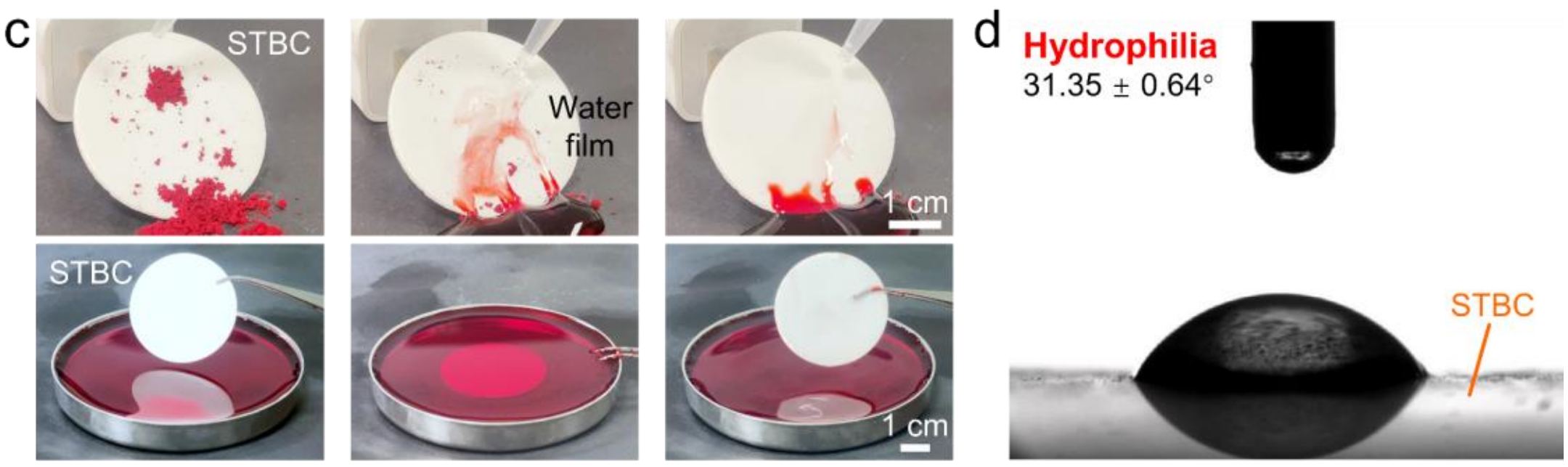

Supplementary Fig. 18 | c Self-cleaning and **d** hydrophilic properties of STBC.

Relevant experimental details have been provided in the revision and supplementary information. (Page 12, lines 244-246; Page 14, line 306; Page 15, lines 307-310; Page S12, lines 187-188; Page S13, line 191)

We thank you for your meticulous review, which has significantly improved the academic quality of the manuscript.

Reply to Reviewer #3

Comment: Some revised suggestion for this manuscript as following.

Our reply: Thank you for your professional review. Your suggestions regarding standardization and clarity are crucial for improving the manuscript's quality. All revisions have been implemented in both the manuscript and supplementary information. By standardizing data formats, optimizing the presentation of figures and tables, refining the analysis of structural parameters, and improving language quality, we have strengthened the manuscript's attention to detail and adherence to academic standards, ensuring consistency with the journal's requirements. We appreciate your meticulous review and these changes have materially improved the work's presentation and academic integrity.

Question 1: Information of the same category (temperature quantification metrics) should theoretically adhere to a unified format standard.

Our reply: Thank you for highlighting the need for consistent data formatting. Consistency within each category is essential for professionalism and readability. Following your suggestion, we systematically review and correct all temperature-related quantitative metrics across the manuscript. All temperature values are now reported uniformly in Kelvin (K) according to SI conventions, with consistent numerical precision and formatting. The same standardized format has also been applied throughout the supplementary experiments.

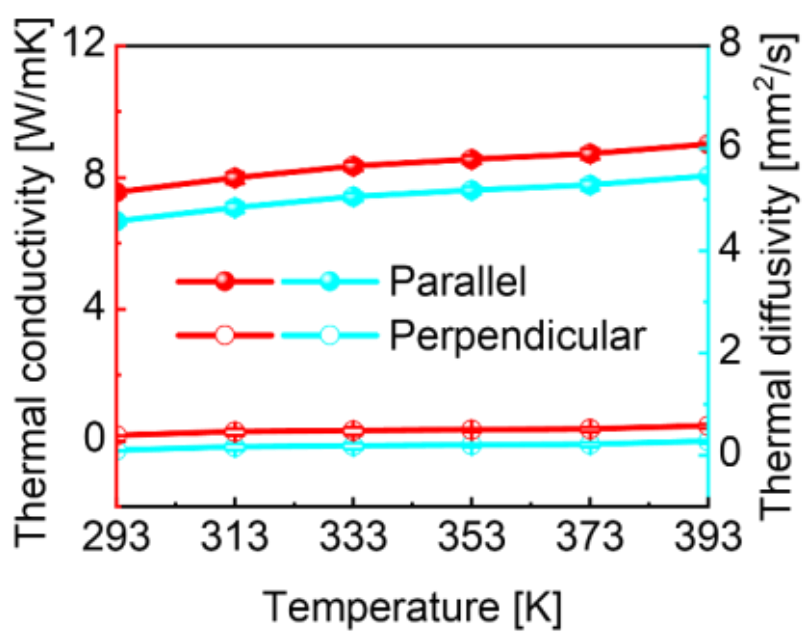

Fig. 2b | Temperature-dependent profiles of STBC in the parallel and perpendicular directions.

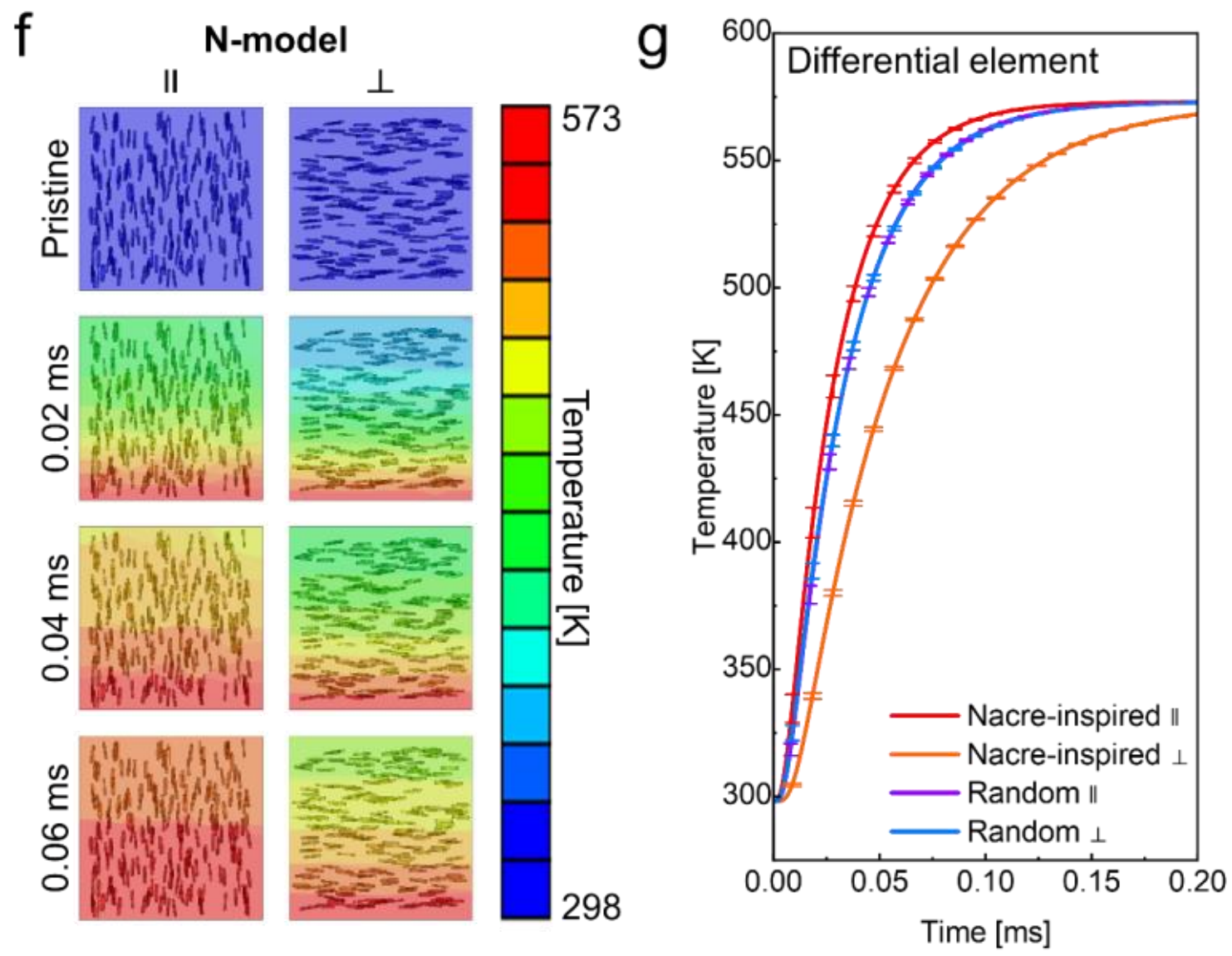

Fig. 2 | f Time-resolved temperature maps of the N-model in the parallel and perpendicular directions. **g** Temperature-time traces at differential elements for nacre-inspired and random structures in both directions.

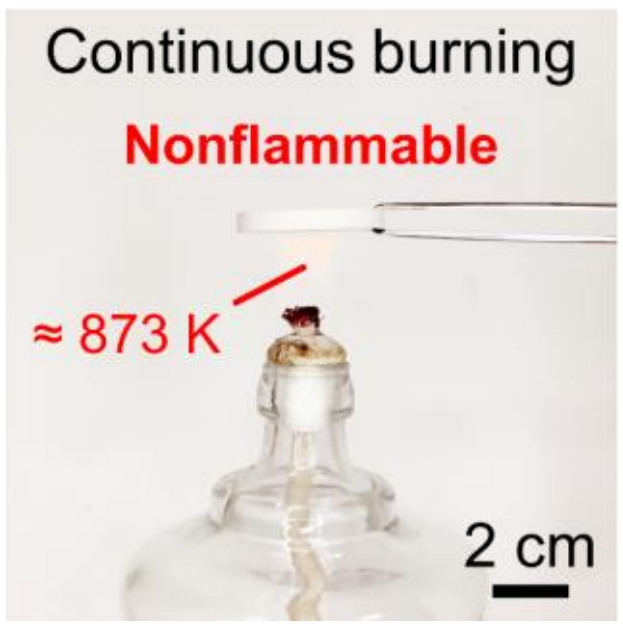

Fig. 2h | Flame-impact resistance of STBC.

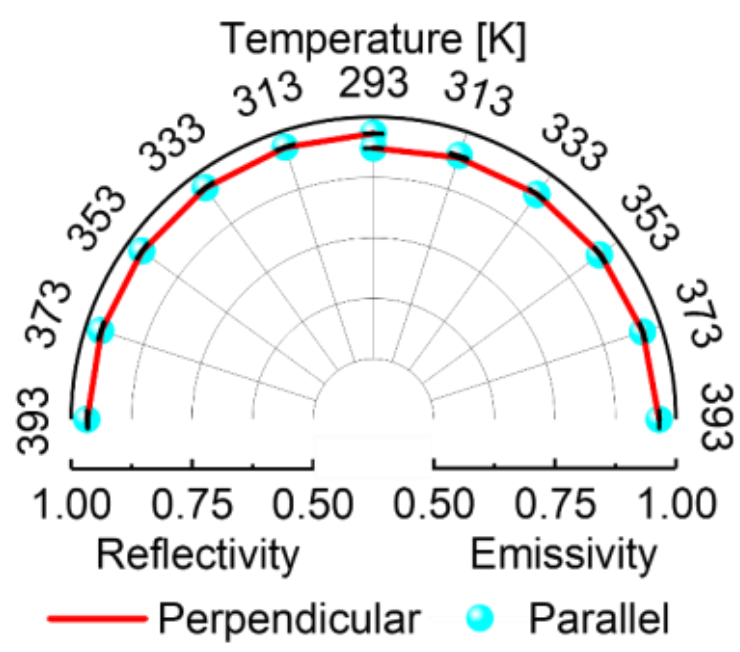

Fig. 3b | Temperature-insensitive polar plots of STBC measured in the parallel and perpendicular directions.

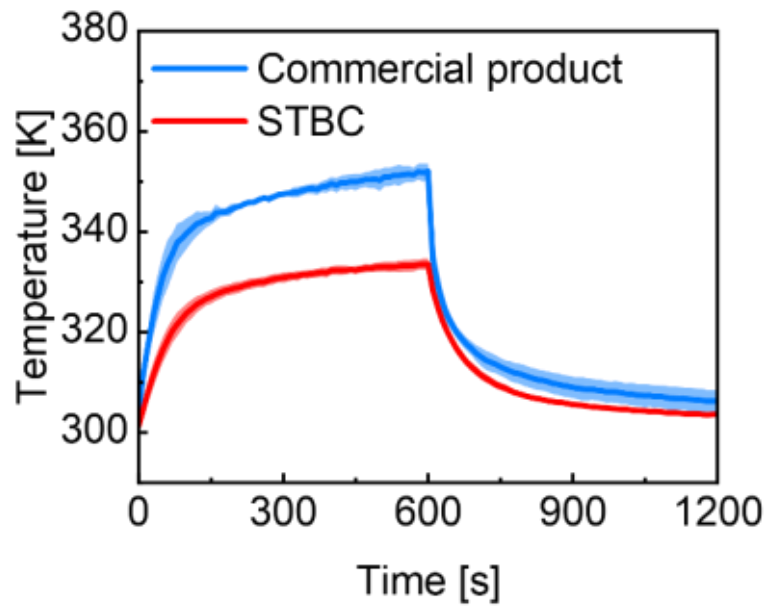

Fig. 3d | Time-dependent temperature comparisons between a commercial radiative cooling product and STBC under 1 sun exposure.

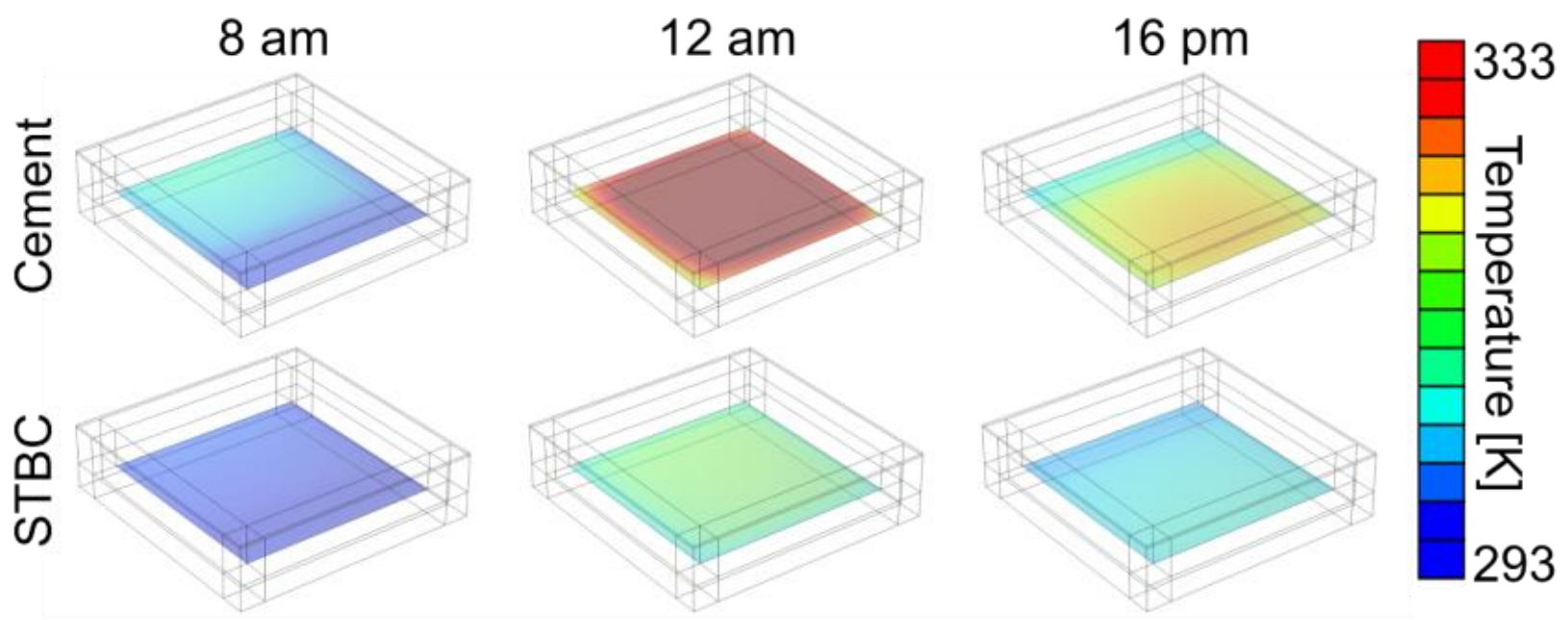

Fig. 3f | Finite-element temperature maps of conventional cement building material versus STBC.

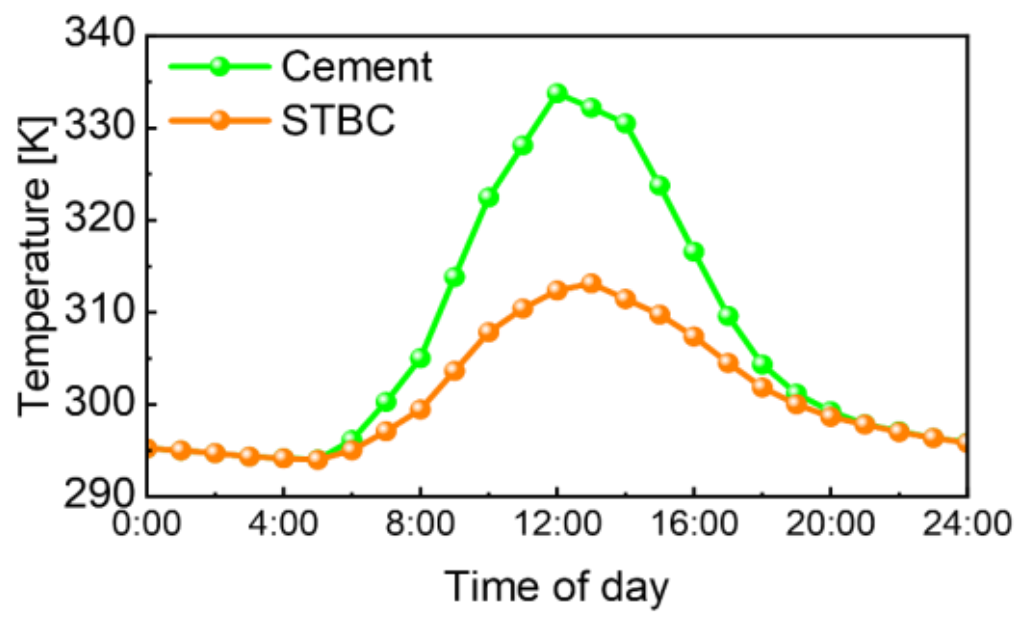

Fig. 3h | Temperature of cement and STBC over a simulated 1-day period in Hefei, China.

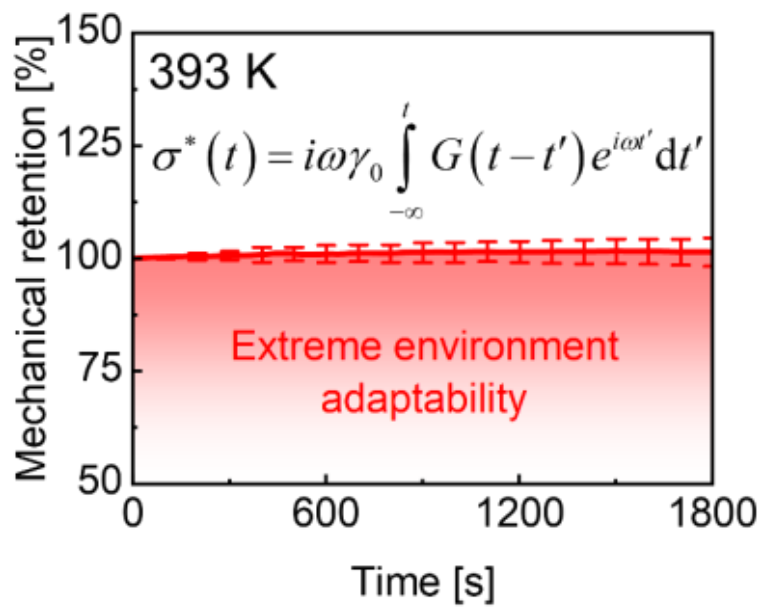

Fig. 4d | Aging resistance of STBC.

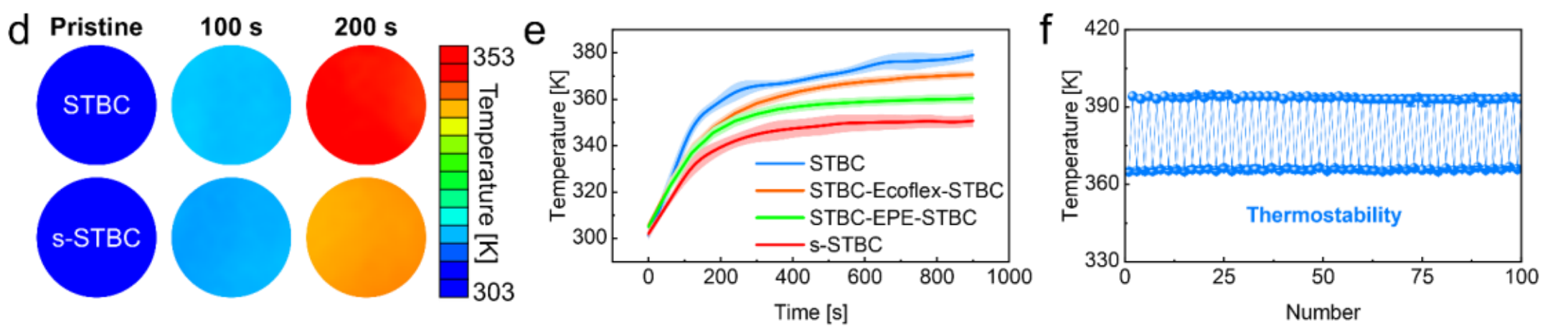

Fig. 5 | d Infrared images, **e** temporal temperature profiles and **f** thermal stability of heater-integrated passive insulators under 1 sun irradiation.

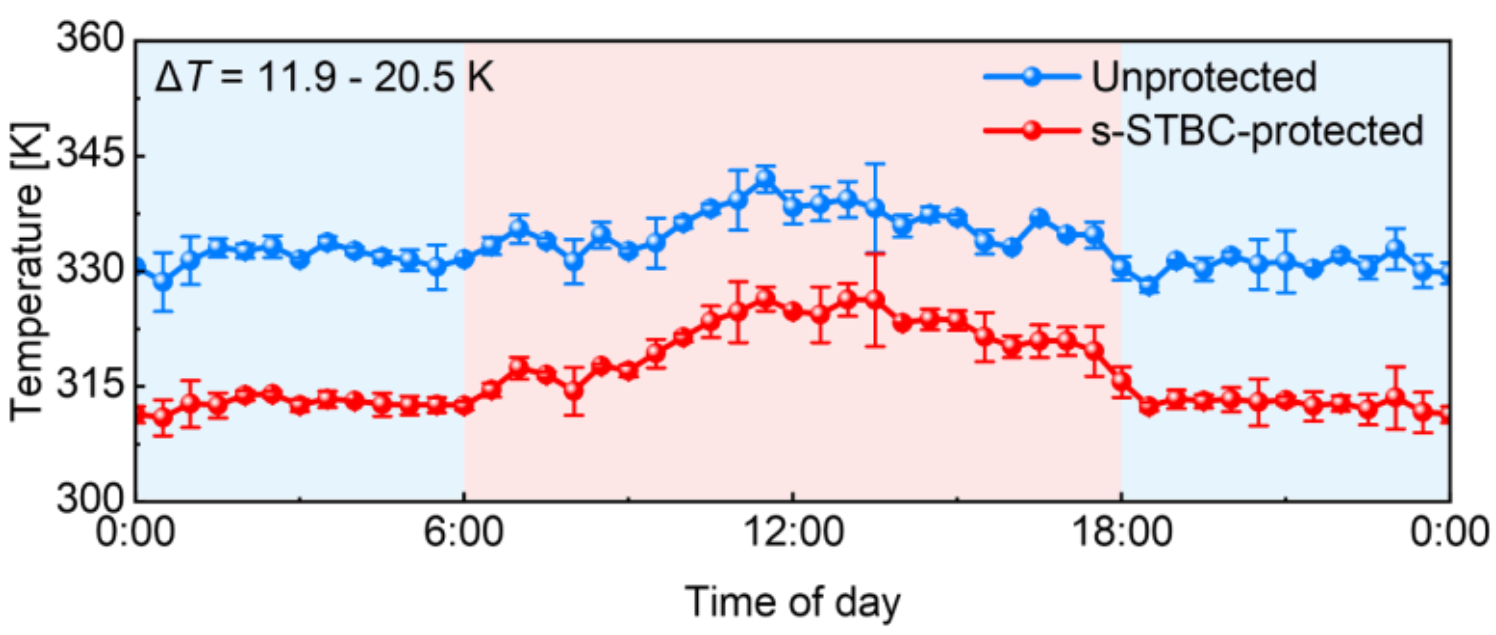

Fig. 5k | Temperature evolution of unprotected and s-STBC-protected heaters during outdoor passive cooling in Hefei, China.

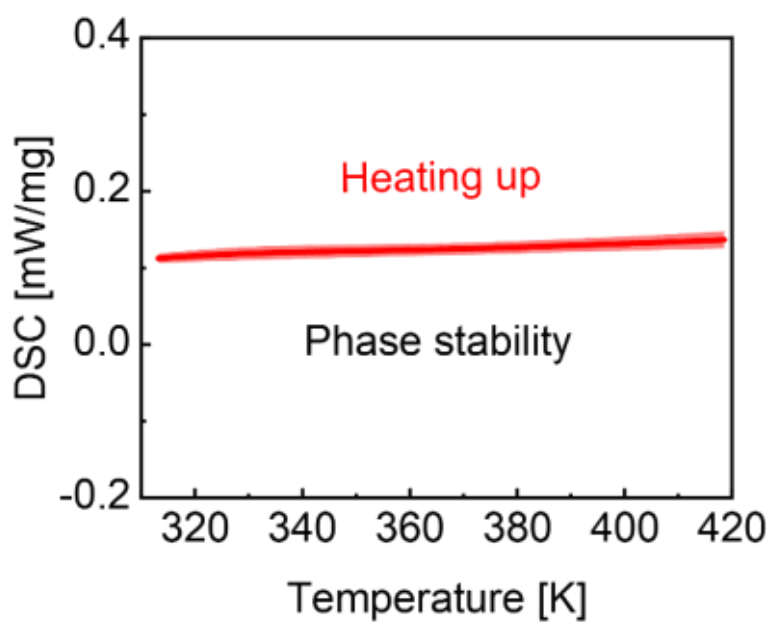

Supplementary Fig. 5 | Temperature-dependent DSC profile of STBC.

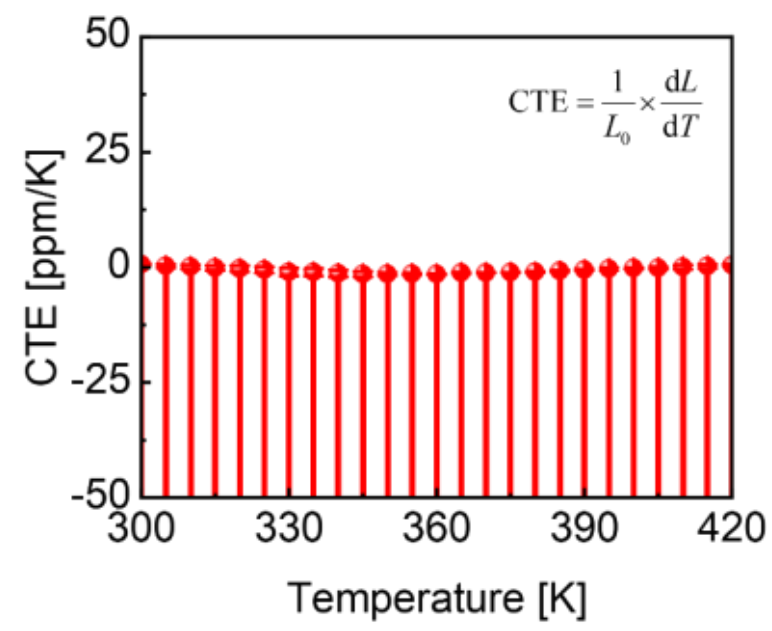

Supplementary Fig. 6 | Coefficients of thermal expansion for STBC from 300 to 420 K.

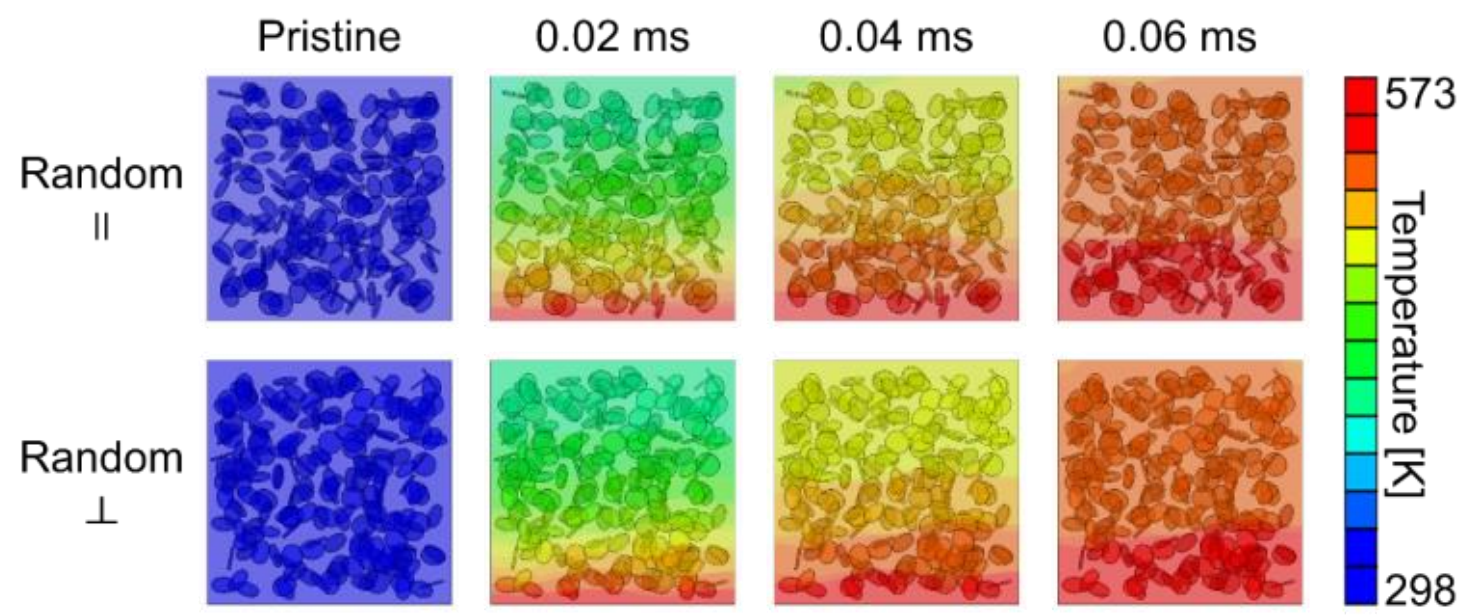

Supplementary Fig. 7 | Temporal temperature maps of the R-model in the parallel and perpendicular directions.

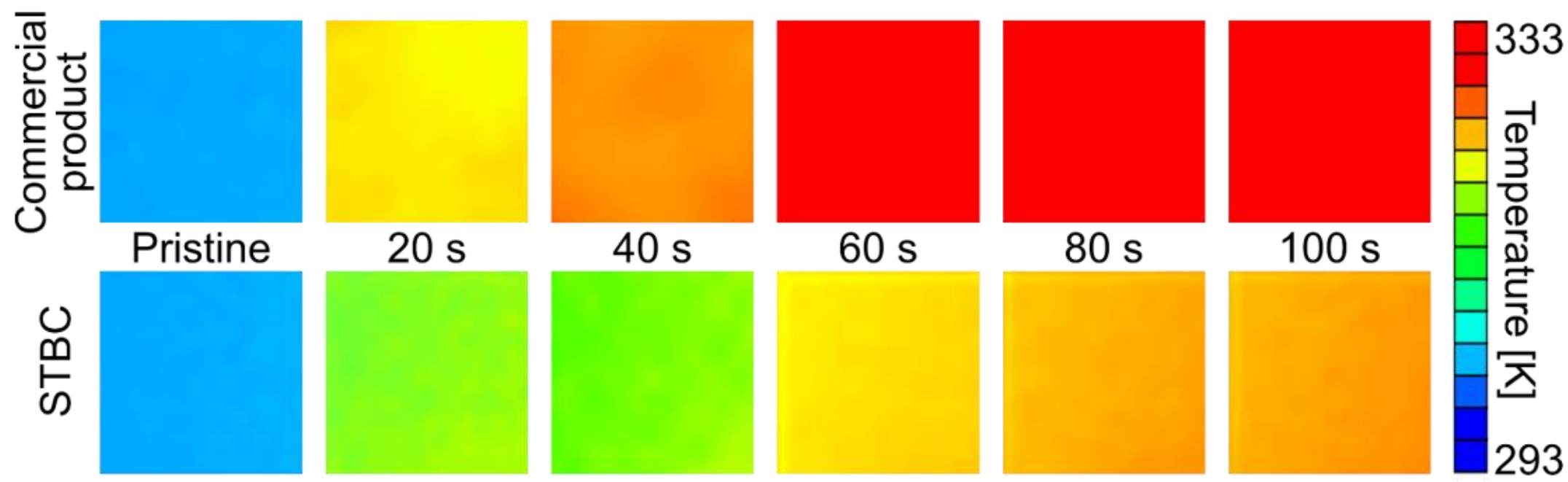

Supplementary Fig. 14 | Infrared images of a commercial radiative cooling product and STBC under 1 sun exposure.

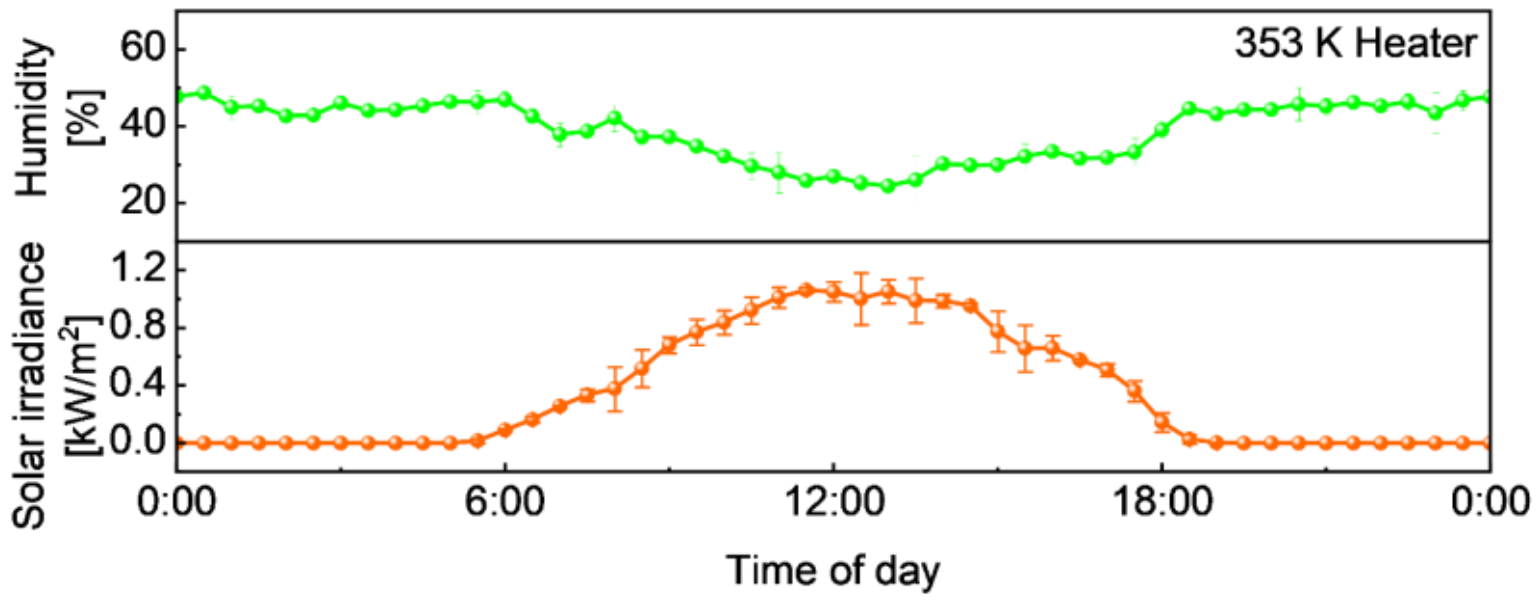

Supplementary Fig. 34 | Diurnal profiles of solar irradiance and ambient humidity in Hefei, China.

Question 2: The enlarged image on the right side of Figure 1c has lower clarity compared to the surrounding images. Please optimize it.

Our reply: Thank you for your careful assessment of image quality. We acknowledge that the enlarged image on the right side of Fig. 1c lacks sufficient clarity, resulting in inconsistency within the figure set and limiting the visibility of fine details. We have re-acquired and replaced the SEM image to ensure high resolution. We also review all figures in the manuscript and supplementary information to ensure consistently high-quality presentation for readers.

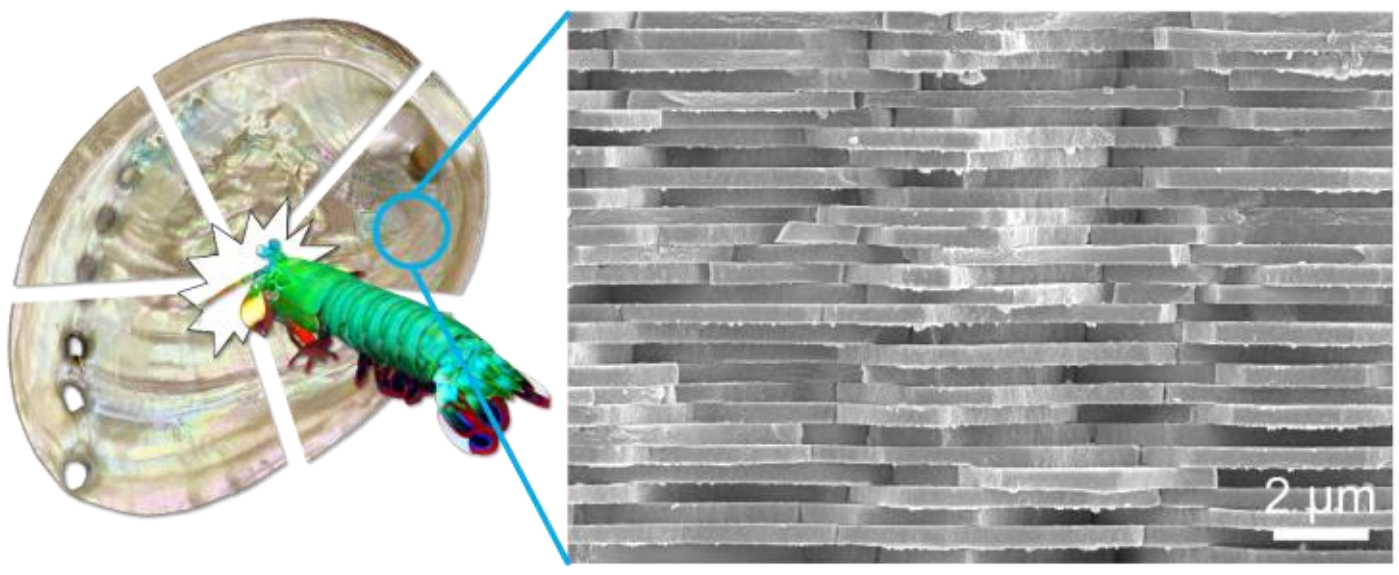

Fig. 1c | Impact-resistant brick-and-mortar configuration inspired by nacre.

Question 3: Figure 1a contains several points that may be subject to some debate. Please be advised.

(1) Can arrows be drawn in the section from the "heat source" to the "conductive" interface to indicate heat transfer from the heat source to the transfer layer?

(2) The model on the right is not closely connected to the actual environment shown on the left. Could a more intuitive connection method be used, such as a magnifying glass

focused on the roof?

(3) This regulation reflects my personal perspective and represents what I consider the most contentious aspect: The right diagram in Fig. 1a visually depicts solar radiation reflection and thermal conduction on the uppermost surface. However, the schematic in the right diagram of Fig. 5a illustrates the material's top-layer reflection, intermediate thermal insulation, and lateral thermal conduction properties. Regarding the “heat radiation surface” section, I personally believe the author's visual continuity in the diagrams is incomplete. In fact, a thorough reading reveals the author intends to convey that their material can reflect sunlight and radiate heat from any surface. However, viewing the images in isolation may confuse readers about which specific surface constitutes the “heat radiation surface.”

Our reply: Thank you for your meticulous and constructive questions, which are invaluable for optimizing the graphics and improving readability. In response to the issues raised for Fig. 1a, we implement the following comprehensive revisions:

(1) Clear arrows are added between the “heat source” and the “conductive” interface to intuitively indicate the heat-flow path from the source to the transfer layer.

(2) The linkage between the real environment on the left and the model on the right is strengthened by introducing a magnified inset focused on the roof, clarifying the logical correspondence between the two.

(3) To address the lack of visual continuity between the right panels of Fig. 1a and Fig. 5a, we emphasize the core concept in Fig. 1a by refining annotations and visual elements. This makes explicit that any surface of the material can radiate heat, thereby eliminating potential confusion about the “heat-radiating surface” when the figure is viewed in isolation and aligning the depiction with the material’s actual functional characteristics.

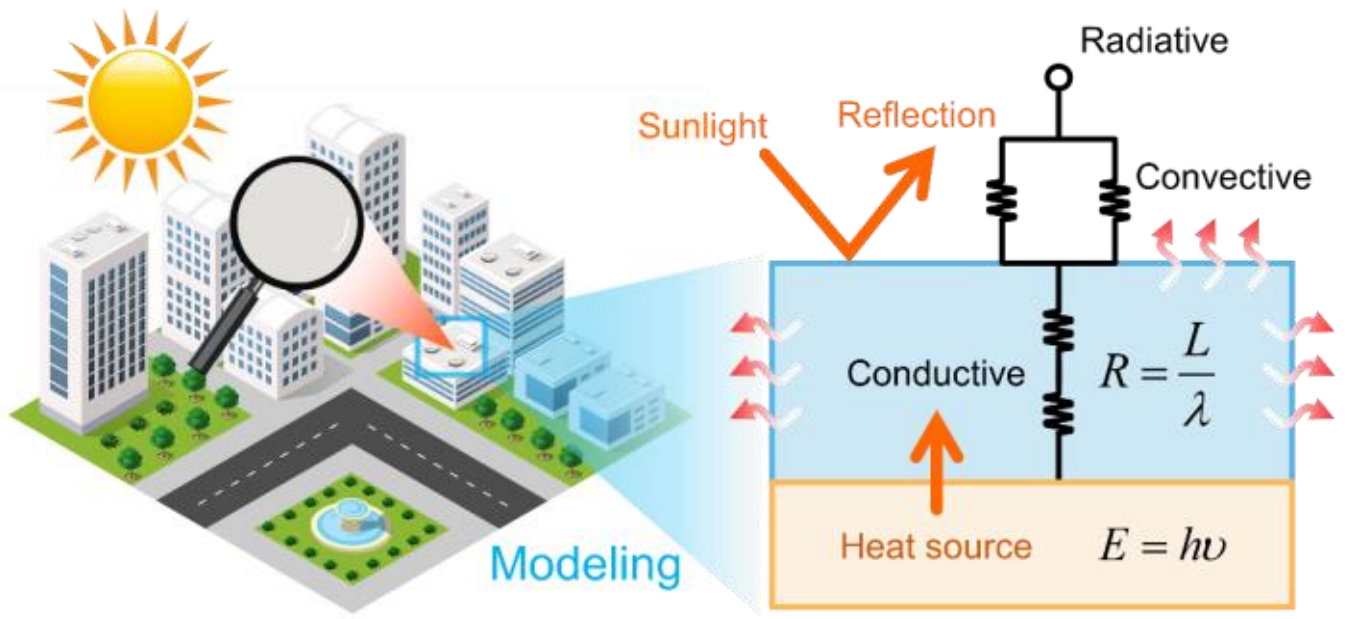

Fig. 1a | Passive cooling model for urban heat islands.

We sincerely appreciate your thorough review. These revisions have substantially enhanced the clarity and overall quality of the work.

Question 4: Regarding the s-STBC structural design: The thickness of the SSG layer directly impacts the balance between thermal insulation and impact resistance. Could you provide "comparative experimental data on the maximum cooling temperature difference and residual impact force of s-STBC under different SSG thicknesses (e.g., 1mm, 3mm, 5mm)"? Please also explain the core considerations for the current thickness selection. Neither the main text nor the references section of this article addresses research on SSG layer thickness.

Our reply: Thank you for highlighting this critical point, which prompts a systematic quantitative study of the SSG-layer thickness. Understanding the trade-offs among thickness, thermal insulation, and impact resistance is essential for optimizing s-STBC design. In response, we conduct comparative experiments with SSG thicknesses of 1, 3, and 5 mm (Supplementary Fig. 27a).

(1) **Thermal insulation:** Under identical 1-sun irradiation, the maximum temperatures of s-STBC with SSG thicknesses of 1, 3, and 5 mm are 375.4, 363.7, and 350.7 K, respectively (Supplementary Fig. 27b). The maximum steady-state temperature decreases markedly with increasing SSG thickness. This is attributed to the SSG layer's low thermal conductivity and high specific heat capacity, which increase thermal resistance between the STBC layers and enable the composite to achieve higher cooling efficiency.

(2) Impact resistance: Under a 3.2 kg drop-hammer impact from a height of 50 cm, the peak residual forces for s-STBC with SSG thicknesses of 1, 3, and 5 mm are 0.82, 0.51, and 0.24 kN, respectively (Supplementary Fig. 27c). Increased SSG thickness enhances impact resistance. Upon impact, the SSG layer adheres to adjacent STBC layers to suppress crack initiation and undergoes plastic deformation to dissipate substantial kinetic energy[R15,R42].

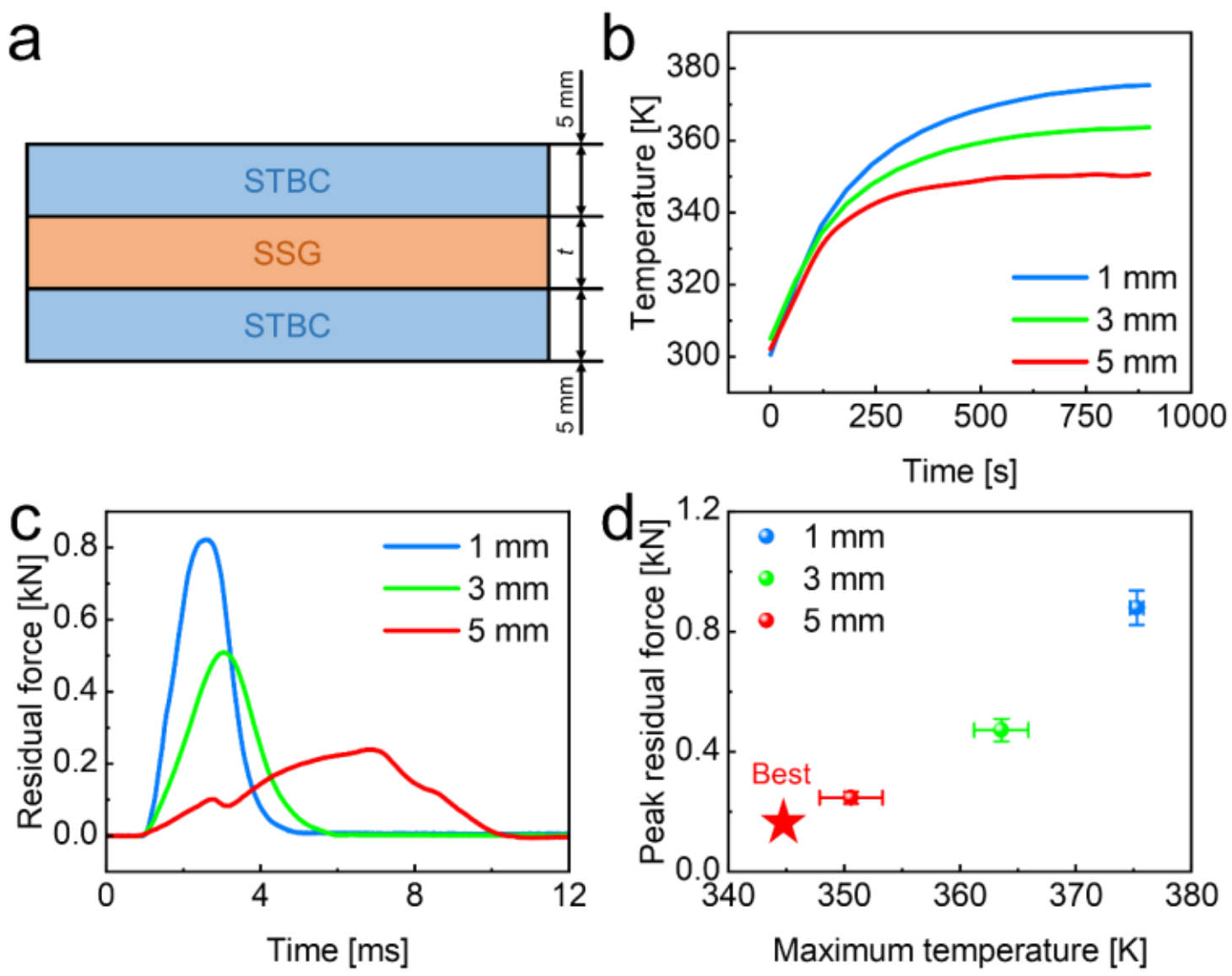

Supplementary Fig. 27 | a s-STBC structural design schematic. **b** Temperature-time curves, **c** residual force-time curves, and **d** thermo-mechanical comparison of s-STBC with SSG interlayer thickness of 1, 3, and 5 mm.

This supplementary work confirms that SSG-layer thickness is pivotal for regulating the multifunctional properties of s-STBC. Balancing maximum temperature, residual force transmission, overall thickness, and creep constraints, a 5-mm-thick SSG interlayer is selected for thermal insulation and impact resistance (Supplementary Fig. 27d).

The corresponding experiments and discussion have been added to the revised manuscript and supplementary information. (Page 20, lines 418-421; Page S17, lines 230-234)

Question 5: The English need to improve if possible.

Our reply: Thank you for pointing out the need for language improvements. We recognize that writing quality is critical for reader comprehension and engagement. Accordingly, we consult recent articles in *Nature Communications* on passive cooling, impact resistance, and bioinspired design, and undertake a comprehensive revision of the English throughout the manuscript to sharpen logic and coherence. We now describe material properties with greater specificity, avoiding overly general terms, to better convey the material's practical value and applications. We believe the revised manuscript meets the journal's English-language standards and facilitates a clear understanding of our research. Thank you for your constructive comment.

Reply to Reviewer #4

Comment: The manuscript by Li et al. designed and fabricated a nacre-inspired composites with passive cooling and impact resistance properties. There is no doubt that the nacre-mimetic materials could exhibit excellent mechanical properties. The anisotropy of the materials is also logic due to the unique structure of nacre-like structure. What make the materials with excellent overall properties compared with other nacre-like materials could be the unique composition. I think this is one of the main contribution and novelty of the work. Overall, the manuscript is well-written with a lot of data, but the design principles and synthesis/fabrication part should be enhanced with more control experiments. I would suggest major revision of manuscripts and the following concerns should be addressed in the revised manuscript.

Our reply: Thank you for recognizing the innovation, comprehensive performance, and data integrity of this work, and for your targeted suggestions. Your feedback has been instrumental in refining the design principles and strengthening the experimental logic. We have expanded the synthesis and fabrication section by incorporating a series of control experiments that systematically probe how the loadings of core components influence the material's structure and properties. The rationale and workflow of the preparation protocol have been clarified. Figures, captions, and accompanying textual descriptions have been refined for clarity, and the reproducibility of the results has been confirmed through repeated experiments. All revisions have been integrated into the revised manuscript and supplementary information, thereby enhancing the scientific rigor and integrity of the work. We appreciate your meticulous review, which has substantially improved the logical coherence and academic value of this study.

Question 1: The authors showed that they can fabricate the composites with nacre-mimetic structure, but the influence of different parameters on the structure and properties have not been investigated. For example, the authors claimed that "Cellulose nanocrystals (CNCs), prepared through the hydrolysis of cellulose, were incorporated to enhance the overall thermo-mechanical performance.", however, they did not

provide any evidence. The authors should vary the amount of CNCs and TA, the content of BNNS and SSG polymer and see how would the structure and properties (both thermal behavior and mechanical properties) change with them.

Our reply: Thank you for your constructive suggestion. In response, we conduct a systematic investigation into how key parameters influence the structure and properties of STBC and standardize the nomenclature of specimens prepared under different conditions. Specimens with BNNS-to-SSG mass ratios of 1:0, 4:1, 2:1 and 1:1 are designated S_iTBC (i = 0-3); those with TA contents of 0, 0.0078, 0.0155, and 0.0310 wt% are denoted ST_jBC (j = 0-3); and those with CNC contents of 0, 0.0155, 0.0310, and 0.0620 wt% are denoted $STBC_k$ (k = 0-3). All specimens prepared under these conditions exhibit well-ordered stacked microstructures, highlighting the versatility and robustness of the preparation method (Supplementary Fig. 3). Experimental measurements show that both thermal conductivity and flexural strength increase with increasing SSG, TA, and CNC contents (Supplementary Figs. 4 and 20c). These findings substantiate the role of each component in enhancing the coupled thermo-mechanical performance of the materials and provide clear experimental evidence for the benefits of CNC reinforcement[R43,R44].

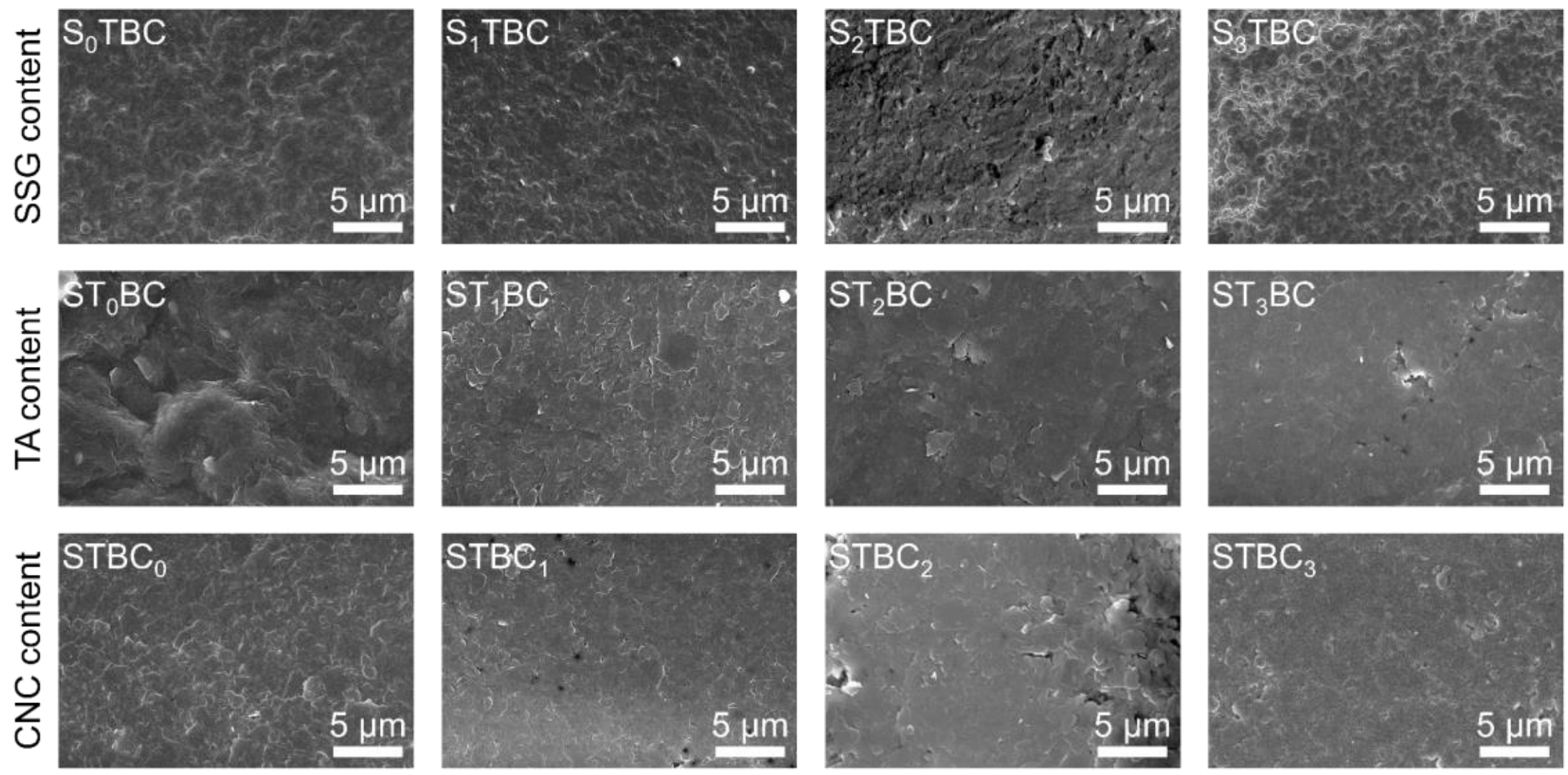

Supplementary Fig. 3 | Top-view microstructures of STBC as a function of material parameters.

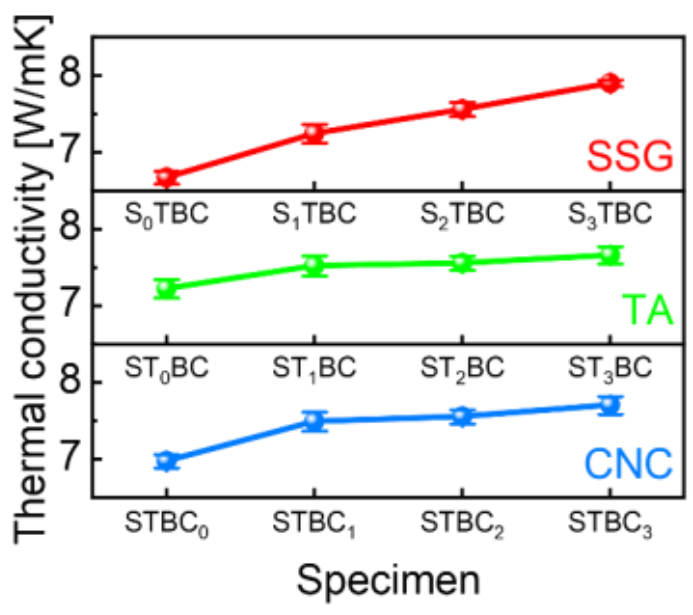

Supplementary Fig. 4 | Thermal conductivity of STBC as a function of material parameters.

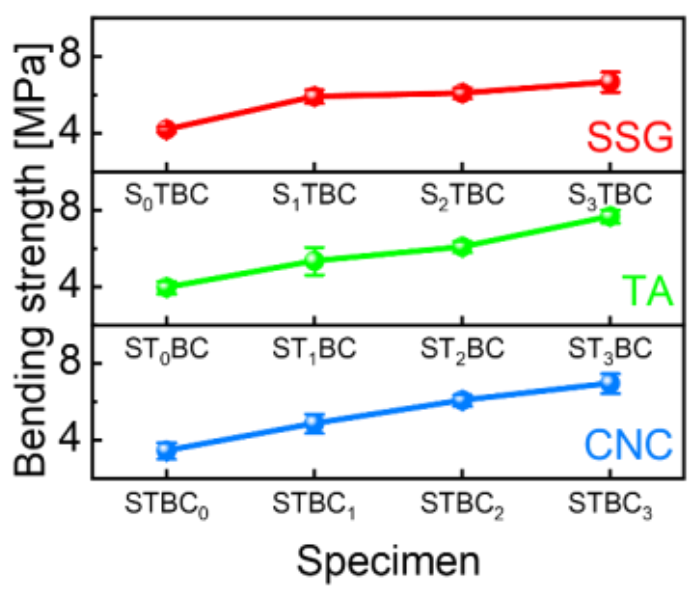

Supplementary Fig. 20c | Bending strength of STBC as a function of material parameters.

Detailed discussion and supporting data on the influence of these parameters are provided in the supplementary information. (Page 8, line 162; Page 10, line 188; Page 16, line 335; Page S7, lines 118-125, 127-129; Page S13, lines 196-199)

Question 2: What is the role of ethyl alcohol? What does dissolution/immersion/drying mean in the Figure 1e. The authors should clear explain the procedures since it is one of the main figure in the article and tell the readers the design principles.

Our reply: Thank you for the detailed question clarifying the preparation logic and composite design principles. In this work, ethanol serves as a solvent carrier to facilitate uniform integration of the SSG mortar with the TBC skeleton. Ethanol is selected because SSG is insoluble in water but stably soluble in organic solvents such as ethanol (Supplementary Fig. 2a-c). Importantly, the dissolution and drying process does not alter the inherent shear-stiffening behavior, ensuring that the material's mechanical

response remains unchanged (Supplementary Fig. 2d).

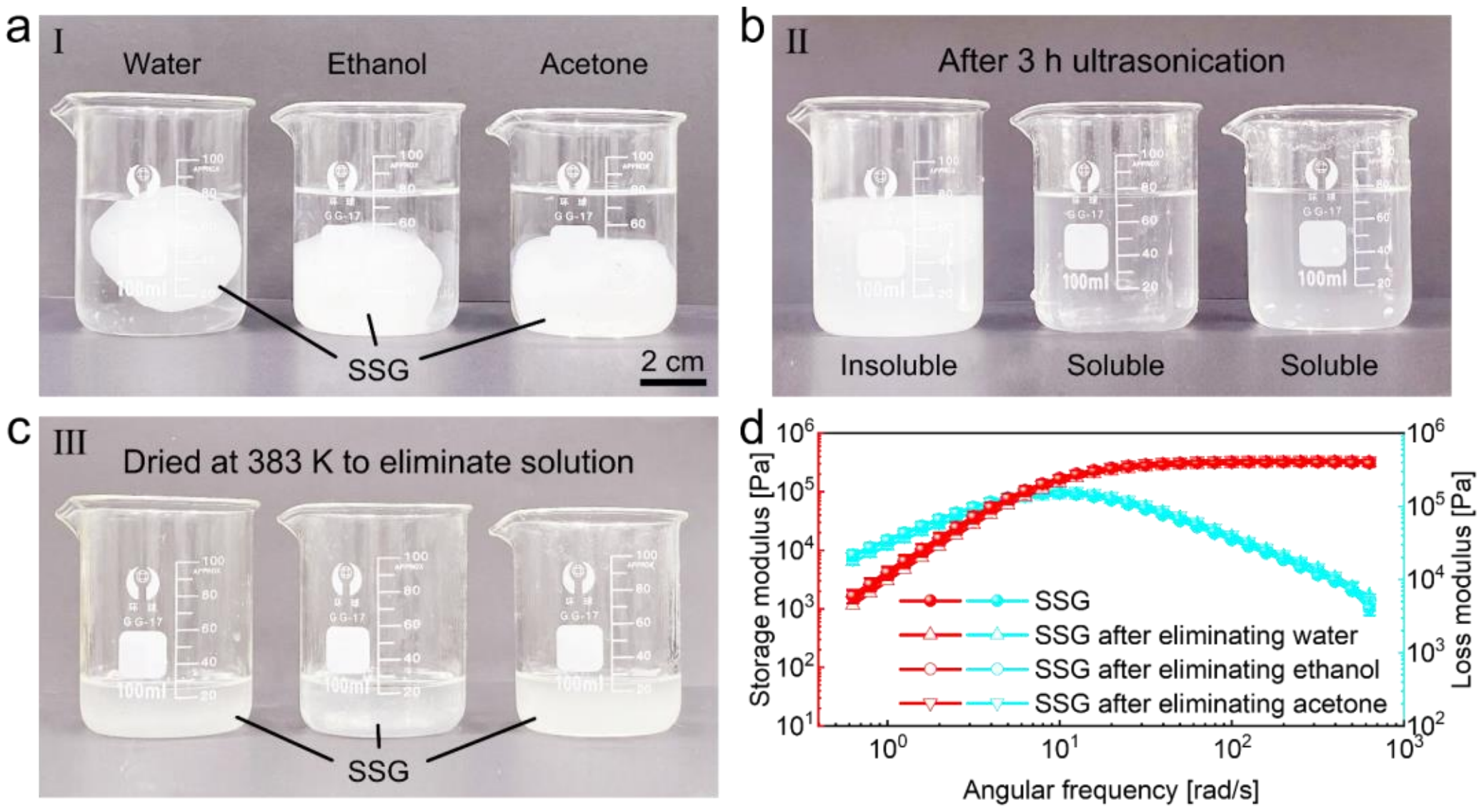

Supplementary Fig. 2 | Stability of SSG in humid and polar environments: **a** Pristine state. **b** After 3 h of ultrasonication. **c** Dried to remove solvent. **d** Corresponding storage and loss moduli.

The key steps in preparing STBC composites, as shown in Fig. 1e, are dissolution, immersion, and drying. Specifically:

(1) Dissolution: SSG is uniformly dissolved in ethanol via ultrasonication to form a stable SSG-ethanol solution.

(2) Immersion: The TBC framework is immersed in this solution and fully permeated under vacuum, ensuring intimate bonding between SSG and TBC.

(3) Drying: The impregnated composite block is heated in an oven at 353 K to remove ethanol, yielding STBC nanocomposite with a brick-and-mortar architecture.

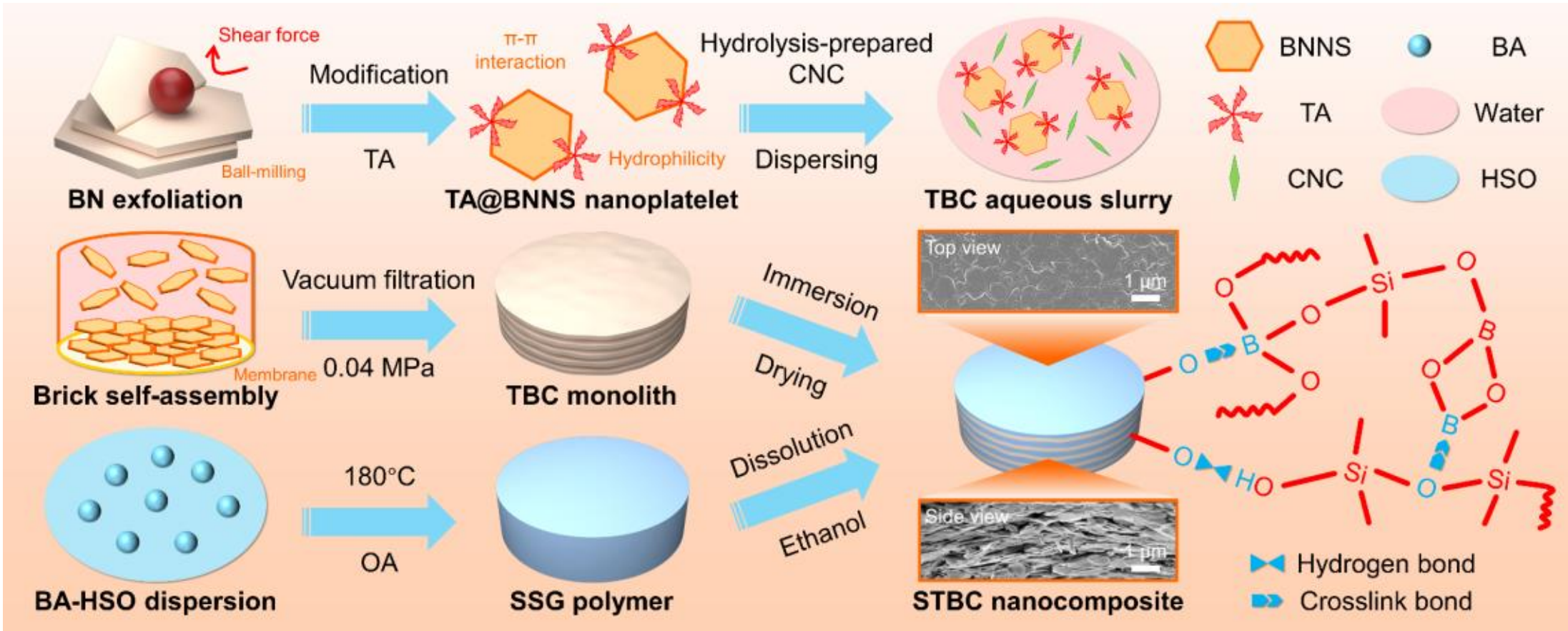

Fig. 1e | Preparation of STBC nanocomposites based on a nacre-mimetic design.

To provide an intuitive representation of the process, we have added a detailed flowchart of the preparation procedure (Supplementary Fig. 1). The Methods section now contains specific parameter values and operational details for each step, clearly articulating the solvent-carrier design principle that enables uniform integration of functional components while preserving their core properties. This clarifies the material preparation logic.

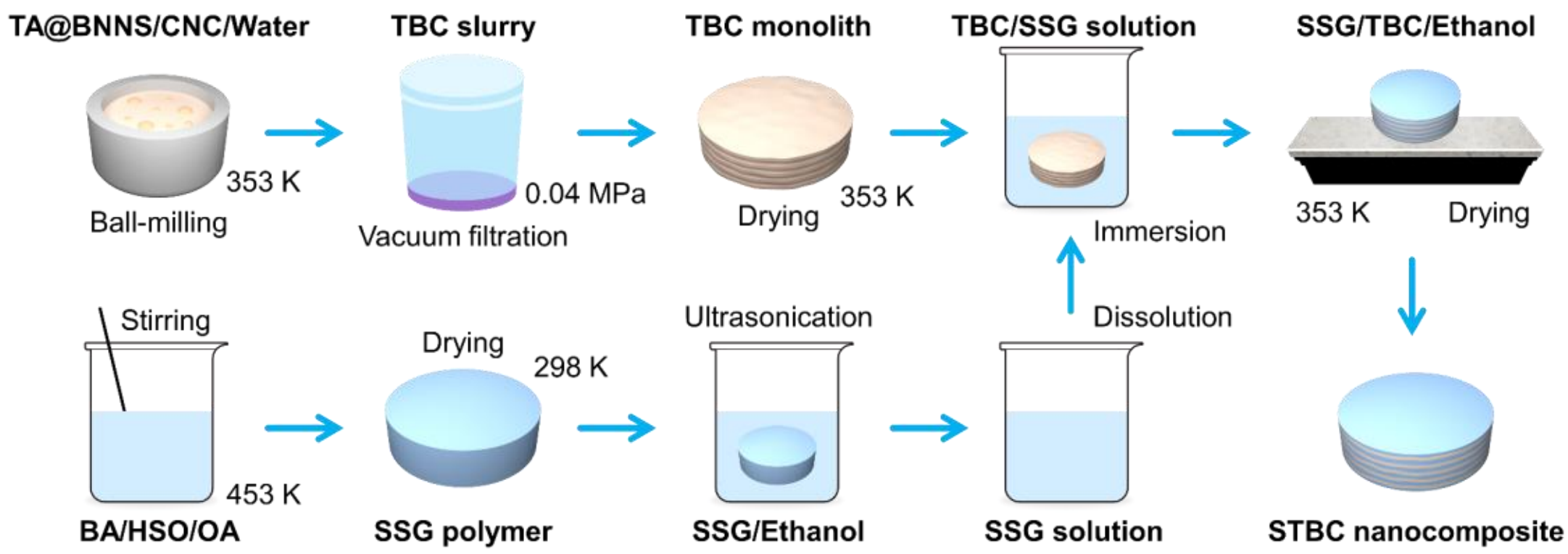

Supplementary Fig. 1 | Detailed flowchart of the STBC preparation procedure.

The corresponding schematics and experimental results have been incorporated into the manuscript. (Page 6, line 113; Page 7, line 147; Page 8, lines 159-160; Page S6, lines 103-109, 111-116)

Question 3: Furthermore, there are numerous similar procedures to fabricate nacre-mimetic composites materials. What makes this one unique compared with those

reported in literature? For example, Nat. Commun. 2017, 8 (1), 287. https://doi.org/10.1038/s41467-017-00392-z.

Our reply: Thank you for your insightful question. The uniqueness of this work lies not only in the preparation strategy but also in overcoming the limitations of traditional biomimetic materials that primarily optimize a single function. Instead, we establish a synergistic multi-performance system. The key distinctions and innovations are as follows:

(1) **Functional integration:** The cited literature[R45] represents a notable advance in nacre-inspired materials, primarily by enabling large-scale fabrication of 3D bulk structures via self-assembly of pre-fabricated 2D nacre-like films. This approach significantly surpasses the mechanical properties of natural nacre and supports structural applications. However, most biomimetic studies have focused on replicating and enhancing mechanical properties, leaving critical functions such as thermal management largely unaddressed and limiting utility in complex or extreme environments.

In contrast, we do not merely replicate nacre's mechanical advantages. Through a synergistic design that combines 1D/2D interpenetrating networks with dynamic B-O crosslinking, we integrate high strength, anisotropic thermal transport, passive radiative cooling, and kinetic impact protection into a single material (Fig. R1). This multifunctional synergy resolves the traditional trade-off between mechanical performance and thermal management, meeting core demands in extreme outdoor settings. By comparison, the cited work and most existing studies optimize mechanical performance alone, without exploring coupled thermal functionality, thereby confining applications to single domains such as structural support or biomedical substitutes.

(2) **Structural and chemical design:** Building on the classic brick-and-mortar framework, our approach introduces a dynamically responsive interface: TBC constructs a directionally thermally conductive brick framework, while B-O crosslinked SSG functions as a dynamic mortar. Through the synergistic interplay of interfacial hydrogen bonding and dynamic crosslinking, the material preserves

nacre's ordered structural advantages while enabling precise regulation of energy dissipation and heat conduction via reversible bond fracture-reformation under external stress fields. In contrast, the cited literature employs a static organic sodium alginate-chitosan matrix with inorganic layers, lacking dynamic responsiveness and therefore unable to achieve coordination across multiple physical fields.

(3) **Transferable paradigm:** The biomimetic integration paradigm of functional skeletons and dynamic response matrices developed here is applicable beyond mechanics-thermotics coupling. It can be generalized to more complex operating conditions, such as electromagnetic interference shielding and adaptation to extreme temperatures through material selection and systematic tuning, thereby providing a transferable design for multifunctional biomimetic materials. By contrast, existing studies largely optimize fabrication processes within specific material systems without establishing a generalizable cross-functional integration strategy.

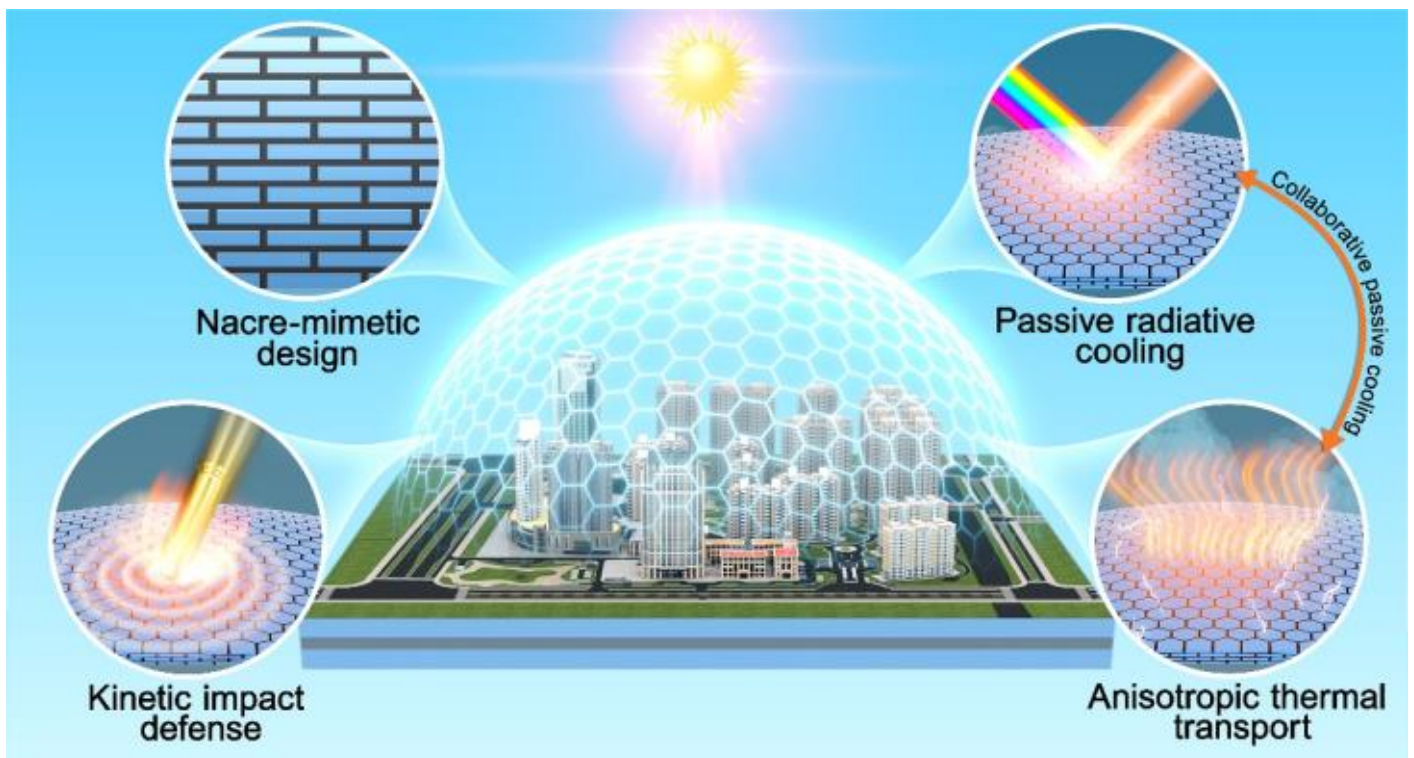

Fig. R1 | Nacre-inspired structural STBC featuring advanced anisotropic thermal transport, passive radiative cooling, and kinetic impact defense.

In summary, the novelty of this work lies in achieving multifunctional synergistic adaptation to extreme environments. Through the innovative design of dynamically responsive architectures and chemistries, we overcome the single-function optimization paradigm of traditional nacre-mimetic materials. This represents a shift from structural replication to functional customization, addressing gaps in thermo-mechanical synergy

and sustainability, and offering practical material solutions for complex scenarios such as outdoor equipment protection. We have supplemented the manuscript with additional explanations to further clarify the innovative positioning and unique value of this work. (Page 3, lines 55-59; Page 8, lines 165-167; Page 22, lines 483-492; Page 23, line 493-494)

Question 4: Figure 2d-e, heat flux mappings at 0.01 ms. What is the X and Y axis of the mapping? And what does the color and arrow mean in the figure? Is there any scale in this figure? Furthermore, the detailed measurement methods should be provided.

Our reply: Thank you for your professional questions. Figs. 2d, e present the nacre-inspired heterogeneous and random homogeneous structural models alongside their corresponding heat-flux maps at 0.01 ms. The *x*- and *y*-axes denote nanosheet orientation in the model, with *x* corresponding to the out-of-plane direction and *y* to the in-plane direction. The color and arrow jointly convey the magnitude and direction of heat flux: variations in color intensity indicate changes in flux magnitude, while arrow orientation shows the heat-transport pathways. A scale bar is provided to facilitate quantitative comparison. The heat-flux maps are generated via simulations using commercial finite-element software. All material parameters used in the simulations are sourced from the literature[R15,R46], ensuring the reliability and validity of the results.

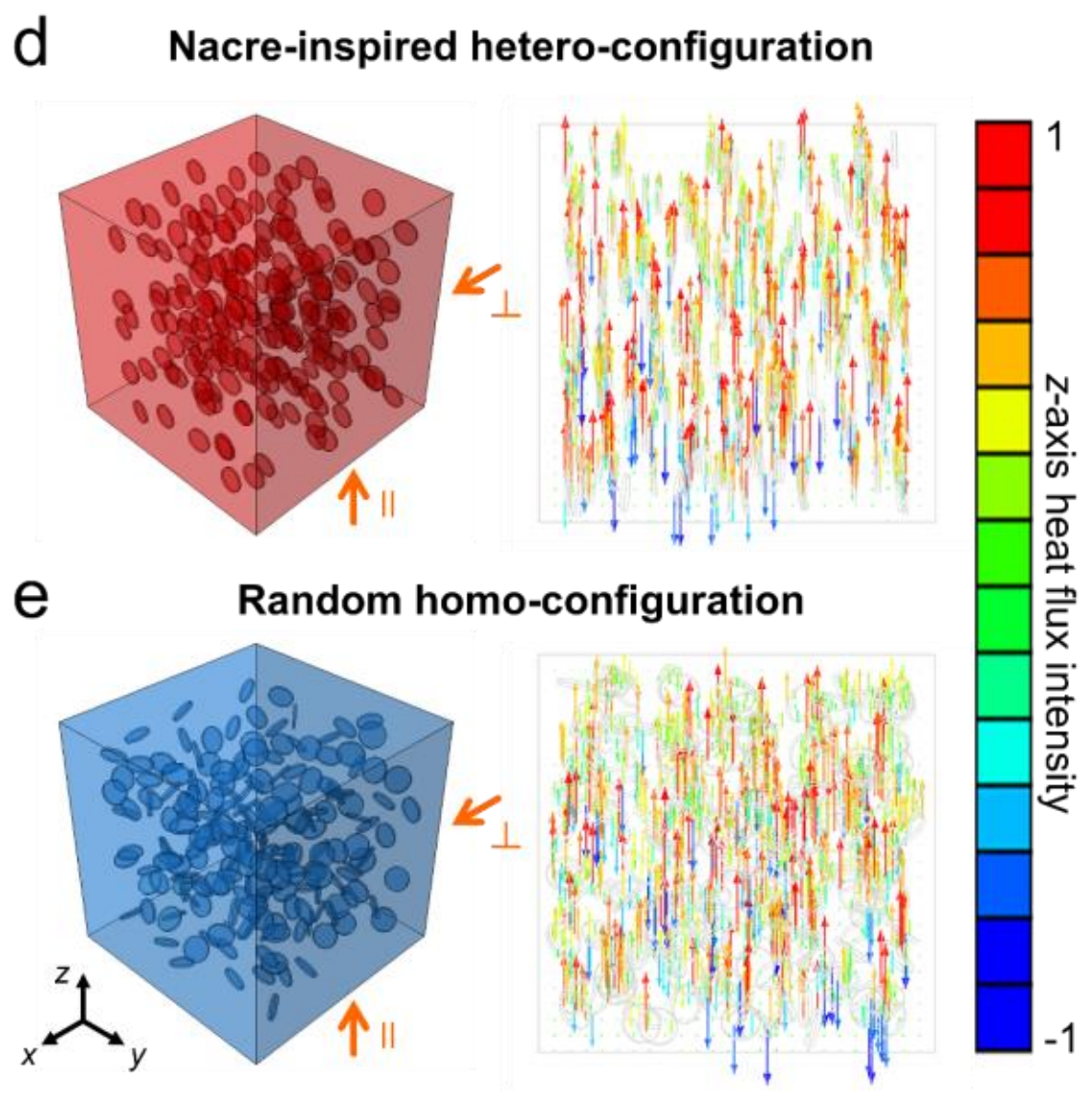

Fig. 2 | **d** Nacre-inspired hetero-configuration and **e** random homo-configuration

models, with corresponding *y*-*z* plane heat-flux maps at 0.01 ms.

Relevant discussion has been incorporated into the manuscript. (Page 9, lines 174-175; Page 10, lines 204-207; Page S8, lines 140-141)

Question 5: Figure 3, the authors only showed the properties of one sample. Again, is this reproducible? What is the influence of different parameters on these properties?

Our reply: Thank you for your constructive feedback.

(1) We replicate all experiments underlying Fig. 3 to ensure the reliability and credibility of the results. The corresponding figures have been updated accordingly. Data are presented as the mean ± SD from $n = 3$ independent samples. Additionally, error bars have been added to all experimental data presentations in this work in accordance with *Nature Communications*' reporting standards.

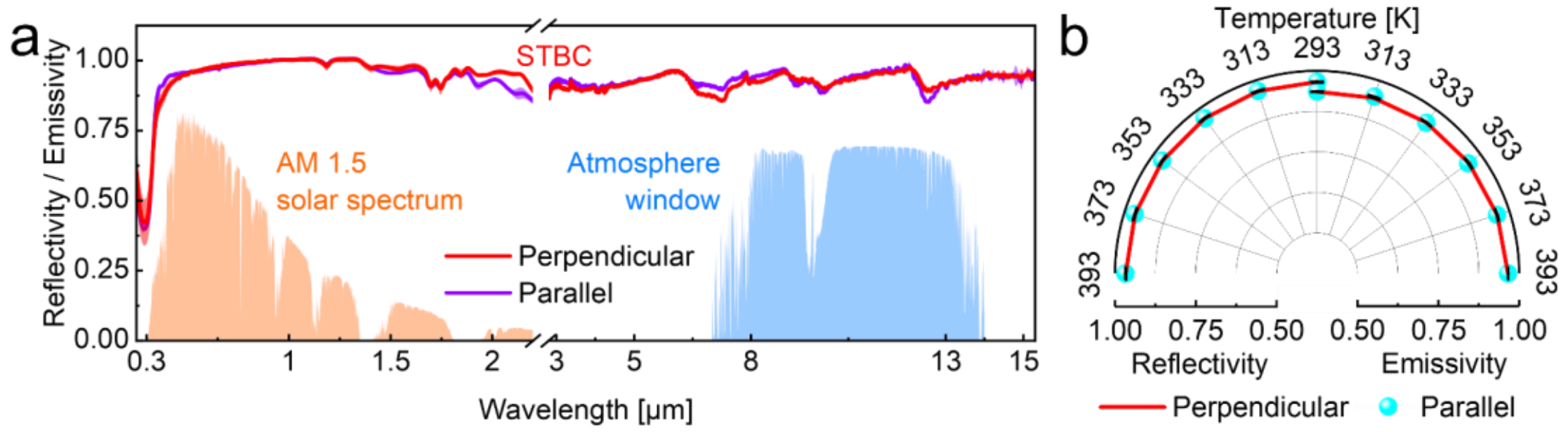

Fig. 3 | a Reflectivity and emissivity of STBC measured in the parallel and perpendicular directions and **b** their temperature-insensitive polar plots.

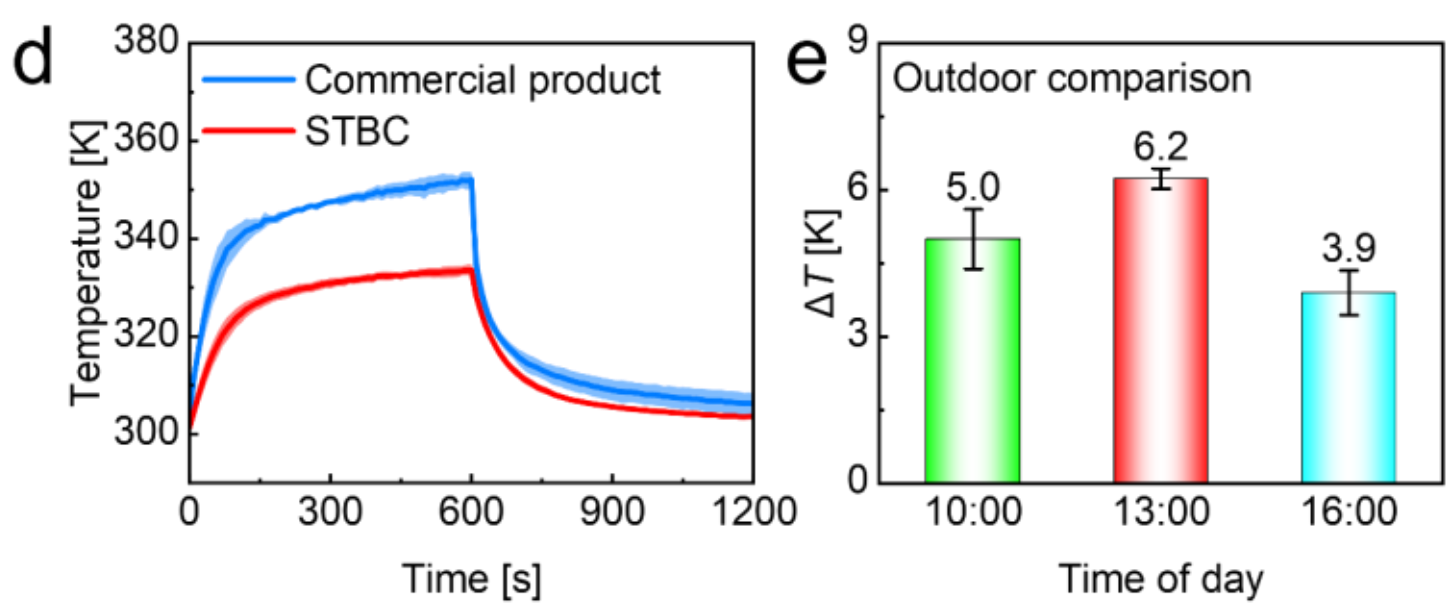

Fig. 3 | Time-dependent temperature comparisons between a commercial radiative cooling product and STBC under **d** 1-sun exposure and **e** real-world outdoor sub-ambient conditions.

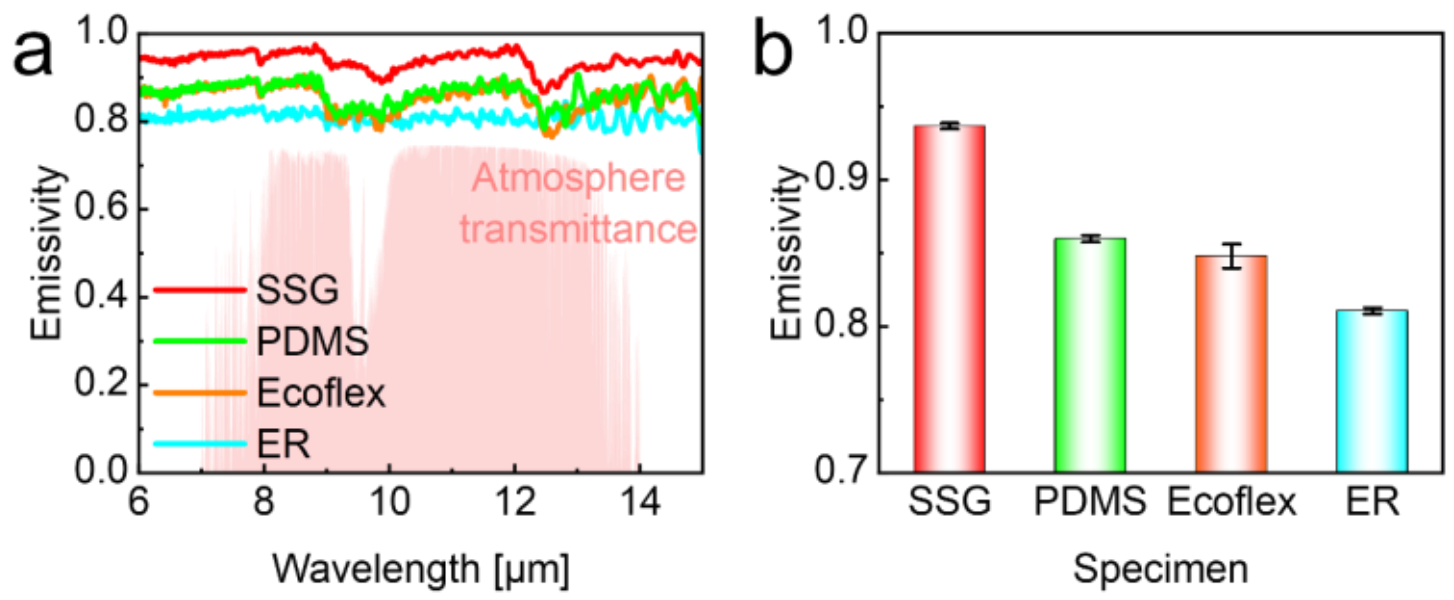

Supplementary Fig. 12 | a Emissivity-wavelength spectra and **b** emissivity comparison of SSG versus high-molecular-weight PDMS, Ecoflex, and epoxy resin (ER) engineering polymers.

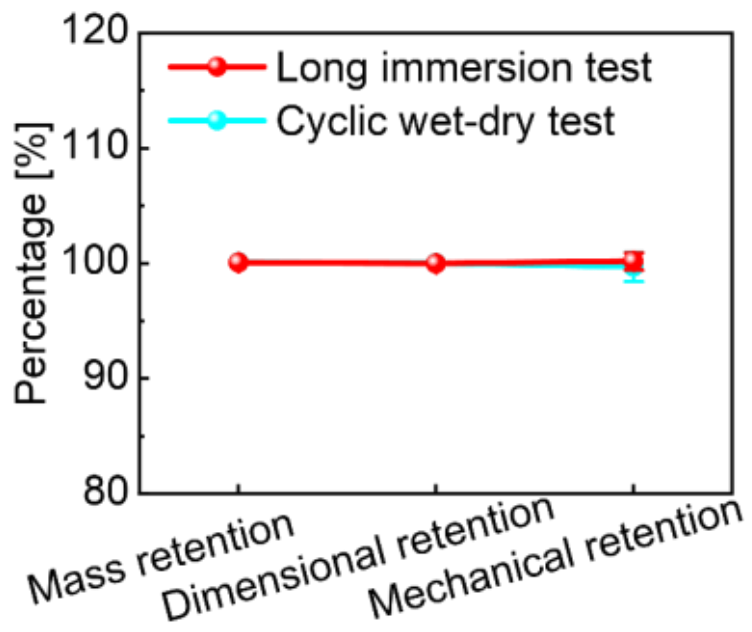

Supplementary Fig. 18b | Mass, dimensional, and mechanical retention of STBC after prolonged immersion and cyclic wet-dry tests.

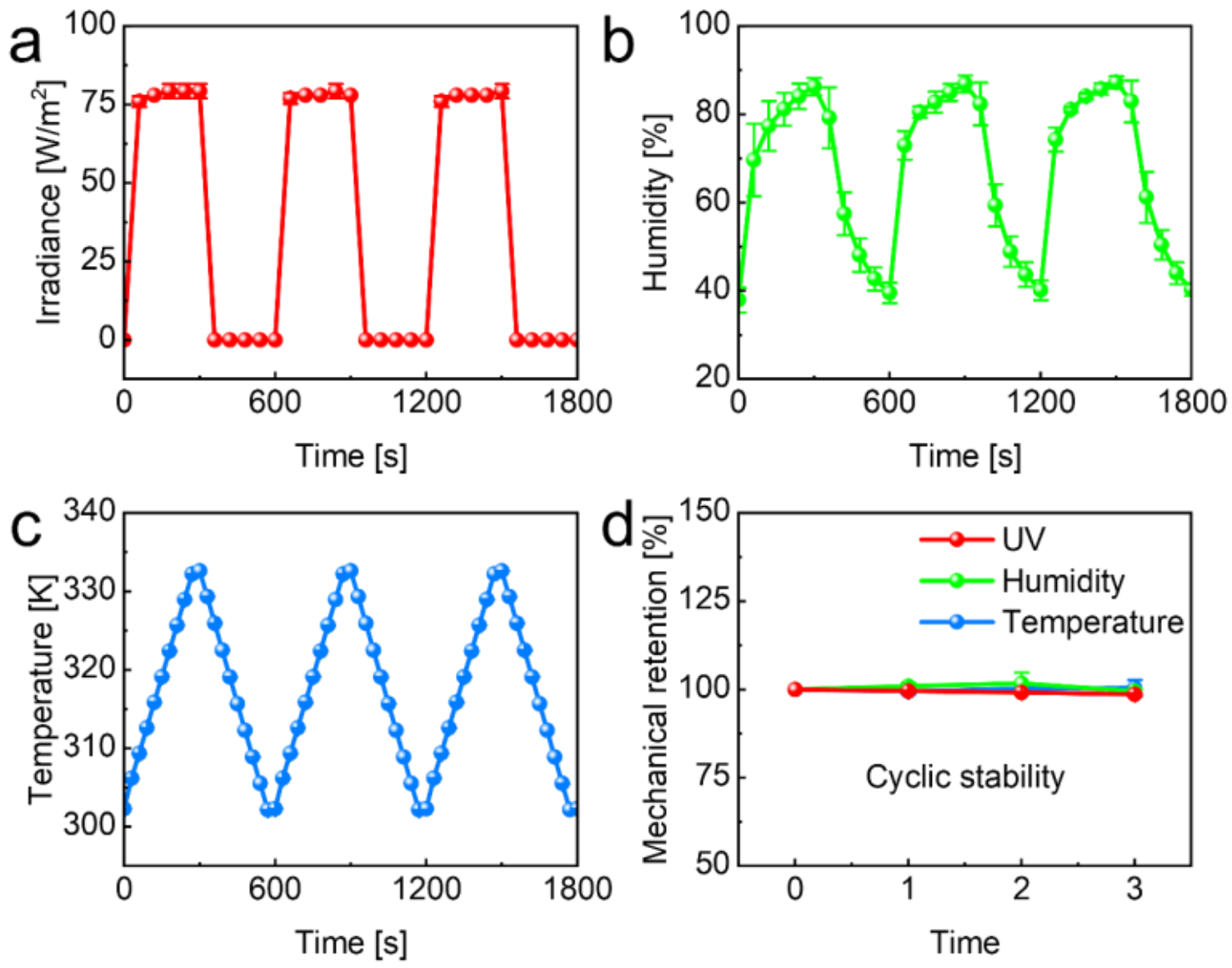

Supplementary Fig. 21 | a UV, **b** humidity, and **c** temperature cycling tests, and **d**

corresponding mechanical retention of STBC.

(2) We systematically investigate the effects of key parameters on STBC properties and standardize the nomenclature for specimens prepared under different conditions: Specimens with BNNS-to-SSG mass ratios of 1:0, 4:1, 2:1 and 1:1 are designated S_iTBC (i = 0-3); those with TA contents of 0, 0.0078, 0.0155, and 0.0310 wt% are denoted ST_jBC (j = 0-3); and those with CNC contents of 0, 0.0155, 0.0310, and 0.0620 wt% are denoted $STBC_k$ (k = 0-3).

The experimental results show that increasing the SSG content leads to a slight decrease in the reflectance of STBC, primarily due to mild suppression of light reflection arising from substrate encapsulation by the SSG matrix (Supplementary Fig. 11). In contrast, emissivity significantly increases, which is attributed to the optical properties of SSG. For TA and CNC, given their extremely low mass fractions in the system, their effects on the reflectance and emissivity of STBC are weak and do not exhibit a systematic trend.

The relevant data and discussion have been incorporated into the manuscript. (Page 13, lines 252-253; Page S10, lines 158-160)

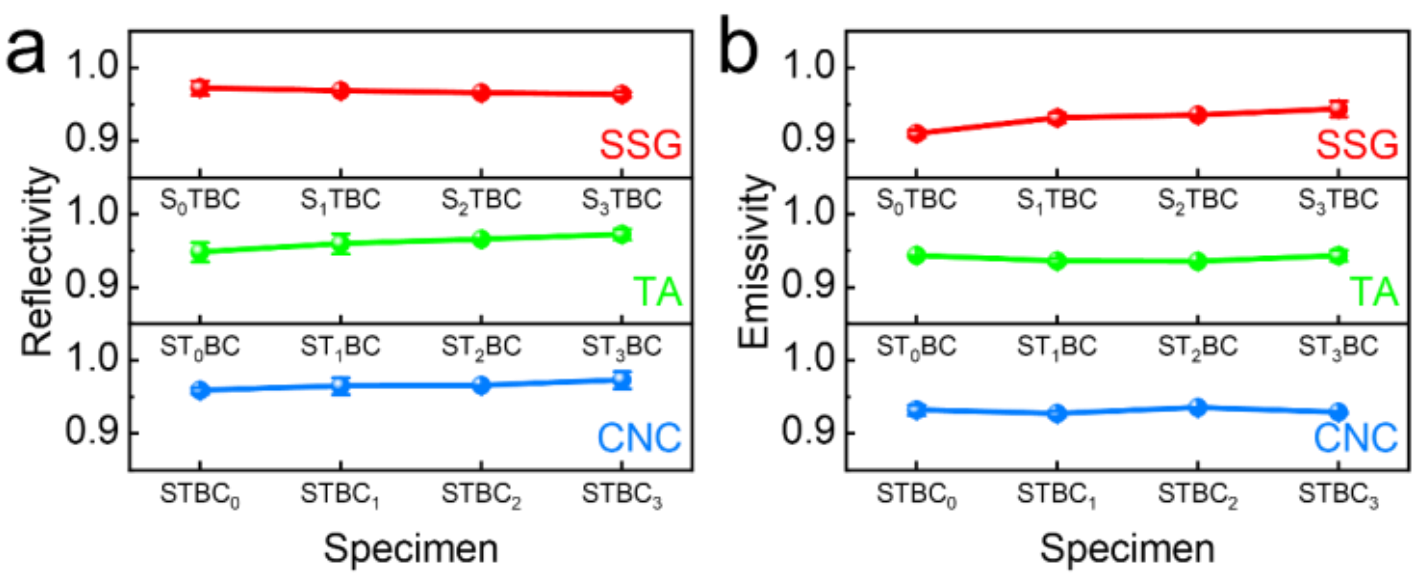

Supplementary Fig. 11 | a Reflectivity and **b** emissivity of STBC as a function of material parameters.

Question 6: In the design strategy, Figure 1b, the authors compared the thermal conduction of amorphous materials and crystalline materials. How does this mechanism contribute to the design of the composites? Could the author provide examples showing similar composites made of amorphous materials that exhibited lower thermal

properties?

Our reply: Thank you for your interest in contrasting thermal-conduction mechanisms in amorphous versus crystalline materials. Fig. 1b provides a visual analogy of vibrational transmission, revealing the intrinsic link between structural order and thermal-conduction efficiency: the regular arrangement in crystalline materials can be likened to a series of uniformly aligned Newton's cradles, where vibrational energy propagates rapidly along oriented pathways with minimal thermal resistance. By contrast, the disordered structure of amorphous materials (e.g., homogeneous epoxy resin[R47]) resembles randomly arranged cradles. Initial vibrations disperse among randomly positioned atoms or molecules, hindering the formation of continuous heat-conduction pathways and resulting in high intrinsic thermal resistance. This understanding provides clear design guidance for composites: by emulating nacre's ordered brick-and-mortar architecture, highly thermally conductive TBC can be engineered as a directionally aligned thermal-conduction skeleton. This structural strategy circumvents the inherent thermal-resistance limitations of amorphous SSG matrices, enabling efficient directional heat transfer.

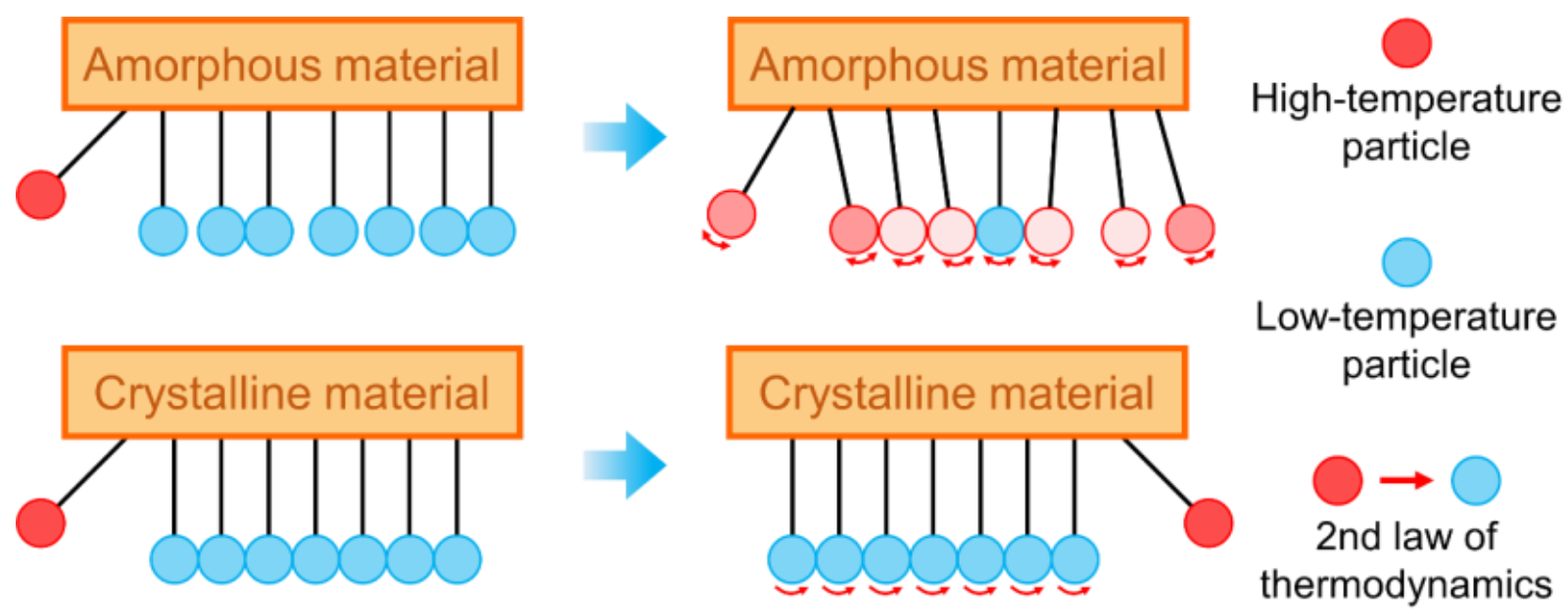

Fig. 1b | Thermal-conduction efficiency of amorphous and crystalline materials.

For example, BNNS-based networks have been integrated into amorphous epoxy matrices to enhance thermal transport[R48]. However, the resulting composites show modest gains in thermal conductivity as a single-function metric, falling short of the values by STBC developed in this work. This underscores the rationale and innovation of our approach: rather than relying solely on filler networks, we overcome the thermal-transport bottlenecks inherent to amorphous matrices through structural ordering,

yielding thermal conductivities far exceeding those of comparable amorphous-matrix composites.

The relevant discussion has been added to the manuscript. (Page 6, lines 110; Page 7, 122-130)

Question 7: In the design strategy, Figure 1d, the authors demonstrate the possible intelligent adjustable stiffness. How would this contribute to the overall function of the materials? It would be good if the authors could demonstrate the "intelligent" function of the final composites.

Our reply: Thank you for the valuable question regarding intelligent adjustable stiffness. The property demonstrated in Fig. 1d arises from the unique rate-sensitive mechanical behavior of SSG, which is central to the material's overall functionality. In practical use, when outdoor equipment experiences low-frequency small-amplitude disturbances (e.g., daily vibrations), dynamic B-O bonds within the SSG network can move freely. The material exhibits viscous flow, accommodating deformations and avoiding damage associated with excessive rigidity. Under high-rate impacts (e.g., hail strikes), these dynamic crosslinks rapidly entangle and reinforce[R15,R49], and the storage modulus increases by approximately three orders of magnitude, transitioning the material into an elastomeric state with pronounced impact-hardening effects. This efficiently dissipates impact kinetic energy and minimizes force transmission to underlying equipment. Such rate-dependent stiffness enables excellent adaptability under benign conditions while providing robust protection during extreme events, meeting the dual requirement for flexible adaptation and rigid protection in outdoor equipment.

The "intelligent" functionality of the final composite is validated through multiple experiments: In dynamic mechanical testing, bending stress increases markedly with strain rate, evidencing stiffness adaptation to load intensity (Fig. 4b). In ballistic impact tests at 50 m s^{-1}, the material responds rapidly, deflecting projectiles and preserving structural integrity via abrupt stiffness changes (Fig. 4e). Meanwhile, energy dissipation remains consistent across daily temperature fluctuations (298-373 K), demonstrating

environmental adaptability (Fig. 4c). Furthermore, the sandwich-structured s-STBC modulates stress distribution according to impact energy, achieving 97.90% kinetic-energy dissipation through stiffness adaptation while maintaining structural stability (Fig. 5h). Collectively, these experiments confirm that the material's rate-dependent, tunable stiffness has been successfully implemented in the final composite, providing essential mechanical protection and thermal reliability for the integrated functions of impact mitigation and passive radiative cooling.

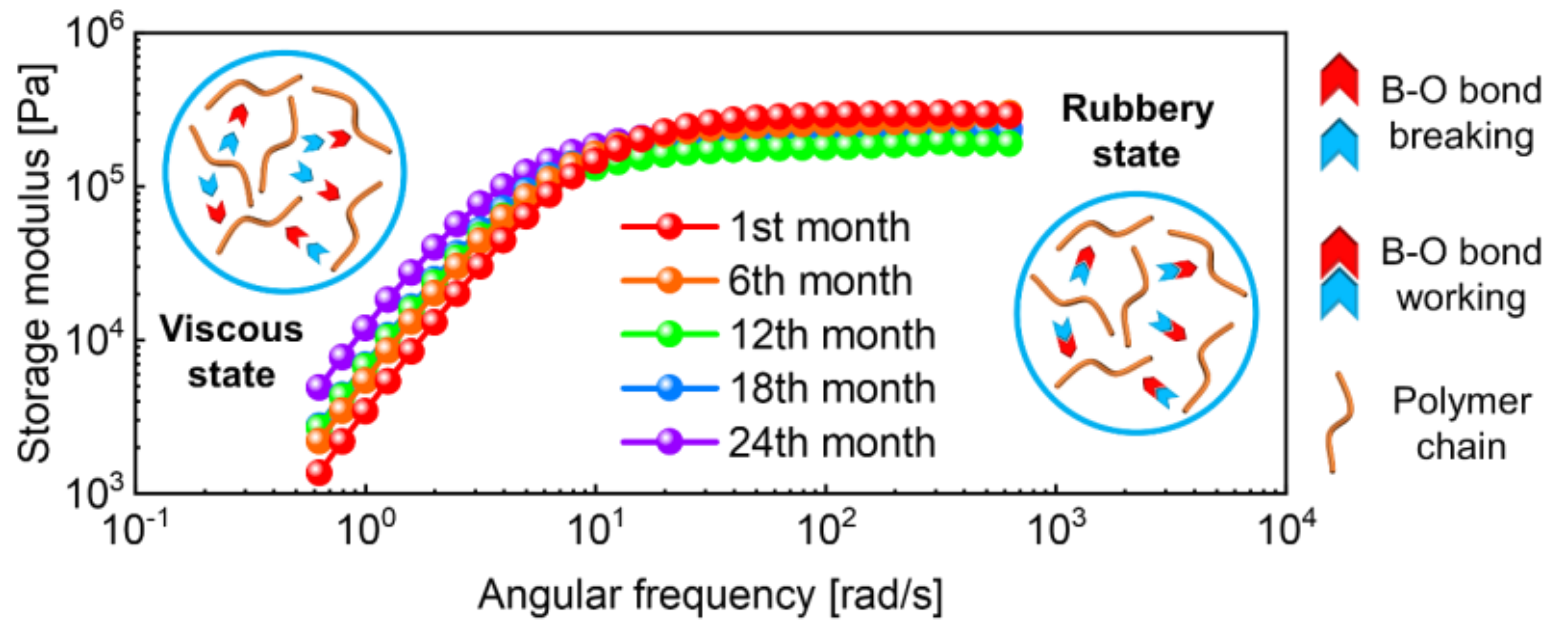

Fig. 1d | Intelligent tunable stiffness and long-term stability of SSG.

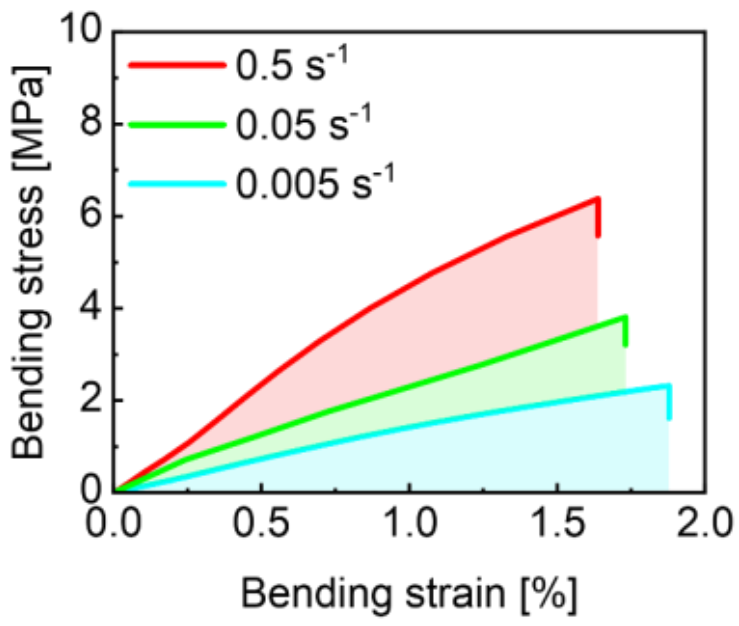

Fig. 4b | Shear stiffening of STBC.

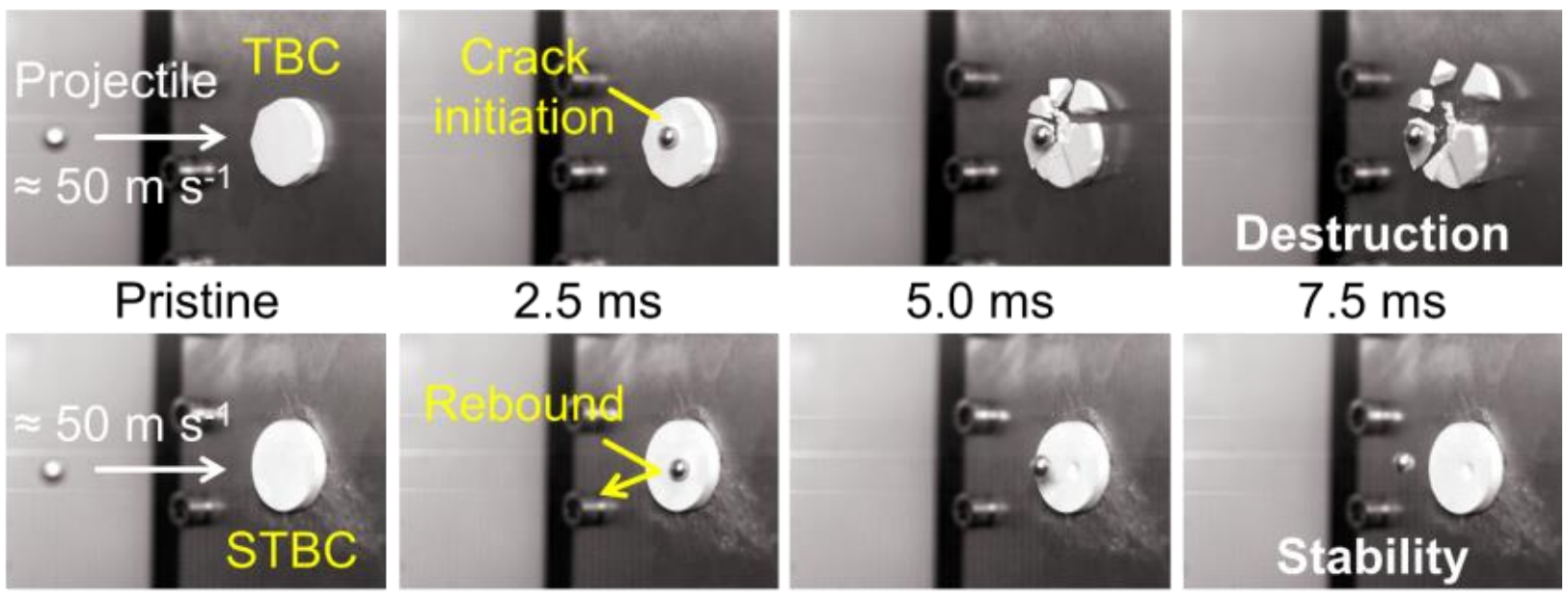

Fig. 4e | High-speed projectile impact processes of TBC and STBC.

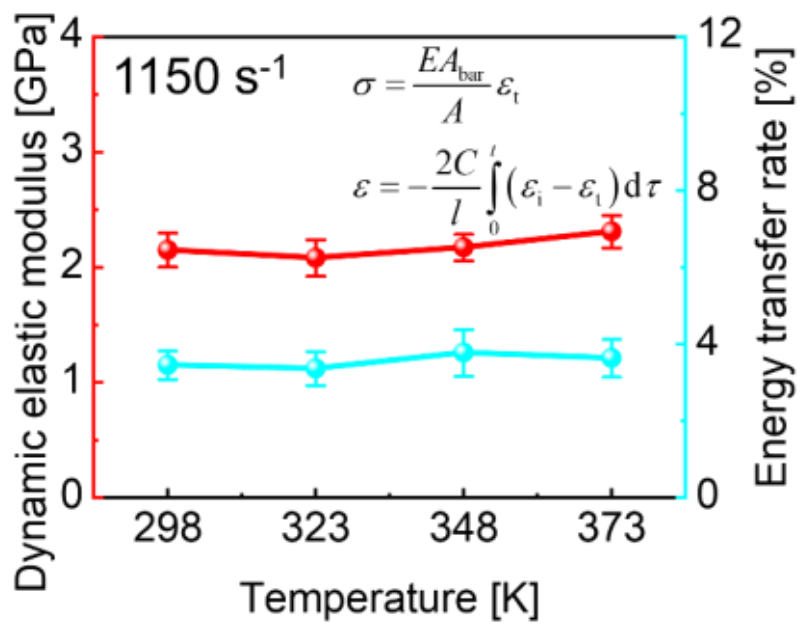

Fig. 4c | Thermal robustness of STBC.

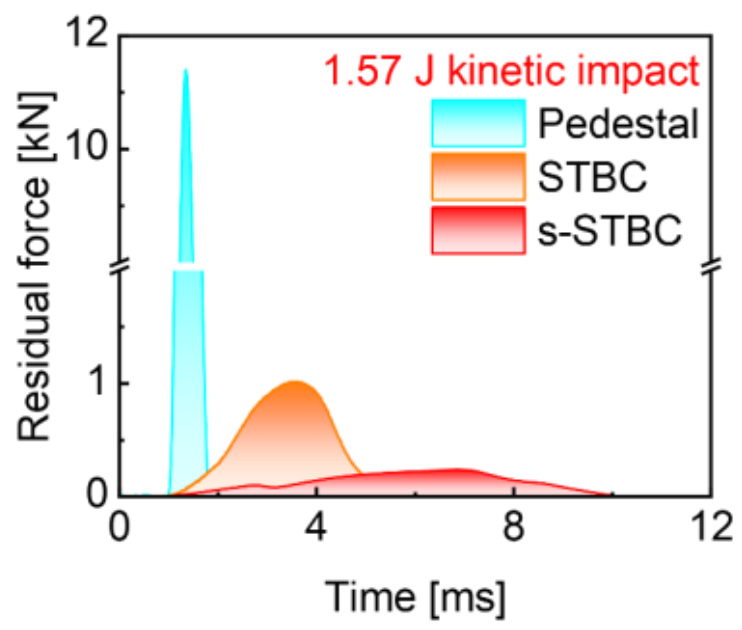

Fig. 5h | Residual forces recorded for pedestal, STBC and s-STBC under a 3.2 kg impact.

The corresponding discussion has been incorporated into the revised manuscript. (Page 15, line 318; Page 16, lines 333-334, 337-339; Page 17, lines 357-358; Page 19, line 398-399; Page 21, lines 452-453)

Question 8: It would be good if the authors could further elaborate on the future direction to improve the properties of these kind of materials. For example, by changing the composition of brick and mortar to which materials could potentially be better?

Our reply: We appreciate your insightful suggestion. The brick-and-mortar architecture affords substantial design latitude and provides a compelling pathway for further performance enhancement. Future iterations may enable autonomous tuning of anisotropic thermal conductivity through mechanically reconfigurable designs that permits controlled deformation, allowing reversible switching between thermally conductive and insulating states. Moreover, judicious selection of brick-and-mortar constituents, coupled with targeted channel architectures, can precisely tailor properties

and realize multifunctionality. For example, employing harder inorganic nanosheets as the brick can further improve mechanical strength and impact resistance; incorporating shape-memory smart materials as the brick can conform to complex curved surfaces for device protection, thereby enhancing conformability and protective efficiency; introducing conductive fillers into the mortar imparts electrical conductivity, enabling selective transmission or attenuation of electromagnetic waves and broadening applications in electronic device protection. By synergistically controlling the composition and microstructure of the brick and mortar, comprehensive improvements in mechanical performance and functional adaptability can be achieved. These considerations motivate continued exploration of biomimetic brick-and-mortar composites and will facilitate their deployment in practical settings.

The corresponding discussion has been incorporated into the revised manuscript. (Page 22, lines 483-492; Page 23, lines 493-494)

We sincerely hope that these efforts further improve the quality of the manuscript. Thank you again for your thoughtful review.

References

R1. Liang, X. et al. Impact-resistant hydrogels by harnessing 2D hierarchical structures. *Adv. Mater.* **35**, 2207587 (2022).

R2. Wu, J. et al. Acid-assisted toughening aramid aerogel monoliths with ultralow thermal conductivity and superior tensile toughness. *Adv. Funct. Mater.* **34**, 2307072 (2023).

R3. Zhang, Z., Qian, L., Cheng, J., Xie, Q., Ma, C. & Zhang, G. Room-temperature self-healing polyurea with high puncture and impact resistances. *Chem. of Mater.* **35**, 1806-1817 (2023).

R4. Tan, Y. et al. Bioinspired hierarchical hydrogels engineered with extreme impact resistance. *Adv. Funct. Mater.* **35**, e08034 (2025).

R5. Li, M. et al. Resilient and antipuncturing Si_3N_4 nanofiber sponge. *Nano Lett.* **23**, 1289-1297 (2023).

R6. Rauner, N., Meuris, M., Zoric, M. & Tiller, J. C. Enzymatic mineralization generates ultrastiff and tough hydrogels with tunable mechanics. *Nature* **543**, 407-410 (2017).

R7. Liu, X. et al. Engineered self-healable elastomer with giant strength and toughness via phase regulation and mechano-responsive self-reinforcing. *Chem. Eng. J.* **410**, 128300 (2021).

R8. Zhang, H. J. et al. Tough physical double-network hydrogels based on amphiphilic triblock copolymers. *Adv. Mater.* **28**, 4884-4890 (2016).

R9. Chen, X. et al. Extremely tough, puncture-resistant, transparent, and photoluminescent polyurethane elastomers for crack self-diagnose and healing tracking. *ACS Appl. Mater. Interfaces* **12**, 30847-30855 (2020).

R10. Yin, Z., Hannard, F. & Barthelat, F. Impact-resistant nacre-like transparent materials. *Science* **364**, 1260-1263 (2019).

R11. Fan, Z. et al. Synchronous enhancement of safety protection and impact perception in intelligent leather. *Adv. Compos. Hybrid Ma.* **8**, 146 (2025).

R12. Zhang, Z. et al. In-situ localised alignment assisted salting-out enhanced ionogels with high strength, toughness and impact resistance. *Nat. Commun.* **16**, 7683 (2025).

R13. Chen, G., Wu, J., Wang, Z., Zhu, H., Zhu, S. & Zhang, Q. Armored polymer-fluid gels with integrated damping and impact protection across broad temperatures. *Sci. Adv.* **11**, eadv5292 (2025).

R14. Yang, J., Zhao, C., Lai, S., Wang, D. & Gong, X. Hybrid additive manufacturing of shear-stiffening elastomer composites for enhanced mechanical properties and intelligent wearable applications. *Adv. Mater.* **37**, 2419096 (2025).

R15. Li, Z. et al. Bioinspired flame-retardant and impact-resistant aramid composites via nacre-mimetic self-assembly for firefighting applications. *Adv. Mater.* **37**, 2508606 (2025).

R16. Wu, J. et al. A lightweight aramid-based structural composite with ultralow thermal conductivity and high-impact force dissipation. *Matter* **5**, 2265-2284 (2022).

R17. https://epb.zibo.gov.cn/art/2019/5/16/art_2210_1669107.html.

R18. Chen, H., Jiang, K., Wu, Q., Zhang, X., Qian, C. & Fan, D. Mussel byssus cuticle-inspired low-carbon footprint soybean meal adhesives with high-strength and anti-mildew performance. *Ind. Crop. Prod.* **222**, 119433 (2024).
R19. Malovrh Rebec, K., Turk, J. & Kunaver, M. Quantifying the environmental implication of cotton-fiber-based nanocrystalline cellulose: A life-cycle assessment. *Sustain. Prod. Consump.* **45**, 192-202 (2024).
R20. Cai, C. et al. Cellulose metamaterials with hetero-profiled topology via structure rearrangement during ball milling for daytime radiative cooling. *Adv. Funct. Mater.* **34**, 2405903 (2024).
R21. Ji, C., Chen, X., Jia, Y., Ding, N. & Wang, Y. Green modification of maple leaf-derived lignin-containing nanocellulose through mechanochemistry for 3D printable pickering emulsions. *ACS Sustainable Chem. Eng.* **13**, 22084-22097 (2025).
R22. Yu, D. et al. Volume-metallization 3D-printed polymer composites. *Adv. Mater.* **36**, 2403088 (2024).
R23. Han, Y., Ruan, K. & Gu, J. Multifunctional thermally conductive composite films based on fungal tree-like heterostructured silver nanowires@boron nitride nanosheets and aramid nanofibers. *Angew. Chem. Int. Edit.* **62**, e202216093 (2022).
R24. Zhang, Z. et al. Highly oriented SiC@SiO_2 ceramic fiber aerogels with good anisotropy of the thermal conductivity and high-temperature resistance. *Adv. Sci.* **12**, 2416740 (2025).
R25. Zhang, Z. B., Gao, H. L., Chen, S. M. & Yu, S. H. Anisotropically thermal-protective porous ceramics enabled by nacre-like framework. *Adv. Mater.* **37**, 2506308 (2025).
R26. Chan, K.-Y. et al. Scalable anisotropic cooling aerogels by additive freeze-casting. *Nat. Commun.* **13**, 5553 (2022).
R27. Yu, H. et al. Highly thermally conductive polymer/graphene composites with rapid room-temperature self-healing capacity. *Nano-Micro Lett.* **14**, 135 (2022).
R28. Wang, Z. Y. et al. Functional carbon springs enabled dynamic tunable microwave absorption and thermal insulation. *Adv. Mater.* **36**, 2412605 (2024).
R29. Liao, X. et al. Extremely low thermal conductivity and high electrical conductivity of sustainable carbon-ceramic electrospun nonwoven materials. *Sci. Adv.* **9**, eade6066 (2023).
R30. Su, L. et al. Anisotropic and hierarchical SiC@SiO_2 nanowire aerogel with exceptional stiffness and stability for thermal superinsulation. *Sci. Adv.* **6**, eaay6689 (2020).
R31. Zhou, Y. et al. Defects vibrations engineering for enhancing interfacial thermal transport in polymer composites. *Sci. Adv.* **11**, eadp6516 (2025).
R32. Zhuang, Y. et al. Flexible graphene nanocomposites with simultaneous highly anisotropic thermal and electrical conductivities prepared by engineered graphene with flat morphology. *ACS Nano* **14**, 11733-11742 (2020).
R33. Han, Y. et al. Highly thermally conductive aramid nanofiber composite films with synchronous visible/infrared camouflages and information encryption. *Angew. Chem. Int. Edit.* **63**, e202401538 (2024).

R34. Ruan, K., Shi, X., Zhang, Y., Guo, Y., Zhong, X. & Gu, J. Electric-field-induced alignment of functionalized carbon nanotubes inside thermally conductive liquid crystalline polyimide composite films. *Angew. Chem. Int. Edit.* **62**, e202309010 (2023).
R35. Sun, Y., Su, Y., Chai, Z., Jiang, L. & Heng, L. Flexible solid-liquid bi-continuous electrically and thermally conductive nanocomposite for electromagnetic interference shielding and heat dissipation. *Nat. Commun.* **15**, 7290 (2024).
R36. Ruan, K. et al. Molecular brush-grafted liquid crystalline hetero-structured fillers for boosting thermal conductivity of polyimide composite films. *Adv. Funct. Mater.* **35**, 2506563 (2025).
R37. Zhou, J. et al. Advanced functional Kevlar composite with excellent mechanical properties for thermal management and intelligent safeguarding. *Chem. Eng. J.* **428**, 131878 (2022).
R38. Li, Z. et al. Nacre-inspired MMT-MXene integrated shear-stiffening gel composites for personal safeguard and multi-functional electronics. *Compos. Part B:-Eng.* **280**, 111526 (2024).
R39. Wang, W. et al. Bio-inspired semi-active safeguarding design with enhanced impact resistance via shape memory effect. *Adv. Funct. Mater.* **33**, 2212093 (2023).
R40. Wang, W. et al. Thermo-mechanical behavior of shear stiffening elastomer via finite deformation temperature-dependent hyper-viscoelastic constitutive model. *Int. J. Appl. Mech.* **17**, 2550098 (2025).
R41. Zhao, N., Li, J., Wang, W., Gao, W. & Bai, H. Isotropically ultrahigh thermal conductive polymer composites by assembling anisotropic boron nitride nanosheets into a biaxially oriented network. *ACS Nano* **16**, 18959-18967 (2022).
R42. Lai, S. et al. Beetle elytra-inspired shear-stiffening warp-knitted spacer fabric composites with enhanced impact resistance. *Compos. Struct.* **370**, 119443 (2025).
R43. Cai, C. et al. Harnessing the Hofmeister effect for simultaneous strengthening and toughening of cellulosic triboelectric materials. *Angew. Chem. Int. Edit.* **65**, e21123 (2025).
R44. Sun, X., Mao, Y., Yu, Z., Yang, P. & Jiang, F. A biomimetic "salting out-alignment-locking" tactic to design strong and tough hydrogel. *Adv. Mater.* **36**, 2400084 (2024).
R45. Gao, H.-L. et al. Mass production of bulk artificial nacre with excellent mechanical properties. *Nat. Commun.* **8**, 287 (2017).
R46. Wu, N. et al. A binder-free ice template method for vertically aligned 3D boron nitride polymer composites towards thermal management. *J. Colloid Interf. Sci.* **647**, 43-51 (2023).
R47. Burger, N., Laachachi, A., Ferriol, M., Lutz, M., Toniazzo, V. & Ruch, D. Review of thermal conductivity in composites: Mechanisms, parameters and theory. *Prog. Polym. Sci.* **61**, 1-28 (2016).
R48. Han, C. et al. Fabrication of Curdlan connected honeycomb-like 3D BN-CNT network for enhancing the thermal conductivity of epoxy composites. *Chem. Eng. J.* **507**, 160443 (2025).
R49. Boland, C. S. et al. Sensitive electromechanical sensors using viscoelastic

graphene-polymer nanocomposites. *Science* **354**, 1257-1260 (2016).

Reply to Reviewer #1

Comment: The authors carefully revised and improved the manuscript. I recommend acceptance.

Our reply: We sincerely thank you for your positive evaluation and recommendation. We are grateful that the revisions have improved the manuscript, and we appreciate your constructive comments throughout the review process.

Reply to Reviewer #2

Comment: The authors have comprehensively addressed the major concerns raised in the previous review. In particular, the revised manuscript now provides (i) strengthened conceptual framing of the dynamic-response, multi-physics design principle, (ii) quantitative microstructure–property correlations supported by parametric modeling, (iii) independent validation of thermal conductivity via laser flash analysis, (iv) standardized impact metrics in accordance with ISO protocols, (v) detailed durability assessments including water, UV, and environmental cycling tests, and (vi) expanded life-cycle analysis with clarified system boundaries and practical functional units.

The additional experimental controls, benchmarking against state-of-the-art materials, and improved mechanistic explanations significantly enhance the scientific rigor and clarity of the work. The multifunctional integration is now convincingly demonstrated rather than descriptively presented, and the translational relevance is substantially strengthened.

In my opinion, the manuscript now meets the standards of Nature Communications in terms of novelty, depth, and technical completeness. I recommend acceptance.

Our reply: We sincerely thank you for your encouraging assessment and recommendation for acceptance. We are pleased that the added analyses, experimental controls, durability evaluations, and clarified mechanistic discussions have addressed the previous concerns and strengthened the manuscript. We thank you for the constructive feedback, which has greatly helped us improve the rigor, clarity, and overall quality of the work.

Reply to Reviewer #4

Comment: The authors have addressed all my concerns and I suggested acceptance of the manuscript.

Our reply: We sincerely thank you for your positive assessment and recommendation. We are grateful for your valuable comments, which helped us refine the manuscript and improve its overall presentation.

Reply to Reviewer #5

Comment: This manuscript presents a highly innovative and meticulously researched study on the development of a nacre-mimetic composite (STBC and its sandwich derivative, s-STBC) that synergistically integrates passive radiative cooling with kinetic impact defense. The integration of a dynamic chemical network with an ordered biomimetic architecture successfully overcomes the performance trade-offs typical of conventional composites. The authors have done an excellent job addressing the previous rounds of peer review, making the paper absolutely worth accepting. A decision of Minor Revision is recommended to allow the authors to finalize the formatting of their newly added figures and standardize the nomenclature before publication.

1. The revised manuscript and the accompanying response letter clearly articulate the fundamental principle of "dynamic-response and interface-mediated multi-physics synergistic regulation". By coupling a functional boron nitride nanosheet/cellulose nanocrystal (BNNS/CNC) skeleton with an environmentally responsive shear-stiffening gel (SSG) matrix, the authors have established a transferable design paradigm for polymer engineering that moves far beyond simple performance stacking.

2. The authors comprehensively addressed concerns regarding the causal link between dynamic crosslinking and impact resistance. The inclusion of Split Hopkinson Pressure Bar (SHPB) tests at a high strain rate of 2000 s^-1 and dynamic mechanical analysis (DMA) provides definitive quantitative evidence of the impact-hardening effect. The updated comparisons confirm that STBC significantly outperforms numerous state-of-the-art engineering materials in both maximum force and specific puncture energy.

3. The revisions highlighted in blue demonstrate exceptional methodological rigor. Validating the initial thermal conductivity measurements with laser-flash analysis (yielding 7.56 W m-1 K^-1 and confirming a thermal anisotropy ratio of 44.47) solidifies the claims regarding directional heat dissipation. Furthermore, the decoupling of passive thermal insulation from active synergistic cooling using Ecoflex and EPE foam control experiments elegantly proves the true synergy of the s-STBC design.

4. The extensive life-cycle assessment (LCA) boundary clarifications, alongside the newly added quantitative flammability metrics (e.g., an LOI of 69%), significantly elevate the practical relevance of this work. The outdoor cyclic aging tests encompassing UV, humidity, and temperature variations confirm the material's viability for protecting outdoor devices in extreme, prolonged environmental conditions.

5. While the scientific merit is now exceptionally solid, the authors should ensure that all newly replaced high-resolution SEM images and the standardized SI temperature metrics (Kelvin) are perfectly formatted and free of any residual typographical errors in the final galley proofs.

6. The authors should consider adding references related to passive radiation cooling materials to provide a broader context for their work. Some relevant references include:

https://doi.org/10.1016/j.energy.2025.136840

https://doi.org/10.1016/j.jobe.2025.113382

https://doi.org/10.1002/adsu.202500734
https://doi.org/10.1016/j.nanoen.2020.105600
https://doi.org/10.1021/acsami.5c14931

Our reply: We sincerely thank you for your careful and constructive suggestions. Following your advice, we have thoroughly checked all SEM images and standardized the units throughout the manuscript. The recommended references on passive radiative cooling materials have also been incorporated into the Introduction and Results section to provide a broader context for our work. These suggestions have not only improved the rigor and completeness of the manuscript, but also provided valuable inspiration for our future research.

Reply to Reviewer #6

Comment: The author has very well addressed all the questions raised by reviewer #3. Regarding the radiation cooling section, the current revised manuscript is basically free from any issues. Besides, it is to refer to the recent articles published in Nature Communications related to radiation cooling, such as Nature Communications, 2025, 16, 10533. Nature Communications, 2024, 15, 3076.

Our reply: We sincerely thank you for your positive evaluation and valuable suggestion. The recommended recent studies on radiative cooling have been added to the Introduction and Results section to better position our work within the current literature. These references also inspired us to further consider how this strategy may contribute to greener and more sustainable thermal-management technologies.
